# Electronic Cigarette Topography in the Natural Environment

**DOI:** 10.1371/journal.pone.0129296

**Published:** 2015-06-08

**Authors:** R. J. Robinson, E. C. Hensel, P. N. Morabito, K. A. Roundtree

**Affiliations:** Department of Mechanical Engineering, Rochester Institute of Technology, Rochester, New York, United States of America; Mario Negri Institute for Pharmacology Research, ITALY

## Abstract

This paper presents the results of a clinical, observational, descriptive study to quantify the use patterns of electronic cigarette users in their natural environment. Previously published work regarding puff topography has been widely indirect in nature, and qualitative rather than quantitative, with the exception of three studies conducted in a laboratory environment for limited amounts of time. The current study quantifies the variation in puffing behaviors among users as well as the variation for a given user throughout the course of a day. Puff topography characteristics computed for each puffing session by each subject include the number of subject puffs per puffing session, the mean puff duration per session, the mean puff flow rate per session, the mean puff volume per session, and the cumulative puff volume per session. The same puff topography characteristics are computed across all puffing sessions by each single subject and across all subjects in the study cohort. Results indicate significant inter-subject variability with regard to puffing topography, suggesting that a range of representative puffing topography patterns should be used to drive machine-puffed electronic cigarette aerosol evaluation systems.

## Introduction

### Background and Rationale

When low-yield cigarettes were introduced in the early 1970’s, the health community anticipated a significant reduction in lung cancer incidence that was never realized. In 1981, the Surgeon General reported that the Federal Trade Commission / International Standards Organization (FTC/ISO) cigarette testing protocols were inadequate and that no health claims could be made based on data generated by these standards. The FTC/ISO protocols, influenced largely by the tobacco industry [1] failed to account for compensatory behavior and were rescinded by the Federal Trade Commission (FTC) in 2008 [2].

Electronic cigarettes have become widely popular as a nicotine delivery system, yet there are no widely accepted testing standards to evaluate their health impact. Thus, although the Food and Drug Administration (FDA) now has authority to regulate health claims as part of the Family Smoking Prevention and Tobacco Control Act (TCA) of 2009, their ability to carry out the TCA is hindered by the lack of meaningful data and appropriate test protocols.

The ability to measure topography for conventional cigarette users has been widely demonstrated [3–13]. A review of electronic cigarette usage called for the need to measure electronic cigarette topography and frequency of use in the natural environment [14]. Conventional cigarette topography and smoking behavior have been widely studied [3–13], while electronic cigarette topography data has just recently begun to appear in the literature (Table 1). Puffing topography studies using video recordings of experienced users reported average puff durations of *4 seconds* [15, 16]. The CReSS device (Plowshare, Inc.) has been used to obtain electronic cigarette topography data in the lab environment with some limitations [17–19]. Specifically, one study reported device failure causing a loss of data for 15 of the subject sessions [18]. Another study reported inaccurate puff counts for 26% of the subjects tested for 10 min durations, because the CReSS Pocket is pre-set by the manufacturer to stop recording topography data after a user reaches 43 puffs [19]. Another study reported some device failure and significant loss of data for 15 of the subject sessions. Philip Morris is marketing the SMOKIO US Pat. (8,851,081 B2, 2014) with a smart phone app that reports puff intensity and nicotine exposure. An internet survey study of *81* experienced users reported frequency of use to be *175 puffs/day*, and in a later study of *3*,*587* experienced users [20], reported the average usage was *120 puffs/day* [15, 16][17–19]. What is lacking is a comprehensive understanding of use frequency (puffs per session, sessions per day), and topography (puff duration, puff volume and time between puffs) and a knowledge of cumulative exposure for users when allowed to puff in their natural environment. This study demonstrates a method to measure smoking topography of electronic cigarette users in their natural environment. Realistic smoking topographies will enhance our ability to estimate actual user exposures, adding perspective to the currently available electronic cigarette aerosol production data (Table 2) [17, 21–27].

**Table 1:** Previous studies involving electronic cigarette topography measurements.

Study	Number of Subjects	Testing Conditions	Puff Duration	Puff Volume	Time between Puffs	Flow Rate	Method
(1) Norton	N = 18	Lab	3.0 sec (SE 1.6 sec)	118.2-mL (SE 13.3 mL)	29.6 (SE 11.7)	*NR*	CReSS
(2) Behar	N = 20	Lab, *ad lib* puffing for 10 min	2.65 ± 0.98 sec	51 ± 21 mL	17.9 ± 7.5 sec	20 ± 6 mL/s	CReSS
(3) Goniewicz	N = 10	Lab, fixed number of puffs and time between sessions	1.8 ± 0.9 sec (0.3 sec)	70 ± 68 mL (21 mL)	10 ± 13 sec	*NR*	CReSS
(4) Farsalinos	N = 45	Lab, *ad lib* puffing for *20 min*	4.2 ± 0.7 sec1 (0.1 sec)	NR	20–30 sec	*NR*	Video recording
(5) Hua	N = 64	unknown	4.3 ± 1.5 sec (0.2 sec)	NR	NR	*NR*	YouTube videos

Studies included in each row of the table include (1) Norton [18], (2) Behar [19], (3) Goniewicz [17], (4) Farsalinos [16], and (5) Hua [15]. Values are reported as Mean ± Std. Dev. (SE Mean), NR = not reported. Note 1: Value represents puff duration plus inhalation time.

**Table 2:** Aerosol studies on electronic cigarettes and the puff topography used.

Study	Puff Duration	Puff Volume	Time between Puffs
(1) Westenberger	Not Reported	100 mL	Not Reported
(2) Trtchounian	2.2 sec	Not Reported	1 min
(3) Trehy	2 sec	100 mL	1 min
(4) Williams	2.2 sec	NR	1 min
(5) Goniewicz	1.8 sec	70 mL	10 sec
(6) Goniewicz	1.8 sec	70 mL	10 sec
(7) Goniewicz	1.8 sec	70 mL	10 sec
(8) Kosmider	1.8 sec	70 mL	17 sec

Studies included in each row of the table include (1) Westenberger [21], (2) Trtchounian [22], (3) Trehy [23], (4) Williams [24], (5) Goniewicz [17], (6) Goniewicz [25], (7) Goniewicz [26], and (8) Kosmider [27].

### Objectives

The current study aims to provide a better understanding of certain aspects of the puffing topography including puff duration, volume and flow rate, time between puffs and time between sessions. In addition, the study looks to quantify the variation in puffing behaviors among electronic cigarette users as well as the variation for a given user throughout the course of a day. This information will provide a rigorous analysis of topography and use in the natural environment, and enhance our understanding of actual nicotine aerosol exposure for these electronic cigarette users.

## Methods

### Study Design

This is an observational descriptive study involving electronic cigarette users. The study involved no intervention other than to request users to utilize a hand-held monitoring device for each puffing event for one *24 hour* period. The study involved students at the Rochester Institute of Technology (RIT), Henrietta campus and was completed over the course of six months, from Dec 2013 to May 2014.

### Cohort Recruitment and Protocol

The study protocol, including subject recruitment, informed consent, survey instrument, subject testing schedule, advertising, exclusion criteria and the study purpose were reviewed and approved by the Rochester Institute of Technology Human Subjects Research Office Institutional Review Board (IRB). The subject’s participation consisted of: written informed consent, survey, wireless Personal Use Monitor (wPUM) training, wPUM usage, and device return. RIT students were recruited to the study through the use of posters placed around campus. The posters advertised a research study regarding electronic cigarettes and stated that subjects would get a monetary incentive for participating. The poster also provided an email for the interested individuals to contact for more information. Everyone who sent an email to the address received the same response which provided a detailed summary of the study, and highlighted what was required of the subject if they chose to participate. They were also told that to participate in the study they must currently be a regular electronic cigarette user, and they would be paid *$50* if they participated and correctly fulfilled their requirements for the study. Finally, they were asked to reply via email if they were still interested in participating in the study.

Each test subject was scheduled a time to participate in the study based on their availability. The subjects were first surveyed on smoking history and behavior. Next, each subject attended an individual training session on how to use the wPUM, conducted in the Respiratory Technology Lab (RTL) at RIT. At the conclusion of the training session, each subject was provided with a packet of instructions for how to correctly use the monitor in case they forgot something covered during the training. The subjects were then provided with the wireless personal use monitor and a *1 day* supply of Blu Rechargeable electronic cigarettes and a summary of what they were required to do. The labeled nicotine level of the provided cigarettes was “approximately *16 mg*.” The subjects then left the RTL with the instructions to use the wPUM for each smoking event for *24 hours*. The subjects were asked to return all of the materials, including the wPUM and the electronic cigarettes to the RTL after the *24 hour* trial, at a pre-determined time, that was convenient for them.

### Wireless Personal Use Monitor

**Fig 1:**
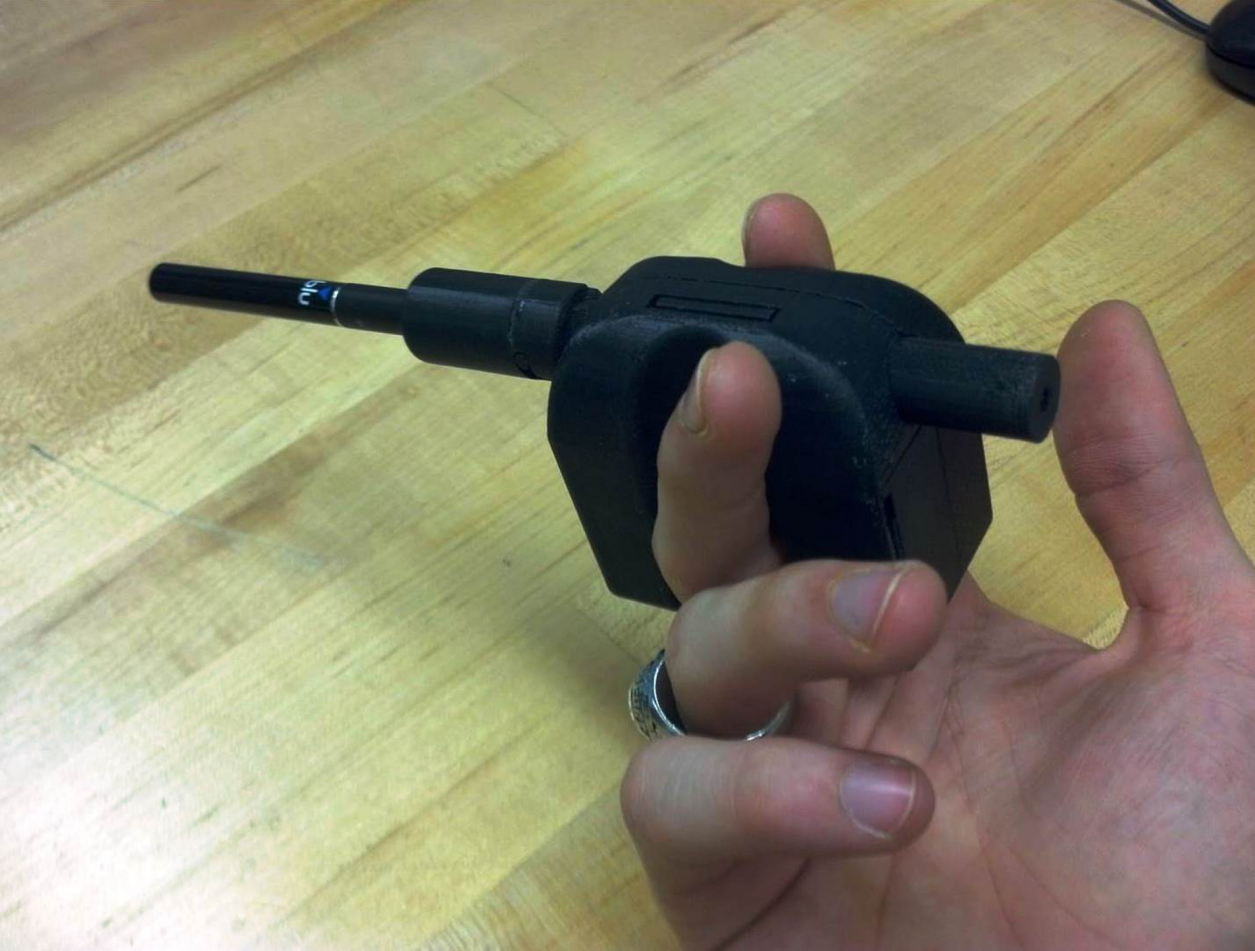
RIT wireless Personal Use Monitor (wPUM). The RIT wPUM is employed to record subject's use of electronic cigarettes in their natural environment.

Puffing topography was measured with a wireless personal use monitor (wPUM) designed, built and tested at RIT in collaboration with FSI Systems, Inc. The wPUM combines proven technologies to create one cohesive portable unit; an integral orifice plate and a differential pressure sensor, used to measure the change in dynamic pressure associated with flow through the orifice plate. Monitors were calibrated with an Alicat flow meter. The wPUM (Fig 1) is small and unobtrusive, with ergonomically designed finger grips, and a cigarette aperture that can accommodate different brand sizes. The wPUM automatically begins recording when the subject takes the first puff, to eliminate missed events due to user error. As fluid flows through the orifice plate a pressure difference is induced upon the differential pressure sensor. A transducer converts the differential pressure into an analog voltage, sampled with an analog to digital converter at *40 samples/ second*. This digitized signal is recorded as a digital file to the memory of the device, marked with date and time stamps. The wPUM is unique in its ability to record fine temporal detail of each puff taken as a function date and of time of day. Data is accumulated on-board the wPUM for the duration of time that the subject uses the monitor, and has memory capacity sufficient for one month of use. Each puffing session by the subject results in one data file stored on the wPUM.

### Puff Analysis

After the subject returns the device to the lab, the digital voltage and time stamp data files are exported from the wPUM memory to a computer. The data is analyzed using a processing code that performs the following sequence of digital signal processing operations. The wPUM digital voltage data is converted to a volumetric flow rate using an empirical equation, consistent with first principles of operation of an orifice plate flow meter, based upon the wPUM laboratory calibration. The start time of an individual puff is taken to be when the instantaneous discrete voltage exceeds a minimum threshold and the end time is taken to be when the instantaneous voltage descends below the same threshold. In an effort to eliminate the influence of discrete time sampling error but retain potentially significant subject topography characteristics, data processing was conducted to remove short-time (high frequency) events that are attributed to discrete time signal processing limitations associated with the Nyquist sampling rate. First, “raw” puffs with an intra-puff gap less than or equal a Nyquist threshold were merged into a "consolidated" puff. Next, any "consolidated" puffs having a duration below that expected by the Nyquist rate were neglected as being short time-duration events not associated with subject behavior, but attributed to being a manifestation of discrete time signal processing [28]. The remaining puffs were determined to be indicative of subject use-behavior, and included in the topography analysis.

**Fig 2:**
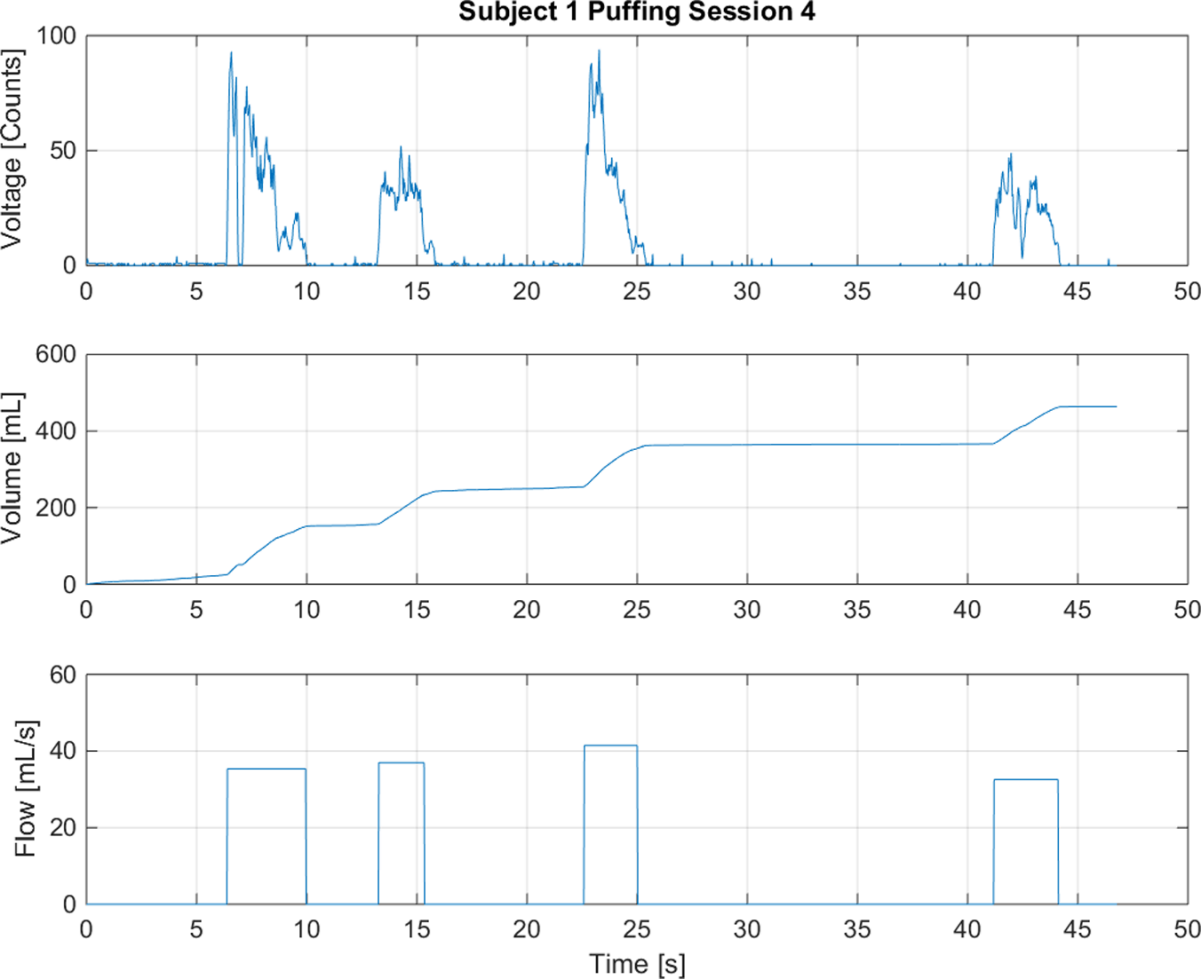
Puffing Profile. Puffing profile example obtained using the processing code to interpret data acquired with the RIT wireless Personal Use Monitor (wPUM).

The three puff topography characteristics computed for each discrete puff within a session are the per-puff duration, per-puff volume and the average flow rate. A sample of the results obtained for a single puffing session of a single subject is presented in Fig 2. The data is available as S1 File. The upper plot in Fig 2 illustrates the discrete sampled voltage data, expressed in A/D converter "counts" as a function of time for a single puffing session. This raw data is converted into an instantaneous flow rate and then integrated over time to provide the cumulative flow in the middle plot.

Four puff topography characteristics are computed for each puffing session by each subject. These include the number of subject puffs per puffing session, the mean puff duration per session, the mean puff flow rate per session, the mean puff volume per session, and the cumulative puff volume per session. Additional statistical descriptors including the standard deviation, standard error of the mean and variance are computed for each topographical characteristic for each puffing session. The same puff topography characteristics are computed across all sessions by each single subject (Subject Average) and across all subjects in the study cohort (Cohort Average).

## Results and Discussion

### Study Cohort

**Fig 3:**
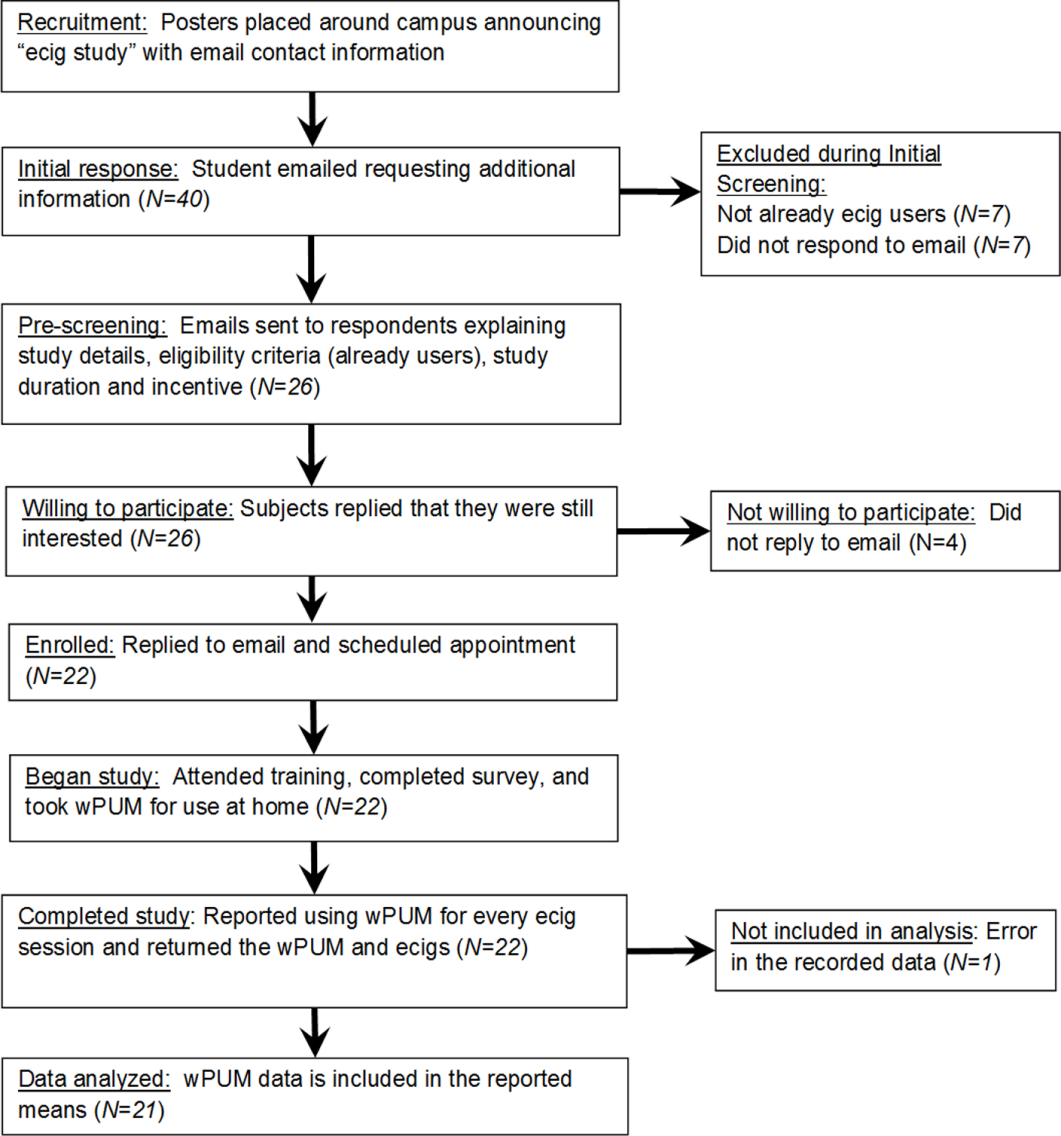
Cohort study flow chart. Forty individuals responded to the initial recruitment, from which data for 21 subjects is reported in this study.

A total of *40* individuals sent initial emails requesting more information about study participation. The flow of these *40* subjects and data through the study is shown in Fig 3. Of the original *40* subjects, *7* were ineligible because they were not electronic cigarette users, and another seven did not send reply confirmation emails saying they were still interested. From the remaining *26*, only *22* replied to subsequent emails and scheduled a date for participation. All *22* subjects who scheduled a participation date presented themselves at the appointed date and time. All *22* subjects completed the study and returned the wPUM and electronic cigarettes to the Respiratory Technology Lab to conclude their full one day trial. There was an error in the recording of data for one of these *22* subjects (subject number 19), causing their files to be lost, and so data was collected from a total of *21* subjects. Of these *21* subjects, *19* were male and two were female, and they ranged in ages from *18* to *29 years*. Two of the subjects had never smoked traditional cigarettes before, and ten of the subjects had smoked traditional cigarettes for at least one year. Of the *19* participants who had smoked traditional cigarettes before, nine had ceased smoking traditional cigarettes and one continues to smoke traditional cigarettes on a daily basis. Six participants reported previously using a form of nicotine replacement therapy, such as a nicotine patch or gum. Eleven participants reported that they started using electronic cigarettes to either quit smoking or prevent themselves from resuming smoking.

### Puff Topography for Electronic Cigarette Users

Puff topography characteristics for each subject and the cohort average are presented in Table 3. The puffing sessions were analyzed in detail to gather insight into inter-subject variability (differences between subjects in the cohort). A total of 309 puffing sessions and 4,723 puffs were analyzed. With the exception of 1 subject out of 22, the device functioned as expected with no loss of data. The mean puff duration, puff flow rate volume, and puff volume varied significantly across the cohort. Subject average mean puff duration ranged from *0*.*9 sec to 6*.*9 sec*. Subject average mean puff flow rate ranged from *23 mL/sec* to *102 mL/sec*, and subject average mean puff volume varied from *29 mL to 388 mL*. Low standard error of the mean (SE Mean) indicates that the data gathered for each subject over the *24 hour* period was sufficient to characterize the mean values for the subjects’ topography. The SE Mean for each subject was less than *1%* of the nominal value for all reported data except for the puff duration and puff volume for subjects 4, 6 and 8, where the SE Mean was just over *1%* of the nominal value.

**Table 3:** Subject and Cohort Average Puffing Topography Characteristics.

	Mean Puff Duration *(s)*			Mean Puff Flow rate *(mL/s)*			Mean Puff Volume *(mL)*		
Subject	Mean	SE Mean	Std. Dev.	Mean	SE Mean	Std. Dev.	Mean	SE Mean	Std. Dev.
1	0.9	1.0E-03	1.0	24	1.1E-02	12	29	3.4E-02	37
2	2.3	6.0E-03	1.2	35	3.7E-02	8	83	2.2E-01	48
3	4.0	2.6E-02	2.2	34	8.5E-02	7	144	1.1E+00	96
4	0.7	1.0E-02	0.5	41	2.4E-01	13	29	3.6E-01	19
5	2.3	1.0E-02	1.3	30	6.2E-02	8	72	3.5E-01	44
6	5.1	8.7E-02	9.1	28	1.5E-01	15	128	1.5E+00	160
7	6.9	1.3E-02	3.2	44	4.8E-02	13	310	6.8E-01	175
8	4.8	6.4E-02	1.5	36	2.8E-01	7	167	1.9E+00	45
9	1.9	8.0E-03	0.5	42	1.5E-01	9	77	4.7E-01	27
10	4.8	2.0E-02	2.1	40	8.5E-02	9	200	1.0E+00	107
11	6.7	2.4E-02	3.1	32	4.0E-02	5	219	8.6E-01	108
12	1.4	4.0E-03	0.8	30	4.1E-02	9	44	1.4E-01	30
13	2.9	1.7E-02	1.0	33	1.1E-01	7	97	6.7E-01	40
14	2.5	1.5E-02	1.5	39	8.9E-02	9	97	8.0E-01	82
15	4.5	1.3E-02	1.7	34	4.8E-02	6	147	4.1E-01	52
16	3.7	5.0E-03	2.0	34	2.3E-02	10	129	1.9E-01	80
17	3.7	3.0E-03	1.6	102	7.0E-02	33	388	4.8E-01	223
18	4.9	4.0E-03	3.0	23	8.0E-03	7	115	8.0E-02	66
20	5.2	2.8E-02	2.0	35	5.4E-02	4	179	9.9E-01	70
21	2.3	1.1E-02	0.6	29	8.4E-02	5	65	3.4E-01	20
22	2.2	3.0E-03	0.5	33	4.3E-02	6	70	9.2E-02	12
Cohort Average	3.5	3.9E-01	1.8	37	3.5E+00	16	133	2.0E+01	90

Topography characteristics from the current study were compared to the limited currently available topography data. The cohort-mean puff duration of *3*.*5 ± 0*.*39 sec* from this study fell within the range of *1*.*8 ± 0*.*3 sec* [17] and *4*.*3 ±* 0.2 sec [15, 16] of previous reported estimates. The cohort-mean puff volume of *133 ± 2 mL* from the current study was larger than the previously reported values of *70 ± 21 mL* [17, 18, 19] measured in the laboratory environment. The SE Mean of puff volume in the current study was *1*.*5%* of the nominal value, which is much lower than the SE Mean (*30%* of the nominal value [17]) of the data currently used in aerosol production studies [17, 25–27]. Data to compare the cohort-mean puff flow rate from this study to previous studies was not available.

**Fig 4:**
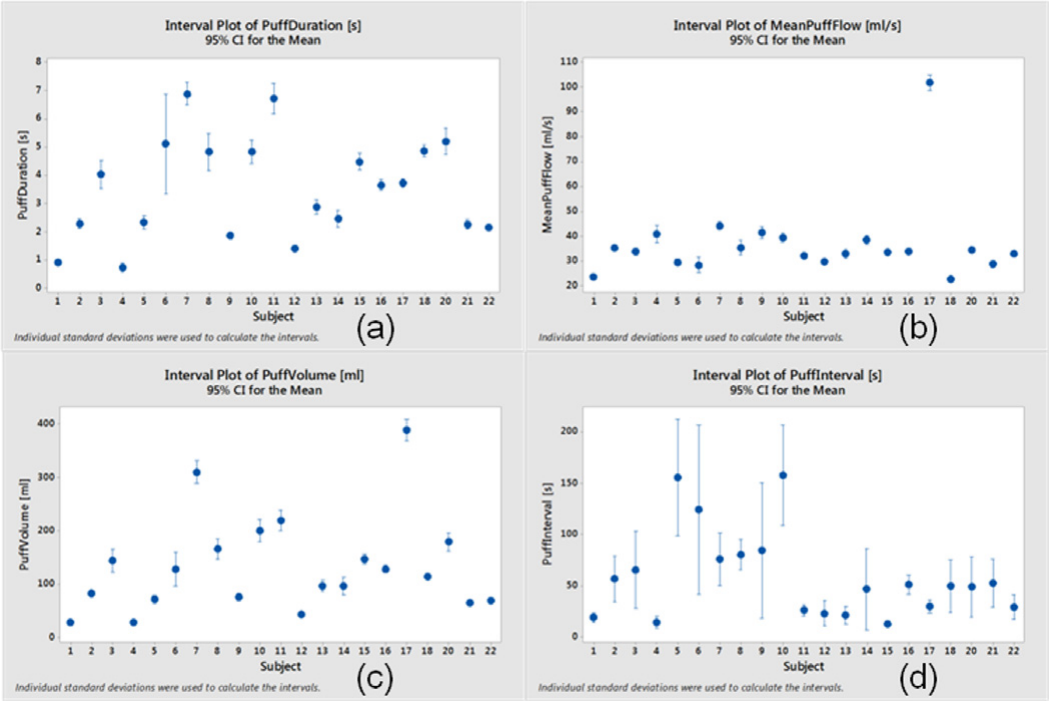
Interval Plots of Topography Characteristics. Interval plot subject means with 95% CI around the mean for (a) puff duration, (b) puff flow rate, (c) puff volume, and (d) time between puffs.

A one-way ANOVA (Minitab V.17, Minitab Inc.) with Tukey method of multiple comparisons of each topography characteristic versus subject indicated that the variation in means across the cohort was significant for puff volume and puff duration, but was not significant for puff interval. Interval plots for topography presented in Fig 4 for *95% CI* (confidence interval) illustrate the degree of inter-subject variability. The Fig 4 data is available as S2 File. Panel 4a illustrates the subject variability in the subject mean puff duration with variability as much as seven to one, suggesting that individuals use electronic cigarettes in a variety of manners. Although subjects 5, 12 and 17 had not previously been conventional cigarette smokers, their puff durations were not outside the range of other electronic cigarettes users tested in this study. The subject mean puff flow rate, shown in panel 4b, generally exhibits lower variability between subjects, on the order of two to one, with the exception of Subject 17, who exhibited behaviors atypical of the cohort. The other two non-smokers (subjects 5 and 7) exhibited flow rates typical of the norm. The subject mean puff volume is illustrated in panel 4c, again demonstrating significant user variation. Subject 17 exhibits uncharacteristic behavior in both puff flow rate and puff volume. The time history of puffing sessions associated with Subject 17 further suggests this subject to be atypical of the cohort. However, the data does not suggest subject 17’s atypical behavior is a result of being a non-smoker, since the other non-smokers (subjects 5 and 12) do not exhibit similar behavior. Subject 7 exhibits a relatively high puff volume, corresponding to a relatively long puff duration and flow rate. Subject 12, conversely, exhibits a relatively low puff volume, corresponding to a relatively short puff duration and moderate puff flow rate. Subject 1 exhibits atypical usage characteristics relative to the cohort with an unusually low puff duration, flow rate, volume and interval.

**Fig 5:**
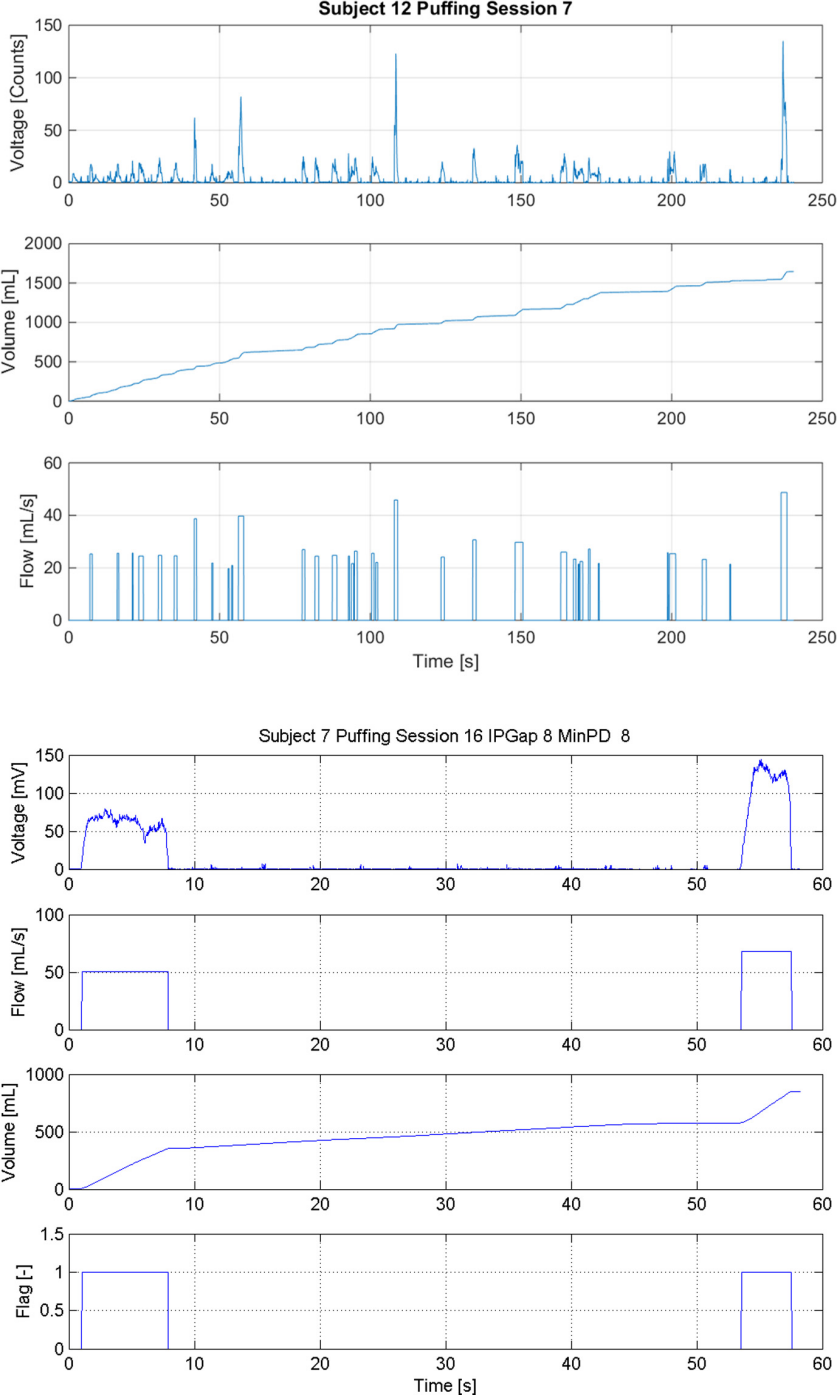
Subject Usage Patterns. Exemplar usage pattern topography (a) Subject 12 tended to take several puffs of short duration during a single session, (b) Subject 7 tended to take fewer puffs of longer duration during a single session.

**Fig 6:**
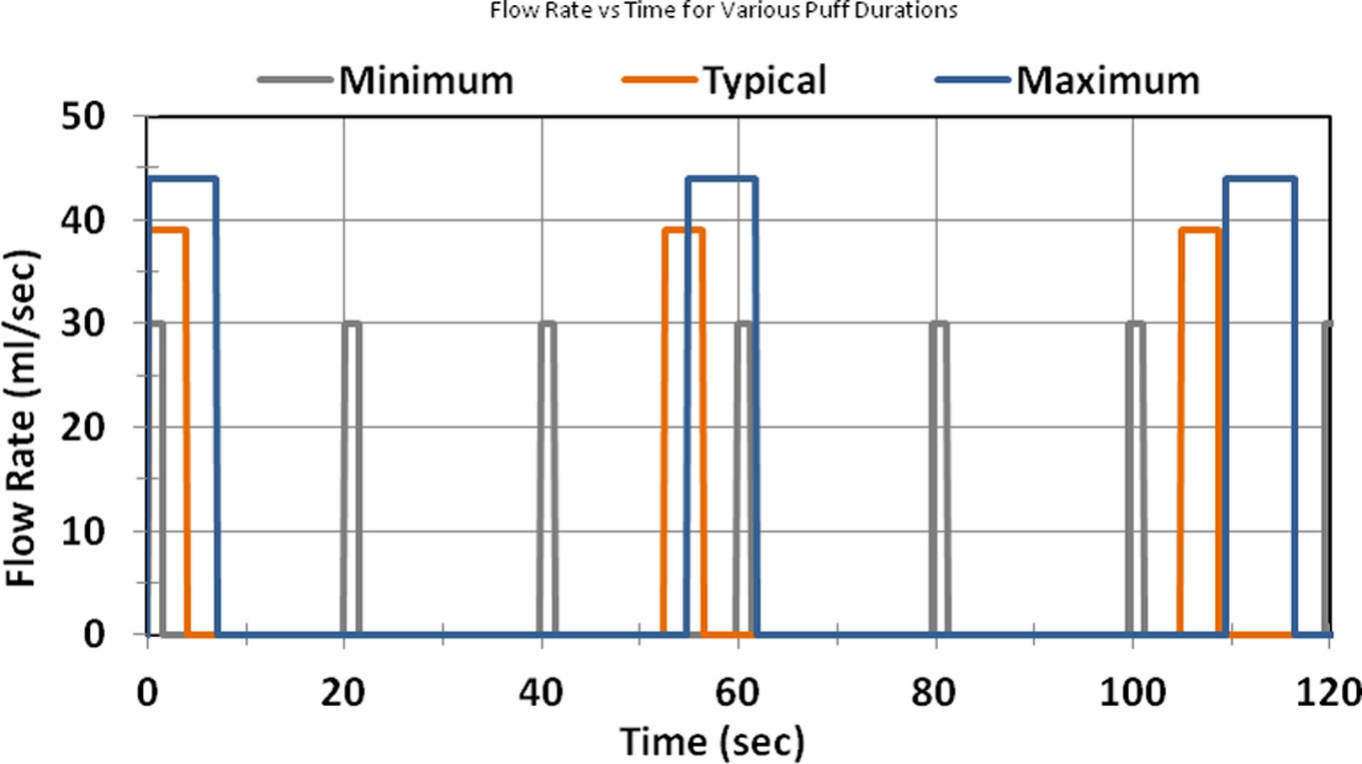
Programmable Electronic Cigarette Topographies. Natural environment topographies for three exemplar puff duration cases. These waveforms can be programmed into electronic cigarette aerosol evaluation systems to assess nicotine aerosol production testing.

Significant inter-subject variability indicates that further analysis to determine the effect of inter-subject variability of topography on electronic cigarette aerosol production is justified. The quantitative ANOVA Tukey comparisons, as well as a qualitative analysis of each subjects’ puffing characteristics, were done to choose natural-environment topographies representative of the electronic cigarette users monitored in this study. Exemplar topography profiles for Subject 12 and Subject 7 are illustrated in Fig 5, panel (a) and panel (b) respectively. The Fig 5 data is available as S3 and S4 Files. Puffing session number 7 for Subject 12 illustrates a common scenario observed among certain users, of taking several puffs of relatively short duration during a session. Puffing session number 16 for Subject 7, conversely, illustrates a scenario observed among users tending to take fewer discrete puffs of longer duration during a session. These topography differences became apparent when comparing the time histories across the entire cohort of *21* subjects and the numerous puffing sessions by each. After evaluation of individual responses of all *21* subjects across more than *300* puffing sessions, three characteristic use-cases were observed, hereinafter referred to as "Many Short" duration puffs, "Typical" duration and quantity of puffs, and "Fewer Long" duration puffs. The "Typical" use-case represents the cohort attributes averaged across several users, while the "Many Short" use-case represents the subject mean topography of Subject 12, and the "Fewer Long" use-case represents the subject mean topography of Subject 7. The topographies of three representative use-cases are presented in Table 4, and illustrated in Fig 6. Note that the puff period is defined as the summation of the puff duration and the puff interval, while the puff volume is the product of the puff duration and the puff flow rate.

**Table 4:** Three representative puff topographies of subjects using electronic cigarettes in their natural environment.

Indicative Subject(s)	Puff Duration Case	Puff Duration (s)	Flow Rate *(mL/s)*	Puff Volume *(mL)*	Puff Interval *(s)*	Puff Period *(s)*
1, 4, 9, 12	Many Short	1.4	30	42	18.4	19.8
2, 3, 5, 8, 10, 13, 14, 15, 16, 17, 18, 21, 22	Typical	3.7	39	144	48.7	52.4
6, 7, 11, 20	Fewer Long	6.9	44	304	47.7	54.6

### Frequency of Use and Cumulative Exposure from Ecig Use

Table 5 provides the total volume of nicotine aerosol inhaled in a *24 hour* period for each subject. Exposure varies widely from subject to subject, and does not appear to be affected by whether or not the subjects had previously smoked conventional cigarettes. The mean total number of puffs ranges from *24 puffs/day* to over *1000 puffs/day*, with a mean of *225 ± 59 puffs/day* (mean ± SE Mean). Compared to self-report surveys, this current study found that total puffs per day measured in the natural environment was almost twice that reported in survey data [14, 20]. Because the puff volume and total number of puffs taken varied widely across the cohort, the cumulative volume of nicotine aerosol was determine for each subject. The mean cumulative volume of nicotine aerosol inhaled over a *24 hour* period under natural conditions ranged from *3*.*7 liters* to over *182 liters* of aerosol, with a mean of *29*.*7 ± 30 liters* (mean ± SE Mean). The cumulative volume is not directly comparable to other studies. The total number of discrete puffing sessions indicated by the wPUM data is also reported in Table 5, along with the calculated average number of puffs/session and average nicotine aerosol inhaled per session. A regression analysis indicated that total puffs taken in a *24 hour* period is not a predictor for cumulative volume inhaled, nor are the number of sessions or number of puffs per session. Less than *66%* of the variation in cumulative puff volume can be explained by a linear regression model in puff number, indicating that cumulative exposure may be better determined by measuring aerosol in a usage profile representative of subjects' electronic cigarette use in the natural environment.

**Table 5:** Cumulative electronic cigarette use and e-liquid aerosol exposure in a *24 hr*. period measured in the user’s natural environment.

	Exposure in *1 day*			Average exposure per session	
Subject	Sessions *(-)*	Total puffs *(-)*	Total volume *(mL)*	Number of puffs *(-/session)*	Volume (mL/session)
1	26	1,091	31,820	42	1,224
2	13	216	17,908	17	83
3	12	84	12,102	7	144
4	5	53	1,553	11	29
5	41	126	9,074	3	72
6	7	105	13,448	15	128
7	59	258	79,921	4	310
8	5	24	4,000	5	167
9	14	58	4,452	4	77
10	30	106	21,235	4	200
11	6	126	27,611	21	219
12	7	213	9,448	30	44
13	7	60	5,839	9	97
14	5	103	10,000	21	97
15	11	128	18,870	12	147
16	13	420	54,081	32	129
17	16	469	182,147	29	388
18	7	821	94,627	117	115
20	12	71	12,741	6	179
21	7	57	3,723	8	65
22	6	134	9,437	22	70
Cohort Ave.	15	225	29,716	15	132
Std. Dev.	14	272	29,616	25	252
SE Mean	3	59	30,173	5	55

## Conclusions

The current study is the first to present topography and frequency of use data for electronic cigarette users in their natural environment for a 24 hour period. By monitoring subjects over an entire day, rather than a fixed time in the laboratory environment, the data provides a more holistic view of the subjects’ actual frequency of use than previous studies. Furthermore, the wPUM does not set a limit on the number of allowed puffs per session, which has been reported by studies using the commercially available CReSS device.

A total of 295 sessions and 4,723 puffs were analyzed for *21* subjects who used Blu electronic rechargeable cigarettes in their natural environment for *24 hours*. Results indicate significant inter-subject variability with regard to puffing topography, suggesting that a range of representative puffing topography patterns should be used to drive aerosol production evaluation systems.

There may be a tendency to estimate total exposure based on the “cohort-average” puff volume and the “cohort-average” puffs taken per day. The natural environment topography use characteristics presented herein demonstrate that electronic cigarette users vary widely in the manner in which they puff. It is expected that aerosol production measured with an exemplar electronic cigarette are strongly dependent upon usage topography. Therefore, it is unreasonable to assume that mean values of cohort topography characteristics may be used to accurately estimate the aerosol exposure of individual subjects.

The standard deviations observed in puff topography characteristics indicate that individual subject puffing patterns varied significantly over the course of a *24 hour* period. A detailed statistical analysis is needed to determine the effect of daily patterns as reflected in the reported large standard deviations, but the small reported values of SE Mean indicate that sample sizes were sufficiently large to estimate the mean values. The relatively large standard deviations associated with each subject's puffing topography characteristics indicate more accurate aerosol exposure estimates may be obtained from evaluations utilizing the entire *24 hour* instantaneous puffing behavior protocol, rather than the cohort mean values or potentially, subject mean values.

The topography results provided in this study should not be generalized to other brands or other electronic cigarette designs. Other brands should be tested and compared to determine the effect of device design on puffing topography. Finally, the ultimate goal is to devise a machine-yield testing protocol that can be used in regulatory science to devise a testing protocol for laboratory aerosol production studies. Therefore, results of realistic topographies such as those presented in this study could be utilized to drive smoking machine aerosol production tests.

## Supporting Information

S1 File Data underlying Fig 2. This file contains the data used to generate Fig 2, describing Subject 1, Puffing Session 4.
(PDF)

S2 File Data underlying Fig 4. This file contains the data used to generate Fig 4, describing the puff topography.
(PDF)

S3 File Data underlying Fig 5. This file contains the data used to generate Fig 5, Panel A, describing Subject 12, Puffing Session 7.
(PDF)

S4 File Data underlying Fig 5. This file contains the data used to generate Fig 5, Panel B, describing Subject 7, Puffing Session 16.
(PDF)
