# Supplementary material for: Electronic Cigarette Topography in the Natural Environment
Source: PLoS One. 2015 Jun 8;10(6):e0129296. doi: 10.1371/journal.pone.0129296 (PMC4460076; doi:10.1371/journal.pone.0129296)
Supplement: S1 File — This file contains the data used to generate Fig 2, describing Subject 1, Puffing Session 4. (PDF) [file pone.0129296.s001.pdf]

RelativeTime,CleanData,AccumulatedVolume,MeanPuffFlowRate,PuffFlag
0,1,0.16425,0,0
0.025,1,0.3285,0,0
0.05,3,0.552869672571594,0,0
0.075,1,0.777239345143188,0,0
0.1,1,0.941489345143188,0,0
0.125,1,1.10573934514319,0,0
0.15,1,1.26998934514319,0,0
0.175,1,1.43423934514319,0,0
0.2,1,1.59848934514319,0,0
0.225,1,1.76273934514319,0,0
0.25,1,1.92698934514319,0,0
0.275,1,2.09123934514319,0,0
0.3,1,2.25548934514319,0,0
0.325,1,2.41973934514319,0,0
0.35,1,2.58398934514319,0,0
0.375,1,2.74823934514319,0,0
0.4,1,2.91248934514319,0,0
0.425,1,3.07673934514319,0,0
0.45,0,3.15886434514319,0,0
0.475,1,3.24098934514319,0,0
0.5,0,3.32311434514319,0,0
0.525,1,3.40523934514319,0,0
0.55,1,3.56948934514319,0,0
0.575,1,3.73373934514319,0,0
0.6,1,3.89798934514319,0,0
0.625,1,4.06223934514319,0,0
0.65,1,4.22648934514319,0,0
0.675,1,4.39073934514319,0,0
0.7,1,4.55498934514319,0,0
0.725,1,4.71923934514319,0,0
0.75,1,4.88348934514319,0,0
0.775,1,5.04773934514319,0,0
0.8,1,5.21198934514319,0,0
0.825,1,5.37623934514319,0,0
0.85,1,5.54048934514319,0,0
0.875,0,5.62261434514319,0,0
0.9,0,5.62261434514319,0,0
0.925,0,5.62261434514319,0,0
0.950000000000001,1,5.70473934514319,0,0
0.975000000000001,1,5.86898934514319,0,0
1,0,5.95111434514319,0,0
1.025,0,5.95111434514319,0,0
1.05,0,5.95111434514319,0,0
1.075,1,6.03323934514319,0,0
1.1,1,6.19748934514319,0,0
1.125,1,6.36173934514319,0,0
1.15,1,6.52598934514319,0,0
1.175,1,6.69023934514319,0,0
1.2,0,6.77236434514319,0,0
1.225,0,6.77236434514319,0,0

1.25,0,6.77236434514319,0,0
1.275,0,6.77236434514319,0,0
1.3,0,6.77236434514319,0,0
1.325,0,6.77236434514319,0,0
1.35,0,6.77236434514319,0,0
1.375,0,6.77236434514319,0,0
1.4,1,6.85448934514319,0,0
1.425,0,6.93661434514319,0,0
1.45,1,7.01873934514319,0,0
1.475,1,7.18298934514319,0,0
1.5,1,7.34723934514319,0,0
1.525,0,7.42936434514319,0,0
1.55,0,7.42936434514319,0,0
1.575,1,7.51148934514319,0,0
1.6,1,7.67573934514319,0,0
1.625,1,7.83998934514319,0,0
1.65,0,7.92211434514319,0,0
1.675,1,8.00423934514319,0,0
1.7,0,8.08636434514318,0,0
1.725,0,8.08636434514318,0,0
1.75,1,8.16848934514318,0,0
1.775,0,8.25061434514318,0,0
1.8,0,8.25061434514318,0,0
1.825,0,8.25061434514318,0,0
1.85,0,8.25061434514318,0,0
1.875,1,8.33273934514318,0,0
1.9,1,8.49698934514318,0,0
1.925,0,8.57911434514318,0,0
1.95,0,8.57911434514318,0,0
1.975,0,8.57911434514318,0,0
2,0,8.57911434514318,0,0
2.025,0,8.57911434514318,0,0
2.05,0,8.57911434514318,0,0
2.075,0,8.57911434514318,0,0
2.1,0,8.57911434514318,0,0
2.125,0,8.57911434514318,0,0
2.15,0,8.57911434514318,0,0
2.175,1,8.66123934514318,0,0
2.2,0,8.74336434514318,0,0
2.225,0,8.74336434514318,0,0
2.25,0,8.74336434514318,0,0
2.275,0,8.74336434514318,0,0
2.3,0,8.74336434514318,0,0
2.325,0,8.74336434514318,0,0
2.35,0,8.74336434514318,0,0
2.375,0,8.74336434514318,0,0
2.4,0,8.74336434514318,0,0
2.425,0,8.74336434514318,0,0
2.45,0,8.74336434514318,0,0
2.475,0,8.74336434514318,0,0
2.5,0,8.74336434514318,0,0
2.525,0,8.74336434514318,0,0

2.54999999999999,1,8.82548934514318,0,0
2.57499999999999,0,8.90761434514318,0,0
2.59999999999999,0,8.90761434514318,0,0
2.62499999999999,0,8.90761434514318,0,0
2.64999999999999,1,8.98973934514318,0,0
2.67499999999999,1,9.15398934514318,0,0
2.69999999999999,0,9.23611434514318,0,0
2.72499999999999,0,9.23611434514318,0,0
2.74999999999999,0,9.23611434514318,0,0
2.77499999999999,0,9.23611434514318,0,0
2.79999999999999,0,9.23611434514318,0,0
2.82499999999999,0,9.23611434514318,0,0
2.84999999999999,0,9.23611434514318,0,0
2.87499999999999,1,9.31823934514318,0,0
2.89999999999999,0,9.40036434514318,0,0
2.92499999999999,0,9.40036434514318,0,0
2.94999999999999,1,9.48248934514318,0,0
2.97499999999999,1,9.64673934514318,0,0
2.99999999999999,1,9.81098934514318,0,0
3.02499999999999,1,9.97523934514318,0,0
3.04999999999999,1,10.1394893451432,0,0
3.07499999999999,1,10.3037393451432,0,0
3.09999999999999,0,10.3858643451432,0,0
3.12499999999999,0,10.3858643451432,0,0
3.14999999999999,0,10.3858643451432,0,0
3.17499999999999,0,10.3858643451432,0,0
3.19999999999999,0,10.3858643451432,0,0
3.22499999999999,0,10.3858643451432,0,0
3.24999999999999,0,10.3858643451432,0,0
3.27499999999999,0,10.3858643451432,0,0
3.29999999999999,0,10.3858643451432,0,0
3.32499999999999,1,10.4679893451432,0,0
3.34999999999999,1,10.6322393451432,0,0
3.37499999999999,1,10.7964893451432,0,0
3.39999999999999,1,10.9607393451432,0,0
3.42499999999999,0,11.0428643451432,0,0
3.44999999999999,1,11.1249893451432,0,0
3.47499999999999,1,11.2892393451432,0,0
3.49999999999999,0,11.3713643451432,0,0
3.52499999999999,0,11.3713643451432,0,0
3.54999999999999,1,11.4534893451432,0,0
3.57499999999999,1,11.6177393451432,0,0
3.59999999999999,1,11.7819893451432,0,0
3.62499999999999,1,11.9462393451432,0,0
3.64999999999999,0,12.0283643451432,0,0
3.67499999999999,1,12.1104893451432,0,0
3.69999999999999,1,12.2747393451432,0,0
3.72499999999999,1,12.4389893451432,0,0
3.74999999999999,0,12.5211143451432,0,0
3.77499999999999,1,12.6032393451432,0,0
3.79999999999999,1,12.7674893451432,0,0
3.82499999999999,1,12.9317393451432,0,0

3.84999999999999,0,13.0138643451432,0,0
3.87499999999999,0,13.0138643451432,0,0
3.89999999999999,0,13.0138643451432,0,0
3.92499999999999,0,13.0138643451432,0,0
3.94999999999999,1,13.0959893451432,0,0
3.97499999999999,1,13.2602393451432,0,0
3.99999999999999,0,13.3423643451432,0,0
4.02499999999999,1,13.4244893451432,0,0
4.04999999999999,1,13.5887393451432,0,0
4.07499999999999,1,13.7529893451432,0,0
4.09999999999999,4,13.9993643451432,0,0
4.12499999999999,1,14.2457393451432,0,0
4.14999999999999,1,14.4099893451432,0,0
4.17499999999999,1,14.5742393451432,0,0
4.19999999999999,1,14.7384893451432,0,0
4.22499999999999,1,14.9027393451432,0,0
4.24999999999999,1,15.0669893451432,0,0
4.27499999999999,1,15.2312393451432,0,0
4.29999999999999,1,15.3954893451432,0,0
4.32499999999999,1,15.5597393451432,0,0
4.34999999999999,0,15.6418643451432,0,0
4.37499999999999,0,15.6418643451432,0,0
4.4,0,15.6418643451432,0,0
4.425,0,15.6418643451432,0,0
4.45,0,15.6418643451432,0,0
4.475,0,15.6418643451432,0,0
4.5,0,15.6418643451432,0,0
4.525,0,15.6418643451432,0,0
4.55,2,15.758006633953,0,0
4.575,0,15.8741489227629,0,0
4.6,0,15.8741489227629,0,0
4.625,0,15.8741489227629,0,0
4.65,1,15.9562739227629,0,0
4.675,1,16.1205239227629,0,0
4.7,1,16.2847739227629,0,0
4.725,1,16.4490239227629,0,0
4.75,1,16.6132739227629,0,0
4.775,1,16.7775239227629,0,0
4.8,1,16.9417739227629,0,0
4.825,1,17.1060239227629,0,0
4.85,1,17.2702739227629,0,0
4.875,1,17.4345239227629,0,0
4.9,1,17.5987739227629,0,0
4.925,1,17.7630239227629,0,0
4.95,1,17.9272739227629,0,0
4.975,1,18.0915239227629,0,0
5,0,18.1736489227629,0,0
5.025,1,18.2557739227629,0,0
5.05,1,18.4200239227629,0,0
5.075,1,18.5842739227629,0,0
5.10000000000001,1,18.7485239227629,0,0
5.12500000000001,1,18.9127739227629,0,0

5.15000000000001,1,19.0770239227629,0,0
5.17500000000001,1,19.2412739227629,0,0
5.20000000000001,1,19.4055239227629,0,0
5.22500000000001,1,19.5697739227629,0,0
5.25000000000001,1,19.7340239227629,0,0
5.27500000000001,1,19.8982739227629,0,0
5.30000000000001,1,20.0625239227629,0,0
5.32500000000001,1,20.2267739227629,0,0
5.35000000000001,0,20.3088989227629,0,0
5.37500000000001,1,20.3910239227629,0,0
5.40000000000001,1,20.5552739227629,0,0
5.42500000000001,1,20.7195239227629,0,0
5.45000000000001,0,20.8016489227629,0,0
5.47500000000001,1,20.8837739227629,0,0
5.50000000000001,1,21.0480239227629,0,0
5.52500000000001,1,21.2122739227629,0,0
5.55000000000001,0,21.2943989227629,0,0
5.57500000000001,1,21.3765239227629,0,0
5.60000000000001,0,21.4586489227629,0,0
5.62500000000001,0,21.4586489227629,0,0
5.65000000000001,1,21.5407739227629,0,0
5.67500000000001,1,21.7050239227629,0,0
5.70000000000001,0,21.7871489227629,0,0
5.72500000000001,0,21.7871489227629,0,0
5.75000000000001,0,21.7871489227629,0,0
5.77500000000001,0,21.7871489227629,0,0
5.80000000000001,0,21.7871489227629,0,0
5.82500000000002,1,21.8692739227629,0,0
5.85000000000002,1,22.0335239227629,0,0
5.87500000000002,1,22.1977739227629,0,0
5.90000000000002,0,22.2798989227629,0,0
5.92500000000002,1,22.3620239227629,0,0
5.95000000000002,1,22.5262739227629,0,0
5.97500000000002,1,22.6905239227629,0,0
6.00000000000002,1,22.8547739227629,0,0
6.02500000000002,1,23.0190239227629,0,0
6.05000000000002,1,23.1832739227629,0,0
6.07500000000002,1,23.3475239227629,0,0
6.10000000000002,1,23.5117739227629,0,0
6.12500000000002,1,23.6760239227629,0,0
6.15000000000002,1,23.8402739227629,0,0
6.17500000000002,1,24.0045239227629,0,0
6.20000000000002,1,24.1687739227629,0,0
6.22500000000002,1,24.3330239227629,0,0
6.25000000000002,1,24.4972739227629,0,0
6.27500000000002,1,24.6615239227629,0,0
6.30000000000002,0,24.7436489227629,0,0
6.32500000000002,0,24.7436489227629,0,0
6.35000000000002,0,24.7436489227629,0,0
6.37500000000002,6,24.944813267889,0,0
6.40000000000002,23,25.5398352768671,35.3558562783496,1
6.42500000000002,40,26.4530970464018,35.3558562783496,1

6.45000000000002,61,27.6139179067183,35.3558562783496,1
6.47500000000002,74,28.961800623908,35.3558562783496,1
6.50000000000002,84,30.4209546443604,35.3558562783496,1
6.52500000000003,86,31.9352386211995,35.3558562783496,1
6.55000000000003,89,33.4716002406122,35.3558562783496,1
6.57500000000003,93,35.0383507598289,35.3558562783496,1
6.60000000000003,87,36.5963465833104,35.3558562783496,1
6.62500000000003,80,38.096905918654,35.3558562783496,1
6.65000000000003,66,39.4986409032434,35.3558562783496,1
6.67500000000003,59,40.7966416517678,35.3558562783496,1
6.70000000000003,56,42.0420229720891,35.3558562783496,1
6.72500000000003,62,43.30324309452,35.3558562783496,1
6.75000000000003,75,44.6611193540312,35.3558562783496,1
6.77500000000003,76,46.0882918683657,35.3558562783496,1
6.80000000000003,82,47.5479145243118,35.3558562783496,1
6.82500000000003,63,48.9434350080449,35.3558562783496,1
6.85000000000003,38,50.1015344873022,35.3558562783496,1
6.87500000000003,10,50.8674890401374,35.3558562783496,1
6.90000000000003,0,51.1271910929787,35.3558562783496,1
6.92500000000003,0,51.1271910929787,35.3558562783496,1
6.95000000000003,0,51.1271910929787,35.3558562783496,1
6.97500000000003,1,51.2093160929787,35.3558562783496,1
7.00000000000003,0,51.2914410929787,35.3558562783496,1
7.02500000000003,0,51.2914410929787,35.3558562783496,1
7.05000000000003,0,51.2914410929787,35.3558562783496,1
7.07500000000003,1,51.3735660929787,35.3558562783496,1
7.10000000000003,10,51.71539314582,35.3558562783496,1
7.12500000000003,25,52.3857201986614,35.3558562783496,1
7.15000000000003,40,53.315749304344,35.3558562783496,1
7.17500000000003,68,54.5123735090344,35.3558562783496,1
7.20000000000003,69,55.8717750927842,35.3558562783496,1
7.22500000000004,66,57.2211432315071,35.3558562783496,1
7.25000000000004,70,58.5754369322789,35.3558562783496,1
7.27500000000004,78,59.9878523402172,35.3558562783496,1
7.30000000000004,70,61.4002677481555,35.3558562783496,1
7.32500000000004,65,62.7494877125256,35.3558562783496,1
7.35000000000004,67,64.0838227264947,35.3558562783496,1
7.37500000000004,67,65.4282669192747,35.3558562783496,1
7.40000000000004,70,66.7875960624559,35.3558562783496,1
7.42500000000004,60,68.1108406238615,35.3558562783496,1
7.45000000000004,57,69.3670082914727,35.3558562783496,1
7.47500000000004,52,70.5792502414643,35.3558562783496,1
7.50000000000004,51,71.7579518481533,35.3558562783496,1
7.52500000000004,47,72.9074622919053,35.3558562783496,1
7.55000000000004,61,74.1118996805971,35.3558562783496,1
7.57500000000004,66,75.4205030892117,35.3558562783496,1
7.60000000000004,61,76.7291064978262,35.3558562783496,1
7.62500000000004,55,77.9795785532128,35.3558562783496,1
7.65000000000004,54,79.1921268893437,35.3558562783496,1
7.67500000000004,51,80.382109734416,35.3558562783496,1
7.70000000000004,56,81.5831667698877,35.3558562783496,1
7.72500000000004,56,82.812301221443,35.3558562783496,1

7.75000000000004,40,83.9462725529033,35.3558562783496,1
7.77500000000004,37,84.9652235313867,35.3558562783496,1
7.80000000000004,43,86.0033000429957,35.3558562783496,1
7.82500000000004,37,87.0413765546048,35.3558562783496,1
7.85000000000004,33,88.0126956347524,35.3558562783496,1
7.87500000000004,34,88.9633347664635,35.3558562783496,1
7.90000000000004,48,90.0111803811141,35.3558562783496,1
7.92500000000005,41,91.1060156493996,35.3558562783496,1
7.95000000000005,32,92.0964413826384,35.3558562783496,1
7.97500000000005,32,93.0255796931175,35.3558562783496,1
8.00000000000005,40,94.0095529540397,35.3558562783496,1
8.02500000000005,42,95.0611878895791,35.3558562783496,1
8.05000000000005,38,96.0996712194297,35.3558562783496,1
8.07500000000005,41,97.1317802974227,35.3558562783496,1
8.10000000000005,48,98.2266155657082,35.3558562783496,1
8.12500000000005,52,99.3878060529895,35.3558562783496,1
8.15000000000005,53,100.577896874642,35.3558562783496,1
8.17500000000005,56,101.790343125076,35.3558562783496,1
8.20000000000005,50,102.985621794904,35.3558562783496,1
8.22500000000005,49,104.141208238953,35.3558562783496,1
8.25000000000005,46,105.273082088817,35.3558562783496,1
8.27500000000005,48,106.399059628968,35.3558562783496,1
8.30000000000005,45,107.518949567211,35.3558562783496,1
8.32500000000005,48,108.638839505453,35.3558562783496,1
8.35000000000005,42,109.740049025597,35.3558562783496,1
8.37500000000005,34,110.751146779817,35.3558562783496,1
8.40000000000005,42,111.762244534038,35.3558562783496,1
8.42500000000005,39,112.807345824514,35.3558562783496,1
8.45000000000005,33,113.791988492479,35.3558562783496,1
8.47500000000005,42,114.795991529683,35.3558562783496,1
8.50000000000005,44,115.872977981355,35.3558562783496,1
8.52500000000005,41,116.94359018117,35.3558562783496,1
8.55000000000005,33,117.941218966516,35.3558562783496,1
8.57500000000005,26,118.831748151417,35.3558562783496,1
8.60000000000005,26,119.669262106525,35.3558562783496,1
8.62500000000006,17,120.426629133582,35.3558562783496,1
8.65000000000006,15,121.083307940394,35.3558562783496,1
8.67500000000006,10,121.661078750542,35.3558562783496,1
8.70000000000006,7,122.138063129805,35.3558562783496,1
8.72500000000006,6,122.556509801352,35.3558562783496,1
8.75000000000006,7,122.974956472899,35.3558562783496,1
8.77500000000006,9,123.43861379932,35.3558562783496,1
8.80000000000006,10,123.944690852162,35.3558562783496,1
8.82500000000006,12,124.488882250146,35.3558562783496,1
8.85000000000006,12,125.057860940433,35.3558562783496,1
8.87500000000006,13,125.638456184073,35.3558562783496,1
8.90000000000006,13,126.230667981068,35.3558562783496,1
8.92500000000006,15,126.844842636873,35.3558562783496,1
8.95000000000006,15,127.480980151488,35.3558562783496,1
8.97500000000006,12,128.083538253938,35.3558562783496,1
9.00000000000006,12,128.652516944224,35.3558562783496,1
9.02500000000006,17,129.275616338871,35.3558562783496,1

9.05000000000006,16,129.942726388375,35.3558562783496,1
9.07500000000006,13,130.567332286873,35.3558562783496,1
9.10000000000006,12,131.147927530513,35.3558562783496,1
9.12500000000006,11,131.704794686565,35.3558562783496,1
9.15000000000006,10,132.236874550314,35.3558562783496,1
9.17500000000006,8,132.728861180775,35.3558562783496,1
9.20000000000006,9,133.207520758395,35.3558562783496,1
9.22500000000006,7,133.671178084816,35.3558562783496,1
9.25000000000006,7,134.105742737658,35.3558562783496,1
9.27500000000006,8,134.555309641699,35.3558562783496,1
9.30000000000006,9,135.033969219319,35.3558562783496,1
9.32500000000007,11,135.552722030227,35.3558562783496,1
9.35000000000007,12,136.109589186278,35.3558562783496,1
9.37500000000007,16,136.722578531421,35.3558562783496,1
9.40000000000007,18,137.399505397851,35.3558562783496,1
9.42500000000007,18,138.09635913071,35.3558562783496,1
9.45000000000007,20,138.812060162444,35.3558562783496,1
9.47500000000007,20,139.546608493053,35.3558562783496,1
9.50000000000007,23,140.307740322209,35.3558562783496,1
9.52500000000007,20,141.068872151366,35.3558562783496,1
9.55000000000007,19,141.794120892408,35.3558562783496,1
9.57500000000007,23,142.545953131999,35.3558562783496,1
9.60000000000007,23,143.333668459703,35.3558562783496,1
9.62500000000007,21,144.103870152503,35.3558562783496,1
9.65000000000007,15,144.798282938759,35.3558562783496,1
9.67500000000007,12,145.400841041209,35.3558562783496,1
9.70000000000007,11,145.95770819726,35.3558562783496,1
9.72500000000007,11,146.502463819076,35.3558562783496,1
9.75000000000007,11,147.047219440892,35.3558562783496,1
9.77500000000007,12,147.604086596943,35.3558562783496,1
9.80000000000007,11,148.160953752994,35.3558562783496,1
9.82500000000007,8,148.665616141522,35.3558562783496,1
9.85000000000007,7,149.115183045563,35.3558562783496,1
9.87500000000007,8,149.564749949604,35.3558562783496,1
9.90000000000007,10,150.056736580065,35.3558562783496,1
9.92500000000007,10,150.576140685748,35.3558562783496,1
9.95000000000007,7,151.05312506501,35.3558562783496,1
9.97500000000007,3,151.412652064003,0,0
10.0000000000001,1,151.637021736574,0,0
10.0250000000001,1,151.801271736574,0,0
10.0500000000001,0,151.883396736574,0,0
10.0750000000001,0,151.883396736574,0,0
10.1000000000001,1,151.965521736574,0,0
10.1250000000001,1,152.129771736574,0,0
10.1500000000001,1,152.294021736574,0,0
10.1750000000001,1,152.458271736574,0,0
10.2000000000001,0,152.540396736574,0,0
10.2250000000001,0,152.540396736574,0,0
10.2500000000001,0,152.540396736574,0,0
10.2750000000001,0,152.540396736574,0,0
10.3000000000001,0,152.540396736574,0,0
10.3250000000001,0,152.540396736574,0,0

10.3500000000001,1,152.622521736574,0,0
10.3750000000001,1,152.786771736574,0,0
10.4000000000001,0,152.868896736574,0,0
10.4250000000001,0,152.868896736574,0,0
10.4500000000001,0,152.868896736574,0,0
10.4750000000001,0,152.868896736574,0,0
10.5000000000001,0,152.868896736574,0,0
10.5250000000001,0,152.868896736574,0,0
10.5500000000001,0,152.868896736574,0,0
10.5750000000001,0,152.868896736574,0,0
10.6000000000001,0,152.868896736574,0,0
10.6250000000001,0,152.868896736574,0,0
10.6500000000001,0,152.868896736574,0,0
10.6750000000001,0,152.868896736574,0,0
10.7000000000001,0,152.868896736574,0,0
10.7250000000001,0,152.868896736574,0,0
10.7500000000001,0,152.868896736574,0,0
10.7750000000001,0,152.868896736574,0,0
10.8000000000001,0,152.868896736574,0,0
10.8250000000001,0,152.868896736574,0,0
10.8500000000001,0,152.868896736574,0,0
10.8750000000001,0,152.868896736574,0,0
10.9000000000001,0,152.868896736574,0,0
10.9250000000001,0,152.868896736574,0,0
10.9500000000001,0,152.868896736574,0,0
10.9750000000001,0,152.868896736574,0,0
11.0000000000001,0,152.868896736574,0,0
11.0250000000001,0,152.868896736574,0,0
11.0500000000001,0,152.868896736574,0,0
11.0750000000001,0,152.868896736574,0,0
11.1000000000001,0,152.868896736574,0,0
11.1250000000001,1,152.951021736574,0,0
11.1500000000001,0,153.033146736574,0,0
11.1750000000001,1,153.115271736574,0,0
11.2000000000001,0,153.197396736574,0,0
11.2250000000001,0,153.197396736574,0,0
11.2500000000001,0,153.197396736574,0,0
11.2750000000001,0,153.197396736574,0,0
11.3000000000001,0,153.197396736574,0,0
11.3250000000001,0,153.197396736574,0,0
11.3500000000001,0,153.197396736574,0,0
11.3750000000001,0,153.197396736574,0,0
11.4000000000001,0,153.197396736574,0,0
11.4250000000001,0,153.197396736574,0,0
11.4500000000001,0,153.197396736574,0,0
11.4750000000001,0,153.197396736574,0,0
11.5000000000001,0,153.197396736574,0,0
11.5250000000001,0,153.197396736574,0,0
11.5500000000001,0,153.197396736574,0,0
11.5750000000001,0,153.197396736574,0,0
11.6000000000001,0,153.197396736574,0,0
11.6250000000001,0,153.197396736574,0,0

11.6500000000001,0,153.197396736574,0,0
11.6750000000001,0,153.197396736574,0,0
11.7000000000001,0,153.197396736574,0,0
11.7250000000001,1,153.279521736574,0,0
11.7500000000001,0,153.361646736574,0,0
11.7750000000001,1,153.443771736574,0,0
11.8000000000001,0,153.525896736574,0,0
11.8250000000001,0,153.525896736574,0,0
11.8500000000001,0,153.525896736574,0,0
11.8750000000001,0,153.525896736574,0,0
11.9000000000001,0,153.525896736574,0,0
11.9250000000001,0,153.525896736574,0,0
11.9500000000001,0,153.525896736574,0,0
11.9750000000001,0,153.525896736574,0,0
12.0000000000001,0,153.525896736574,0,0
12.0250000000001,0,153.525896736574,0,0
12.0500000000001,0,153.525896736574,0,0
12.0750000000001,0,153.525896736574,0,0
12.1000000000001,0,153.525896736574,0,0
12.1250000000001,1,153.608021736574,0,0
12.1500000000001,0,153.690146736574,0,0
12.1750000000001,0,153.690146736574,0,0
12.2000000000001,4,153.854396736574,0,0
12.2250000000001,0,154.018646736574,0,0
12.2500000000001,1,154.100771736574,0,0
12.2750000000001,0,154.182896736574,0,0
12.3000000000001,1,154.265021736574,0,0
12.3250000000001,1,154.429271736574,0,0
12.3500000000001,0,154.511396736574,0,0
12.3750000000001,0,154.511396736574,0,0
12.4000000000001,0,154.511396736574,0,0
12.4250000000001,0,154.511396736574,0,0
12.4500000000001,0,154.511396736574,0,0
12.4750000000001,1,154.593521736574,0,0
12.5000000000001,0,154.675646736574,0,0
12.5250000000001,0,154.675646736574,0,0
12.5500000000001,1,154.757771736574,0,0
12.5750000000001,1,154.922021736574,0,0
12.6000000000001,1,155.086271736574,0,0
12.6250000000001,1,155.250521736574,0,0
12.6500000000001,0,155.332646736574,0,0
12.6750000000001,1,155.414771736574,0,0
12.7000000000001,0,155.496896736574,0,0
12.7250000000001,0,155.496896736574,0,0
12.7500000000001,0,155.496896736574,0,0
12.7750000000001,1,155.579021736574,0,0
12.8000000000001,1,155.743271736574,0,0
12.8250000000001,0,155.825396736574,0,0
12.8500000000001,1,155.907521736574,0,0
12.8750000000001,1,156.071771736574,0,0
12.9000000000001,0,156.153896736574,0,0
12.9250000000001,1,156.236021736574,0,0

12.9500000000001,0,156.318146736574,0,0
12.9750000000001,0,156.318146736574,0,0
13.0000000000001,0,156.318146736574,0,0
13.0250000000001,0,156.318146736574,0,0
13.0500000000001,0,156.318146736574,0,0
13.0750000000001,0,156.318146736574,0,0
13.1000000000001,1,156.400271736574,0,0
13.1250000000001,0,156.482396736574,0,0
13.1500000000001,0,156.482396736574,0,0
13.1750000000001,0,156.482396736574,0,0
13.2000000000001,2,156.598539025384,0,0
13.2250000000001,5,156.898318396846,0,0
13.2500000000001,7,157.29923780592,0,0
13.2750000000001,11,157.788897943249,36.9588206766533,1
13.3000000000001,17,158.39988580366,36.9588206766533,1
13.3250000000001,25,159.149120853164,36.9588206766533,1
13.3500000000001,31,160.016998501462,36.9588206766533,1
13.3750000000001,32,160.938820304999,36.9588206766533,1
13.4000000000001,35,161.889247512425,36.9588206766533,1
13.4250000000001,34,162.853972488977,36.9588206766533,1
13.4500000000001,35,163.818697465528,36.9588206766533,1
13.4750000000001,36,164.797305517715,36.9588206766533,1
13.5000000000001,32,165.754624672954,36.9588206766533,1
13.5250000000001,36,166.711943828194,36.9588206766533,1
13.5500000000001,41,167.730550406193,36.9588206766533,1
13.5750000000001,38,168.762659484186,36.9588206766533,1
13.6000000000001,34,169.747778908544,36.9588206766533,1
13.6250000000001,32,170.691214988148,36.9588206766533,1
13.6500000000001,31,171.613036791685,36.9588206766533,1
13.6750000000001,35,172.556147492169,36.9588206766533,1
13.7000000000001,33,173.513777751703,36.9588206766533,1
13.7250000000001,32,174.45011911429,36.9588206766533,1
13.7500000000001,30,175.364505419881,36.9588206766533,1
13.7750000000001,32,176.278891725471,36.9588206766533,1
13.8000000000001,34,177.222327805075,36.9588206766533,1
13.8250000000001,34,178.180061653803,36.9588206766533,1
13.8500000000001,32,179.123497733407,36.9588206766533,1
13.8750000000001,34,180.066933813011,36.9588206766533,1
13.9000000000001,32,181.010369892615,36.9588206766533,1
13.9250000000001,32,181.939508203094,36.9588206766533,1
13.9500000000001,31,182.861330006631,36.9588206766533,1
13.9750000000001,32,183.783151810168,36.9588206766533,1
14.0000000000001,29,184.689977625193,36.9588206766533,1
14.0250000000001,24,185.534562975231,36.9588206766533,1
14.0500000000001,24,186.339220355735,36.9588206766533,1
14.0750000000001,24,187.14387773624,36.9588206766533,1
14.1000000000001,30,187.996023576843,36.9588206766533,1
14.1250000000001,27,188.872574744909,36.9588206766533,1
14.1500000000001,30,189.749125912975,36.9588206766533,1
14.1750000000001,28,190.633507716168,36.9588206766533,1
14.2000000000001,33,191.539844576357,36.9588206766533,1
14.2250000000001,46,192.568615633569,36.9588206766533,1

14.2500000000001,47,193.688635117491,36.9588206766533,1
14.2750000000001,52,194.843867548544,36.9588206766533,1
14.3000000000001,48,196.005058035825,36.9588206766533,1
14.3250000000001,43,197.11256636492,36.9588206766533,1
14.3500000000001,41,198.176952581727,36.9588206766533,1
14.3750000000001,31,199.160061808024,36.9588206766533,1
14.4000000000001,38,200.123566956315,36.9588206766533,1
14.4250000000001,34,201.108686380673,36.9588206766533,1
14.4500000000001,36,202.080303305037,36.9588206766533,1
14.4750000000001,28,203.007617957879,36.9588206766533,1
14.5000000000001,30,203.891999761073,36.9588206766533,1
14.5250000000001,28,204.776381564266,36.9588206766533,1
14.5500000000001,32,205.675515372348,36.9588206766533,1
14.5750000000001,32,206.604653682827,36.9588206766533,1
14.6000000000001,30,207.519039988418,36.9588206766533,1
14.6250000000001,40,208.488261244452,36.9588206766533,1
14.6500000000001,48,209.576644040421,36.9588206766533,1
14.6750000000001,44,210.690378352523,36.9588206766533,1
14.7000000000001,37,211.73468084714,36.9588206766533,1
14.7250000000001,32,212.69879687518,36.9588206766533,1
14.7500000000001,33,213.635138237767,36.9588206766533,1
14.7750000000001,36,214.599660445113,36.9588206766533,1
14.8000000000001,33,215.56418265246,36.9588206766533,1
14.8250000000001,31,216.493207508105,36.9588206766533,1
14.8500000000001,32,217.415029311642,36.9588206766533,1
14.8750000000001,29,218.321855126667,36.9588206766533,1
14.9000000000001,31,219.221364434751,36.9588206766533,1
14.9250000000001,36,220.171367083048,36.9588206766533,1
14.9500000000001,32,221.128686238287,36.9588206766533,1
14.9750000000001,35,222.079113445714,36.9588206766533,1
15.0000000000001,34,223.043838422265,36.9588206766533,1
15.0250000000001,33,223.994477553976,36.9588206766533,1
15.0500000000001,28,224.900814414166,36.9588206766533,1
15.0750000000001,32,225.799948222248,36.9588206766533,1
15.1000000000001,32,226.729086532727,36.9588206766533,1
15.1250000000001,29,227.635912347752,36.9588206766533,1
15.1500000000001,33,228.549941214885,36.9588206766533,1
15.1750000000001,23,229.415571086084,36.9588206766533,1
15.2000000000001,25,230.220053749936,36.9588206766533,1
15.2250000000001,25,231.041303749936,36.9588206766533,1
15.2500000000001,18,231.800355616366,36.9588206766533,1
15.2750000000002,14,232.456066095684,36.9588206766533,1
15.3000000000002,11,233.035727519481,36.9588206766533,1
15.3250000000002,9,233.554480330389,36.9588206766533,1
15.3500000000002,9,234.047230330389,0,0
15.3750000000002,6,234.494769675515,0,0
15.4000000000002,7,234.913216347062,0,0
15.4250000000002,6,235.33166301861,0,0
15.4500000000002,7,235.750109690157,0,0
15.4750000000002,5,236.15102909923,0,0
15.5000000000002,6,236.535830527008,0,0
15.5250000000002,7,236.954277198556,0,0

15.5500000000002,7,237.388841851398,0,0
15.5750000000002,10,237.865826230661,0,1
15.6000000000002,10,238.385230336343,0,1
15.6250000000002,10,238.904634442026,0,1
15.6500000000002,11,239.436714305775,0,1
15.6750000000002,10,239.968794169524,0,1
15.7000000000002,9,240.474871222366,0,0
15.7250000000002,8,240.953530799986,0,0
15.7500000000002,8,241.418099955225,0,0
15.7750000000002,7,241.867666859266,0,0
15.8000000000002,4,242.249199185687,0,0
15.8250000000002,0,242.413449185687,0,0
15.8500000000002,2,242.529591474497,0,0
15.8750000000002,0,242.645733763307,0,0
15.9000000000002,1,242.727858763307,0,0
15.9250000000002,1,242.892108763307,0,0
15.9500000000002,1,243.056358763307,0,0
15.9750000000002,1,243.220608763307,0,0
16.0000000000002,1,243.384858763307,0,0
16.0250000000002,0,243.466983763307,0,0
16.0500000000002,0,243.466983763307,0,0
16.0750000000002,0,243.466983763307,0,0
16.1000000000002,1,243.549108763307,0,0
16.1250000000002,1,243.713358763307,0,0
16.1500000000002,0,243.795483763307,0,0
16.1750000000002,0,243.795483763307,0,0
16.2000000000001,0,243.795483763307,0,0
16.2250000000001,0,243.795483763307,0,0
16.2500000000001,0,243.795483763307,0,0
16.2750000000001,0,243.795483763307,0,0
16.3000000000001,0,243.795483763307,0,0
16.3250000000001,0,243.795483763307,0,0
16.3500000000001,0,243.795483763307,0,0
16.3750000000001,0,243.795483763307,0,0
16.4000000000001,0,243.795483763307,0,0
16.4250000000001,0,243.795483763307,0,0
16.4500000000001,0,243.795483763307,0,0
16.4750000000001,0,243.795483763307,0,0
16.5000000000001,0,243.795483763307,0,0
16.5250000000001,0,243.795483763307,0,0
16.5500000000001,0,243.795483763307,0,0
16.5750000000001,1,243.877608763307,0,0
16.6000000000001,0,243.959733763307,0,0
16.6250000000001,1,244.041858763307,0,0
16.6500000000001,0,244.123983763307,0,0
16.6750000000001,1,244.206108763307,0,0
16.7000000000001,2,244.404376052117,0,0
16.7250000000001,1,244.602643340927,0,0
16.7500000000001,0,244.684768340927,0,0
16.7750000000001,0,244.684768340927,0,0
16.8000000000001,1,244.766893340927,0,0
16.8250000000001,1,244.931143340927,0,0

16.8500000000001,1,245.095393340927,0,0
16.8750000000001,1,245.259643340927,0,0
16.9000000000001,0,245.341768340927,0,0
16.9250000000001,0,245.341768340927,0,0
16.9500000000001,0,245.341768340927,0,0
16.9750000000001,0,245.341768340927,0,0
17.0000000000001,0,245.341768340927,0,0
17.0250000000001,0,245.341768340927,0,0
17.0500000000001,0,245.341768340927,0,0
17.0750000000001,0,245.341768340927,0,0
17.1000000000001,1,245.423893340927,0,0
17.1250000000001,1,245.588143340927,0,0
17.1500000000001,4,245.834518340927,0,0
17.1750000000001,0,245.998768340927,0,0
17.2000000000001,1,246.080893340927,0,0
17.2250000000001,1,246.245143340927,0,0
17.2500000000001,1,246.409393340927,0,0
17.2750000000001,0,246.491518340927,0,0
17.3000000000001,0,246.491518340927,0,0
17.3250000000001,0,246.491518340927,0,0
17.3500000000001,1,246.573643340927,0,0
17.3750000000001,0,246.655768340927,0,0
17.4000000000001,0,246.655768340927,0,0
17.4250000000001,0,246.655768340927,0,0
17.4500000000001,0,246.655768340927,0,0
17.4750000000001,0,246.655768340927,0,0
17.5000000000001,0,246.655768340927,0,0
17.5250000000001,0,246.655768340927,0,0
17.5500000000001,0,246.655768340927,0,0
17.5750000000001,0,246.655768340927,0,0
17.6000000000001,0,246.655768340927,0,0
17.6250000000001,0,246.655768340927,0,0
17.6500000000001,0,246.655768340927,0,0
17.6750000000001,0,246.655768340927,0,0
17.7000000000001,0,246.655768340927,0,0
17.7250000000001,0,246.655768340927,0,0
17.7500000000001,1,246.737893340927,0,0
17.7750000000001,1,246.902143340927,0,0
17.8000000000001,0,246.984268340927,0,0
17.8250000000001,0,246.984268340927,0,0
17.8500000000001,0,246.984268340927,0,0
17.8750000000001,0,246.984268340927,0,0
17.9000000000001,0,246.984268340927,0,0
17.9250000000001,0,246.984268340927,0,0
17.95,0,246.984268340927,0,0
17.975,0,246.984268340927,0,0
18,0,246.984268340927,0,0
18.025,0,246.984268340927,0,0
18.05,0,246.984268340927,0,0
18.075,0,246.984268340927,0,0
18.1,0,246.984268340927,0,0
18.125,0,246.984268340927,0,0

18.15,0,246.984268340927,0,0
18.175,1,247.066393340927,0,0
18.2,0,247.148518340927,0,0
18.225,1,247.230643340927,0,0
18.25,1,247.394893340927,0,0
18.275,1,247.559143340927,0,0
18.3,0,247.641268340927,0,0
18.325,0,247.641268340927,0,0
18.35,0,247.641268340927,0,0
18.375,0,247.641268340927,0,0
18.4,0,247.641268340927,0,0
18.425,0,247.641268340927,0,0
18.45,0,247.641268340927,0,0
18.475,0,247.641268340927,0,0
18.5,1,247.723393340927,0,0
18.525,0,247.805518340927,0,0
18.55,0,247.805518340927,0,0
18.575,0,247.805518340927,0,0
18.6,0,247.805518340927,0,0
18.625,0,247.805518340927,0,0
18.65,0,247.805518340927,0,0
18.675,0,247.805518340927,0,0
18.7,0,247.805518340927,0,0
18.725,0,247.805518340927,0,0
18.75,1,247.887643340927,0,0
18.775,0,247.969768340927,0,0
18.8,0,247.969768340927,0,0
18.825,0,247.969768340927,0,0
18.85,0,247.969768340927,0,0
18.875,1,248.051893340927,0,0
18.9,0,248.134018340927,0,0
18.925,0,248.134018340927,0,0
18.95,5,248.317655423579,0,0
18.975,0,248.501292506231,0,0
19,0,248.501292506231,0,0
19.025,0,248.501292506231,0,0
19.05,0,248.501292506231,0,0
19.075,0,248.501292506231,0,0
19.1,0,248.501292506231,0,0
19.125,0,248.501292506231,0,0
19.15,0,248.501292506231,0,0
19.175,0,248.501292506231,0,0
19.2,0,248.501292506231,0,0
19.225,1,248.583417506231,0,0
19.25,0,248.665542506231,0,0
19.275,1,248.747667506231,0,0
19.3,1,248.911917506231,0,0
19.325,0,248.994042506231,0,0
19.35,0,248.994042506231,0,0
19.375,0,248.994042506231,0,0
19.4,0,248.994042506231,0,0
19.425,0,248.994042506231,0,0

19.45,0,248.994042506231,0,0
19.475,0,248.994042506231,0,0
19.5,0,248.994042506231,0,0
19.525,0,248.994042506231,0,0
19.55,0,248.994042506231,0,0
19.575,0,248.994042506231,0,0
19.6,0,248.994042506231,0,0
19.625,0,248.994042506231,0,0
19.65,0,248.994042506231,0,0
19.675,1,249.076167506231,0,0
19.7,0,249.158292506231,0,0
19.7249999999999,0,249.158292506231,0,0
19.7499999999999,0,249.158292506231,0,0
19.7749999999999,1,249.240417506231,0,0
19.7999999999999,0,249.322542506231,0,0
19.8249999999999,0,249.322542506231,0,0
19.8499999999999,0,249.322542506231,0,0
19.8749999999999,0,249.322542506231,0,0
19.8999999999999,0,249.322542506231,0,0
19.9249999999999,0,249.322542506231,0,0
19.9499999999999,0,249.322542506231,0,0
19.9749999999999,0,249.322542506231,0,0
19.9999999999999,0,249.322542506231,0,0
20.0249999999999,0,249.322542506231,0,0
20.0499999999999,0,249.322542506231,0,0
20.0749999999999,0,249.322542506231,0,0
20.0999999999999,0,249.322542506231,0,0
20.1249999999999,0,249.322542506231,0,0
20.1499999999999,0,249.322542506231,0,0
20.1749999999999,0,249.322542506231,0,0
20.1999999999999,0,249.322542506231,0,0
20.2249999999999,0,249.322542506231,0,0
20.2499999999999,0,249.322542506231,0,0
20.2749999999999,1,249.404667506231,0,0
20.2999999999999,2,249.602934795041,0,0
20.3249999999999,1,249.801202083851,0,0
20.3499999999999,0,249.883327083851,0,0
20.3749999999999,0,249.883327083851,0,0
20.3999999999999,0,249.883327083851,0,0
20.4249999999999,0,249.883327083851,0,0
20.4499999999999,0,249.883327083851,0,0
20.4749999999999,0,249.883327083851,0,0
20.4999999999999,0,249.883327083851,0,0
20.5249999999999,0,249.883327083851,0,0
20.5499999999999,0,249.883327083851,0,0
20.5749999999999,0,249.883327083851,0,0
20.5999999999999,0,249.883327083851,0,0
20.6249999999999,0,249.883327083851,0,0
20.6499999999999,0,249.883327083851,0,0
20.6749999999999,0,249.883327083851,0,0
20.6999999999999,0,249.883327083851,0,0
20.7249999999999,0,249.883327083851,0,0

20.7499999999999,2,249.999469372661,0,0
20.7749999999999,0,250.115611661471,0,0
20.7999999999999,0,250.115611661471,0,0
20.8249999999999,0,250.115611661471,0,0
20.8499999999999,0,250.115611661471,0,0
20.8749999999999,0,250.115611661471,0,0
20.8999999999999,0,250.115611661471,0,0
20.9249999999999,0,250.115611661471,0,0
20.9499999999999,0,250.115611661471,0,0
20.9749999999999,0,250.115611661471,0,0
20.9999999999999,0,250.115611661471,0,0
21.0249999999999,1,250.197736661471,0,0
21.0499999999999,0,250.279861661471,0,0
21.0749999999999,1,250.361986661471,0,0
21.0999999999999,0,250.444111661471,0,0
21.1249999999999,1,250.526236661471,0,0
21.1499999999999,0,250.608361661471,0,0
21.1749999999999,1,250.69048666147,0,0
21.1999999999999,2,250.88875395028,0,0
21.2249999999999,1,251.08702123909,0,0
21.2499999999999,1,251.25127123909,0,0
21.2749999999999,1,251.41552123909,0,0
21.2999999999999,1,251.57977123909,0,0
21.3249999999999,1,251.74402123909,0,0
21.3499999999999,1,251.90827123909,0,0
21.3749999999999,1,252.07252123909,0,0
21.3999999999999,1,252.23677123909,0,0
21.4249999999999,1,252.40102123909,0,0
21.4499999999999,0,252.48314623909,0,0
21.4749999999998,0,252.48314623909,0,0
21.4999999999998,0,252.48314623909,0,0
21.5249999999998,0,252.48314623909,0,0
21.5499999999998,0,252.48314623909,0,0
21.5749999999998,0,252.48314623909,0,0
21.5999999999998,0,252.48314623909,0,0
21.6249999999998,0,252.48314623909,0,0
21.6499999999998,1,252.56527123909,0,0
21.6749999999998,0,252.64739623909,0,0
21.6999999999998,0,252.64739623909,0,0
21.7249999999998,0,252.64739623909,0,0
21.7499999999998,0,252.64739623909,0,0
21.7749999999998,0,252.64739623909,0,0
21.7999999999998,0,252.64739623909,0,0
21.8249999999998,0,252.64739623909,0,0
21.8499999999998,1,252.72952123909,0,0
21.8749999999998,0,252.81164623909,0,0
21.8999999999998,1,252.89377123909,0,0
21.9249999999998,1,253.05802123909,0,0
21.9499999999998,1,253.22227123909,0,0
21.9749999999998,1,253.38652123909,0,0
21.9999999999998,0,253.46864623909,0,0
22.0249999999998,0,253.46864623909,0,0

22.0499999999998,1,253.55077123909,0,0
22.0749999999998,0,253.63289623909,0,0
22.0999999999998,0,253.63289623909,0,0
22.1249999999998,0,253.63289623909,0,0
22.1499999999998,0,253.63289623909,0,0
22.1749999999998,0,253.63289623909,0,0
22.1999999999998,0,253.63289623909,0,0
22.2249999999998,0,253.63289623909,0,0
22.2499999999998,1,253.71502123909,0,0
22.2749999999998,0,253.79714623909,0,0
22.2999999999998,1,253.87927123909,0,0
22.3249999999998,0,253.96139623909,0,0
22.3499999999998,0,253.96139623909,0,0
22.3749999999998,1,254.04352123909,0,0
22.3999999999998,0,254.12564623909,0,0
22.4249999999998,0,254.12564623909,0,0
22.4499999999998,0,254.12564623909,0,0
22.4749999999998,0,254.12564623909,0,0
22.4999999999998,0,254.12564623909,0,0
22.5249999999998,1,254.20777123909,0,0
22.5499999999998,4,254.45414623909,0,0
22.5749999999998,8,254.85068081671,0,0
22.5999999999998,25,255.49359039433,41.4380011367546,1
22.6249999999998,32,256.368784549569,41.4380011367546,1
22.6499999999998,36,257.326103704809,41.4380011367546,1
22.6749999999998,45,258.369764952765,41.4380011367546,1
22.6999999999998,52,259.512887997717,41.4380011367546,1
22.7249999999998,53,260.702978819369,41.4380011367546,1
22.7499999999998,50,261.881569288076,41.4380011367546,1
22.7749999999998,48,263.031259422411,41.4380011367546,1
22.7999999999998,62,264.246891009351,41.4380011367546,1
22.8249999999998,71,265.585541456126,41.4380011367546,1
22.8499999999998,81,267.016664006249,41.4380011367546,1
22.8749999999998,86,268.517384925191,41.4380011367546,1
22.8999999999998,87,270.044991848869,41.4380011367546,1
22.9249999999998,88,271.581403642155,41.4380011367546,1
22.9499999999998,83,273.099998788393,41.4380011367546,1
22.9749999999998,73,274.549869453664,41.4380011367546,1
22.9999999999998,67,275.923767857638,41.4380011367546,1
23.0249999999998,70,277.28309700082,41.4380011367546,1
23.0499999999998,64,278.627204047611,41.4380011367546,1
23.0749999999998,69,279.966385532353,41.4380011367546,1
23.0999999999998,69,281.330748501837,41.4380011367546,1
23.1249999999998,76,282.728879138056,41.4380011367546,1
23.1499999999998,80,284.179376620141,41.4380011367546,1
23.1749999999998,78,285.639233311897,41.4380011367546,1
23.1999999999998,75,287.075765035902,41.4380011367546,1
23.2249999999997,74,288.493454361316,41.4380011367546,1
23.2499999999997,87,289.965931328607,41.4380011367546,1
23.2749999999997,94,291.528173749922,41.4380011367546,1
23.2999999999997,79,293.054348133025,41.4380011367546,1
23.3249999999997,76,294.500240251024,41.4380011367546,1

23.3499999999997,72,295.913043135359,41.4380011367546,1
23.3749999999997,66,297.2770835222,41.4380011367546,1
23.3999999999997,70,298.631377222971,41.4380011367546,1
23.4249999999997,75,300.02970763262,41.4380011367546,1
23.4499999999997,65,301.403043913057,41.4380011367546,1
23.4749999999997,62,302.71180972729,41.4380011367546,1
23.4999999999997,56,303.97302984972,41.4380011367546,1
23.5249999999997,49,305.162472075498,41.4380011367546,1
23.5499999999997,48,306.306325765784,41.4380011367546,1
23.5749999999997,43,307.413834094879,41.4380011367546,1
23.5999999999997,40,308.47176783937,41.4380011367546,1
23.6249999999997,42,309.52340277491,41.4380011367546,1
23.6499999999997,40,310.575037710449,41.4380011367546,1
23.6749999999997,39,311.60731227675,41.4380011367546,1
23.6999999999997,44,312.664938359184,41.4380011367546,1
23.7249999999997,42,313.741924810857,41.4380011367546,1
23.7499999999997,41,314.800012218713,41.4380011367546,1
23.7749999999997,44,315.870624418528,41.4380011367546,1
23.7999999999997,43,316.953909679152,41.4380011367546,1
23.8249999999997,44,318.037194939776,41.4380011367546,1
23.8499999999997,47,319.14497119565,41.4380011367546,1
23.8749999999997,39,320.220862290327,41.4380011367546,1
23.8999999999997,40,321.253136856628,41.4380011367546,1
23.9249999999997,39,322.285411422929,41.4380011367546,1
23.9499999999997,43,323.336811522356,41.4380011367546,1
23.9749999999997,42,324.407571991021,41.4380011367546,1
23.9999999999997,38,325.446055320871,41.4380011367546,1
24.0249999999997,42,326.484538650722,41.4380011367546,1
24.0499999999997,45,327.567680728535,41.4380011367546,1
24.0749999999997,42,328.650822806348,41.4380011367546,1
24.0999999999997,38,329.689306136199,41.4380011367546,1
24.1249999999997,34,330.674425560557,41.4380011367546,1
24.1499999999997,33,331.625064692268,41.4380011367546,1
24.1749999999997,31,332.554089547912,41.4380011367546,1
24.1999999999997,30,333.461159346561,41.4380011367546,1
24.2249999999997,28,334.345541149754,41.4380011367546,1
24.2499999999997,27,335.206839820312,41.4380011367546,1
24.2749999999997,29,336.075830497812,41.4380011367546,1
24.2999999999997,29,336.960343817384,41.4380011367546,1
24.3249999999997,28,337.837165130012,41.4380011367546,1
24.3499999999997,30,338.721546933206,41.4380011367546,1
24.3749999999997,33,339.643136290904,41.4380011367546,1
24.3999999999997,33,340.586680705598,41.4380011367546,1
24.4249999999997,26,341.477209890499,41.4380011367546,1
24.4499999999997,26,342.314723845606,41.4380011367546,1
24.4749999999997,24,343.135809513412,41.4380011367546,1
24.4999999999997,20,343.905412368969,41.4380011367546,1
24.5249999999997,22,344.657886928548,41.4380011367546,1
24.5499999999997,21,345.419431351772,41.4380011367546,1
24.5749999999997,17,346.134385430224,41.4380011367546,1
24.5999999999997,14,346.780279092617,41.4380011367546,1
24.6249999999997,16,347.416062705506,41.4380011367546,1

24.6499999999997,14,348.051846318395,41.4380011367546,1
24.6749999999997,12,348.643619276427,41.4380011367546,1
24.6999999999997,11,349.200486432478,41.4380011367546,1
24.7249999999997,11,349.745242054294,41.4380011367546,1
24.7499999999997,8,350.249904442821,41.4380011367546,1
24.7749999999997,6,350.683353365567,41.4380011367546,1
24.7999999999997,5,351.068154793345,41.4380011367546,1
24.8249999999997,6,351.452956221124,41.4380011367546,1
24.8499999999997,7,351.871402892671,41.4380011367546,1
24.8749999999997,8,352.320969796712,41.4380011367546,1
24.8999999999997,8,352.785538951951,41.4380011367546,1
24.9249999999997,13,353.313929428069,41.4380011367546,1
24.9499999999997,12,353.894524671709,41.4380011367546,1
24.9749999999997,10,354.438716069694,41.4380011367546,1
24.9999999999996,9,354.944793122535,41.4380011367546,1
25.0249999999996,8,355.423452700155,0,0
25.0499999999996,9,355.902112277775,0,0
25.0749999999996,8,356.380771855395,0,0
25.0999999999996,9,356.859431433014,0,0
25.1249999999996,9,357.352181433014,0,0
25.1499999999996,9,357.844931433014,0,0
25.1749999999996,8,358.323591010634,0,0
25.1999999999996,8,358.788160165874,0,0
25.2249999999996,9,359.266819743494,0,0
25.2499999999996,10,359.772896796335,0,1
25.2749999999996,9,360.278973849176,0,0
25.2999999999996,7,360.742631175597,0,0
25.3249999999996,5,361.143550584671,0,0
25.3499999999996,4,361.491437667323,0,0
25.3749999999996,3,361.797932339894,0,0
25.3999999999996,0,361.940177012466,0,0
25.4249999999996,1,362.022302012466,0,0
25.4499999999996,0,362.104427012466,0,0
25.4749999999996,0,362.104427012466,0,0
25.4999999999996,1,362.186552012466,0,0
25.5249999999996,1,362.350802012466,0,0
25.5499999999996,0,362.432927012466,0,0
25.5749999999996,0,362.432927012466,0,0
25.5999999999996,0,362.432927012466,0,0
25.6249999999996,1,362.515052012466,0,0
25.6499999999996,0,362.597177012466,0,0
25.6749999999996,0,362.597177012466,0,0
25.6999999999996,5,362.780814095118,0,0
25.7249999999996,0,362.964451177771,0,0
25.7499999999996,0,362.964451177771,0,0
25.7749999999996,0,362.964451177771,0,0
25.7999999999996,0,362.964451177771,0,0
25.8249999999996,0,362.964451177771,0,0
25.8499999999996,0,362.964451177771,0,0
25.8749999999996,0,362.964451177771,0,0
25.8999999999996,0,362.964451177771,0,0
25.9249999999996,0,362.964451177771,0,0

25.9499999999996,0,362.964451177771,0,0
25.9749999999996,0,362.964451177771,0,0
25.9999999999996,0,362.964451177771,0,0
26.0249999999996,0,362.964451177771,0,0
26.0499999999996,0,362.964451177771,0,0
26.0749999999996,0,362.964451177771,0,0
26.0999999999996,0,362.964451177771,0,0
26.1249999999996,0,362.964451177771,0,0
26.1499999999996,0,362.964451177771,0,0
26.1749999999996,0,362.964451177771,0,0
26.1999999999996,0,362.964451177771,0,0
26.2249999999996,0,362.964451177771,0,0
26.2499999999996,0,362.964451177771,0,0
26.2749999999996,0,362.964451177771,0,0
26.2999999999996,0,362.964451177771,0,0
26.3249999999996,0,362.964451177771,0,0
26.3499999999996,0,362.964451177771,0,0
26.3749999999996,0,362.964451177771,0,0
26.3999999999996,0,362.964451177771,0,0
26.4249999999996,0,362.964451177771,0,0
26.4499999999996,0,362.964451177771,0,0
26.4749999999996,1,363.046576177771,0,0
26.4999999999996,0,363.128701177771,0,0
26.5249999999996,0,363.128701177771,0,0
26.5499999999996,0,363.128701177771,0,0
26.5749999999996,0,363.128701177771,0,0
26.5999999999996,0,363.128701177771,0,0
26.6249999999996,0,363.128701177771,0,0
26.6499999999996,0,363.128701177771,0,0
26.6749999999996,0,363.128701177771,0,0
26.6999999999996,0,363.128701177771,0,0
26.7249999999996,0,363.128701177771,0,0
26.7499999999995,0,363.128701177771,0,0
26.7749999999995,0,363.128701177771,0,0
26.7999999999995,0,363.128701177771,0,0
26.8249999999995,0,363.128701177771,0,0
26.8499999999995,0,363.128701177771,0,0
26.8749999999995,0,363.128701177771,0,0
26.8999999999995,0,363.128701177771,0,0
26.9249999999995,0,363.128701177771,0,0
26.9499999999995,0,363.128701177771,0,0
26.9749999999995,0,363.128701177771,0,0
26.9999999999995,0,363.128701177771,0,0
27.0249999999995,0,363.128701177771,0,0
27.0499999999995,5,363.312338260423,0,0
27.0749999999995,0,363.495975343075,0,0
27.0999999999995,0,363.495975343075,0,0
27.1249999999995,0,363.495975343075,0,0
27.1499999999995,0,363.495975343075,0,0
27.1749999999995,0,363.495975343075,0,0
27.1999999999995,0,363.495975343075,0,0
27.2249999999995,0,363.495975343075,0,0

27.2499999999995,0,363.495975343075,0,0
27.2749999999995,0,363.495975343075,0,0
27.2999999999995,0,363.495975343075,0,0
27.3249999999995,0,363.495975343075,0,0
27.3499999999995,0,363.495975343075,0,0
27.3749999999995,0,363.495975343075,0,0
27.3999999999995,0,363.495975343075,0,0
27.4249999999995,0,363.495975343075,0,0
27.4499999999995,0,363.495975343075,0,0
27.4749999999995,0,363.495975343075,0,0
27.4999999999995,0,363.495975343075,0,0
27.5249999999995,0,363.495975343075,0,0
27.5499999999995,0,363.495975343075,0,0
27.5749999999995,0,363.495975343075,0,0
27.5999999999995,0,363.495975343075,0,0
27.6249999999995,0,363.495975343075,0,0
27.6499999999995,0,363.495975343075,0,0
27.6749999999995,0,363.495975343075,0,0
27.6999999999995,0,363.495975343075,0,0
27.7249999999995,0,363.495975343075,0,0
27.7499999999995,0,363.495975343075,0,0
27.7749999999995,0,363.495975343075,0,0
27.7999999999995,0,363.495975343075,0,0
27.8249999999995,0,363.495975343075,0,0
27.8499999999995,0,363.495975343075,0,0
27.8749999999995,0,363.495975343075,0,0
27.8999999999995,0,363.495975343075,0,0
27.9249999999995,0,363.495975343075,0,0
27.9499999999995,0,363.495975343075,0,0
27.9749999999995,0,363.495975343075,0,0
27.9999999999995,0,363.495975343075,0,0
28.0249999999995,0,363.495975343075,0,0
28.0499999999995,0,363.495975343075,0,0
28.0749999999995,0,363.495975343075,0,0
28.0999999999995,0,363.495975343075,0,0
28.1249999999995,0,363.495975343075,0,0
28.1499999999995,0,363.495975343075,0,0
28.1749999999995,0,363.495975343075,0,0
28.1999999999995,0,363.495975343075,0,0
28.2249999999995,0,363.495975343075,0,0
28.2499999999995,0,363.495975343075,0,0
28.2749999999995,0,363.495975343075,0,0
28.2999999999995,0,363.495975343075,0,0
28.3249999999995,0,363.495975343075,0,0
28.3499999999995,0,363.495975343075,0,0
28.3749999999995,0,363.495975343075,0,0
28.3999999999995,2,363.612117631885,0,0
28.4249999999995,0,363.728259920695,0,0
28.4499999999995,0,363.728259920695,0,0
28.4749999999995,0,363.728259920695,0,0
28.4999999999994,0,363.728259920695,0,0
28.5249999999994,0,363.728259920695,0,0

28.5499999999994,0,363.728259920695,0,0
28.5749999999994,0,363.728259920695,0,0
28.5999999999994,0,363.728259920695,0,0
28.6249999999994,0,363.728259920695,0,0
28.6499999999994,0,363.728259920695,0,0
28.6749999999994,0,363.728259920695,0,0
28.6999999999994,0,363.728259920695,0,0
28.7249999999994,0,363.728259920695,0,0
28.7499999999994,0,363.728259920695,0,0
28.7749999999994,0,363.728259920695,0,0
28.7999999999994,0,363.728259920695,0,0
28.8249999999994,0,363.728259920695,0,0
28.8499999999994,0,363.728259920695,0,0
28.8749999999994,0,363.728259920695,0,0
28.8999999999994,0,363.728259920695,0,0
28.9249999999994,0,363.728259920695,0,0
28.9499999999994,0,363.728259920695,0,0
28.9749999999994,0,363.728259920695,0,0
28.9999999999994,0,363.728259920695,0,0
29.0249999999994,0,363.728259920695,0,0
29.0499999999994,0,363.728259920695,0,0
29.0749999999994,0,363.728259920695,0,0
29.0999999999994,0,363.728259920695,0,0
29.1249999999994,0,363.728259920695,0,0
29.1499999999994,0,363.728259920695,0,0
29.1749999999994,0,363.728259920695,0,0
29.1999999999994,0,363.728259920695,0,0
29.2249999999994,0,363.728259920695,0,0
29.2499999999994,0,363.728259920695,0,0
29.2749999999994,0,363.728259920695,0,0
29.2999999999994,2,363.844402209505,0,0
29.3249999999994,0,363.960544498315,0,0
29.3499999999994,0,363.960544498315,0,0
29.3749999999994,0,363.960544498315,0,0
29.3999999999994,0,363.960544498315,0,0
29.4249999999994,0,363.960544498315,0,0
29.4499999999994,0,363.960544498315,0,0
29.4749999999994,0,363.960544498315,0,0
29.4999999999994,0,363.960544498315,0,0
29.5249999999994,0,363.960544498315,0,0
29.5499999999994,0,363.960544498315,0,0
29.5749999999994,0,363.960544498315,0,0
29.5999999999994,0,363.960544498315,0,0
29.6249999999994,0,363.960544498315,0,0
29.6499999999994,0,363.960544498315,0,0
29.6749999999994,0,363.960544498315,0,0
29.6999999999994,0,363.960544498315,0,0
29.7249999999994,0,363.960544498315,0,0
29.7499999999994,1,364.042669498315,0,0
29.7749999999994,0,364.124794498315,0,0
29.7999999999994,0,364.124794498315,0,0
29.8249999999994,0,364.124794498315,0,0

29.8499999999994,0,364.124794498315,0,0
29.8749999999994,0,364.124794498315,0,0
29.8999999999994,0,364.124794498315,0,0
29.9249999999994,0,364.124794498315,0,0
29.9499999999994,0,364.124794498315,0,0
29.9749999999994,0,364.124794498315,0,0
29.9999999999994,0,364.124794498315,0,0
30.0249999999994,0,364.124794498315,0,0
30.0499999999994,0,364.124794498315,0,0
30.0749999999994,0,364.124794498315,0,0
30.0999999999994,0,364.124794498315,0,0
30.1249999999994,0,364.124794498315,0,0
30.1499999999994,0,364.124794498315,0,0
30.1749999999994,0,364.124794498315,0,0
30.1999999999994,3,364.267039170886,0,0
30.2249999999994,0,364.409283843458,0,0
30.2499999999994,0,364.409283843458,0,0
30.2749999999993,0,364.409283843458,0,0
30.2999999999993,0,364.409283843458,0,0
30.3249999999993,0,364.409283843458,0,0
30.3499999999993,0,364.409283843458,0,0
30.3749999999993,0,364.409283843458,0,0
30.3999999999993,0,364.409283843458,0,0
30.4249999999993,0,364.409283843458,0,0
30.4499999999993,0,364.409283843458,0,0
30.4749999999993,0,364.409283843458,0,0
30.4999999999993,0,364.409283843458,0,0
30.5249999999993,0,364.409283843458,0,0
30.5499999999993,0,364.409283843458,0,0
30.5749999999993,0,364.409283843458,0,0
30.5999999999993,0,364.409283843458,0,0
30.6249999999993,0,364.409283843458,0,0
30.6499999999993,1,364.491408843458,0,0
30.6749999999993,0,364.573533843458,0,0
30.6999999999993,0,364.573533843458,0,0
30.7249999999993,0,364.573533843458,0,0
30.7499999999993,0,364.573533843458,0,0
30.7749999999993,0,364.573533843458,0,0
30.7999999999993,0,364.573533843458,0,0
30.8249999999993,0,364.573533843458,0,0
30.8499999999993,0,364.573533843458,0,0
30.8749999999993,0,364.573533843458,0,0
30.8999999999993,0,364.573533843458,0,0
30.9249999999993,0,364.573533843458,0,0
30.9499999999993,0,364.573533843458,0,0
30.9749999999993,0,364.573533843458,0,0
30.9999999999993,0,364.573533843458,0,0
31.0249999999993,0,364.573533843458,0,0
31.0499999999993,0,364.573533843458,0,0
31.0749999999993,0,364.573533843458,0,0
31.0999999999993,3,364.715778516029,0,0
31.1249999999993,0,364.858023188601,0,0

31.1499999999993,0,364.858023188601,0,0
31.1749999999993,0,364.858023188601,0,0
31.1999999999993,0,364.858023188601,0,0
31.2249999999993,0,364.858023188601,0,0
31.2499999999993,0,364.858023188601,0,0
31.2749999999993,0,364.858023188601,0,0
31.2999999999993,0,364.858023188601,0,0
31.3249999999993,0,364.858023188601,0,0
31.3499999999993,0,364.858023188601,0,0
31.3749999999993,0,364.858023188601,0,0
31.3999999999993,0,364.858023188601,0,0
31.4249999999993,0,364.858023188601,0,0
31.4499999999993,0,364.858023188601,0,0
31.4749999999993,0,364.858023188601,0,0
31.4999999999993,0,364.858023188601,0,0
31.5249999999993,0,364.858023188601,0,0
31.5499999999993,0,364.858023188601,0,0
31.5749999999993,0,364.858023188601,0,0
31.5999999999993,0,364.858023188601,0,0
31.6249999999993,0,364.858023188601,0,0
31.6499999999993,0,364.858023188601,0,0
31.6749999999993,0,364.858023188601,0,0
31.6999999999993,0,364.858023188601,0,0
31.7249999999993,0,364.858023188601,0,0
31.7499999999993,0,364.858023188601,0,0
31.7749999999993,0,364.858023188601,0,0
31.7999999999993,0,364.858023188601,0,0
31.8249999999993,0,364.858023188601,0,0
31.8499999999993,0,364.858023188601,0,0
31.8749999999993,0,364.858023188601,0,0
31.8999999999993,0,364.858023188601,0,0
31.9249999999993,0,364.858023188601,0,0
31.9499999999993,0,364.858023188601,0,0
31.9749999999993,0,364.858023188601,0,0
31.9999999999993,0,364.858023188601,0,0
32.0249999999993,0,364.858023188601,0,0
32.0499999999993,0,364.858023188601,0,0
32.0749999999993,0,364.858023188601,0,0
32.0999999999992,0,364.858023188601,0,0
32.1249999999992,0,364.858023188601,0,0
32.1499999999992,0,364.858023188601,0,0
32.1749999999992,0,364.858023188601,0,0
32.1999999999992,0,364.858023188601,0,0
32.2249999999992,0,364.858023188601,0,0
32.2499999999992,0,364.858023188601,0,0
32.2749999999992,0,364.858023188601,0,0
32.2999999999992,0,364.858023188601,0,0
32.3249999999992,0,364.858023188601,0,0
32.3499999999992,0,364.858023188601,0,0
32.3749999999992,0,364.858023188601,0,0
32.3999999999992,0,364.858023188601,0,0
32.4249999999992,0,364.858023188601,0,0

32.4499999999992,0,364.858023188601,0,0
32.4749999999992,0,364.858023188601,0,0
32.4999999999992,0,364.858023188601,0,0
32.5249999999992,0,364.858023188601,0,0
32.5499999999992,0,364.858023188601,0,0
32.5749999999992,0,364.858023188601,0,0
32.5999999999992,0,364.858023188601,0,0
32.6249999999992,0,364.858023188601,0,0
32.6499999999992,0,364.858023188601,0,0
32.6749999999992,0,364.858023188601,0,0
32.6999999999992,0,364.858023188601,0,0
32.7249999999992,0,364.858023188601,0,0
32.7499999999992,0,364.858023188601,0,0
32.7749999999992,0,364.858023188601,0,0
32.7999999999992,0,364.858023188601,0,0
32.8249999999992,0,364.858023188601,0,0
32.8499999999992,0,364.858023188601,0,0
32.8749999999992,0,364.858023188601,0,0
32.8999999999992,1,364.940148188601,0,0
32.9249999999992,1,365.104398188601,0,0
32.9499999999992,0,365.186523188601,0,0
32.9749999999992,0,365.186523188601,0,0
32.9999999999992,0,365.186523188601,0,0
33.0249999999992,0,365.186523188601,0,0
33.0499999999992,0,365.186523188601,0,0
33.0749999999992,0,365.186523188601,0,0
33.0999999999992,0,365.186523188601,0,0
33.1249999999992,0,365.186523188601,0,0
33.1499999999992,0,365.186523188601,0,0
33.1749999999992,0,365.186523188601,0,0
33.1999999999992,0,365.186523188601,0,0
33.2249999999992,0,365.186523188601,0,0
33.2499999999992,0,365.186523188601,0,0
33.2749999999992,0,365.186523188601,0,0
33.2999999999992,0,365.186523188601,0,0
33.3249999999992,0,365.186523188601,0,0
33.3499999999992,0,365.186523188601,0,0
33.3749999999992,0,365.186523188601,0,0
33.3999999999992,0,365.186523188601,0,0
33.4249999999992,0,365.186523188601,0,0
33.4499999999992,0,365.186523188601,0,0
33.4749999999992,0,365.186523188601,0,0
33.4999999999992,0,365.186523188601,0,0
33.5249999999992,0,365.186523188601,0,0
33.5499999999992,0,365.186523188601,0,0
33.5749999999992,0,365.186523188601,0,0
33.5999999999992,0,365.186523188601,0,0
33.6249999999992,0,365.186523188601,0,0
33.6499999999992,0,365.186523188601,0,0
33.6749999999992,0,365.186523188601,0,0
33.6999999999992,0,365.186523188601,0,0
33.7249999999992,0,365.186523188601,0,0

33.7499999999992,0,365.186523188601,0,0
33.7749999999992,0,365.186523188601,0,0
33.7999999999992,0,365.186523188601,0,0
33.8249999999992,0,365.186523188601,0,0
33.8499999999991,0,365.186523188601,0,0
33.8749999999991,0,365.186523188601,0,0
33.8999999999991,0,365.186523188601,0,0
33.9249999999991,0,365.186523188601,0,0
33.9499999999991,0,365.186523188601,0,0
33.9749999999991,0,365.186523188601,0,0
33.9999999999991,0,365.186523188601,0,0
34.0249999999991,0,365.186523188601,0,0
34.0499999999991,0,365.186523188601,0,0
34.0749999999991,0,365.186523188601,0,0
34.0999999999991,0,365.186523188601,0,0
34.1249999999991,0,365.186523188601,0,0
34.1499999999991,0,365.186523188601,0,0
34.1749999999991,0,365.186523188601,0,0
34.1999999999991,0,365.186523188601,0,0
34.2249999999991,0,365.186523188601,0,0
34.2499999999991,0,365.186523188601,0,0
34.2749999999991,0,365.186523188601,0,0
34.2999999999991,0,365.186523188601,0,0
34.3249999999991,0,365.186523188601,0,0
34.3499999999991,0,365.186523188601,0,0
34.3749999999991,0,365.186523188601,0,0
34.3999999999991,0,365.186523188601,0,0
34.4249999999991,0,365.186523188601,0,0
34.4499999999991,0,365.186523188601,0,0
34.4749999999991,0,365.186523188601,0,0
34.4999999999991,0,365.186523188601,0,0
34.5249999999991,0,365.186523188601,0,0
34.5499999999991,0,365.186523188601,0,0
34.5749999999991,0,365.186523188601,0,0
34.5999999999991,0,365.186523188601,0,0
34.6249999999991,0,365.186523188601,0,0
34.6499999999991,0,365.186523188601,0,0
34.6749999999991,0,365.186523188601,0,0
34.6999999999991,0,365.186523188601,0,0
34.7249999999991,0,365.186523188601,0,0
34.7499999999991,0,365.186523188601,0,0
34.7749999999991,0,365.186523188601,0,0
34.7999999999991,0,365.186523188601,0,0
34.8249999999991,0,365.186523188601,0,0
34.8499999999991,0,365.186523188601,0,0
34.8749999999991,0,365.186523188601,0,0
34.8999999999991,0,365.186523188601,0,0
34.9249999999991,0,365.186523188601,0,0
34.9499999999991,0,365.186523188601,0,0
34.9749999999991,0,365.186523188601,0,0
34.9999999999991,0,365.186523188601,0,0
35.0249999999991,0,365.186523188601,0,0

35.0499999999991,0,365.186523188601,0,0
35.0749999999991,0,365.186523188601,0,0
35.0999999999991,0,365.186523188601,0,0
35.1249999999991,0,365.186523188601,0,0
35.1499999999991,0,365.186523188601,0,0
35.1749999999991,0,365.186523188601,0,0
35.1999999999991,0,365.186523188601,0,0
35.2249999999991,0,365.186523188601,0,0
35.2499999999991,0,365.186523188601,0,0
35.2749999999991,0,365.186523188601,0,0
35.2999999999991,0,365.186523188601,0,0
35.3249999999991,0,365.186523188601,0,0
35.3499999999991,0,365.186523188601,0,0
35.3749999999991,0,365.186523188601,0,0
35.3999999999991,0,365.186523188601,0,0
35.4249999999991,0,365.186523188601,0,0
35.4499999999991,0,365.186523188601,0,0
35.4749999999991,0,365.186523188601,0,0
35.4999999999991,0,365.186523188601,0,0
35.5249999999991,0,365.186523188601,0,0
35.5499999999991,0,365.186523188601,0,0
35.5749999999991,0,365.186523188601,0,0
35.599999999999,0,365.186523188601,0,0
35.624999999999,0,365.186523188601,0,0
35.649999999999,0,365.186523188601,0,0
35.674999999999,0,365.186523188601,0,0
35.699999999999,0,365.186523188601,0,0
35.724999999999,0,365.186523188601,0,0
35.749999999999,0,365.186523188601,0,0
35.774999999999,0,365.186523188601,0,0
35.799999999999,0,365.186523188601,0,0
35.824999999999,0,365.186523188601,0,0
35.849999999999,0,365.186523188601,0,0
35.874999999999,0,365.186523188601,0,0
35.899999999999,0,365.186523188601,0,0
35.924999999999,0,365.186523188601,0,0
35.949999999999,0,365.186523188601,0,0
35.974999999999,0,365.186523188601,0,0
35.999999999999,0,365.186523188601,0,0
36.024999999999,0,365.186523188601,0,0
36.049999999999,0,365.186523188601,0,0
36.074999999999,0,365.186523188601,0,0
36.099999999999,0,365.186523188601,0,0
36.124999999999,0,365.186523188601,0,0
36.149999999999,0,365.186523188601,0,0
36.174999999999,0,365.186523188601,0,0
36.199999999999,0,365.186523188601,0,0
36.224999999999,0,365.186523188601,0,0
36.249999999999,0,365.186523188601,0,0
36.274999999999,0,365.186523188601,0,0
36.299999999999,0,365.186523188601,0,0
36.324999999999,0,365.186523188601,0,0

36.349999999999,0,365.186523188601,0,0
36.374999999999,0,365.186523188601,0,0
36.399999999999,0,365.186523188601,0,0
36.424999999999,0,365.186523188601,0,0
36.449999999999,0,365.186523188601,0,0
36.474999999999,0,365.186523188601,0,0
36.499999999999,0,365.186523188601,0,0
36.524999999999,0,365.186523188601,0,0
36.549999999999,0,365.186523188601,0,0
36.574999999999,0,365.186523188601,0,0
36.599999999999,0,365.186523188601,0,0
36.624999999999,0,365.186523188601,0,0
36.649999999999,0,365.186523188601,0,0
36.674999999999,0,365.186523188601,0,0
36.699999999999,0,365.186523188601,0,0
36.724999999999,0,365.186523188601,0,0
36.749999999999,0,365.186523188601,0,0
36.774999999999,0,365.186523188601,0,0
36.799999999999,0,365.186523188601,0,0
36.824999999999,0,365.186523188601,0,0
36.849999999999,0,365.186523188601,0,0
36.874999999999,0,365.186523188601,0,0
36.899999999999,0,365.186523188601,0,0
36.924999999999,0,365.186523188601,0,0
36.949999999999,1,365.268648188601,0,0
36.974999999999,0,365.350773188601,0,0
36.999999999999,0,365.350773188601,0,0
37.024999999999,0,365.350773188601,0,0
37.049999999999,0,365.350773188601,0,0
37.074999999999,0,365.350773188601,0,0
37.099999999999,0,365.350773188601,0,0
37.124999999999,0,365.350773188601,0,0
37.149999999999,0,365.350773188601,0,0
37.174999999999,0,365.350773188601,0,0
37.199999999999,0,365.350773188601,0,0
37.224999999999,0,365.350773188601,0,0
37.249999999999,0,365.350773188601,0,0
37.274999999999,0,365.350773188601,0,0
37.299999999999,0,365.350773188601,0,0
37.324999999999,0,365.350773188601,0,0
37.349999999999,0,365.350773188601,0,0
37.3749999999989,0,365.350773188601,0,0
37.3999999999989,0,365.350773188601,0,0
37.4249999999989,0,365.350773188601,0,0
37.4499999999989,0,365.350773188601,0,0
37.4749999999989,0,365.350773188601,0,0
37.4999999999989,0,365.350773188601,0,0
37.5249999999989,0,365.350773188601,0,0
37.5499999999989,0,365.350773188601,0,0
37.5749999999989,0,365.350773188601,0,0
37.5999999999989,0,365.350773188601,0,0
37.6249999999989,0,365.350773188601,0,0

37.6499999999989,0,365.350773188601,0,0
37.6749999999989,0,365.350773188601,0,0
37.6999999999989,0,365.350773188601,0,0
37.7249999999989,0,365.350773188601,0,0
37.7499999999989,0,365.350773188601,0,0
37.7749999999989,0,365.350773188601,0,0
37.7999999999989,0,365.350773188601,0,0
37.8249999999989,0,365.350773188601,0,0
37.8499999999989,0,365.350773188601,0,0
37.8749999999989,0,365.350773188601,0,0
37.8999999999989,0,365.350773188601,0,0
37.9249999999989,0,365.350773188601,0,0
37.9499999999989,0,365.350773188601,0,0
37.9749999999989,0,365.350773188601,0,0
37.9999999999989,0,365.350773188601,0,0
38.0249999999989,0,365.350773188601,0,0
38.0499999999989,0,365.350773188601,0,0
38.0749999999989,0,365.350773188601,0,0
38.0999999999989,0,365.350773188601,0,0
38.1249999999989,0,365.350773188601,0,0
38.1499999999989,0,365.350773188601,0,0
38.1749999999989,0,365.350773188601,0,0
38.1999999999989,0,365.350773188601,0,0
38.2249999999989,0,365.350773188601,0,0
38.2499999999989,0,365.350773188601,0,0
38.2749999999989,0,365.350773188601,0,0
38.2999999999989,0,365.350773188601,0,0
38.3249999999989,0,365.350773188601,0,0
38.3499999999989,0,365.350773188601,0,0
38.3749999999989,0,365.350773188601,0,0
38.3999999999989,0,365.350773188601,0,0
38.4249999999989,0,365.350773188601,0,0
38.4499999999989,0,365.350773188601,0,0
38.4749999999989,0,365.350773188601,0,0
38.4999999999989,0,365.350773188601,0,0
38.5249999999989,0,365.350773188601,0,0
38.5499999999989,0,365.350773188601,0,0
38.5749999999989,0,365.350773188601,0,0
38.5999999999989,0,365.350773188601,0,0
38.6249999999989,0,365.350773188601,0,0
38.6499999999989,0,365.350773188601,0,0
38.6749999999989,0,365.350773188601,0,0
38.6999999999989,0,365.350773188601,0,0
38.7249999999989,0,365.350773188601,0,0
38.7499999999989,1,365.432898188601,0,0
38.7749999999989,0,365.515023188601,0,0
38.7999999999989,0,365.515023188601,0,0
38.8249999999989,0,365.515023188601,0,0
38.8499999999989,0,365.515023188601,0,0
38.8749999999989,0,365.515023188601,0,0
38.8999999999989,0,365.515023188601,0,0
38.9249999999989,0,365.515023188601,0,0

38.9499999999989,0,365.515023188601,0,0
38.9749999999989,1,365.597148188601,0,0
38.9999999999989,0,365.679273188601,0,0
39.0249999999989,0,365.679273188601,0,0
39.0499999999989,0,365.679273188601,0,0
39.0749999999989,0,365.679273188601,0,0
39.0999999999989,0,365.679273188601,0,0
39.1249999999988,0,365.679273188601,0,0
39.1499999999988,0,365.679273188601,0,0
39.1749999999988,0,365.679273188601,0,0
39.1999999999988,0,365.679273188601,0,0
39.2249999999988,0,365.679273188601,0,0
39.2499999999988,0,365.679273188601,0,0
39.2749999999988,0,365.679273188601,0,0
39.2999999999988,0,365.679273188601,0,0
39.3249999999988,0,365.679273188601,0,0
39.3499999999988,0,365.679273188601,0,0
39.3749999999988,0,365.679273188601,0,0
39.3999999999988,0,365.679273188601,0,0
39.4249999999988,0,365.679273188601,0,0
39.4499999999988,0,365.679273188601,0,0
39.4749999999988,0,365.679273188601,0,0
39.4999999999988,0,365.679273188601,0,0
39.5249999999988,0,365.679273188601,0,0
39.5499999999988,0,365.679273188601,0,0
39.5749999999988,0,365.679273188601,0,0
39.5999999999988,0,365.679273188601,0,0
39.6249999999988,1,365.761398188601,0,0
39.6499999999988,0,365.843523188601,0,0
39.6749999999988,0,365.843523188601,0,0
39.6999999999988,0,365.843523188601,0,0
39.7249999999988,1,365.925648188601,0,0
39.7499999999988,0,366.007773188601,0,0
39.7749999999988,0,366.007773188601,0,0
39.7999999999988,0,366.007773188601,0,0
39.8249999999988,0,366.007773188601,0,0
39.8499999999988,0,366.007773188601,0,0
39.8749999999988,0,366.007773188601,0,0
39.8999999999988,0,366.007773188601,0,0
39.9249999999988,0,366.007773188601,0,0
39.9499999999988,0,366.007773188601,0,0
39.9749999999988,0,366.007773188601,0,0
39.9999999999988,0,366.007773188601,0,0
40.0249999999988,0,366.007773188601,0,0
40.0499999999988,0,366.007773188601,0,0
40.0749999999988,0,366.007773188601,0,0
40.0999999999988,0,366.007773188601,0,0
40.1249999999988,0,366.007773188601,0,0
40.1499999999988,0,366.007773188601,0,0
40.1749999999988,0,366.007773188601,0,0
40.1999999999988,0,366.007773188601,0,0
40.2249999999988,0,366.007773188601,0,0

```
40.2499999999988,0,366.007773188601,0,0
40.2749999999988,0,366.007773188601,0,0
40.2999999999988,0,366.007773188601,0,0
40.3249999999988,0,366.007773188601,0,0
40.3499999999988,0,366.007773188601,0,0
40.3749999999988,0,366.007773188601,0,0
40.3999999999988,1,366.089898188601,0,0
40.4249999999988,0,366.172023188601,0,0
40.4499999999988,0,366.172023188601,0,0
40.4749999999988,0,366.172023188601,0,0
40.4999999999988,0,366.172023188601,0,0
40.5249999999988,0,366.172023188601,0,0
40.5499999999988,0,366.172023188601,0,0
40.5749999999988,1,366.254148188601,0,0
40.5999999999988,0,366.336273188601,0,0
40.6249999999988,0,366.336273188601,0,0
40.6499999999988,0,366.336273188601,0,0
40.6749999999988,0,366.336273188601,0,0
40.6999999999988,0,366.336273188601,0,0
40.7249999999988,0,366.336273188601,0,0
40.7499999999988,0,366.336273188601,0,0
40.7749999999988,0,366.336273188601,0,0
40.7999999999988,0,366.336273188601,0,0
40.8249999999988,0,366.336273188601,0,0
40.8499999999988,0,366.336273188601,0,0
40.8749999999987,0,366.336273188601,0,0
40.8999999999987,0,366.336273188601,0,0
40.9249999999987,0,366.336273188601,0,0
40.9499999999987,0,366.336273188601,0,0
40.9749999999987,0,366.336273188601,0,0
40.9999999999987,1,366.418398188601,0,0
41.0249999999987,0,366.500523188601,0,0
41.0499999999987,0,366.500523188601,0,0
41.0749999999987,0,366.500523188601,0,0
41.0999999999987,0,366.500523188601,0,0
41.1249999999987,0,366.500523188601,0,0
41.1499999999987,3,366.642767861173,0,0
41.1749999999987,9,367.031387533745,0,0
41.1999999999987,16,367.606262533745,32.5677373169553,1
41.2249999999987,18,368.283189400174,32.5677373169553,1
41.2499999999987,19,368.989590842342,32.5677373169553,1
41.2749999999987,25,369.758190418081,32.5677373169553,1
41.2999999999987,29,370.611072077866,32.5677373169553,1
41.3249999999987,26,371.472085715206,32.5677373169553,1
41.3499999999987,24,372.293171383012,32.5677373169553,1
41.3749999999987,21,373.071844102212,32.5677373169553,1
41.3999999999987,32,373.9127572864,32.5677373169553,1
41.4249999999987,31,374.834579089937,32.5677373169553,1
41.4499999999987,34,375.770698662599,32.5677373169553,1
41.4749999999987,27,376.676299604678,32.5677373169553,1
41.4999999999987,28,377.537598275235,32.5677373169553,1
41.5249999999987,35,378.458020980264,32.5677373169553,1
```

41.5499999999987,38,379.450131532445,32.5677373169553,1
41.5749999999987,40,380.475788138122,32.5677373169553,1
41.5999999999987,41,381.521048821804,32.5677373169553,1
41.6249999999987,37,382.546452272603,32.5677373169553,1
41.6499999999987,36,383.538749145404,32.5677373169553,1
41.6749999999987,33,384.503271352751,32.5677373169553,1
41.6999999999987,33,385.446815767445,32.5677373169553,1
41.7249999999987,32,386.383157130031,32.5677373169553,1
41.7499999999987,32,387.312295440511,32.5677373169553,1
41.7749999999987,31,388.234117244048,32.5677373169553,1
41.7999999999987,31,389.148622540642,32.5677373169553,1
41.8249999999987,32,390.070444344179,32.5677373169553,1
41.8499999999987,40,391.054417605102,32.5677373169553,1
41.8749999999987,47,392.136842344842,32.5677373169553,1
41.8999999999987,44,393.244618600716,32.5677373169553,1
41.9249999999987,45,394.340285470488,32.5677373169553,1
41.9499999999987,41,395.417053296444,32.5677373169553,1
41.9749999999987,49,396.517784874443,32.5677373169553,1
41.9999999999987,41,397.618516452442,32.5677373169553,1
42.0249999999987,30,398.594190180793,32.5677373169553,1
42.0499999999987,31,399.501259979441,32.5677373169553,1
42.0749999999987,23,400.352370291591,32.5677373169553,1
42.0999999999987,22,401.131428349718,32.5677373169553,1
42.1249999999987,18,401.865055610424,32.5677373169553,1
42.1499999999987,16,402.541982476853,32.5677373169553,1
42.1749999999987,16,403.198982476853,32.5677373169553,1
42.1999999999987,16,403.855982476853,32.5677373169553,1
42.2249999999987,21,404.560826505801,32.5677373169553,1
42.2499999999987,26,405.355927512303,32.5677373169553,1
42.2749999999987,32,406.239253645097,32.5677373169553,1
42.2999999999987,34,407.1826897247,32.5677373169553,1
42.3249999999987,32,408.126125804304,32.5677373169553,1
42.3499999999987,30,409.040512109895,32.5677373169553,1
42.3749999999987,26,409.9090862378,32.5677373169553,1
42.3999999999987,20,410.695117380658,32.5677373169553,1
42.4249999999987,12,411.346880891105,32.5677373169553,1
42.4499999999987,7,411.84865256267,32.5677373169553,1
42.4749999999987,3,412.208179561663,32.5677373169553,1
42.4999999999987,4,412.514674234234,32.5677373169553,1
42.5249999999987,10,412.938626287076,32.5677373169553,1
42.5499999999987,12,413.48281768506,32.5677373169553,1
42.5749999999987,12,414.051796375346,32.5677373169553,1
42.5999999999987,14,414.643569333378,32.5677373169553,1
42.6249999999987,20,415.318127111572,32.5677373169553,1
42.6499999999986,23,416.079258940728,32.5677373169553,1
42.6749999999986,22,416.858316998855,32.5677373169553,1
42.6999999999986,25,417.654142393131,32.5677373169553,1
42.7249999999986,29,418.507024052917,32.5677373169553,1
42.7499999999986,27,419.376014730418,32.5677373169553,1
42.7749999999986,25,420.213373748132,32.5677373169553,1
42.7999999999986,26,421.042755725686,32.5677373169553,1
42.8249999999986,29,421.903769363026,32.5677373169553,1

```
42.8499999999986,37,422.845572895613,32.5677373169553,1
42.8749999999986,34,423.823986692777,32.5677373169553,1
42.8999999999986,35,424.788711669329,32.5677373169553,1
42.9249999999986,33,425.746341928863,32.5677373169553,1
42.9499999999986,36,426.71086413621,32.5677373169553,1
42.9749999999986,35,427.689472188397,32.5677373169553,1
42.9999999999986,35,428.661188292771,32.5677373169553,1
43.0249999999986,37,429.646593217759,32.5677373169553,1
43.0499999999986,33,430.617912297906,32.5677373169553,1
43.0749999999986,33,431.5614567126,32.5677373169553,1
43.0999999999986,39,432.546099380566,32.5677373169553,1
43.1249999999986,36,433.551719841184,32.5677373169553,1
43.1499999999986,31,434.501722489481,32.5677373169553,1
43.1749999999986,24,435.361303828031,32.5677373169553,1
43.1999999999986,23,436.157490182135,32.5677373169553,1
43.2249999999986,27,436.978081863702,32.5677373169553,1
43.2499999999986,24,437.807144571669,32.5677373169553,1
43.2749999999986,24,438.611801952173,32.5677373169553,1
43.2999999999986,22,439.399331036701,32.5677373169553,1
43.3249999999986,23,440.178389094828,32.5677373169553,1
43.3499999999986,25,440.98287175868,32.5677373169553,1
43.3749999999986,27,441.820230776395,32.5677373169553,1
43.3999999999986,23,442.640822457962,32.5677373169553,1
43.4249999999986,26,443.453437099368,32.5677373169553,1
43.4499999999986,25,444.282819076922,32.5677373169553,1
43.4749999999986,24,445.095772767174,32.5677373169553,1
43.4999999999986,23,445.891959121278,32.5677373169553,1
43.5249999999986,27,446.712550802845,32.5677373169553,1
43.5499999999986,26,447.558041798113,32.5677373169553,1
43.5749999999986,27,448.403532793382,32.5677373169553,1
43.5999999999986,22,449.215467205372,32.5677373169553,1
43.6249999999986,23,449.9945252635,32.5677373169553,1
43.6499999999986,23,450.782240591204,32.5677373169553,1
43.6749999999986,21,451.552442284004,32.5677373169553,1
43.6999999999986,23,452.322643976805,32.5677373169553,1
43.7249999999986,20,453.083775805961,32.5677373169553,1
43.7499999999986,19,453.809024547004,32.5677373169553,1
43.7749999999986,20,454.534273288046,32.5677373169553,1
43.7999999999986,18,455.24997431978,32.5677373169553,1
43.8249999999986,17,455.937011235714,32.5677373169553,1
43.8499999999986,18,456.624048151647,32.5677373169553,1
43.8749999999986,16,457.300975018077,32.5677373169553,1
43.8999999999986,12,457.91396436322,32.5677373169553,1
43.9249999999986,11,458.470831519271,32.5677373169553,1
43.9499999999986,10,459.00291138302,32.5677373169553,1
43.9749999999986,9,459.508988435862,32.5677373169553,1
43.9999999999986,9,460.001738435862,32.5677373169553,1
44.0249999999986,10,460.507815488703,32.5677373169553,1
44.0499999999986,10,461.027219594386,32.5677373169553,1
44.0749999999986,10,461.546623700068,32.5677373169553,1
44.0999999999986,9,462.05270075291,32.5677373169553,1
44.1249999999986,8,462.531360330529,0,0
```

44.1499999999986,4,462.927894908149,0,0
44.1749999999986,1,463.174269908149,0,0
44.1999999999986,1,463.338519908149,0,0
44.2249999999986,0,463.420644908149,0,0
44.2499999999986,0,463.420644908149,0,0
44.2749999999986,0,463.420644908149,0,0
44.2999999999986,0,463.420644908149,0,0
44.3249999999986,0,463.420644908149,0,0
44.3499999999986,0,463.420644908149,0,0
44.3749999999986,0,463.420644908149,0,0
44.3999999999985,0,463.420644908149,0,0
44.4249999999985,0,463.420644908149,0,0
44.4499999999985,0,463.420644908149,0,0
44.4749999999985,0,463.420644908149,0,0
44.4999999999985,0,463.420644908149,0,0
44.5249999999985,0,463.420644908149,0,0
44.5499999999985,0,463.420644908149,0,0
44.5749999999985,0,463.420644908149,0,0
44.5999999999985,1,463.502769908149,0,0
44.6249999999985,0,463.584894908149,0,0
44.6499999999985,0,463.584894908149,0,0
44.6749999999985,0,463.584894908149,0,0
44.6999999999985,0,463.584894908149,0,0
44.7249999999985,0,463.584894908149,0,0
44.7499999999985,0,463.584894908149,0,0
44.7749999999985,0,463.584894908149,0,0
44.7999999999985,0,463.584894908149,0,0
44.8249999999985,0,463.584894908149,0,0
44.8499999999985,0,463.584894908149,0,0
44.8749999999985,0,463.584894908149,0,0
44.8999999999985,0,463.584894908149,0,0
44.9249999999985,0,463.584894908149,0,0
44.9499999999985,0,463.584894908149,0,0
44.9749999999985,0,463.584894908149,0,0
44.9999999999985,0,463.584894908149,0,0
45.0249999999985,0,463.584894908149,0,0
45.0499999999985,0,463.584894908149,0,0
45.0749999999985,0,463.584894908149,0,0
45.0999999999985,0,463.584894908149,0,0
45.1249999999985,0,463.584894908149,0,0
45.1499999999985,0,463.584894908149,0,0
45.1749999999985,0,463.584894908149,0,0
45.1999999999985,0,463.584894908149,0,0
45.2249999999985,0,463.584894908149,0,0
45.2499999999985,0,463.584894908149,0,0
45.2749999999985,0,463.584894908149,0,0
45.2999999999985,0,463.584894908149,0,0
45.3249999999985,0,463.584894908149,0,0
45.3499999999985,0,463.584894908149,0,0
45.3749999999985,0,463.584894908149,0,0
45.3999999999985,0,463.584894908149,0,0
45.4249999999985,0,463.584894908149,0,0

45.4499999999985,0,463.584894908149,0,0
45.4749999999985,0,463.584894908149,0,0
45.4999999999985,0,463.584894908149,0,0
45.5249999999985,0,463.584894908149,0,0
45.5499999999985,0,463.584894908149,0,0
45.5749999999985,0,463.584894908149,0,0
45.5999999999985,0,463.584894908149,0,0
45.6249999999985,0,463.584894908149,0,0
45.6499999999985,0,463.584894908149,0,0
45.6749999999985,0,463.584894908149,0,0
45.6999999999985,0,463.584894908149,0,0
45.7249999999985,0,463.584894908149,0,0
45.7499999999985,0,463.584894908149,0,0
45.7749999999985,0,463.584894908149,0,0
45.7999999999985,0,463.584894908149,0,0
45.8249999999985,0,463.584894908149,0,0
45.8499999999985,0,463.584894908149,0,0
45.8749999999985,0,463.584894908149,0,0
45.8999999999985,0,463.584894908149,0,0
45.9249999999985,0,463.584894908149,0,0
45.9499999999985,0,463.584894908149,0,0
45.9749999999985,0,463.584894908149,0,0
45.9999999999985,0,463.584894908149,0,0
46.0249999999985,0,463.584894908149,0,0
46.0499999999985,0,463.584894908149,0,0
46.0749999999985,0,463.584894908149,0,0
46.0999999999985,0,463.584894908149,0,0
46.1249999999985,0,463.584894908149,0,0
46.1499999999985,0,463.584894908149,0,0
46.1749999999984,0,463.584894908149,0,0
46.1999999999984,0,463.584894908149,0,0
46.2249999999984,0,463.584894908149,0,0
46.2499999999984,0,463.584894908149,0,0
46.2749999999984,0,463.584894908149,0,0
46.2999999999984,0,463.584894908149,0,0
46.3249999999984,0,463.584894908149,0,0
46.3499999999984,0,463.584894908149,0,0
46.3749999999984,0,463.584894908149,0,0
46.3999999999984,3,463.727139580721,0,0
46.4249999999984,0,463.869384253292,0,0
46.4499999999984,0,463.869384253292,0,0
46.4749999999984,0,463.869384253292,0,0
46.4999999999984,0,463.869384253292,0,0
46.5249999999984,0,463.869384253292,0,0
46.5499999999984,0,463.869384253292,0,0
46.5749999999984,0,463.869384253292,0,0
46.5999999999984,0,463.869384253292,0,0
46.6249999999984,0,463.869384253292,0,0
46.6499999999984,0,463.869384253292,0,0
46.6749999999984,0,463.869384253292,0,0
46.6999999999984,0,463.869384253292,0,0
46.7249999999984,0,463.869384253292,0,0

```
46.7499999999984,0,463.869384253292,0,0
46.7749999999984,0,463.869384253292,0,0
```
