# Supplementary material for: Electronic Cigarette Topography in the Natural Environment
Source: PLoS One. 2015 Jun 8;10(6):e0129296. doi: 10.1371/journal.pone.0129296 (PMC4460076; doi:10.1371/journal.pone.0129296)
Supplement: S2 File — This file contains the data used to generate Fig 4, describing the puff topography. (PDF) [file pone.0129296.s002.pdf]

Subject	Number of Puffs [-/subject]	Mean Puff Duration [s]	Puff Duration StdDev [s]	Mean Puff Flow [ml/s]	Puff Flow StdDev [ml/s]	Mean Puff Volume [ml]	Puff Vol StdDev [ml]
1	1091	0.93	0.98	23.63	12.11	29.17	37.23
2	216	2.30	1.23	35.27	8.08	82.91	48.34
3	84	4.04	2.21	33.94	7.13	144.07	96.34
4	53	0.74	0.53	41.02	12.69	29.29	19.02
5	126	2.33	1.28	29.66	7.84	72.01	43.61
6	105	5.12	9.11	28.45	15.43	128.08	160.45
7	258	6.88	3.24	44.26	12.57	309.77	174.69
8	24	4.83	1.54	35.53	6.68	166.65	45.30
9	58	1.87	0.47	41.54	8.82	76.75	27.37
10	106	4.83	2.13	39.57	8.97	200.33	106.75
11	126	6.72	3.08	32.07	5.04	219.14	108.02
12	213	1.42	0.84	29.69	8.75	44.36	29.89
13	60	2.89	0.99	33.11	6.79	97.32	40.05
14	103	2.46	1.49	38.67	9.16	97.09	82.00
15	128	4.48	1.71	33.77	6.15	147.42	52.23
16	420	3.65	2.00	33.99	9.53	128.76	80.05
17	469	3.72	1.63	101.83	32.90	388.37	223.34
18	817	4.39	2.32	24.38	6.04	115.90	65.30
20	71	5.20	1.99	34.62	3.83	179.46	70.25
21	57	2.27	0.63	28.81	4.77	65.31	19.53
22	134	2.17	0.46	33.03	5.78	70.42	12.37
