# Supplementary material for: Electronic Cigarette Topography in the Natural Environment
Source: PLoS One. 2015 Jun 8;10(6):e0129296. doi: 10.1371/journal.pone.0129296 (PMC4460076; doi:10.1371/journal.pone.0129296)
Supplement: S3 File — This file contains the data used to generate Fig 5, Panel A, describing Subject 12, Puffing Session 7. (PDF) [file pone.0129296.s003.pdf]

RelativeTime,CleanData,AccumulatedVolume,MeanPuffFlowRate,PuffFla
g
0,1,0.16425,0,0
0.025,1,0.3285,0,0
0.05,2,0.52676728880989,0,0
0.075,1,0.725034577619781,0,0
0.1,1,0.889284577619781,0,0
0.125,1,1.05353457761978,0,0
0.15,1,1.21778457761978,0,0
0.175,1,1.38203457761978,0,0
0.2,1,1.54628457761978,0,0
0.225,1,1.71053457761978,0,0
0.25,1,1.87478457761978,0,0
0.275,1,2.03903457761978,0,0
0.3,1,2.20328457761978,0,0
0.325,1,2.36753457761978,0,0
0.35,1,2.53178457761978,0,0
0.375,1,2.69603457761978,0,0
0.4,1,2.86028457761978,0,0
0.425,1,3.02453457761978,0,0
0.45,1,3.18878457761978,0,0
0.475,1,3.35303457761978,0,0
0.5,1,3.51728457761978,0,0
0.525,0,3.59940957761978,0,0
0.55,1,3.68153457761978,0,0
0.575,1,3.84578457761978,0,0
0.6,1,4.01003457761978,0,0
0.625,1,4.17428457761978,0,0
0.65,1,4.33853457761978,0,0
0.675,1,4.50278457761978,0,0
0.7,1,4.66703457761978,0,0
0.725,0,4.74915957761978,0,0
0.75,0,4.74915957761978,0,0
0.775,0,4.74915957761978,0,0
0.8,1,4.83128457761978,0,0
0.825,1,4.99553457761978,0,0
0.85,1,5.15978457761978,0,0
0.875,1,5.32403457761978,0,0
0.9,1,5.48828457761978,0,0
0.925,1,5.65253457761978,0,0
0.950000000000001,1,5.81678457761978,0,0
0.975000000000001,1,5.98103457761978,0,0
1,1,6.14528457761978,0,0
1.025,1,6.30953457761978,0,0
1.05,1,6.47378457761978,0,0
1.075,1,6.63803457761978,0,0
1.1,1,6.80228457761978,0,0
1.125,1,6.96653457761978,0,0
1.15,1,7.13078457761978,0,0
1.175,2,7.32905186642967,0,0
1.2,3,7.58743882781115,0,0
1.225,4,7.89393350038275,0,0

1.25,4,8.22243350038275,0,0
1.275,5,8.57032058303492,0,0
1.3,7,8.97123999210827,0,0
1.325,8,9.42080689614923,0,0
1.35,9,9.89946647376901,0,0
1.375,8,10.3781260513888,0,0
1.4,8,10.8426952066284,0,0
1.425,8,11.3072643618679,0,0
1.45,9,11.7859239394877,0,0
1.475,9,12.2786739394877,0,0
1.5,9,12.7714239394877,0,0
1.525,9,13.2641739394877,0,0
1.55,9,13.7569239394877,0,0
1.575,8,14.2355835171075,0,0
1.6,8,14.700152672347,0,0
1.625,9,15.1788122499668,0,0
1.65,8,15.6574718275866,0,0
1.675,8,16.1220409828262,0,0
1.7,8,16.5866101380657,0,0
1.725,8,17.0511792933053,0,0
1.75,7,17.5007461973462,0,0
1.775,5,17.9016656064196,0,0
1.8,5,18.2689397717239,0,0
1.825,6,18.6537411995022,0,0
1.85,6,19.0560698897543,0,0
1.875,5,19.4408713175326,0,0
1.9,5,19.8081454828369,0,0
1.925,4,20.1560325654891,0,0
1.95,5,20.5039196481412,0,0
1.975,5,20.8711938134456,0,0
2,5,21.2384679787499,0,0
2.025,4,21.5863550614021,0,0
2.05,4,21.9148550614021,0,0
2.075,4,22.2433550614021,0,0
2.1,4,22.5718550614021,0,0
2.125,5,22.9197421440543,0,0
2.15,5,23.2870163093586,0,0
2.175,5,23.6542904746629,0,0
2.2,5,24.0215646399673,0,0
2.225,4,24.3694517226195,0,0
2.25,4,24.6979517226195,0,0
2.275,3,25.0044463951911,0,0
2.3,2,25.2628333565725,0,0
2.325,2,25.4951179341923,0,0
2.35,2,25.7274025118121,0,0
2.375,2,25.9596870894319,0,0
2.4,3,26.2180740508134,0,0
2.425,2,26.4764610121949,0,0
2.45,2,26.7087455898146,0,0
2.475,3,26.9671325511961,0,0
2.5,3,27.2516218963393,0,0
2.525,3,27.5361112414825,0,0

2.54999999999999,3,27.8206005866257,0,0
2.57499999999999,3,28.1050899317689,0,0
2.59999999999999,3,28.3895792769121,0,0
2.62499999999999,3,28.6740686220553,0,0
2.64999999999999,2,28.9324555834367,0,0
2.67499999999999,2,29.1647401610565,0,0
2.69999999999999,3,29.423127122438,0,0
2.72499999999999,3,29.7076164675812,0,0
2.74999999999999,1,29.9319861401528,0,0
2.77499999999999,1,30.0962361401528,0,0
2.79999999999999,1,30.2604861401528,0,0
2.82499999999999,1,30.4247361401528,0,0
2.84999999999999,1,30.5889861401528,0,0
2.87499999999999,2,30.7872534289627,0,0
2.89999999999999,1,30.9855207177726,0,0
2.92499999999999,1,31.1497707177726,0,0
2.94999999999999,1,31.3140207177726,0,0
2.97499999999999,1,31.4782707177726,0,0
2.99999999999999,1,31.6425207177726,0,0
3.02499999999999,1,31.8067707177726,0,0
3.04999999999999,1,31.9710207177726,0,0
3.07499999999999,1,32.1352707177726,0,0
3.09999999999999,1,32.2995207177726,0,0
3.12499999999999,1,32.4637707177726,0,0
3.14999999999999,1,32.6280207177726,0,0
3.17499999999999,1,32.7922707177726,0,0
3.19999999999999,1,32.9565207177726,0,0
3.22499999999999,1,33.1207707177726,0,0
3.24999999999999,1,33.2850207177726,0,0
3.27499999999999,1,33.4492707177726,0,0
3.29999999999999,1,33.6135207177726,0,0
3.32499999999999,1,33.7777707177726,0,0
3.34999999999999,0,33.8598957177726,0,0
3.37499999999999,1,33.9420207177726,0,0
3.39999999999999,1,34.1062707177726,0,0
3.42499999999999,1,34.2705207177726,0,0
3.44999999999999,1,34.4347707177726,0,0
3.47499999999999,1,34.5990207177726,0,0
3.49999999999999,1,34.7632707177726,0,0
3.52499999999999,1,34.9275207177726,0,0
3.54999999999999,1,35.0917707177726,0,0
3.57499999999999,1,35.2560207177726,0,0
3.59999999999999,1,35.4202707177726,0,0
3.62499999999999,1,35.5845207177726,0,0
3.64999999999999,1,35.7487707177726,0,0
3.67499999999999,1,35.9130207177726,0,0
3.69999999999999,1,36.0772707177726,0,0
3.72499999999999,1,36.2415207177726,0,0
3.74999999999999,1,36.4057707177726,0,0
3.77499999999999,1,36.5700207177726,0,0
3.79999999999999,1,36.7342707177726,0,0
3.82499999999999,0,36.8163957177726,0,0

3.84999999999999,0,36.8163957177726,0,0
3.87499999999999,4,36.9806457177726,0,0
3.89999999999999,2,37.2610380065825,0,0
3.92499999999999,1,37.4593052953924,0,0
3.94999999999999,2,37.6575725842023,0,0
3.97499999999999,0,37.7737148730122,0,0
3.99999999999999,2,37.8898571618221,0,0
4.02499999999999,1,38.088124450632,0,0
4.04999999999999,6,38.371413795758,0,0
4.07499999999999,2,38.688720429694,0,0
4.09999999999999,0,38.8048627185039,0,0
4.12499999999999,0,38.8048627185039,0,0
4.14999999999999,6,39.00602706363,0,0
4.17499999999999,3,39.3494360813276,0,0
4.19999999999999,0,39.4916807538992,0,0
4.22499999999999,1,39.5738057538992,0,0
4.24999999999999,1,39.7380557538992,0,0
4.27499999999999,0,39.8201807538992,0,0
4.29999999999999,1,39.9023057538992,0,0
4.32499999999999,1,40.0665557538992,0,0
4.34999999999999,1,40.2308057538992,0,0
4.37499999999999,1,40.3950557538992,0,0
4.4,1,40.5593057538992,0,0
4.425,1,40.7235557538992,0,0
4.45,1,40.8878057538992,0,0
4.475,1,41.0520557538992,0,0
4.5,1,41.2163057538992,0,0
4.525,1,41.3805557538992,0,0
4.55,1,41.5448057538992,0,0
4.575,1,41.7090557538992,0,0
4.6,1,41.8733057538992,0,0
4.625,1,42.0375557538992,0,0
4.65,1,42.2018057538992,0,0
4.675,1,42.3660557538992,0,0
4.7,1,42.5303057538992,0,0
4.725,1,42.6945557538992,0,0
4.75,0,42.7766807538992,0,0
4.775,1,42.8588057538992,0,0
4.8,1,43.0230557538992,0,0
4.825,1,43.1873057538992,0,0
4.85,1,43.3515557538992,0,0
4.875,1,43.5158057538993,0,0
4.9,1,43.6800557538993,0,0
4.925,1,43.8443057538993,0,0
4.95,1,44.0085557538993,0,0
4.975,1,44.1728057538993,0,0
5,1,44.3370557538993,0,0
5.025,0,44.4191807538993,0,0
5.05,0,44.4191807538993,0,0
5.075,0,44.4191807538993,0,0
5.10000000000001,1,44.5013057538993,0,0
5.12500000000001,1,44.6655557538993,0,0

5.15000000000001,1,44.8298057538993,0,0
5.17500000000001,1,44.9940557538993,0,0
5.20000000000001,1,45.1583057538993,0,0
5.22500000000001,1,45.3225557538993,0,0
5.25000000000001,1,45.4868057538993,0,0
5.27500000000001,1,45.6510557538993,0,0
5.30000000000001,1,45.8153057538993,0,0
5.32500000000001,1,45.9795557538993,0,0
5.35000000000001,1,46.1438057538993,0,0
5.37500000000001,1,46.3080557538993,0,0
5.40000000000001,1,46.4723057538993,0,0
5.42500000000001,1,46.6365557538993,0,0
5.45000000000001,1,46.8008057538993,0,0
5.47500000000001,1,46.9650557538993,0,0
5.50000000000001,1,47.1293057538993,0,0
5.52500000000001,1,47.2935557538993,0,0
5.55000000000001,1,47.4578057538993,0,0
5.57500000000001,1,47.6220557538993,0,0
5.60000000000001,1,47.7863057538993,0,0
5.62500000000001,1,47.9505557538993,0,0
5.65000000000001,1,48.1148057538993,0,0
5.67500000000001,0,48.1969307538993,0,0
5.70000000000001,1,48.2790557538993,0,0
5.72500000000001,1,48.4433057538993,0,0
5.75000000000001,1,48.6075557538993,0,0
5.77500000000001,1,48.7718057538993,0,0
5.80000000000001,1,48.9360557538993,0,0
5.82500000000002,1,49.1003057538993,0,0
5.85000000000002,1,49.2645557538993,0,0
5.87500000000002,1,49.4288057538993,0,0
5.90000000000002,0,49.5109307538993,0,0
5.92500000000002,1,49.5930557538993,0,0
5.95000000000002,1,49.7573057538993,0,0
5.97500000000002,0,49.8394307538993,0,0
6.00000000000002,0,49.8394307538993,0,0
6.02500000000002,1,49.9215557538993,0,0
6.05000000000002,1,50.0858057538993,0,0
6.07500000000002,1,50.2500557538993,0,0
6.10000000000002,1,50.4143057538993,0,0
6.12500000000002,1,50.5785557538993,0,0
6.15000000000002,0,50.6606807538993,0,0
6.17500000000002,1,50.7428057538993,0,0
6.20000000000002,0,50.8249307538993,0,0
6.22500000000002,7,51.0422130803205,0,0
6.25000000000002,0,51.2594954067417,0,0
6.27500000000002,0,51.2594954067417,0,0
6.30000000000002,0,51.2594954067417,0,0
6.32500000000002,2,51.3756376955516,0,0
6.35000000000002,0,51.4917799843615,0,0
6.37500000000002,7,51.7090623107826,0,0
6.40000000000002,2,52.0424869260137,0,0
6.42500000000002,0,52.1586292148236,0,0

6.45000000000002,0,52.1586292148236,0,0
6.47500000000002,1,52.2407542148236,0,0
6.50000000000002,1,52.4050042148236,0,0
6.52500000000003,1,52.5692542148236,0,0
6.55000000000003,1,52.7335042148236,0,0
6.57500000000003,1,52.8977542148236,0,0
6.60000000000003,1,53.0620042148236,0,0
6.62500000000003,2,53.2602715036335,0,0
6.65000000000003,1,53.4585387924434,0,0
6.67500000000003,1,53.6227887924434,0,0
6.70000000000003,1,53.7870387924434,0,0
6.72500000000003,1,53.9512887924434,0,0
6.75000000000003,1,54.1155387924434,0,0
6.77500000000003,1,54.2797887924434,0,0
6.80000000000003,0,54.3619137924434,0,0
6.82500000000003,1,54.4440387924434,0,0
6.85000000000003,1,54.6082887924434,0,0
6.87500000000003,1,54.7725387924434,0,0
6.90000000000003,1,54.9367887924434,0,0
6.92500000000003,1,55.1010387924434,0,0
6.95000000000003,1,55.2652887924434,0,0
6.97500000000003,1,55.4295387924434,0,0
7.00000000000003,10,55.7713658452847,25.2891249092172,1
7.02500000000003,12,56.3155572432693,25.2891249092172,1
7.05000000000003,15,56.9181153457197,25.2891249092172,1
7.07500000000003,15,57.5542528603343,25.2891249092172,1
7.10000000000003,14,58.1796052305304,25.2891249092172,1
7.12500000000003,14,58.794172456308,25.2891249092172,1
7.15000000000003,16,59.4299560691968,25.2891249092172,1
7.17500000000003,16,60.0869560691968,25.2891249092172,1
7.20000000000003,16,60.7439560691968,25.2891249092172,1
7.22500000000004,17,61.4110661187007,25.2891249092172,1
7.25000000000004,16,62.0781761682045,25.2891249092172,1
7.27500000000004,16,62.7351761682045,25.2891249092172,1
7.30000000000004,18,63.4121030346342,25.2891249092172,1
7.32500000000004,17,64.0991399505677,25.2891249092172,1
7.35000000000004,16,64.7662500000716,25.2891249092172,1
7.37500000000004,17,65.4333600495754,25.2891249092172,1
7.40000000000004,17,66.1105801485831,25.2891249092172,1
7.42500000000004,17,66.7878002475908,25.2891249092172,1
7.45000000000004,16,67.4549102970947,25.2891249092172,1
7.47500000000004,17,68.1220203465985,25.2891249092172,1
7.50000000000004,16,68.7891303961024,25.2891249092172,1
7.52500000000004,16,69.4461303961023,25.2891249092172,1
7.55000000000004,16,70.1031303961023,25.2891249092172,1
7.57500000000004,11,70.7040082070103,25.2891249092172,1
7.60000000000004,12,71.2608753630614,25.2891249092172,1
7.62500000000004,15,71.8634334655119,25.2891249092172,1
7.65000000000004,13,72.4776081213166,25.2891249092172,1
7.67500000000004,14,73.0809976327029,25.2891249092172,1
7.70000000000004,15,73.706350002899,25.2891249092172,1
7.72500000000004,12,74.3089081053495,25.2891249092172,1

7.75000000000004,11,74.8657752614006,25.2891249092172,1
7.77500000000004,8,75.3704376499283,25.2891249092172,1
7.80000000000004,5,75.7863593102003,0,0
7.82500000000004,8,76.2022809704722,0,0
7.85000000000004,6,76.6357298932181,0,0
7.87500000000004,9,77.0832692383442,0,0
7.90000000000004,6,77.5308085834702,0,0
7.92500000000005,0,77.7319729285963,0,0
7.95000000000005,6,77.9331372737224,0,0
7.97500000000005,6,78.3354659639745,0,0
8.00000000000005,5,78.7202673917528,0,0
8.02500000000005,5,79.0875415570571,0,0
8.05000000000005,3,79.4134233122809,0,0
8.07500000000005,3,79.6979126574241,0,0
8.10000000000005,2,79.9562996188055,0,0
8.12500000000005,2,80.1885841964253,0,0
8.15000000000005,2,80.4208687740451,0,0
8.17500000000005,1,80.619136062855,0,0
8.20000000000005,1,80.783386062855,0,0
8.22500000000005,1,80.947636062855,0,0
8.25000000000005,1,81.111886062855,0,0
8.27500000000005,2,81.3101533516649,0,0
8.30000000000005,2,81.5424379292846,0,0
8.32500000000005,2,81.7747225069044,0,0
8.35000000000005,3,82.0331094682859,0,0
8.37500000000005,3,82.3175988134291,0,0
8.40000000000005,3,82.6020881585723,0,0
8.42500000000005,3,82.8865775037155,0,0
8.45000000000005,4,83.1930721762871,0,0
8.47500000000005,4,83.5215721762871,0,0
8.50000000000005,2,83.801964465097,0,0
8.52500000000005,4,84.0823567539069,0,0
8.55000000000005,4,84.4108567539069,0,0
8.57500000000005,5,84.758743836559,0,0
8.60000000000005,4,85.1066309192112,0,0
8.62500000000006,4,85.4351309192112,0,0
8.65000000000006,4,85.7636309192112,0,0
8.67500000000006,4,86.0921309192112,0,0
8.70000000000006,4,86.4206309192112,0,0
8.72500000000006,4,86.7491309192112,0,0
8.75000000000006,3,87.0556255917828,0,0
8.77500000000006,3,87.340114936926,0,0
8.80000000000006,4,87.6466096094976,0,0
8.82500000000006,7,88.0281419359188,0,0
8.85000000000006,8,88.4777088399597,0,0
8.87500000000006,8,88.9422779951993,0,0
8.90000000000006,8,89.4068471504388,0,0
8.92500000000006,8,89.8714163056784,0,0
8.95000000000006,8,90.335985460918,0,0
8.97500000000006,6,90.7694343836638,0,0
9.00000000000006,7,91.1878810552111,0,0
9.02500000000006,6,91.6063277267583,0,0

9.05000000000006,6,92.0086564170104,0,0
9.07500000000006,5,92.3934578447887,0,0
9.10000000000006,5,92.760732010093,0,0
9.12500000000006,5,93.1280061753973,0,0
9.15000000000006,5,93.4952803407017,0,0
9.17500000000006,5,93.862554506006,0,0
9.20000000000006,4,94.2104415886582,0,0
9.22500000000006,4,94.5389415886582,0,0
9.25000000000006,4,94.8674415886582,0,0
9.27500000000006,4,95.1959415886582,0,0
9.30000000000006,4,95.5244415886582,0,0
9.32500000000007,4,95.8529415886582,0,0
9.35000000000007,4,96.1814415886582,0,0
9.37500000000007,4,96.5099415886582,0,0
9.40000000000007,4,96.8384415886582,0,0
9.42500000000007,4,97.1669415886582,0,0
9.45000000000007,3,97.4734362612298,0,0
9.47500000000007,3,97.757925606373,0,0
9.50000000000007,3,98.0424149515162,0,0
9.52500000000007,3,98.3269042966594,0,0
9.55000000000007,3,98.6113936418026,0,0
9.57500000000007,3,98.8958829869458,0,0
9.60000000000007,3,99.1803723320889,0,0
9.62500000000007,3,99.4648616772321,0,0
9.65000000000007,3,99.7493510223753,0,0
9.67500000000007,3,100.033840367518,0,0
9.70000000000007,3,100.318329712662,0,0
9.72500000000007,2,100.576716674043,0,0
9.75000000000007,3,100.835103635425,0,0
9.77500000000007,2,101.093490596806,0,0
9.80000000000007,2,101.325775174426,0,0
9.82500000000007,2,101.558059752046,0,0
9.85000000000007,1,101.756327040856,0,0
9.87500000000007,1,101.920577040856,0,0
9.90000000000007,1,102.084827040856,0,0
9.92500000000007,1,102.249077040856,0,0
9.95000000000007,1,102.413327040856,0,0
9.97500000000007,1,102.577577040856,0,0
10.0000000000001,2,102.775844329665,0,0
10.0250000000001,2,103.008128907285,0,0
10.0500000000001,2,103.240413484905,0,0
10.0750000000001,2,103.472698062525,0,0
10.1000000000001,2,103.704982640145,0,0
10.1250000000001,1,103.903249928954,0,0
10.1500000000001,1,104.067499928954,0,0
10.1750000000001,1,104.231749928954,0,0
10.2000000000001,0,104.313874928954,0,0
10.2250000000001,0,104.313874928954,0,0
10.2500000000001,1,104.395999928954,0,0
10.2750000000001,0,104.478124928954,0,0
10.3000000000001,1,104.560249928954,0,0
10.3250000000001,1,104.724499928954,0,0

10.3500000000001,0,104.806624928954,0,0
10.3750000000001,0,104.806624928954,0,0
10.4000000000001,1,104.888749928954,0,0
10.4250000000001,0,104.970874928954,0,0
10.4500000000001,1,105.052999928954,0,0
10.4750000000001,0,105.135124928955,0,0
10.5000000000001,0,105.135124928955,0,0
10.5250000000001,0,105.135124928955,0,0
10.5500000000001,0,105.135124928955,0,0
10.5750000000001,0,105.135124928955,0,0
10.6000000000001,0,105.135124928955,0,0
10.6250000000001,0,105.135124928955,0,0
10.6500000000001,0,105.135124928955,0,0
10.6750000000001,0,105.135124928955,0,0
10.7000000000001,0,105.135124928955,0,0
10.7250000000001,1,105.217249928955,0,0
10.7500000000001,0,105.299374928955,0,0
10.7750000000001,0,105.299374928955,0,0
10.8000000000001,1,105.381499928955,0,0
10.8250000000001,0,105.463624928955,0,0
10.8500000000001,0,105.463624928955,0,0
10.8750000000001,0,105.463624928955,0,0
10.9000000000001,1,105.545749928955,0,0
10.9250000000001,0,105.627874928955,0,0
10.9500000000001,1,105.709999928955,0,0
10.9750000000001,1,105.874249928955,0,0
11.0000000000001,1,106.038499928955,0,0
11.0250000000001,1,106.202749928955,0,0
11.0500000000001,1,106.366999928955,0,0
11.0750000000001,1,106.531249928955,0,0
11.1000000000001,1,106.695499928955,0,0
11.1250000000001,1,106.859749928955,0,0
11.1500000000001,1,107.023999928955,0,0
11.1750000000001,1,107.188249928954,0,0
11.2000000000001,1,107.352499928954,0,0
11.2250000000001,1,107.516749928954,0,0
11.2500000000001,1,107.680999928954,0,0
11.2750000000001,1,107.845249928954,0,0
11.3000000000001,1,108.009499928954,0,0
11.3250000000001,2,108.207767217764,0,0
11.3500000000001,0,108.323909506574,0,0
11.3750000000001,4,108.488159506574,0,0
11.4000000000001,1,108.734534506574,0,0
11.4250000000001,0,108.816659506574,0,0
11.4500000000001,0,108.816659506574,0,0
11.4750000000001,4,108.980909506574,0,0
11.5000000000001,0,109.145159506574,0,0
11.5250000000001,1,109.227284506574,0,0
11.5500000000001,6,109.5105738517,0,0
11.5750000000001,0,109.711738196826,0,0
11.6000000000001,0,109.711738196826,0,0
11.6250000000001,0,109.711738196826,0,0

11.6500000000001,0,109.711738196826,0,0
11.6750000000001,1,109.793863196826,0,0
11.7000000000001,0,109.875988196826,0,0
11.7250000000001,2,109.992130485636,0,0
11.7500000000001,0,110.108272774446,0,0
11.7750000000001,0,110.108272774446,0,0
11.8000000000001,0,110.108272774446,0,0
11.8250000000001,0,110.108272774446,0,0
11.8500000000001,0,110.108272774446,0,0
11.8750000000001,0,110.108272774446,0,0
11.9000000000001,0,110.108272774446,0,0
11.9250000000001,0,110.108272774446,0,0
11.9500000000001,0,110.108272774446,0,0
11.9750000000001,0,110.108272774446,0,0
12.0000000000001,1,110.190397774446,0,0
12.0250000000001,1,110.354647774446,0,0
12.0500000000001,1,110.518897774446,0,0
12.0750000000001,0,110.601022774446,0,0
12.1000000000001,0,110.601022774446,0,0
12.1250000000001,1,110.683147774446,0,0
12.1500000000001,1,110.847397774446,0,0
12.1750000000001,1,111.011647774446,0,0
12.2000000000001,1,111.175897774446,0,0
12.2250000000001,0,111.258022774446,0,0
12.2500000000001,0,111.258022774446,0,0
12.2750000000001,1,111.340147774446,0,0
12.3000000000001,1,111.504397774446,0,0
12.3250000000001,1,111.668647774446,0,0
12.3500000000001,1,111.832897774446,0,0
12.3750000000001,1,111.997147774446,0,0
12.4000000000001,1,112.161397774446,0,0
12.4250000000001,1,112.325647774446,0,0
12.4500000000001,1,112.489897774446,0,0
12.4750000000001,1,112.654147774446,0,0
12.5000000000001,1,112.818397774446,0,0
12.5250000000001,1,112.982647774446,0,0
12.5500000000001,1,113.146897774446,0,0
12.5750000000001,1,113.311147774446,0,0
12.6000000000001,1,113.475397774446,0,0
12.6250000000001,1,113.639647774446,0,0
12.6500000000001,1,113.803897774446,0,0
12.6750000000001,1,113.968147774446,0,0
12.7000000000001,1,114.132397774446,0,0
12.7250000000001,1,114.296647774446,0,0
12.7500000000001,1,114.460897774446,0,0
12.7750000000001,1,114.625147774446,0,0
12.8000000000001,1,114.789397774446,0,0
12.8250000000001,1,114.953647774446,0,0
12.8500000000001,7,115.253055100867,0,0
12.8750000000001,7,115.68761975371,0,0
12.9000000000001,7,116.122184406552,0,0
12.9250000000001,7,116.556749059394,0,0

12.9500000000001,6,116.975195730942,0,0
12.9750000000001,6,117.377524421194,0,0
13.0000000000001,6,117.779853111446,0,0
13.0250000000001,5,118.164654539224,0,0
13.0500000000001,5,118.531928704528,0,0
13.0750000000001,4,118.879815787181,0,0
13.1000000000001,4,119.208315787181,0,0
13.1250000000001,4,119.536815787181,0,0
13.1500000000001,4,119.865315787181,0,0
13.1750000000001,4,120.193815787181,0,0
13.2000000000001,6,120.559230132307,0,0
13.2250000000001,6,120.961558822559,0,0
13.2500000000001,5,121.346360250337,0,0
13.2750000000001,4,121.694247332989,0,0
13.3000000000001,4,122.022747332989,0,0
13.3250000000001,3,122.329242005561,0,0
13.3500000000001,2,122.587628966942,0,0
13.3750000000001,2,122.819913544562,0,0
13.4000000000001,2,123.052198122182,0,0
13.4250000000001,3,123.310585083563,0,0
13.4500000000001,4,123.617079756135,0,0
13.4750000000001,4,123.945579756135,0,0
13.5000000000001,4,124.274079756135,0,0
13.5250000000001,3,124.580574428707,0,0
13.5500000000001,3,124.86506377385,0,0
13.5750000000001,3,125.149553118993,0,0
13.6000000000001,3,125.434042464136,0,0
13.6250000000001,3,125.718531809279,0,0
13.6500000000001,3,126.003021154422,0,0
13.6750000000001,4,126.309515826994,0,0
13.7000000000001,8,126.706050404614,0,0
13.7250000000001,10,127.198037035075,0,1
13.7500000000001,11,127.730116898824,0,1
13.7750000000001,11,128.27487252064,0,1
13.8000000000001,9,128.793625331548,0,0
13.8250000000001,8,129.272284909168,0,0
13.8500000000001,7,129.721851813209,0,0
13.8750000000001,6,130.140298484756,0,0
13.9000000000001,5,130.525099912534,0,0
13.9250000000001,5,130.892374077839,0,0
13.9500000000001,5,131.259648243143,0,0
13.9750000000001,5,131.626922408447,0,0
14.0000000000001,5,131.994196573752,0,0
14.0250000000001,5,132.361470739056,0,0
14.0500000000001,4,132.709357821708,0,0
14.0750000000001,4,133.037857821708,0,0
14.1000000000001,4,133.366357821708,0,0
14.1250000000001,3,133.67285249428,0,0
14.1500000000001,3,133.957341839423,0,0
14.1750000000001,2,134.215728800804,0,0
14.2000000000001,2,134.448013378424,0,0
14.2250000000001,1,134.646280667234,0,0

14.2500000000001,1,134.810530667234,0,0
14.2750000000001,1,134.974780667234,0,0
14.3000000000001,1,135.139030667234,0,0
14.3250000000001,1,135.303280667234,0,0
14.3500000000001,2,135.501547956044,0,0
14.3750000000001,2,135.733832533664,0,0
14.4000000000001,2,135.966117111284,0,0
14.4250000000001,2,136.198401688903,0,0
14.4500000000001,2,136.430686266523,0,0
14.4750000000001,2,136.662970844143,0,0
14.5000000000001,2,136.895255421763,0,0
14.5250000000001,2,137.127539999383,0,0
14.5500000000001,2,137.359824577002,0,0
14.5750000000001,2,137.592109154622,0,0
14.6000000000001,2,137.824393732242,0,0
14.6250000000001,3,138.082780693623,0,0
14.6500000000001,3,138.367270038767,0,0
14.6750000000001,3,138.65175938391,0,0
14.7000000000001,2,138.910146345291,0,0
14.7250000000001,2,139.142430922911,0,0
14.7500000000001,1,139.340698211721,0,0
14.7750000000001,1,139.504948211721,0,0
14.8000000000001,1,139.669198211721,0,0
14.8250000000001,1,139.833448211721,0,0
14.8500000000001,1,139.997698211721,0,0
14.8750000000001,1,140.161948211721,0,0
14.9000000000001,1,140.326198211721,0,0
14.9250000000001,1,140.490448211721,0,0
14.9500000000001,2,140.688715500531,0,0
14.9750000000001,2,140.921000078151,0,0
15.0000000000001,2,141.15328465577,0,0
15.0250000000001,1,141.35155194458,0,0
15.0500000000001,1,141.51580194458,0,0
15.0750000000001,4,141.76217694458,0,0
15.1000000000001,0,141.92642694458,0,0
15.1250000000001,0,141.92642694458,0,0
15.1500000000001,6,142.127591289706,0,0
15.1750000000001,0,142.328755634833,0,0
15.2000000000001,0,142.328755634833,0,0
15.2250000000001,0,142.328755634833,0,0
15.2500000000001,0,142.328755634833,0,0
15.2750000000002,3,142.471000307404,0,0
15.3000000000002,1,142.695369979976,0,0
15.3250000000002,1,142.859619979976,0,0
15.3500000000002,2,143.057887268786,0,0
15.3750000000002,1,143.256154557595,0,0
15.4000000000002,0,143.338279557595,0,0
15.4250000000002,2,143.454421846405,0,0
15.4500000000002,4,143.734814135215,0,0
15.4750000000002,6,144.100228480341,0,0
15.5000000000002,8,144.533677403087,0,0
15.5250000000002,10,145.025664033548,0,1

15.5500000000002,10,145.545068139231,0,1
15.5750000000002,11,146.07714800298,0,1
15.6000000000002,11,146.621903624796,0,1
15.6250000000002,11,147.166659246612,0,1
15.6500000000002,11,147.711414868428,0,1
15.6750000000002,10,148.243494732177,0,1
15.7000000000002,8,148.735481362638,0,0
15.7250000000002,7,149.185048266679,0,0
15.7500000000002,7,149.619612919522,0,0
15.7750000000002,6,150.038059591069,0,0
15.8000000000002,6,150.440388281321,0,0
15.8250000000002,6,150.842716971573,0,0
15.8500000000002,5,151.227518399351,0,0
15.8750000000002,5,151.594792564656,0,0
15.9000000000002,5,151.96206672996,0,0
15.9250000000002,5,152.329340895264,0,0
15.9500000000002,6,152.714142323043,0,0
15.9750000000002,7,153.13258899459,0,0
16.0000000000002,7,153.567153647432,0,0
16.0250000000002,10,154.044138026695,25.5564427283659,1
16.0500000000002,12,154.588329424679,25.5564427283659,1
16.0750000000002,13,155.16892466832,25.5564427283659,1
16.1000000000002,16,155.793530566817,25.5564427283659,1
16.1250000000002,17,156.460640616321,25.5564427283659,1
16.1500000000002,17,157.137860715329,25.5564427283659,1
16.1750000000002,18,157.824897631262,25.5564427283659,1
16.2000000000001,18,158.521751364122,25.5564427283659,1
16.2250000000001,18,159.218605096981,25.5564427283659,1
16.2500000000001,17,159.905642012915,25.5564427283659,1
16.2750000000001,17,160.582862111922,25.5564427283659,1
16.3000000000001,18,161.269899027856,25.5564427283659,1
16.3250000000001,18,161.966752760715,25.5564427283659,1
16.3500000000001,18,162.663606493575,25.5564427283659,1
16.3750000000001,17,163.350643409508,25.5564427283659,1
16.4000000000001,16,164.017753459012,25.5564427283659,1
16.4250000000001,15,164.664322216319,25.5564427283659,1
16.4500000000001,14,165.289674586515,25.5564427283659,1
16.4750000000001,13,165.893064097902,25.5564427283659,1
16.5000000000001,14,166.496453609288,25.5564427283659,1
16.5250000000001,11,167.076115033085,25.5564427283659,1
16.5500000000001,10,167.608194896834,25.5564427283659,1
16.5750000000001,8,168.100181527295,25.5564427283659,1
16.6000000000001,7,168.549748431336,0,0
16.6250000000001,6,168.968195102883,0,0
16.6500000000001,6,169.370523793135,0,0
16.6750000000001,6,169.772852483388,0,0
16.7000000000001,6,170.17518117364,0,0
16.7250000000001,6,170.577509863892,0,0
16.7500000000001,7,170.995956535439,0,0
16.7750000000001,7,171.430521188282,0,0
16.8000000000001,7,171.865085841124,0,0
16.8250000000001,7,172.29965049396,0,0

16.8500000000001,6,172.718097165514,0,0
16.8750000000001,6,173.120425855766,0,0
16.9000000000001,5,173.505227283544,0,0
16.9250000000001,5,173.872501448848,0,0
16.9500000000001,4,174.2203885315,0,0
16.9750000000001,5,174.568275614153,0,0
17.0000000000001,4,174.916162696805,0,0
17.0250000000001,4,175.244662696805,0,0
17.0500000000001,4,175.573162696805,0,0
17.0750000000001,5,175.921049779457,0,0
17.1000000000001,5,176.288323944761,0,0
17.1250000000001,6,176.673125372539,0,0
17.1500000000001,5,177.057926800318,0,0
17.1750000000001,4,177.40581388297,0,0
17.2000000000001,3,177.712308555541,0,0
17.2250000000001,2,177.970695516923,0,0
17.2500000000001,2,178.202980094543,0,0
17.2750000000001,2,178.435264672163,0,0
17.3000000000001,2,178.667549249782,0,0
17.3250000000001,2,178.899833827402,0,0
17.3500000000001,2,179.132118405022,0,0
17.3750000000001,2,179.364402982642,0,0
17.4000000000001,2,179.596687560261,0,0
17.4250000000001,1,179.794954849071,0,0
17.4500000000001,1,179.959204849071,0,0
17.4750000000001,1,180.123454849071,0,0
17.5000000000001,1,180.287704849071,0,0
17.5250000000001,1,180.451954849071,0,0
17.5500000000001,1,180.616204849071,0,0
17.5750000000001,1,180.780454849071,0,0
17.6000000000001,1,180.944704849071,0,0
17.6250000000001,0,181.026829849071,0,0
17.6500000000001,0,181.026829849071,0,0
17.6750000000001,1,181.108954849071,0,0
17.7000000000001,0,181.191079849071,0,0
17.7250000000001,1,181.273204849071,0,0
17.7500000000001,1,181.437454849071,0,0
17.7750000000001,1,181.601704849071,0,0
17.8000000000001,1,181.765954849071,0,0
17.8250000000001,1,181.930204849071,0,0
17.8500000000001,1,182.094454849071,0,0
17.8750000000001,0,182.176579849071,0,0
17.9000000000001,1,182.258704849071,0,0
17.9250000000001,1,182.422954849071,0,0
17.95,1,182.587204849071,0,0
17.975,1,182.751454849071,0,0
18,1,182.915704849071,0,0
18.025,1,183.079954849071,0,0
18.05,1,183.244204849071,0,0
18.075,1,183.408454849071,0,0
18.1,1,183.572704849072,0,0
18.125,1,183.736954849072,0,0

18.15,1,183.901204849072,0,0
18.175,1,184.065454849072,0,0
18.2,1,184.229704849072,0,0
18.225,1,184.393954849072,0,0
18.25,1,184.558204849072,0,0
18.275,1,184.722454849072,0,0
18.3,1,184.886704849072,0,0
18.325,1,185.050954849072,0,0
18.35,2,185.249222137881,0,0
18.375,1,185.447489426691,0,0
18.4,1,185.611739426691,0,0
18.425,1,185.775989426691,0,0
18.45,1,185.940239426691,0,0
18.475,1,186.104489426691,0,0
18.5,1,186.268739426691,0,0
18.525,1,186.432989426691,0,0
18.55,1,186.597239426691,0,0
18.575,1,186.761489426691,0,0
18.6,1,186.925739426691,0,0
18.625,1,187.089989426691,0,0
18.65,1,187.254239426691,0,0
18.675,1,187.418489426691,0,0
18.7,1,187.582739426691,0,0
18.725,1,187.746989426692,0,0
18.75,1,187.911239426692,0,0
18.775,1,188.075489426692,0,0
18.8,0,188.157614426692,0,0
18.825,4,188.321864426692,0,0
18.85,1,188.568239426692,0,0
18.875,1,188.732489426692,0,0
18.9,0,188.814614426692,0,0
18.925,0,188.814614426692,0,0
18.95,6,189.015778771818,0,0
18.975,1,189.299068116944,0,0
19,0,189.381193116944,0,0
19.025,1,189.463318116944,0,0
19.05,0,189.545443116944,0,0
19.075,0,189.545443116944,0,0
19.1,0,189.545443116944,0,0
19.125,4,189.709693116944,0,0
19.15,1,189.956068116944,0,0
19.175,1,190.120318116944,0,0
19.2,8,190.434727694563,0,0
19.225,1,190.749137272183,0,0
19.25,1,190.913387272183,0,0
19.275,1,191.077637272183,0,0
19.3,1,191.241887272183,0,0
19.325,1,191.406137272183,0,0
19.35,1,191.570387272183,0,0
19.375,1,191.734637272183,0,0
19.4,1,191.898887272183,0,0
19.425,1,192.063137272183,0,0

```
19.45,1,192.227387272183,0,0
19.475,1,192.391637272183,0,0
19.5,1,192.555887272183,0,0
19.525,1,192.720137272183,0,0
19.55,1,192.884387272183,0,0
19.575,1,193.048637272183,0,0
19.6,1,193.212887272183,0,0
19.625,1,193.377137272183,0,0
19.65,1,193.541387272183,0,0
19.675,1,193.705637272183,0,0
19.7,1,193.869887272183,0,0
19.7249999999999,1,194.034137272183,0,0
19.7499999999999,1,194.198387272183,0,0
19.7749999999999,1,194.362637272183,0,0
19.7999999999999,1,194.526887272183,0,0
19.8249999999999,1,194.691137272183,0,0
19.8499999999999,1,194.855387272183,0,0
19.8749999999999,1,195.019637272183,0,0
19.8999999999999,1,195.183887272183,0,0
19.9249999999999,1,195.348137272183,0,0
19.9499999999999,1,195.512387272184,0,0
19.9749999999999,1,195.676637272184,0,0
19.9999999999999,1,195.840887272184,0,0
20.0249999999999,1,196.005137272184,0,0
20.0499999999999,1,196.169387272184,0,0
20.0749999999999,1,196.333637272184,0,0
20.0999999999999,1,196.497887272184,0,0
20.1249999999999,1,196.662137272184,0,0
20.1499999999999,0,196.744262272184,0,0
20.1749999999999,1,196.826387272184,0,0
20.1999999999999,1,196.990637272184,0,0
20.2249999999999,1,197.154887272184,0,0
20.2499999999999,1,197.319137272184,0,0
20.2749999999999,1,197.483387272184,0,0
20.2999999999999,1,197.647637272184,0,0
20.3249999999999,1,197.811887272184,0,0
20.3499999999999,1,197.976137272184,0,0
20.3749999999999,1,198.140387272184,0,0
20.3999999999999,1,198.304637272184,0,0
20.4249999999999,4,198.551012272184,0,0
20.4499999999999,4,198.879512272184,0,0
20.4749999999999,2,199.159904560994,0,0
20.4999999999999,3,199.418291522375,0,0
20.5249999999999,1,199.642661194947,0,0
20.5499999999999,1,199.806911194947,0,0
20.5749999999999,5,200.072673277599,0,0
20.5999999999999,6,200.457474705377,0,0
20.6249999999999,8,200.890923628123,0,0
20.6499999999999,9,201.369583205743,0,0
20.6749999999999,6,201.817122550869,0,0
20.6999999999999,7,202.235569222416,0,0
20.7249999999999,7,202.670133875258,0,0
```

20.7499999999999,7,203.104698528101,0,0
20.7749999999999,7,203.539263180943,0,0
20.7999999999999,7,203.973827833785,0,0
20.8249999999999,6,204.392274505333,0,0
20.8499999999999,7,204.81072117688,0,0
20.8749999999999,8,205.260288080921,0,0
20.8999999999999,8,205.72485723616,0,0
20.9249999999999,8,206.1894263914,0,0
20.9499999999999,8,206.65399554664,0,0
20.9749999999999,8,207.118564701879,0,0
20.9999999999999,8,207.583133857119,0,0
21.0249999999999,9,208.061793434738,0,0
21.0499999999999,13,208.604274333236,25.6824007994316,1
21.0749999999999,20,209.267654397038,25.6824007994316,1
21.0999999999999,21,210.01127259129,25.6824007994316,1
21.1249999999999,21,210.763960649187,25.6824007994316,1
21.1499999999999,18,211.488731544565,25.6824007994316,1
21.1749999999999,16,212.165658410994,25.6824007994316,1
21.1999999999999,13,212.790264309492,25.6824007994316,1
21.2249999999999,11,213.358748018897,25.6824007994316,1
21.2499999999999,10,213.890827882647,25.6824007994316,1
21.2749999999999,8,214.382814513108,25.6824007994316,1
21.2999999999999,7,214.832381417149,0,0
21.3249999999999,7,215.266946069991,0,0
21.3499999999999,7,215.701510722833,0,0
21.3749999999999,5,216.102430131907,0,0
21.3999999999999,4,216.450317214559,0,0
21.4249999999999,4,216.778817214559,0,0
21.4499999999999,5,217.126704297211,0,0
21.4749999999998,5,217.493978462515,0,0
21.4999999999998,5,217.86125262782,0,0
21.5249999999998,5,218.228526793124,0,0
21.5499999999998,6,218.613328220902,0,0
21.5749999999998,8,219.046777143648,0,0
21.5999999999998,9,219.525436721268,0,0
21.6249999999998,9,220.018186721268,0,0
21.6499999999998,9,220.510936721268,0,0
21.6749999999998,7,220.974594047689,0,0
21.6999999999998,7,221.409158700532,0,0
21.7249999999998,6,221.827605372079,0,0
21.7499999999998,5,222.212406799857,0,0
21.7749999999998,4,222.560293882509,0,0
21.7999999999998,3,222.866788555081,0,0
21.8249999999998,2,223.125175516462,0,0
21.8499999999998,1,223.323442805272,0,0
21.8749999999998,1,223.487692805272,0,0
21.8999999999998,1,223.651942805272,0,0
21.9249999999998,1,223.816192805272,0,0
21.9499999999998,0,223.898317805272,0,0
21.9749999999998,0,223.898317805272,0,0
21.9999999999998,0,223.898317805272,0,0
22.0249999999998,0,223.898317805272,0,0

```
22.0499999999998,0,223.898317805272,0,0
22.0749999999998,0,223.898317805272,0,0
22.0999999999998,0,223.898317805272,0,0
22.1249999999998,0,223.898317805272,0,0
22.1499999999998,0,223.898317805272,0,0
22.1749999999998,0,223.898317805272,0,0
22.1999999999998,0,223.898317805272,0,0
22.2249999999998,0,223.898317805272,0,0
22.2499999999998,0,223.898317805272,0,0
22.2749999999998,0,223.898317805272,0,0
22.2999999999998,0,223.898317805272,0,0
22.3249999999998,0,223.898317805272,0,0
22.3499999999998,0,223.898317805272,0,0
22.3749999999998,0,223.898317805272,0,0
22.3999999999998,1,223.980442805272,0,0
22.4249999999998,1,224.144692805272,0,0
22.4499999999998,1,224.308942805272,0,0
22.4749999999998,1,224.473192805272,0,0
22.4999999999998,1,224.637442805272,0,0
22.5249999999998,1,224.801692805272,0,0
22.5499999999998,1,224.965942805272,0,0
22.5749999999998,0,225.048067805272,0,0
22.5999999999998,1,225.130192805272,0,0
22.6249999999998,1,225.294442805272,0,0
22.6499999999998,0,225.376567805272,0,0
22.6749999999998,0,225.376567805272,0,0
22.6999999999998,0,225.376567805272,0,0
22.7249999999998,0,225.376567805272,0,0
22.7499999999998,2,225.492710094082,0,0
22.7749999999998,0,225.608852382892,0,0
22.7999999999998,0,225.608852382892,0,0
22.8249999999998,1,225.690977382892,0,0
22.8499999999998,0,225.773102382892,0,0
22.8749999999998,0,225.773102382892,0,0
22.8999999999998,0,225.773102382892,0,0
22.9249999999998,0,225.773102382892,0,0
22.9499999999998,3,225.915347055464,0,0
22.9749999999998,1,226.139716728035,0,0
22.9999999999998,1,226.303966728035,0,0
23.0249999999998,0,226.386091728035,0,0
23.0499999999998,1,226.468216728035,0,0
23.0749999999998,1,226.632466728035,0,0
23.0999999999998,1,226.796716728035,0,0
23.1249999999998,1,226.960966728035,0,0
23.1499999999998,1,227.125216728035,0,0
23.1749999999998,9,227.453716728035,0,0
23.1999999999998,13,227.996197626533,24.4826308114472,1
23.2249999999997,14,228.599587137919,24.4826308114472,1
23.2499999999997,16,229.235370750808,24.4826308114472,1
23.2749999999997,17,229.902480800312,24.4826308114472,1
23.2999999999997,19,230.599065425554,24.4826308114472,1
23.3249999999997,18,231.305466867722,24.4826308114472,1
```

23.3499999999997,18,232.002320600581,24.4826308114472,1
23.3749999999997,18,232.69917433344,24.4826308114472,1
23.3999999999997,18,233.3960280663,24.4826308114472,1
23.4249999999997,18,234.092881799159,24.4826308114472,1
23.4499999999997,18,234.789735532018,24.4826308114472,1
23.4749999999997,19,235.496136974186,24.4826308114472,1
23.4999999999997,19,236.212086125663,24.4826308114472,1
23.5249999999997,19,236.92803527714,24.4826308114472,1
23.5499999999997,18,237.634436719308,24.4826308114472,1
23.5749999999997,17,238.321473635241,24.4826308114472,1
23.5999999999997,16,238.988583684745,24.4826308114472,1
23.6249999999997,15,239.635152442052,24.4826308114472,1
23.6499999999997,14,240.260504812248,24.4826308114472,1
23.6749999999997,13,240.863894323635,24.4826308114472,1
23.6999999999997,15,241.478068979439,24.4826308114472,1
23.7249999999997,14,242.103421349635,24.4826308114472,1
23.7499999999997,15,242.728773719832,24.4826308114472,1
23.7749999999997,15,243.364911234446,24.4826308114472,1
23.7999999999997,15,244.001048749061,24.4826308114472,1
23.8249999999997,17,244.657727555872,24.4826308114472,1
23.8499999999997,16,245.324837605376,24.4826308114472,1
23.8749999999997,17,245.99194765488,24.4826308114472,1
23.8999999999997,18,246.678984570813,24.4826308114472,1
23.9249999999997,17,247.366021486747,24.4826308114472,1
23.9499999999997,15,248.022700293558,24.4826308114472,1
23.9749999999997,13,248.636874949362,24.4826308114472,1
23.9999999999997,10,249.192682900701,24.4826308114472,1
24.0249999999997,9,249.698759953543,24.4826308114472,1
24.0499999999997,9,250.191509953543,24.4826308114472,1
24.0749999999997,9,250.684259953543,24.4826308114472,1
24.0999999999997,10,251.190337006384,24.4826308114472,1
24.1249999999997,11,251.722416870133,24.4826308114472,1
24.1499999999997,12,252.279284026184,24.4826308114472,1
24.1749999999997,14,252.871056984216,24.4826308114472,1
24.1999999999997,15,253.496409354412,24.4826308114472,1
24.2249999999997,14,254.121761724608,24.4826308114472,1
24.2499999999997,12,254.713534682641,24.4826308114472,1
24.2749999999997,11,255.270401838692,24.4826308114472,1
24.2999999999997,11,255.815157460507,24.4826308114472,1
24.3249999999997,10,256.347237324257,24.4826308114472,1
24.3499999999997,11,256.879317188006,24.4826308114472,1
24.3749999999997,11,257.424072809822,24.4826308114472,1
24.3999999999997,11,257.968828431638,24.4826308114472,1
24.4249999999997,11,258.513584053454,24.4826308114472,1
24.4499999999997,12,259.070451209505,24.4826308114472,1
24.4749999999997,12,259.639429899791,24.4826308114472,1
24.4999999999997,13,260.220025143432,24.4826308114472,1
24.5249999999997,13,260.812236940427,24.4826308114472,1
24.5499999999997,12,261.392832184067,24.4826308114472,1
24.5749999999997,12,261.961810874354,24.4826308114472,1
24.5999999999997,11,262.518678030405,24.4826308114472,1
24.6249999999997,12,263.075545186456,24.4826308114472,1

24.6499999999997,12,263.644523876742,24.4826308114472,1
24.6749999999997,11,264.201391032793,24.4826308114472,1
24.6999999999997,9,264.720143843701,24.4826308114472,1
24.7249999999997,7,265.183801170123,0,0
24.7499999999997,7,265.618365822965,0,0
24.7749999999997,6,266.036812494512,0,0
24.7999999999997,6,266.439141184764,0,0
24.8249999999997,6,266.841469875017,0,0
24.8499999999997,6,267.243798565269,0,0
24.8749999999997,8,267.677247488015,0,0
24.8999999999997,9,268.155907065634,0,0
24.9249999999997,7,268.619564392056,0,0
24.9499999999997,6,269.038011063603,0,0
24.9749999999997,6,269.440339753855,0,0
24.9999999999996,3,269.783748771553,0,0
25.0249999999996,2,270.042135732934,0,0
25.0499999999996,1,270.240403021744,0,0
25.0749999999996,1,270.404653021744,0,0
25.0999999999996,1,270.568903021744,0,0
25.1249999999996,1,270.733153021744,0,0
25.1499999999996,1,270.897403021744,0,0
25.1749999999996,2,271.095670310554,0,0
25.1999999999996,1,271.293937599364,0,0
25.2249999999996,0,271.376062599364,0,0
25.2499999999996,2,271.492204888174,0,0
25.2749999999996,1,271.690472176984,0,0
25.2999999999996,0,271.772597176984,0,0
25.3249999999996,0,271.772597176984,0,0
25.3499999999996,1,271.854722176984,0,0
25.3749999999996,4,272.101097176984,0,0
25.3999999999996,0,272.265347176984,0,0
25.4249999999996,0,272.265347176984,0,0
25.4499999999996,0,272.265347176984,0,0
25.4749999999996,1,272.347472176984,0,0
25.4999999999996,1,272.511722176984,0,0
25.5249999999996,1,272.675972176984,0,0
25.5499999999996,1,272.840222176984,0,0
25.5749999999996,0,272.922347176984,0,0
25.5999999999996,1,273.004472176984,0,0
25.6249999999996,1,273.168722176984,0,0
25.6499999999996,0,273.250847176984,0,0
25.6749999999996,1,273.332972176984,0,0
25.6999999999996,1,273.497222176984,0,0
25.7249999999996,1,273.661472176984,0,0
25.7499999999996,1,273.825722176984,0,0
25.7749999999996,1,273.989972176983,0,0
25.7999999999996,1,274.154222176983,0,0
25.8249999999996,1,274.318472176983,0,0
25.8499999999996,0,274.400597176983,0,0
25.8749999999996,1,274.482722176983,0,0
25.8999999999996,1,274.646972176983,0,0
25.9249999999996,1,274.811222176983,0,0

25.9499999999996,1,274.975472176983,0,0
25.9749999999996,1,275.139722176983,0,0
25.9999999999996,1,275.303972176983,0,0
26.0249999999996,1,275.468222176983,0,0
26.0499999999996,1,275.632472176983,0,0
26.0749999999996,1,275.796722176983,0,0
26.0999999999996,1,275.960972176983,0,0
26.1249999999996,1,276.125222176983,0,0
26.1499999999996,1,276.289472176983,0,0
26.1749999999996,1,276.453722176983,0,0
26.1999999999996,1,276.617972176983,0,0
26.2249999999996,1,276.782222176983,0,0
26.2499999999996,1,276.946472176983,0,0
26.2749999999996,1,277.110722176983,0,0
26.2999999999996,0,277.192847176983,0,0
26.3249999999996,0,277.192847176983,0,0
26.3499999999996,0,277.192847176983,0,0
26.3749999999996,2,277.308989465793,0,0
26.3999999999996,0,277.425131754603,0,0
26.4249999999996,0,277.425131754603,0,0
26.4499999999996,5,277.608768837255,0,0
26.4749999999996,0,277.792405919907,0,0
26.4999999999996,0,277.792405919907,0,0
26.5249999999996,0,277.792405919907,0,0
26.5499999999996,0,277.792405919907,0,0
26.5749999999996,0,277.792405919907,0,0
26.5999999999996,0,277.792405919907,0,0
26.6249999999996,0,277.792405919907,0,0
26.6499999999996,2,277.908548208717,0,0
26.6749999999996,0,278.024690497527,0,0
26.6999999999996,0,278.024690497527,0,0
26.7249999999996,0,278.024690497527,0,0
26.7499999999995,0,278.024690497527,0,0
26.7749999999995,1,278.106815497527,0,0
26.7999999999995,0,278.188940497527,0,0
26.8249999999995,0,278.188940497527,0,0
26.8499999999995,0,278.188940497527,0,0
26.8749999999995,0,278.188940497527,0,0
26.8999999999995,0,278.188940497527,0,0
26.9249999999995,0,278.188940497527,0,0
26.9499999999995,0,278.188940497527,0,0
26.9749999999995,1,278.271065497527,0,0
26.9999999999995,1,278.435315497527,0,0
27.0249999999995,1,278.599565497527,0,0
27.0499999999995,0,278.681690497527,0,0
27.0749999999995,0,278.681690497527,0,0
27.0999999999995,1,278.763815497527,0,0
27.1249999999995,1,278.928065497527,0,0
27.1499999999995,1,279.092315497527,0,0
27.1749999999995,1,279.256565497527,0,0
27.1999999999995,1,279.420815497527,0,0
27.2249999999995,1,279.585065497527,0,0

27.2499999999995,2,279.783332786337,0,0
27.2749999999995,1,279.981600075147,0,0
27.2999999999995,1,280.145850075147,0,0
27.3249999999995,1,280.310100075147,0,0
27.3499999999995,1,280.474350075147,0,0
27.3749999999995,1,280.638600075147,0,0
27.3999999999995,8,280.953009652767,0,0
27.4249999999995,6,281.386458575513,0,0
27.4499999999995,5,281.771260003291,0,0
27.4749999999995,3,282.097141758515,0,0
27.4999999999995,3,282.381631103658,0,0
27.5249999999995,2,282.640018065039,0,0
27.5499999999995,2,282.872302642659,0,0
27.5749999999995,1,283.070569931469,0,0
27.5999999999995,1,283.234819931469,0,0
27.6249999999995,1,283.399069931469,0,0
27.6499999999995,1,283.563319931469,0,0
27.6749999999995,1,283.727569931469,0,0
27.6999999999995,1,283.891819931469,0,0
27.7249999999995,1,284.056069931469,0,0
27.7499999999995,1,284.220319931469,0,0
27.7749999999995,1,284.384569931469,0,0
27.7999999999995,1,284.548819931469,0,0
27.8249999999995,1,284.713069931469,0,0
27.8499999999995,1,284.877319931469,0,0
27.8749999999995,1,285.041569931469,0,0
27.8999999999995,1,285.205819931469,0,0
27.9249999999995,1,285.370069931469,0,0
27.9499999999995,1,285.534319931469,0,0
27.9749999999995,1,285.698569931469,0,0
27.9999999999995,1,285.862819931469,0,0
28.0249999999995,1,286.027069931469,0,0
28.0499999999995,1,286.191319931469,0,0
28.0749999999995,1,286.355569931468,0,0
28.0999999999995,1,286.519819931468,0,0
28.1249999999995,1,286.684069931468,0,0
28.1499999999995,1,286.848319931468,0,0
28.1749999999995,1,287.012569931468,0,0
28.1999999999995,1,287.176819931468,0,0
28.2249999999995,1,287.341069931468,0,0
28.2499999999995,1,287.505319931468,0,0
28.2749999999995,1,287.669569931468,0,0
28.2999999999995,1,287.833819931468,0,0
28.3249999999995,1,287.998069931468,0,0
28.3499999999995,1,288.162319931468,0,0
28.3749999999995,1,288.326569931468,0,0
28.3999999999995,1,288.490819931468,0,0
28.4249999999995,1,288.655069931468,0,0
28.4499999999995,1,288.819319931468,0,0
28.4749999999995,1,288.983569931468,0,0
28.4999999999994,1,289.147819931468,0,0
28.5249999999994,1,289.312069931468,0,0

28.5499999999994,1,289.476319931468,0,0
28.5749999999994,1,289.640569931468,0,0
28.5999999999994,1,289.804819931468,0,0
28.6249999999994,1,289.969069931468,0,0
28.6499999999994,0,290.051194931468,0,0
28.6749999999994,1,290.133319931468,0,0
28.6999999999994,1,290.297569931468,0,0
28.7249999999994,1,290.461819931468,0,0
28.7499999999994,0,290.543944931468,0,0
28.7749999999994,1,290.626069931468,0,0
28.7999999999994,1,290.790319931468,0,0
28.8249999999994,1,290.954569931468,0,0
28.8499999999994,1,291.118819931468,0,0
28.8749999999994,1,291.283069931468,0,0
28.8999999999994,1,291.447319931468,0,0
28.9249999999994,1,291.611569931468,0,0
28.9499999999994,1,291.775819931468,0,0
28.9749999999994,1,291.940069931468,0,0
28.9999999999994,1,292.104319931468,0,0
29.0249999999994,1,292.268569931468,0,0
29.0499999999994,1,292.432819931468,0,0
29.0749999999994,1,292.597069931468,0,0
29.0999999999994,1,292.761319931468,0,0
29.1249999999994,2,292.959587220278,0,0
29.1499999999994,1,293.157854509088,0,0
29.1749999999994,1,293.322104509088,0,0
29.1999999999994,2,293.520371797898,0,0
29.2249999999994,2,293.752656375517,0,0
29.2499999999994,4,294.033048664327,0,0
29.2749999999994,6,294.398463009453,0,0
29.2999999999994,7,294.816909681001,0,0
29.3249999999994,8,295.266476585042,0,0
29.3499999999994,8,295.731045740281,0,0
29.3749999999994,8,296.195614895521,0,0
29.3999999999994,7,296.645181799562,0,0
29.4249999999994,7,297.079746452404,0,0
29.4499999999994,6,297.498193123951,0,0
29.4749999999994,6,297.900521814204,0,0
29.4999999999994,6,298.302850504456,0,0
29.5249999999994,5,298.687651932234,0,0
29.5499999999994,5,299.054926097538,0,0
29.5749999999994,6,299.439727525317,0,0
29.5999999999994,7,299.858174196864,0,0
29.6249999999994,6,300.276620868411,0,0
29.6499999999994,7,300.695067539958,0,0
29.6749999999994,11,301.184727677287,24.7525532162562,1
29.6999999999994,13,301.753211386693,24.7525532162562,1
29.7249999999994,16,302.37781728519,24.7525532162562,1
29.7499999999994,16,303.03481728519,24.7525532162562,1
29.7749999999994,17,303.701927334694,24.7525532162562,1
29.7999999999994,18,304.388964250628,24.7525532162562,1
29.8249999999994,16,305.065891117057,24.7525532162562,1

29.8499999999994,16,305.722891117057,24.7525532162562,1
29.8749999999994,16,306.379891117057,24.7525532162562,1
29.8999999999994,15,307.026459874365,24.7525532162562,1
29.9249999999994,16,307.673028631672,24.7525532162562,1
29.9499999999994,17,308.340138681176,24.7525532162562,1
29.9749999999994,18,309.027175597109,24.7525532162562,1
29.9999999999994,17,309.714212513043,24.7525532162562,1
30.0249999999994,19,310.410797138285,24.7525532162562,1
30.0499999999994,20,311.136045879327,24.7525532162562,1
30.0749999999994,24,311.905648734884,24.7525532162562,1
30.0999999999994,22,312.693177819412,24.7525532162562,1
30.1249999999994,22,313.463578607963,24.7525532162562,1
30.1499999999994,21,314.225123031186,24.7525532162562,1
30.1749999999994,16,314.929967060135,24.7525532162562,1
30.1999999999994,21,315.634811089083,24.7525532162562,1
30.2249999999994,18,316.359581984461,24.7525532162562,1
30.2499999999994,20,317.075283016195,24.7525532162562,1
30.2749999999993,18,317.790984047929,24.7525532162562,1
30.2999999999993,14,318.446694527247,24.7525532162562,1
30.3249999999993,15,319.072046897443,24.7525532162562,1
30.3499999999993,9,319.636490654751,24.7525532162562,1
30.3749999999993,9,320.129240654751,24.7525532162562,1
30.3999999999993,15,320.693684412058,24.7525532162562,1
30.4249999999993,9,321.258128169365,24.7525532162562,1
30.4499999999993,15,321.822571926672,24.7525532162562,1
30.4749999999993,11,322.413018494888,24.7525532162562,1
30.4999999999993,10,322.945098358637,24.7525532162562,1
30.5249999999993,10,323.46450246432,24.7525532162562,1
30.5499999999993,10,323.983906570002,24.7525532162562,1
30.5749999999993,9,324.489983622844,24.7525532162562,1
30.5999999999993,10,324.996060675685,24.7525532162562,1
30.6249999999993,10,325.515464781367,24.7525532162562,1
30.6499999999993,9,326.021541834209,24.7525532162562,1
30.6749999999993,9,326.514291834209,24.7525532162562,1
30.6999999999993,9,327.007041834209,24.7525532162562,1
30.7249999999993,9,327.499791834209,24.7525532162562,1
30.7499999999993,10,328.00586888705,24.7525532162562,1
30.7749999999993,10,328.525272992733,24.7525532162562,1
30.7999999999993,9,329.031350045574,24.7525532162562,1
30.8249999999993,9,329.524100045574,0,0
30.8499999999993,8,330.002759623194,0,0
30.8749999999993,7,330.452326527235,0,0
30.8999999999993,6,330.870773198782,0,0
30.9249999999993,6,331.273101889034,0,0
30.9499999999993,5,331.657903316813,0,0
30.9749999999993,5,332.025177482117,0,0
30.9999999999993,3,332.351059237341,0,0
31.0249999999993,4,332.657553909912,0,0
31.0499999999993,3,332.964048582484,0,0
31.0749999999993,2,333.222435543865,0,0
31.0999999999993,1,333.420702832675,0,0
31.1249999999993,1,333.584952832675,0,0

31.1499999999993,0,333.667077832675,0,0
31.1749999999993,1,333.749202832675,0,0
31.1999999999993,1,333.913452832675,0,0
31.2249999999993,1,334.077702832675,0,0
31.2499999999993,1,334.241952832675,0,0
31.2749999999993,1,334.406202832675,0,0
31.2999999999993,0,334.488327832675,0,0
31.3249999999993,0,334.488327832675,0,0
31.3499999999993,0,334.488327832675,0,0
31.3749999999993,0,334.488327832675,0,0
31.3999999999993,0,334.488327832675,0,0
31.4249999999993,0,334.488327832675,0,0
31.4499999999993,0,334.488327832675,0,0
31.4749999999993,0,334.488327832675,0,0
31.4999999999993,0,334.488327832675,0,0
31.5249999999993,0,334.488327832675,0,0
31.5499999999993,0,334.488327832675,0,0
31.5749999999993,0,334.488327832675,0,0
31.5999999999993,0,334.488327832675,0,0
31.6249999999993,0,334.488327832675,0,0
31.6499999999993,0,334.488327832675,0,0
31.6749999999993,0,334.488327832675,0,0
31.6999999999993,0,334.488327832675,0,0
31.7249999999993,0,334.488327832675,0,0
31.7499999999993,0,334.488327832675,0,0
31.7749999999993,0,334.488327832675,0,0
31.7999999999993,1,334.570452832675,0,0
31.8249999999993,1,334.734702832675,0,0
31.8499999999993,1,334.898952832675,0,0
31.8749999999993,1,335.063202832675,0,0
31.8999999999993,1,335.227452832675,0,0
31.9249999999993,1,335.391702832675,0,0
31.9499999999993,1,335.555952832675,0,0
31.9749999999993,1,335.720202832675,0,0
31.9999999999993,0,335.802327832675,0,0
32.0249999999993,1,335.884452832675,0,0
32.0499999999993,1,336.048702832675,0,0
32.0749999999993,1,336.212952832675,0,0
32.0999999999992,0,336.295077832675,0,0
32.1249999999992,0,336.295077832675,0,0
32.1499999999992,0,336.295077832675,0,0
32.1749999999992,1,336.377202832675,0,0
32.1999999999992,1,336.541452832675,0,0
32.2249999999992,0,336.623577832675,0,0
32.2499999999992,0,336.623577832675,0,0
32.2749999999992,0,336.623577832675,0,0
32.2999999999992,0,336.623577832675,0,0
32.3249999999992,0,336.623577832675,0,0
32.3499999999992,0,336.623577832675,0,0
32.3749999999992,0,336.623577832675,0,0
32.3999999999992,0,336.623577832675,0,0
32.4249999999992,0,336.623577832675,0,0

32.4499999999992,0,336.623577832675,0,0
32.4749999999992,0,336.623577832675,0,0
32.4999999999992,0,336.623577832675,0,0
32.5249999999992,0,336.623577832675,0,0
32.5499999999992,0,336.623577832675,0,0
32.5749999999992,0,336.623577832675,0,0
32.5999999999992,0,336.623577832675,0,0
32.6249999999992,1,336.705702832675,0,0
32.6499999999992,1,336.869952832675,0,0
32.6749999999992,1,337.034202832675,0,0
32.6999999999992,1,337.198452832675,0,0
32.7249999999992,1,337.362702832675,0,0
32.7499999999992,1,337.526952832675,0,0
32.7749999999992,1,337.691202832675,0,0
32.7999999999992,1,337.855452832675,0,0
32.8249999999992,1,338.019702832675,0,0
32.8499999999992,1,338.183952832675,0,0
32.8749999999992,0,338.266077832675,0,0
32.8999999999992,1,338.348202832675,0,0
32.9249999999992,1,338.512452832675,0,0
32.9499999999992,1,338.676702832675,0,0
32.9749999999992,1,338.840952832675,0,0
32.9999999999992,0,338.923077832675,0,0
33.0249999999992,1,339.005202832675,0,0
33.0499999999992,1,339.169452832675,0,0
33.0749999999992,1,339.333702832675,0,0
33.0999999999992,1,339.497952832675,0,0
33.1249999999992,1,339.662202832675,0,0
33.1499999999992,1,339.826452832675,0,0
33.1749999999992,1,339.990702832675,0,0
33.1999999999992,1,340.154952832675,0,0
33.2249999999992,0,340.237077832675,0,0
33.2499999999992,1,340.319202832675,0,0
33.2749999999992,1,340.483452832675,0,0
33.2999999999992,1,340.647702832675,0,0
33.3249999999992,1,340.811952832675,0,0
33.3499999999992,0,340.894077832675,0,0
33.3749999999992,1,340.976202832675,0,0
33.3999999999992,0,341.058327832675,0,0
33.4249999999992,1,341.140452832675,0,0
33.4499999999992,0,341.222577832675,0,0
33.4749999999992,1,341.304702832675,0,0
33.4999999999992,1,341.468952832675,0,0
33.5249999999992,1,341.633202832675,0,0
33.5499999999992,1,341.797452832675,0,0
33.5749999999992,0,341.879577832675,0,0
33.5999999999992,0,341.879577832675,0,0
33.6249999999992,1,341.961702832675,0,0
33.6499999999992,1,342.125952832675,0,0
33.6749999999992,0,342.208077832675,0,0
33.6999999999992,1,342.290202832675,0,0
33.7249999999992,1,342.454452832675,0,0

```
33.7499999999992,1,342.618702832675,0,0
33.7749999999992,1,342.782952832675,0,0
33.7999999999992,3,343.007322505246,0,0
33.8249999999992,0,343.149567177818,0,0
33.8499999999991,0,343.149567177818,0,0
33.8749999999991,0,343.149567177818,0,0
33.8999999999991,5,343.33320426047,0,0
33.9249999999991,0,343.516841343122,0,0
33.9499999999991,0,343.516841343122,0,0
33.9749999999991,4,343.681091343122,0,0
33.9999999999991,3,343.987586015694,0,0
34.0249999999991,2,344.245972977076,0,0
34.0499999999991,0,344.362115265885,0,0
34.0749999999991,0,344.362115265885,0,0
34.0999999999991,2,344.478257554695,0,0
34.1249999999991,6,344.795564188631,0,0
34.1499999999991,2,345.112870822567,0,0
34.1749999999991,0,345.229013111377,0,0
34.1999999999991,1,345.311138111377,0,0
34.2249999999991,0,345.393263111377,0,0
34.2499999999991,1,345.475388111377,0,0
34.2749999999991,1,345.639638111377,0,0
34.2999999999991,0,345.721763111377,0,0
34.3249999999991,3,345.864007783949,0,0
34.3499999999991,1,346.08837745652,0,0
34.3749999999991,0,346.17050245652,0,0
34.3999999999991,3,346.312747129092,0,0
34.4249999999991,3,346.597236474235,0,0
34.4499999999991,1,346.821606146807,0,0
34.4749999999991,2,347.019873435617,0,0
34.4999999999991,3,347.278260396998,0,0
34.5249999999991,3,347.562749742141,0,0
34.5499999999991,3,347.847239087285,0,0
34.5749999999991,3,348.131728432428,0,0
34.5999999999991,2,348.390115393809,0,0
34.6249999999991,2,348.622399971429,0,0
34.6499999999991,3,348.88078693281,0,0
34.6749999999991,3,349.165276277954,0,0
34.6999999999991,3,349.449765623097,0,0
34.7249999999991,4,349.756260295668,0,0
34.7499999999991,4,350.084760295668,0,0
34.7749999999991,5,350.432647378321,0,0
34.7999999999991,5,350.799921543625,0,0
34.8249999999991,5,351.167195708929,0,0
34.8499999999991,6,351.551997136708,0,0
34.8749999999991,7,351.970443808255,0,0
34.8999999999991,8,352.420010712296,0,0
34.9249999999991,9,352.898670289916,0,0
34.9499999999991,11,353.417423100824,24.6046342972803,1
34.9749999999991,12,353.974290256875,24.6046342972803,1
34.9999999999991,13,354.554885500515,24.6046342972803,1
35.0249999999991,13,355.14709729751,24.6046342972803,1
```

35.0499999999991,13,355.739309094505,24.6046342972803,1
35.0749999999991,12,356.319904338146,24.6046342972803,1
35.0999999999991,13,356.900499581787,24.6046342972803,1
35.1249999999991,12,357.481094825427,24.6046342972803,1
35.1499999999991,13,358.061690069068,24.6046342972803,1
35.1749999999991,12,358.642285312709,24.6046342972803,1
35.1999999999991,13,359.222880556349,24.6046342972803,1
35.2249999999991,12,359.80347579999,24.6046342972803,1
35.2499999999991,13,360.384071043631,24.6046342972803,1
35.2749999999991,13,360.976282840626,24.6046342972803,1
35.2999999999991,13,361.568494637621,24.6046342972803,1
35.3249999999991,12,362.149089881261,24.6046342972803,1
35.3499999999991,13,362.729685124902,24.6046342972803,1
35.3749999999991,15,363.343859780707,24.6046342972803,1
35.3999999999991,17,364.000538587518,24.6046342972803,1
35.4249999999991,19,364.69712321276,24.6046342972803,1
35.4499999999991,19,365.413072364237,24.6046342972803,1
35.4749999999991,17,366.109656989479,24.6046342972803,1
35.4999999999991,19,366.806241614721,24.6046342972803,1
35.5249999999991,18,367.512643056889,24.6046342972803,1
35.5499999999991,17,368.199679972822,24.6046342972803,1
35.5749999999991,17,368.87690007183,24.6046342972803,1
35.599999999999,16,369.544010121334,24.6046342972803,1
35.624999999999,15,370.190578878641,24.6046342972803,1
35.649999999999,15,370.826716393256,24.6046342972803,1
35.674999999999,19,371.502759726301,24.6046342972803,1
35.699999999999,14,372.168017914928,24.6046342972803,1
35.724999999999,13,372.771407426315,24.6046342972803,1
35.749999999999,14,373.374796937701,24.6046342972803,1
35.774999999999,12,373.966569895733,24.6046342972803,1
35.799999999999,11,374.523437051784,24.6046342972803,1
35.824999999999,10,375.055516915533,24.6046342972803,1
35.849999999999,9,375.561593968375,24.6046342972803,1
35.874999999999,8,376.040253545994,0,0
35.899999999999,7,376.489820450035,0,0
35.924999999999,7,376.924385102878,0,0
35.949999999999,9,377.388042429299,0,0
35.974999999999,8,377.866702006919,0,0
35.999999999999,7,378.31626891096,0,0
36.024999999999,7,378.750833563802,0,0
36.049999999999,6,379.169280235349,0,0
36.074999999999,6,379.571608925601,0,0
36.099999999999,6,379.973937615854,0,0
36.124999999999,6,380.376266306106,0,0
36.149999999999,5,380.761067733884,0,0
36.174999999999,4,381.108954816536,0,0
36.199999999999,5,381.456841899188,0,0
36.224999999999,4,381.804728981841,0,0
36.249999999999,4,382.133228981841,0,0
36.274999999999,4,382.461728981841,0,0
36.299999999999,4,382.790228981841,0,0
36.324999999999,4,383.118728981841,0,0

36.349999999999,3,383.425223654412,0,0
36.374999999999,3,383.709712999555,0,0
36.399999999999,3,383.994202344699,0,0
36.424999999999,3,384.278691689842,0,0
36.449999999999,3,384.563181034985,0,0
36.474999999999,2,384.821567996366,0,0
36.499999999999,2,385.053852573986,0,0
36.524999999999,2,385.286137151606,0,0
36.549999999999,2,385.518421729226,0,0
36.574999999999,2,385.750706306846,0,0
36.599999999999,2,385.982990884465,0,0
36.624999999999,4,386.263383173275,0,0
36.649999999999,2,386.543775462085,0,0
36.674999999999,2,386.776060039705,0,0
36.699999999999,2,387.008344617325,0,0
36.724999999999,2,387.240629194945,0,0
36.749999999999,1,387.438896483754,0,0
36.774999999999,2,387.637163772564,0,0
36.799999999999,2,387.869448350184,0,0
36.824999999999,2,388.101732927804,0,0
36.849999999999,1,388.300000216614,0,0
36.874999999999,1,388.464250216614,0,0
36.899999999999,2,388.662517505424,0,0
36.924999999999,1,388.860784794234,0,0
36.949999999999,1,389.025034794234,0,0
36.974999999999,1,389.189284794234,0,0
36.999999999999,1,389.353534794234,0,0
37.024999999999,1,389.517784794234,0,0
37.049999999999,1,389.682034794234,0,0
37.074999999999,1,389.846284794234,0,0
37.099999999999,0,389.928409794234,0,0
37.124999999999,1,390.010534794234,0,0
37.149999999999,1,390.174784794234,0,0
37.174999999999,1,390.339034794234,0,0
37.199999999999,1,390.503284794234,0,0
37.224999999999,1,390.667534794234,0,0
37.249999999999,1,390.831784794234,0,0
37.274999999999,1,390.996034794233,0,0
37.299999999999,1,391.160284794233,0,0
37.324999999999,1,391.324534794233,0,0
37.349999999999,1,391.488784794233,0,0
37.3749999999989,1,391.653034794233,0,0
37.3999999999989,1,391.817284794233,0,0
37.4249999999989,1,391.981534794233,0,0
37.4499999999989,1,392.145784794233,0,0
37.4749999999989,1,392.310034794233,0,0
37.4999999999989,1,392.474284794233,0,0
37.5249999999989,1,392.638534794233,0,0
37.5499999999989,1,392.802784794233,0,0
37.5749999999989,1,392.967034794233,0,0
37.5999999999989,0,393.049159794233,0,0
37.6249999999989,3,393.191404466805,0,0

37.6499999999989,2,393.449791428186,0,0
37.6749999999989,4,393.730183716996,0,0
37.6999999999989,0,393.894433716996,0,0
37.7249999999989,1,393.976558716996,0,0
37.7499999999989,5,394.242320799648,0,0
37.7749999999989,0,394.425957882301,0,0
37.7999999999989,0,394.425957882301,0,0
37.8249999999989,1,394.508082882301,0,0
37.8499999999989,0,394.590207882301,0,0
37.8749999999989,0,394.590207882301,0,0
37.8999999999989,1,394.672332882301,0,0
37.9249999999989,0,394.754457882301,0,0
37.9499999999989,0,394.754457882301,0,0
37.9749999999989,1,394.836582882301,0,0
37.9999999999989,1,395.000832882301,0,0
38.0249999999989,0,395.082957882301,0,0
38.0499999999989,0,395.082957882301,0,0
38.0749999999989,0,395.082957882301,0,0
38.0999999999989,0,395.082957882301,0,0
38.1249999999989,0,395.082957882301,0,0
38.1499999999989,0,395.082957882301,0,0
38.1749999999989,1,395.165082882301,0,0
38.1999999999989,1,395.329332882301,0,0
38.2249999999989,1,395.493582882301,0,0
38.2499999999989,1,395.657832882301,0,0
38.2749999999989,1,395.822082882301,0,0
38.2999999999989,1,395.986332882301,0,0
38.3249999999989,1,396.150582882301,0,0
38.3499999999989,1,396.314832882301,0,0
38.3749999999989,1,396.479082882301,0,0
38.3999999999989,1,396.643332882301,0,0
38.4249999999989,1,396.8075828823,0,0
38.4499999999989,1,396.9718328823,0,0
38.4749999999989,0,397.0539578823,0,0
38.4999999999989,0,397.0539578823,0,0
38.5249999999989,1,397.136082882301,0,0
38.5499999999989,1,397.3003328823,0,0
38.5749999999989,1,397.4645828823,0,0
38.5999999999989,1,397.6288328823,0,0
38.6249999999989,1,397.7930828823,0,0
38.6499999999989,1,397.9573328823,0,0
38.6749999999989,0,398.0394578823,0,0
38.6999999999989,0,398.0394578823,0,0
38.7249999999989,1,398.1215828823,0,0
38.7499999999989,1,398.2858328823,0,0
38.7749999999989,0,398.3679578823,0,0
38.7999999999989,1,398.4500828823,0,0
38.8249999999989,1,398.6143328823,0,0
38.8499999999989,0,398.6964578823,0,0
38.8749999999989,0,398.6964578823,0,0
38.8999999999989,0,398.6964578823,0,0
38.9249999999989,0,398.6964578823,0,0

38.9499999999989,0,398.6964578823,0,0
38.9749999999989,1,398.778582882301,0,0
38.9999999999989,1,398.9428328823,0,0
39.0249999999989,1,399.1070828823,0,0
39.0499999999989,1,399.2713328823,0,0
39.0749999999989,0,399.3534578823,0,0
39.0999999999989,0,399.3534578823,0,0
39.1249999999988,1,399.4355828823,0,0
39.1499999999988,1,399.5998328823,0,0
39.1749999999988,1,399.7640828823,0,0
39.1999999999988,1,399.9283328823,0,0
39.2249999999988,0,400.0104578823,0,0
39.2499999999988,0,400.0104578823,0,0
39.2749999999988,0,400.0104578823,0,0
39.2999999999988,0,400.0104578823,0,0
39.3249999999988,1,400.0925828823,0,0
39.3499999999988,1,400.2568328823,0,0
39.3749999999988,0,400.3389578823,0,0
39.3999999999988,0,400.3389578823,0,0
39.4249999999988,2,400.45510017111,0,0
39.4499999999988,0,400.57124245992,0,0
39.4749999999988,1,400.65336745992,0,0
39.4999999999988,1,400.81761745992,0,0
39.5249999999988,1,400.98186745992,0,0
39.5499999999988,1,401.14611745992,0,0
39.5749999999988,1,401.31036745992,0,0
39.5999999999988,1,401.47461745992,0,0
39.6249999999988,1,401.63886745992,0,0
39.6499999999988,1,401.80311745992,0,0
39.6749999999988,1,401.96736745992,0,0
39.6999999999988,1,402.13161745992,0,0
39.7249999999988,1,402.29586745992,0,0
39.7499999999988,0,402.37799245992,0,0
39.7749999999988,1,402.46011745992,0,0
39.7999999999988,1,402.62436745992,0,0
39.8249999999988,0,402.70649245992,0,0
39.8499999999988,1,402.78861745992,0,0
39.8749999999988,1,402.95286745992,0,0
39.8999999999988,0,403.03499245992,0,0
39.9249999999988,0,403.03499245992,0,0
39.9499999999988,0,403.03499245992,0,0
39.9749999999988,0,403.03499245992,0,0
39.9999999999988,1,403.11711745992,0,0
40.0249999999988,1,403.28136745992,0,0
40.0499999999988,1,403.44561745992,0,0
40.0749999999988,1,403.60986745992,0,0
40.0999999999988,1,403.77411745992,0,0
40.1249999999988,0,403.85624245992,0,0
40.1499999999988,1,403.93836745992,0,0
40.1749999999988,0,404.02049245992,0,0
40.1999999999988,0,404.02049245992,0,0
40.2249999999988,0,404.02049245992,0,0

40.2499999999988,0,404.02049245992,0,0
40.2749999999988,1,404.10261745992,0,0
40.2999999999988,1,404.26686745992,0,0
40.3249999999988,0,404.34899245992,0,0
40.3499999999988,1,404.43111745992,0,0
40.3749999999988,0,404.51324245992,0,0
40.3999999999988,0,404.51324245992,0,0
40.4249999999988,0,404.51324245992,0,0
40.4499999999988,0,404.51324245992,0,0
40.4749999999988,0,404.51324245992,0,0
40.4999999999988,0,404.51324245992,0,0
40.5249999999988,0,404.51324245992,0,0
40.5499999999988,0,404.51324245992,0,0
40.5749999999988,0,404.51324245992,0,0
40.5999999999988,0,404.51324245992,0,0
40.6249999999988,0,404.51324245992,0,0
40.6499999999988,0,404.51324245992,0,0
40.6749999999988,0,404.51324245992,0,0
40.6999999999988,0,404.51324245992,0,0
40.7249999999988,0,404.51324245992,0,0
40.7499999999988,0,404.51324245992,0,0
40.7749999999988,1,404.59536745992,0,0
40.7999999999988,1,404.75961745992,0,0
40.8249999999988,1,404.92386745992,0,0
40.8499999999988,0,405.00599245992,0,0
40.8749999999987,1,405.08811745992,0,0
40.8999999999987,1,405.25236745992,0,0
40.9249999999987,0,405.33449245992,0,0
40.9499999999987,0,405.33449245992,0,0
40.9749999999987,1,405.41661745992,0,0
40.9999999999987,0,405.49874245992,0,0
41.0249999999987,1,405.58086745992,0,0
41.0499999999987,1,405.74511745992,0,0
41.0749999999987,1,405.90936745992,0,0
41.0999999999987,1,406.07361745992,0,0
41.1249999999987,1,406.23786745992,0,0
41.1499999999987,1,406.40211745992,0,0
41.1749999999987,1,406.56636745992,0,0
41.1999999999987,1,406.73061745992,0,0
41.2249999999987,1,406.89486745992,0,0
41.2499999999987,1,407.05911745992,0,0
41.2749999999987,1,407.22336745992,0,0
41.2999999999987,0,407.30549245992,0,0
41.3249999999987,0,407.30549245992,0,0
41.3499999999987,1,407.38761745992,0,0
41.3749999999987,1,407.55186745992,0,0
41.3999999999987,2,407.75013474873,0,0
41.4249999999987,4,408.03052703754,0,0
41.4499999999987,8,408.42706161516,0,0
41.4749999999987,5,408.842983275432,0,0
41.4999999999987,6,409.22778470321,0,0
41.5249999999987,4,409.593199048336,0,0

41.5499999999987,3,409.899693720908,0,0
41.5749999999987,8,410.274222971099,0,0
41.5999999999987,15,410.824576306026,38.7071340543577,1
41.6249999999987,28,411.577209716176,38.7071340543577,1
41.6499999999987,49,412.586649369018,38.7071340543577,1
41.6749999999987,60,413.797661883633,38.7071340543577,1
41.6999999999987,56,415.048366624025,38.7071340543577,1
41.7249999999987,62,416.309586746456,38.7071340543577,1
41.7499999999987,57,417.576269796105,38.7071340543577,1
41.7749999999987,52,418.788511746097,38.7071340543577,1
41.7999999999987,48,419.949702233378,38.7071340543577,1
41.8249999999987,45,421.069592171621,38.7071340543577,1
41.8499999999987,40,422.13990752526,38.7071340543577,1
41.8749999999987,39,423.172182091561,38.7071340543577,1
41.8999999999987,35,424.170910604367,38.7071340543577,1
41.9249999999987,33,425.128540863901,38.7071340543577,1
41.9499999999987,33,426.072085278595,38.7071340543577,1
41.9749999999987,33,427.015629693289,38.7071340543577,1
41.9999999999987,34,427.966268825,38.7071340543577,1
42.0249999999987,38,428.951388249358,38.7071340543577,1
42.0499999999987,41,429.983497327351,38.7071340543577,1
42.0749999999987,41,431.035210483349,38.7071340543577,1
42.0999999999987,39,432.073937521967,38.7071340543577,1
42.1249999999987,40,433.106212088268,38.7071340543577,1
42.1499999999987,37,434.125163066751,38.7071340543577,1
42.1749999999987,35,435.110567991739,38.7071340543577,1
42.1999999999987,30,436.046243194277,38.7071340543577,1
42.2249999999987,26,436.914817322182,38.7071340543577,1
42.2499999999987,23,437.727431963588,38.7071340543577,1
42.2749999999987,20,438.488563792744,38.7071340543577,1
42.2999999999987,17,439.194448007553,38.7071340543577,1
42.3249999999987,12,439.8175474022,38.7071340543577,1
42.3499999999987,10,440.361738800184,38.7071340543577,1
42.3749999999987,6,440.822605198152,38.7071340543577,1
42.3999999999987,3,441.166014215849,0,0
42.4249999999987,2,441.424401177231,0,0
42.4499999999987,0,441.540543466041,0,0
42.4749999999987,0,441.540543466041,0,0
42.4999999999987,0,441.540543466041,0,0
42.5249999999987,0,441.540543466041,0,0
42.5499999999987,0,441.540543466041,0,0
42.5749999999987,0,441.540543466041,0,0
42.5999999999987,0,441.540543466041,0,0
42.6249999999987,0,441.540543466041,0,0
42.6499999999986,0,441.540543466041,0,0
42.6749999999986,0,441.540543466041,0,0
42.6999999999986,0,441.540543466041,0,0
42.7249999999986,0,441.540543466041,0,0
42.7499999999986,0,441.540543466041,0,0
42.7749999999986,0,441.540543466041,0,0
42.7999999999986,2,441.65668575485,0,0
42.8249999999986,1,441.85495304366,0,0

42.8499999999986,0,441.93707804366,0,0
42.8749999999986,0,441.93707804366,0,0
42.8999999999986,1,442.01920304366,0,0
42.9249999999986,0,442.10132804366,0,0
42.9499999999986,0,442.10132804366,0,0
42.9749999999986,1,442.18345304366,0,0
42.9999999999986,0,442.26557804366,0,0
43.0249999999986,0,442.26557804366,0,0
43.0499999999986,1,442.34770304366,0,0
43.0749999999986,0,442.429828043661,0,0
43.0999999999986,1,442.511953043661,0,0
43.1249999999986,1,442.676203043661,0,0
43.1499999999986,1,442.84045304366,0,0
43.1749999999986,0,442.922578043661,0,0
43.1999999999986,0,442.922578043661,0,0
43.2249999999986,0,442.922578043661,0,0
43.2499999999986,0,442.922578043661,0,0
43.2749999999986,1,443.004703043661,0,0
43.2999999999986,1,443.168953043661,0,0
43.3249999999986,1,443.33320304366,0,0
43.3499999999986,1,443.49745304366,0,0
43.3749999999986,0,443.57957804366,0,0
43.3999999999986,1,443.661703043661,0,0
43.4249999999986,1,443.82595304366,0,0
43.4499999999986,0,443.908078043661,0,0
43.4749999999986,0,443.908078043661,0,0
43.4999999999986,0,443.908078043661,0,0
43.5249999999986,1,443.990203043661,0,0
43.5499999999986,1,444.154453043661,0,0
43.5749999999986,1,444.31870304366,0,0
43.5999999999986,1,444.48295304366,0,0
43.6249999999986,1,444.64720304366,0,0
43.6499999999986,0,444.72932804366,0,0
43.6749999999986,0,444.72932804366,0,0
43.6999999999986,0,444.72932804366,0,0
43.7249999999986,0,444.72932804366,0,0
43.7499999999986,0,444.72932804366,0,0
43.7749999999986,0,444.72932804366,0,0
43.7999999999986,0,444.72932804366,0,0
43.8249999999986,0,444.72932804366,0,0
43.8499999999986,0,444.72932804366,0,0
43.8749999999986,0,444.72932804366,0,0
43.8999999999986,0,444.72932804366,0,0
43.9249999999986,0,444.72932804366,0,0
43.9499999999986,0,444.72932804366,0,0
43.9749999999986,0,444.72932804366,0,0
43.9999999999986,0,444.72932804366,0,0
44.0249999999986,0,444.72932804366,0,0
44.0499999999986,0,444.72932804366,0,0
44.0749999999986,0,444.72932804366,0,0
44.0999999999986,0,444.72932804366,0,0
44.1249999999986,1,444.81145304366,0,0

44.1499999999986,0,444.893578043661,0,0
44.1749999999986,0,444.893578043661,0,0
44.1999999999986,0,444.893578043661,0,0
44.2249999999986,0,444.893578043661,0,0
44.2499999999986,0,444.893578043661,0,0
44.2749999999986,0,444.893578043661,0,0
44.2999999999986,0,444.893578043661,0,0
44.3249999999986,0,444.893578043661,0,0
44.3499999999986,0,444.893578043661,0,0
44.3749999999986,0,444.893578043661,0,0
44.3999999999985,0,444.893578043661,0,0
44.4249999999985,0,444.893578043661,0,0
44.4499999999985,0,444.893578043661,0,0
44.4749999999985,0,444.893578043661,0,0
44.4999999999985,0,444.893578043661,0,0
44.5249999999985,0,444.893578043661,0,0
44.5499999999985,0,444.893578043661,0,0
44.5749999999985,0,444.893578043661,0,0
44.5999999999985,0,444.893578043661,0,0
44.6249999999985,0,444.893578043661,0,0
44.6499999999985,0,444.893578043661,0,0
44.6749999999985,0,444.893578043661,0,0
44.6999999999985,0,444.893578043661,0,0
44.7249999999985,0,444.893578043661,0,0
44.7499999999985,0,444.893578043661,0,0
44.7749999999985,0,444.893578043661,0,0
44.7999999999985,0,444.893578043661,0,0
44.8249999999985,0,444.893578043661,0,0
44.8499999999985,0,444.893578043661,0,0
44.8749999999985,1,444.975703043661,0,0
44.8999999999985,1,445.139953043661,0,0
44.9249999999985,0,445.222078043661,0,0
44.9499999999985,1,445.304203043661,0,0
44.9749999999985,1,445.468453043661,0,0
44.9999999999985,1,445.632703043661,0,0
45.0249999999985,1,445.79695304366,0,0
45.0499999999985,1,445.96120304366,0,0
45.0749999999985,1,446.12545304366,0,0
45.0999999999985,1,446.28970304366,0,0
45.1249999999985,0,446.37182804366,0,0
45.1499999999985,3,446.514072716232,0,0
45.1749999999985,0,446.656317388804,0,0
45.1999999999985,0,446.656317388804,0,0
45.2249999999985,1,446.738442388804,0,0
45.2499999999985,3,446.962812061375,0,0
45.2749999999985,6,447.306221079073,0,0
45.2999999999985,0,447.507385424199,0,0
45.3249999999985,1,447.589510424199,0,0
45.3499999999985,3,447.813880096771,0,0
45.3749999999985,1,448.038249769342,0,0
45.3999999999985,3,448.262619441914,0,0
45.4249999999985,0,448.404864114485,0,0

45.4499999999985,0,448.404864114485,0,0
45.4749999999985,0,448.404864114485,0,0
45.4999999999985,0,448.404864114485,0,0
45.5249999999985,0,448.404864114485,0,0
45.5499999999985,0,448.404864114485,0,0
45.5749999999985,0,448.404864114485,0,0
45.5999999999985,1,448.486989114485,0,0
45.6249999999985,0,448.569114114485,0,0
45.6499999999985,0,448.569114114485,0,0
45.6749999999985,0,448.569114114485,0,0
45.6999999999985,0,448.569114114485,0,0
45.7249999999985,1,448.651239114485,0,0
45.7499999999985,0,448.733364114485,0,0
45.7749999999985,0,448.733364114485,0,0
45.7999999999985,1,448.815489114486,0,0
45.8249999999985,0,448.897614114486,0,0
45.8499999999985,1,448.979739114486,0,0
45.8749999999985,0,449.061864114486,0,0
45.8999999999985,1,449.143989114486,0,0
45.9249999999985,0,449.226114114486,0,0
45.9499999999985,1,449.308239114486,0,0
45.9749999999985,1,449.472489114486,0,0
45.9999999999985,0,449.554614114486,0,0
46.0249999999985,0,449.554614114486,0,0
46.0499999999985,1,449.636739114486,0,0
46.0749999999985,1,449.800989114486,0,0
46.0999999999985,1,449.965239114486,0,0
46.1249999999985,1,450.129489114486,0,0
46.1499999999985,1,450.293739114486,0,0
46.1749999999984,1,450.457989114486,0,0
46.1999999999984,1,450.622239114486,0,0
46.2249999999984,0,450.704364114486,0,0
46.2499999999984,1,450.786489114486,0,0
46.2749999999984,1,450.950739114486,0,0
46.2999999999984,0,451.032864114486,0,0
46.3249999999984,1,451.114989114486,0,0
46.3499999999984,1,451.279239114486,0,0
46.3749999999984,0,451.361364114486,0,0
46.3999999999984,1,451.443489114486,0,0
46.4249999999984,1,451.607739114486,0,0
46.4499999999984,0,451.689864114486,0,0
46.4749999999984,0,451.689864114486,0,0
46.4999999999984,0,451.689864114486,0,0
46.5249999999984,0,451.689864114486,0,0
46.5499999999984,1,451.771989114486,0,0
46.5749999999984,0,451.854114114486,0,0
46.5999999999984,1,451.936239114486,0,0
46.6249999999984,0,452.018364114486,0,0
46.6499999999984,1,452.100489114486,0,0
46.6749999999984,0,452.182614114486,0,0
46.6999999999984,1,452.264739114486,0,0
46.7249999999984,1,452.428989114486,0,0

46.7499999999984,1,452.593239114486,0,0
46.7749999999984,1,452.757489114486,0,0
46.7999999999984,1,452.921739114486,0,0
46.8249999999984,2,453.120006403296,0,0
46.8499999999984,3,453.378393364677,0,0
46.8749999999984,6,453.721802382375,0,0
46.8999999999984,5,454.106603810153,0,0
46.9249999999984,6,454.491405237931,0,0
46.9499999999984,3,454.834814255629,0,0
46.9749999999984,1,455.0591839282,0,0
46.9999999999984,2,455.25745121701,0,0
47.0249999999984,2,455.48973579463,0,0
47.0499999999984,3,455.748122756012,0,0
47.0749999999984,4,456.054617428583,0,0
47.0999999999984,5,456.402504511235,0,0
47.1249999999984,5,456.76977867654,0,0
47.1499999999984,6,457.154580104318,0,0
47.1749999999984,7,457.573026775865,0,0
47.1999999999984,8,458.022593679906,0,0
47.2249999999984,9,458.501253257526,0,0
47.2499999999984,9,458.994003257526,0,0
47.2749999999984,8,459.472662835146,0,0
47.2999999999984,8,459.937231990385,0,0
47.3249999999984,7,460.386798894426,0,0
47.3499999999984,8,460.836365798467,0,0
47.3749999999984,9,461.315025376087,0,0
47.3999999999984,9,461.807775376087,0,0
47.4249999999984,10,462.313852428928,21.8427186116542,1
47.4499999999984,12,462.858043826913,21.8427186116542,1
47.4749999999984,14,463.449816784945,21.8427186116542,1
47.4999999999984,17,464.095710447338,21.8427186116542,1
47.5249999999984,18,464.782747363271,21.8427186116542,1
47.5499999999984,17,465.469784279205,21.8427186116542,1
47.5749999999984,15,466.126463086016,21.8427186116542,1
47.5999999999984,12,466.729021188466,21.8427186116542,1
47.6249999999984,8,467.245795111229,21.8427186116542,1
47.6499999999984,7,467.69536201527,21.8427186116542,1
47.6749999999984,7,468.129926668113,21.8427186116542,1
47.6999999999984,7,468.564491320955,21.8427186116542,1
47.7249999999984,8,469.014058224996,21.8427186116542,1
47.7499999999984,9,469.492717802616,21.8427186116542,1
47.7749999999984,10,469.998794855457,21.8427186116542,1
47.7999999999984,9,470.504871908298,21.8427186116542,1
47.8249999999984,7,470.968529234719,0,0
47.8499999999984,7,471.403093887562,0,0
47.8749999999984,7,471.837658540404,0,0
47.8999999999984,6,472.256105211951,0,0
47.9249999999983,5,472.64090663973,0,0
47.9499999999983,4,472.988793722382,0,0
47.9749999999983,4,473.317293722382,0,0
47.9999999999983,5,473.665180805034,0,0
48.0249999999983,4,474.013067887686,0,0

48.0499999999983,3,474.319562560258,0,0
48.0749999999983,3,474.604051905401,0,0
48.0999999999983,3,474.888541250544,0,0
48.1249999999983,3,475.173030595687,0,0
48.1499999999983,3,475.457519940831,0,0
48.1749999999983,2,475.715906902212,0,0
48.1999999999983,2,475.948191479832,0,0
48.2249999999983,2,476.180476057452,0,0
48.2499999999983,2,476.412760635071,0,0
48.2749999999983,2,476.645045212691,0,0
48.2999999999983,3,476.903432174073,0,0
48.3249999999983,3,477.187921519216,0,0
48.3499999999983,5,477.51380327444,0,0
48.3749999999983,4,477.861690357092,0,0
48.3999999999983,4,478.190190357092,0,0
48.4249999999983,4,478.518690357092,0,0
48.4499999999983,3,478.825185029663,0,0
48.4749999999983,2,479.083571991045,0,0
48.4999999999983,2,479.315856568665,0,0
48.5249999999983,2,479.548141146284,0,0
48.5499999999983,1,479.746408435094,0,0
48.5749999999983,1,479.910658435094,0,0
48.5999999999983,1,480.074908435094,0,0
48.6249999999983,1,480.239158435094,0,0
48.6499999999983,1,480.403408435094,0,0
48.6749999999983,1,480.567658435094,0,0
48.6999999999983,1,480.731908435094,0,0
48.7249999999983,1,480.896158435094,0,0
48.7499999999983,1,481.060408435094,0,0
48.7749999999983,1,481.224658435094,0,0
48.7999999999983,1,481.388908435094,0,0
48.8249999999983,1,481.553158435094,0,0
48.8499999999983,0,481.635283435094,0,0
48.8749999999983,0,481.635283435094,0,0
48.8999999999983,6,481.83644778022,0,0
48.9249999999983,0,482.037612125346,0,0
48.9499999999983,2,482.153754414156,0,0
48.9749999999983,1,482.352021702966,0,0
48.9999999999983,0,482.434146702966,0,0
49.0249999999983,0,482.434146702966,0,0
49.0499999999983,0,482.434146702966,0,0
49.0749999999983,0,482.434146702966,0,0
49.0999999999983,0,482.434146702966,0,0
49.1249999999983,4,482.598396702966,0,0
49.1499999999983,0,482.762646702966,0,0
49.1749999999983,1,482.844771702966,0,0
49.1999999999983,0,482.926896702966,0,0
49.2249999999983,0,482.926896702966,0,0
49.2499999999983,0,482.926896702966,0,0
49.2749999999983,0,482.926896702966,0,0
49.2999999999983,0,482.926896702966,0,0
49.3249999999983,1,483.009021702966,0,0

49.3499999999983,0,483.091146702966,0,0
49.3749999999983,0,483.091146702966,0,0
49.3999999999983,0,483.091146702966,0,0
49.4249999999983,0,483.091146702966,0,0
49.4499999999983,0,483.091146702966,0,0
49.4749999999983,1,483.173271702966,0,0
49.4999999999983,0,483.255396702966,0,0
49.5249999999983,0,483.255396702966,0,0
49.5499999999983,0,483.255396702966,0,0
49.5749999999983,1,483.337521702966,0,0
49.5999999999983,1,483.501771702966,0,0
49.6249999999983,0,483.583896702966,0,0
49.6499999999983,1,483.666021702966,0,0
49.6749999999982,1,483.830271702966,0,0
49.6999999999982,1,483.994521702966,0,0
49.7249999999982,1,484.158771702966,0,0
49.7499999999982,0,484.240896702966,0,0
49.7749999999982,1,484.323021702966,0,0
49.7999999999982,1,484.487271702966,0,0
49.8249999999982,1,484.651521702966,0,0
49.8499999999982,0,484.733646702966,0,0
49.8749999999982,0,484.733646702966,0,0
49.8999999999982,0,484.733646702966,0,0
49.9249999999982,0,484.733646702966,0,0
49.9499999999982,0,484.733646702966,0,0
49.9749999999982,1,484.815771702966,0,0
49.9999999999982,0,484.897896702966,0,0
50.0249999999982,0,484.897896702966,0,0
50.0499999999982,0,484.897896702966,0,0
50.0749999999982,0,484.897896702966,0,0
50.0999999999982,0,484.897896702966,0,0
50.1249999999982,0,484.897896702966,0,0
50.1499999999982,0,484.897896702966,0,0
50.1749999999982,0,484.897896702966,0,0
50.1999999999982,1,484.980021702966,0,0
50.2249999999982,2,485.178288991776,0,0
50.2499999999982,0,485.294431280586,0,0
50.2749999999982,0,485.294431280586,0,0
50.2999999999982,0,485.294431280586,0,0
50.3249999999982,0,485.294431280586,0,0
50.3499999999982,0,485.294431280586,0,0
50.3749999999982,0,485.294431280586,0,0
50.3999999999982,0,485.294431280586,0,0
50.4249999999982,1,485.376556280586,0,0
50.4499999999982,0,485.458681280586,0,0
50.4749999999982,0,485.458681280586,0,0
50.4999999999982,0,485.458681280586,0,0
50.5249999999982,0,485.458681280586,0,0
50.5499999999982,0,485.458681280586,0,0
50.5749999999982,0,485.458681280586,0,0
50.5999999999982,0,485.458681280586,0,0
50.6249999999982,0,485.458681280586,0,0

50.6499999999982,1,485.540806280586,0,0
50.6749999999982,1,485.705056280586,0,0
50.6999999999982,0,485.787181280586,0,0
50.7249999999982,1,485.869306280586,0,0
50.7499999999982,1,486.033556280586,0,0
50.7749999999982,1,486.197806280586,0,0
50.7999999999982,1,486.362056280586,0,0
50.8249999999982,1,486.526306280586,0,0
50.8499999999982,1,486.690556280586,0,0
50.8749999999982,1,486.854806280586,0,0
50.8999999999982,0,486.936931280586,0,0
50.9249999999982,1,487.019056280586,0,0
50.9499999999982,1,487.183306280586,0,0
50.9749999999982,1,487.347556280586,0,0
50.9999999999982,1,487.511806280586,0,0
51.0249999999982,1,487.676056280586,0,0
51.0499999999982,1,487.840306280586,0,0
51.0749999999982,1,488.004556280586,0,0
51.0999999999982,1,488.168806280586,0,0
51.1249999999982,1,488.333056280586,0,0
51.1499999999982,1,488.497306280586,0,0
51.1749999999982,1,488.661556280586,0,0
51.1999999999982,1,488.825806280586,0,0
51.2249999999982,2,489.024073569396,0,0
51.2499999999982,1,489.222340858206,0,0
51.2749999999982,1,489.386590858206,0,0
51.2999999999982,1,489.550840858206,0,0
51.3249999999982,1,489.715090858206,0,0
51.3499999999982,1,489.879340858206,0,0
51.3749999999982,0,489.961465858206,0,0
51.3999999999982,0,489.961465858206,0,0
51.4249999999982,1,490.043590858206,0,0
51.4499999999981,1,490.207840858206,0,0
51.4749999999981,1,490.372090858206,0,0
51.4999999999981,1,490.536340858206,0,0
51.5249999999981,1,490.700590858206,0,0
51.5499999999981,2,490.898858147016,0,0
51.5749999999981,2,491.131142724635,0,0
51.5999999999981,2,491.363427302255,0,0
51.6249999999981,1,491.561694591065,0,0
51.6499999999981,2,491.759961879875,0,0
51.6749999999981,2,491.992246457495,0,0
51.6999999999981,2,492.224531035115,0,0
51.7249999999981,2,492.456815612734,0,0
51.7499999999981,2,492.689100190354,0,0
51.7749999999981,3,492.947487151736,0,0
51.7999999999981,4,493.253981824307,0,0
51.8249999999981,3,493.560476496879,0,0
51.8499999999981,3,493.844965842022,0,0
51.8749999999981,3,494.129455187165,0,0
51.8999999999981,3,494.413944532308,0,0
51.9249999999981,3,494.698433877452,0,0

51.9499999999981,3,494.982923222595,0,0
51.9749999999981,3,495.267412567738,0,0
51.9999999999981,4,495.57390724031,0,0
52.0249999999981,4,495.90240724031,0,0
52.0499999999981,4,496.23090724031,0,0
52.0749999999981,4,496.55940724031,0,0
52.0999999999981,4,496.88790724031,0,0
52.1249999999981,5,497.235794322962,0,0
52.1499999999981,5,497.603068488266,0,0
52.1749999999981,4,497.950955570918,0,0
52.1999999999981,4,498.279455570918,0,0
52.2249999999981,4,498.607955570918,0,0
52.2499999999981,5,498.955842653571,0,0
52.2749999999981,6,499.340644081349,0,0
52.2999999999981,5,499.725445509127,0,0
52.3249999999981,4,500.073332591779,0,0
52.3499999999981,5,500.421219674431,0,0
52.3749999999981,5,500.788493839736,0,0
52.3999999999981,5,501.15576800504,0,0
52.4249999999981,5,501.523042170344,0,0
52.4499999999981,6,501.907843598123,0,0
52.4749999999981,7,502.32629026967,0,0
52.4999999999981,9,502.789947596091,0,0
52.5249999999981,9,503.282697596091,0,0
52.5499999999981,7,503.746354922512,0,0
52.5749999999981,7,504.180919575355,0,0
52.5999999999981,8,504.630486479396,0,0
52.6249999999981,7,505.080053383437,0,0
52.6499999999981,6,505.498500054984,0,0
52.6749999999981,3,505.841909072682,0,0
52.6999999999981,7,506.201436071674,0,0
52.7249999999981,7,506.636000724517,0,0
52.7499999999981,7,507.070565377359,0,0
52.7749999999981,6,507.489012048906,0,0
52.7999999999981,11,507.96255420494,19.7124091840037,1
52.8249999999981,5,508.4185690985,19.7124091840037,1
52.8499999999981,9,508.848581181153,19.7124091840037,1
52.8749999999981,9,509.341331181153,19.7124091840037,1
52.8999999999981,9,509.834081181153,19.7124091840037,1
52.9249999999981,9,510.326831181153,19.7124091840037,1
52.9499999999981,11,510.845583992061,19.7124091840037,1
52.9749999999981,9,511.364336802968,19.7124091840037,1
52.9999999999981,10,511.87041385581,19.7124091840037,1
53.0249999999981,7,512.347398235072,19.7124091840037,1
53.0499999999981,10,512.824382614335,19.7124091840037,1
53.0749999999981,10,513.343786720017,19.7124091840037,1
53.0999999999981,9,513.849863772859,19.7124091840037,1
53.1249999999981,9,514.342613772859,19.7124091840037,1
53.1499999999981,9,514.835363772859,19.7124091840037,1
53.1749999999981,10,515.3414408257,19.7124091840037,1
53.199999999998,9,515.847517878541,19.7124091840037,1
53.224999999998,9,516.340267878541,0,0

53.249999999998,8,516.818927456161,0,0
53.274999999998,9,517.297587033781,0,0
53.299999999998,8,517.776246611401,0,0
53.324999999998,8,518.24081576664,0,0
53.349999999998,8,518.70538492188,0,0
53.374999999998,8,519.169954077119,0,0
53.399999999998,8,519.634523232359,0,0
53.424999999998,9,520.113182809979,0,0
53.449999999998,8,520.591842387598,0,0
53.474999999998,8,521.056411542838,0,0
53.499999999998,7,521.505978446879,0,0
53.524999999998,6,521.924425118426,0,0
53.549999999998,5,522.309226546204,0,0
53.574999999998,4,522.657113628857,0,0
53.599999999998,4,522.985613628857,0,0
53.624999999998,4,523.314113628856,0,0
53.649999999998,5,523.662000711509,0,0
53.674999999998,5,524.029274876813,0,0
53.699999999998,6,524.414076304591,0,0
53.724999999998,6,524.816404994843,0,0
53.749999999998,5,525.201206422622,0,0
53.774999999998,5,525.568480587926,0,0
53.799999999998,5,525.93575475323,0,0
53.824999999998,5,526.303028918535,0,0
53.849999999998,5,526.670303083839,0,0
53.874999999998,5,527.037577249144,0,0
53.899999999998,4,527.385464331796,0,0
53.924999999998,4,527.713964331796,0,0
53.949999999998,4,528.042464331796,0,0
53.974999999998,4,528.370964331796,0,0
53.999999999998,7,528.752496658217,0,0
54.024999999998,9,529.216153984638,0,0
54.049999999998,11,529.734906795546,20.9861763959241,1
54.074999999998,12,530.291773951597,20.9861763959241,1
54.099999999998,12,530.860752641883,20.9861763959241,1
54.124999999998,10,531.404944039868,20.9861763959241,1
54.149999999998,10,531.92434814555,20.9861763959241,1
54.174999999998,10,532.443752251233,20.9861763959241,1
54.199999999998,10,532.963156356916,20.9861763959241,1
54.224999999998,10,533.482560462598,20.9861763959241,1
54.249999999998,9,533.98863751544,20.9861763959241,1
54.274999999998,10,534.494714568281,20.9861763959241,1
54.299999999998,10,535.014118673963,20.9861763959241,1
54.324999999998,8,535.506105304425,20.9861763959241,1
54.349999999998,8,535.970674459664,0,0
54.374999999998,7,536.420241363705,0,0
54.399999999998,7,536.854806016548,0,0
54.424999999998,6,537.273252688095,0,0
54.449999999998,5,537.658054115873,0,0
54.474999999998,4,538.005941198525,0,0
54.499999999998,4,538.334441198525,0,0
54.524999999998,4,538.662941198525,0,0

54.549999999998,4,538.991441198525,0,0
54.574999999998,3,539.297935871097,0,0
54.599999999998,3,539.58242521624,0,0
54.624999999998,3,539.866914561383,0,0
54.649999999998,2,540.125301522765,0,0
54.674999999998,2,540.357586100384,0,0
54.699999999998,2,540.589870678004,0,0
54.724999999998,1,540.788137966814,0,0
54.749999999998,0,540.870262966814,0,0
54.774999999998,1,540.952387966814,0,0
54.799999999998,1,541.116637966814,0,0
54.824999999998,1,541.280887966814,0,0
54.849999999998,1,541.445137966814,0,0
54.874999999998,1,541.609387966814,0,0
54.899999999998,0,541.691512966814,0,0
54.924999999998,0,541.691512966814,0,0
54.9499999999979,0,541.691512966814,0,0
54.9749999999979,0,541.691512966814,0,0
54.9999999999979,0,541.691512966814,0,0
55.0249999999979,0,541.691512966814,0,0
55.0499999999979,0,541.691512966814,0,0
55.0749999999979,1,541.773637966814,0,0
55.0999999999979,0,541.855762966814,0,0
55.1249999999979,0,541.855762966814,0,0
55.1499999999979,0,541.855762966814,0,0
55.1749999999979,0,541.855762966814,0,0
55.1999999999979,0,541.855762966814,0,0
55.2249999999979,1,541.937887966814,0,0
55.2499999999979,1,542.102137966814,0,0
55.2749999999979,1,542.266387966814,0,0
55.2999999999979,1,542.430637966815,0,0
55.3249999999979,1,542.594887966815,0,0
55.3499999999979,1,542.759137966815,0,0
55.3749999999979,0,542.841262966815,0,0
55.3999999999979,1,542.923387966815,0,0
55.4249999999979,1,543.087637966815,0,0
55.4499999999979,1,543.251887966815,0,0
55.4749999999979,0,543.334012966815,0,0
55.4999999999979,0,543.334012966815,0,0
55.5249999999979,0,543.334012966815,0,0
55.5499999999979,0,543.334012966815,0,0
55.5749999999979,1,543.416137966815,0,0
55.5999999999979,1,543.580387966815,0,0
55.6249999999979,0,543.662512966815,0,0
55.6499999999979,0,543.662512966815,0,0
55.6749999999979,0,543.662512966815,0,0
55.6999999999979,0,543.662512966815,0,0
55.7249999999979,1,543.744637966815,0,0
55.7499999999979,1,543.908887966815,0,0
55.7749999999979,1,544.073137966815,0,0
55.7999999999979,1,544.237387966815,0,0
55.8249999999979,1,544.401637966815,0,0

55.8499999999979,1,544.565887966815,0,0
55.8749999999979,1,544.730137966815,0,0
55.8999999999979,1,544.894387966815,0,0
55.9249999999979,1,545.058637966815,0,0
55.9499999999979,1,545.222887966815,0,0
55.9749999999979,0,545.305012966815,0,0
55.9999999999979,1,545.387137966815,0,0
56.0249999999979,0,545.469262966815,0,0
56.0499999999979,1,545.551387966815,0,0
56.0749999999979,0,545.633512966815,0,0
56.0999999999979,0,545.633512966815,0,0
56.1249999999979,0,545.633512966815,0,0
56.1499999999979,0,545.633512966815,0,0
56.1749999999979,0,545.633512966815,0,0
56.1999999999979,0,545.633512966815,0,0
56.2249999999979,2,545.749655255625,0,0
56.2499999999979,10,546.125499597276,39.7264086601985,1
56.2749999999979,18,546.733628516547,39.7264086601985,1
56.2999999999979,23,547.475913046829,39.7264086601985,1
56.3249999999979,27,548.296504728396,39.7264086601985,1
56.3499999999979,32,549.18780790135,39.7264086601985,1
56.3749999999979,35,550.138235108777,39.7264086601985,1
56.3999999999979,29,551.06634982075,39.7264086601985,1
56.4249999999979,29,551.950863140322,39.7264086601985,1
56.4499999999979,31,552.850372448405,39.7264086601985,1
56.4749999999979,34,553.786492021067,39.7264086601985,1
56.4999999999979,34,554.744225869795,39.7264086601985,1
56.5249999999979,26,555.641849771713,39.7264086601985,1
56.5499999999979,30,556.510423899618,39.7264086601985,1
56.5749999999979,30,557.41005820032,39.7264086601985,1
56.5999999999979,37,558.359422223472,39.7264086601985,1
56.6249999999979,44,559.403724718089,39.7264086601985,1
56.6499999999979,47,560.511500973963,39.7264086601985,1
56.6749999999979,53,561.672400632678,39.7264086601985,1
56.6999999999979,53,562.868158681992,39.7264086601985,1
56.7249999999978,55,564.075093007402,39.7264086601985,1
56.7499999999978,54,565.287641343533,39.7264086601985,1
56.7749999999978,55,566.500189679664,39.7264086601985,1
56.7999999999978,60,567.745382495031,39.7264086601985,1
56.8249999999978,60,569.01765752426,39.7264086601985,1
56.8499999999978,62,570.300447935528,39.7264086601985,1
56.8749999999978,60,571.583238346796,39.7264086601985,1
56.8999999999978,61,572.860792616044,39.7264086601985,1
56.9249999999978,65,574.164322288257,39.7264086601985,1
56.9499999999978,67,575.498657302226,39.7264086601985,1
56.9749999999978,66,576.838066052597,39.7264086601985,1
56.9999999999978,71,578.1972502567,39.7264086601985,1
57.0249999999978,75,579.60047116968,39.7264086601985,1
57.0499999999978,82,581.055368037007,39.7264086601985,1
57.0749999999978,81,582.538166541477,39.7264086601985,1
57.0999999999978,79,584.007234507999,39.7264086601985,1
57.1249999999978,74,585.443643437077,39.7264086601985,1

57.1499999999978,75,586.861332762491,39.7264086601985,1
57.1749999999978,68,588.249776224356,39.7264086601985,1
57.1999999999978,67,589.599218419754,39.7264086601985,1
57.2249999999978,63,590.923287495408,39.7264086601985,1
57.2499999999978,63,592.226981453935,39.7264086601985,1
57.2749999999978,57,593.498858586195,39.7264086601985,1
57.2999999999978,53,594.716767763849,39.7264086601985,1
57.3249999999978,50,595.895358232555,39.7264086601985,1
57.3499999999978,48,597.045048366891,39.7264086601985,1
57.3749999999978,41,598.139883635177,39.7264086601985,1
57.3999999999978,39,599.178610673794,39.7264086601985,1
57.4249999999978,36,600.184231134413,39.7264086601985,1
57.4499999999978,33,601.14875334176,39.7264086601985,1
57.4749999999978,30,602.070342699458,39.7264086601985,1
57.4999999999978,28,602.954724502651,39.7264086601985,1
57.5249999999978,26,603.808046133047,39.7264086601985,1
57.5499999999978,26,604.645560088155,39.7264086601985,1
57.5749999999978,23,605.458174729561,39.7264086601985,1
57.5999999999978,21,606.228376422361,39.7264086601985,1
57.6249999999978,21,606.981064480258,39.7264086601985,1
57.6499999999978,19,607.715383084944,39.7264086601985,1
57.6749999999978,18,608.421784527112,39.7264086601985,1
57.6999999999978,15,609.088280150849,39.7264086601985,1
57.7249999999978,13,609.702454806654,39.7264086601985,1
57.7499999999978,13,610.294666603649,39.7264086601985,1
57.7749999999978,13,610.886878400644,39.7264086601985,1
57.7999999999978,11,611.455362110049,39.7264086601985,1
57.8249999999978,10,611.987441973799,39.7264086601985,1
57.8499999999978,10,612.506846079481,39.7264086601985,1
57.8749999999978,9,613.012923132323,39.7264086601985,1
57.8999999999978,9,613.505673132323,39.7264086601985,1
57.9249999999978,9,613.998423132323,39.7264086601985,1
57.9499999999978,15,614.56286688963,39.7264086601985,1
57.9749999999978,10,615.140637699778,39.7264086601985,1
57.9999999999978,9,615.64671475262,39.7264086601985,1
58.0249999999978,9,616.13946475262,0,0
58.0499999999978,7,616.603122079041,0,0
58.0749999999978,6,617.021568750588,0,0
58.0999999999978,6,617.42389744084,0,0
58.1249999999978,5,617.808698868619,0,0
58.1499999999978,4,618.156585951271,0,0
58.1749999999978,2,618.436978240081,0,0
58.1999999999978,2,618.669262817701,0,0
58.2249999999978,2,618.90154739532,0,0
58.2499999999978,1,619.09981468413,0,0
58.2749999999978,1,619.26406468413,0,0
58.2999999999978,0,619.34618968413,0,0
58.3249999999978,0,619.34618968413,0,0
58.3499999999978,0,619.34618968413,0,0
58.3749999999978,0,619.34618968413,0,0
58.3999999999978,1,619.42831468413,0,0
58.4249999999978,1,619.59256468413,0,0

58.4499999999978,0,619.67468968413,0,0
58.4749999999977,1,619.75681468413,0,0
58.4999999999977,1,619.92106468413,0,0
58.5249999999977,1,620.08531468413,0,0
58.5499999999977,0,620.167439684131,0,0
58.5749999999977,0,620.167439684131,0,0
58.5999999999977,0,620.167439684131,0,0
58.6249999999977,0,620.167439684131,0,0
58.6499999999977,0,620.167439684131,0,0
58.6749999999977,0,620.167439684131,0,0
58.6999999999977,0,620.167439684131,0,0
58.7249999999977,1,620.249564684131,0,0
58.7499999999977,0,620.331689684131,0,0
58.7749999999977,0,620.331689684131,0,0
58.7999999999977,0,620.331689684131,0,0
58.8249999999977,0,620.331689684131,0,0
58.8499999999977,0,620.331689684131,0,0
58.8749999999977,0,620.331689684131,0,0
58.8999999999977,0,620.331689684131,0,0
58.9249999999977,0,620.331689684131,0,0
58.9499999999977,0,620.331689684131,0,0
58.9749999999977,0,620.331689684131,0,0
58.9999999999977,0,620.331689684131,0,0
59.0249999999977,0,620.331689684131,0,0
59.0499999999977,0,620.331689684131,0,0
59.0749999999977,0,620.331689684131,0,0
59.0999999999977,0,620.331689684131,0,0
59.1249999999977,1,620.413814684131,0,0
59.1499999999977,0,620.495939684131,0,0
59.1749999999977,0,620.495939684131,0,0
59.1999999999977,0,620.495939684131,0,0
59.2249999999977,1,620.578064684131,0,0
59.2499999999977,1,620.742314684131,0,0
59.2749999999977,0,620.824439684131,0,0
59.2999999999977,1,620.906564684131,0,0
59.3249999999977,1,621.070814684131,0,0
59.3499999999977,1,621.235064684131,0,0
59.3749999999977,0,621.317189684131,0,0
59.3999999999977,0,621.317189684131,0,0
59.4249999999977,1,621.399314684131,0,0
59.4499999999977,0,621.481439684131,0,0
59.4749999999977,0,621.481439684131,0,0
59.4999999999977,0,621.481439684131,0,0
59.5249999999977,0,621.481439684131,0,0
59.5499999999977,0,621.481439684131,0,0
59.5749999999977,0,621.481439684131,0,0
59.5999999999977,0,621.481439684131,0,0
59.6249999999977,0,621.481439684131,0,0
59.6499999999977,0,621.481439684131,0,0
59.6749999999977,0,621.481439684131,0,0
59.6999999999977,0,621.481439684131,0,0
59.7249999999977,0,621.481439684131,0,0

59.7499999999977,0,621.481439684131,0,0
59.7749999999977,1,621.563564684131,0,0
59.7999999999977,0,621.645689684131,0,0
59.8249999999977,0,621.645689684131,0,0
59.8499999999977,0,621.645689684131,0,0
59.8749999999977,0,621.645689684131,0,0
59.8999999999977,0,621.645689684131,0,0
59.9249999999977,0,621.645689684131,0,0
59.9499999999977,0,621.645689684131,0,0
59.9749999999977,1,621.727814684131,0,0
59.9999999999977,0,621.809939684131,0,0
60.0249999999977,1,621.892064684131,0,0
60.0499999999977,0,621.974189684131,0,0
60.0749999999977,0,621.974189684131,0,0
60.0999999999977,0,621.974189684131,0,0
60.1249999999977,1,622.056314684131,0,0
60.1499999999977,0,622.138439684131,0,0
60.1749999999977,0,622.138439684131,0,0
60.1999999999977,1,622.220564684131,0,0
60.2249999999977,0,622.302689684131,0,0
60.2499999999976,0,622.302689684131,0,0
60.2749999999976,6,622.503854029257,0,0
60.2999999999976,0,622.705018374383,0,0
60.3249999999976,1,622.787143374383,0,0
60.3499999999976,0,622.869268374383,0,0
60.3749999999976,4,623.033518374383,0,0
60.3999999999976,0,623.197768374383,0,0
60.4249999999976,2,623.313910663193,0,0
60.4499999999976,0,623.430052952003,0,0
60.4749999999976,0,623.430052952003,0,0
60.4999999999976,0,623.430052952003,0,0
60.5249999999976,0,623.430052952003,0,0
60.5499999999976,0,623.430052952003,0,0
60.5749999999976,0,623.430052952003,0,0
60.5999999999976,1,623.512177952003,0,0
60.6249999999976,0,623.594302952003,0,0
60.6499999999976,0,623.594302952003,0,0
60.6749999999976,0,623.594302952003,0,0
60.6999999999976,0,623.594302952003,0,0
60.7249999999976,0,623.594302952003,0,0
60.7499999999976,0,623.594302952003,0,0
60.7749999999976,0,623.594302952003,0,0
60.7999999999976,0,623.594302952003,0,0
60.8249999999976,0,623.594302952003,0,0
60.8499999999976,0,623.594302952003,0,0
60.8749999999976,1,623.676427952003,0,0
60.8999999999976,0,623.758552952003,0,0
60.9249999999976,0,623.758552952003,0,0
60.9499999999976,1,623.840677952003,0,0
60.9749999999976,0,623.922802952003,0,0
60.9999999999976,1,624.004927952003,0,0
61.0249999999976,1,624.169177952003,0,0

61.0499999999976,0,624.251302952003,0,0
61.0749999999976,0,624.251302952003,0,0
61.0999999999976,1,624.333427952003,0,0
61.1249999999976,0,624.415552952003,0,0
61.1499999999976,0,624.415552952003,0,0
61.1749999999976,0,624.415552952003,0,0
61.1999999999976,1,624.497677952003,0,0
61.2249999999976,0,624.579802952003,0,0
61.2499999999976,0,624.579802952003,0,0
61.2749999999976,1,624.661927952003,0,0
61.2999999999976,0,624.744052952003,0,0
61.3249999999976,0,624.744052952003,0,0
61.3499999999976,0,624.744052952003,0,0
61.3749999999976,0,624.744052952003,0,0
61.3999999999976,0,624.744052952003,0,0
61.4249999999976,0,624.744052952003,0,0
61.4499999999976,1,624.826177952003,0,0
61.4749999999976,0,624.908302952003,0,0
61.4999999999976,1,624.990427952003,0,0
61.5249999999976,0,625.072552952003,0,0
61.5499999999976,0,625.072552952003,0,0
61.5749999999976,0,625.072552952003,0,0
61.5999999999976,0,625.072552952003,0,0
61.6249999999976,1,625.154677952003,0,0
61.6499999999976,0,625.236802952003,0,0
61.6749999999976,0,625.236802952003,0,0
61.6999999999976,0,625.236802952003,0,0
61.7249999999976,0,625.236802952003,0,0
61.7499999999976,0,625.236802952003,0,0
61.7749999999976,1,625.318927952003,0,0
61.7999999999976,0,625.401052952003,0,0
61.8249999999976,0,625.401052952003,0,0
61.8499999999976,0,625.401052952003,0,0
61.8749999999976,0,625.401052952003,0,0
61.8999999999976,0,625.401052952003,0,0
61.9249999999976,0,625.401052952003,0,0
61.9499999999976,0,625.401052952003,0,0
61.9749999999976,0,625.401052952003,0,0
61.9999999999975,0,625.401052952003,0,0
62.0249999999975,0,625.401052952003,0,0
62.0499999999975,0,625.401052952003,0,0
62.0749999999975,0,625.401052952003,0,0
62.0999999999975,1,625.483177952003,0,0
62.1249999999975,0,625.565302952003,0,0
62.1499999999975,0,625.565302952003,0,0
62.1749999999975,0,625.565302952003,0,0
62.1999999999975,0,625.565302952003,0,0
62.2249999999975,0,625.565302952003,0,0
62.2499999999975,0,625.565302952003,0,0
62.2749999999975,0,625.565302952003,0,0
62.2999999999975,0,625.565302952003,0,0
62.3249999999975,0,625.565302952003,0,0

62.3499999999975,0,625.565302952003,0,0
62.3749999999975,0,625.565302952003,0,0
62.3999999999975,0,625.565302952003,0,0
62.4249999999975,0,625.565302952003,0,0
62.4499999999975,0,625.565302952003,0,0
62.4749999999975,0,625.565302952003,0,0
62.4999999999975,0,625.565302952003,0,0
62.5249999999975,0,625.565302952003,0,0
62.5499999999975,0,625.565302952003,0,0
62.5749999999975,0,625.565302952003,0,0
62.5999999999975,0,625.565302952003,0,0
62.6249999999975,0,625.565302952003,0,0
62.6499999999975,0,625.565302952003,0,0
62.6749999999975,0,625.565302952003,0,0
62.6999999999975,0,625.565302952003,0,0
62.7249999999975,1,625.647427952003,0,0
62.7499999999975,0,625.729552952003,0,0
62.7749999999975,0,625.729552952003,0,0
62.7999999999975,0,625.729552952003,0,0
62.8249999999975,0,625.729552952003,0,0
62.8499999999975,0,625.729552952003,0,0
62.8749999999975,1,625.811677952003,0,0
62.8999999999975,0,625.893802952004,0,0
62.9249999999975,0,625.893802952004,0,0
62.9499999999975,0,625.893802952004,0,0
62.9749999999975,0,625.893802952004,0,0
62.9999999999975,0,625.893802952004,0,0
63.0249999999975,0,625.893802952004,0,0
63.0499999999975,0,625.893802952004,0,0
63.0749999999975,0,625.893802952004,0,0
63.0999999999975,0,625.893802952004,0,0
63.1249999999975,0,625.893802952004,0,0
63.1499999999975,0,625.893802952004,0,0
63.1749999999975,0,625.893802952004,0,0
63.1999999999975,0,625.893802952004,0,0
63.2249999999975,0,625.893802952004,0,0
63.2499999999975,0,625.893802952004,0,0
63.2749999999975,0,625.893802952004,0,0
63.2999999999975,0,625.893802952004,0,0
63.3249999999975,0,625.893802952004,0,0
63.3499999999975,1,625.975927952004,0,0
63.3749999999975,0,626.058052952004,0,0
63.3999999999975,1,626.140177952004,0,0
63.4249999999975,1,626.304427952004,0,0
63.4499999999975,1,626.468677952004,0,0
63.4749999999975,1,626.632927952004,0,0
63.4999999999975,0,626.715052952004,0,0
63.5249999999975,0,626.715052952004,0,0
63.5499999999975,0,626.715052952004,0,0
63.5749999999975,0,626.715052952004,0,0
63.5999999999975,0,626.715052952004,0,0
63.6249999999975,0,626.715052952004,0,0

```
63.6499999999975,0,626.715052952004,0,0
63.6749999999975,0,626.715052952004,0,0
63.6999999999975,0,626.715052952004,0,0
63.7249999999975,0,626.715052952004,0,0
63.7499999999974,0,626.715052952004,0,0
63.7749999999974,0,626.715052952004,0,0
63.7999999999974,0,626.715052952004,0,0
63.8249999999974,1,626.797177952004,0,0
63.8499999999974,1,626.961427952004,0,0
63.8749999999974,0,627.043552952004,0,0
63.8999999999974,5,627.227190034656,0,0
63.9249999999974,6,627.611991462434,0,0
63.9499999999974,1,627.89528080756,0,0
63.9749999999974,1,628.05953080756,0,0
63.9999999999974,4,628.30590580756,0,0
64.0249999999974,4,628.63440580756,0,0
64.0499999999974,0,628.79865580756,0,0
64.0749999999974,1,628.88078080756,0,0
64.0999999999975,0,628.96290580756,0,0
64.1249999999975,0,628.96290580756,0,0
64.1499999999975,5,629.146542890212,0,0
64.1749999999975,0,629.330179972864,0,0
64.1999999999975,1,629.412304972864,0,0
64.2249999999975,0,629.494429972865,0,0
64.2499999999975,1,629.576554972865,0,0
64.2749999999975,0,629.658679972865,0,0
64.2999999999975,1,629.740804972865,0,0
64.3249999999975,0,629.822929972865,0,0
64.3499999999975,1,629.905054972865,0,0
64.3749999999975,1,630.069304972865,0,0
64.3999999999975,1,630.233554972865,0,0
64.4249999999975,0,630.315679972865,0,0
64.4499999999975,0,630.315679972865,0,0
64.4749999999975,0,630.315679972865,0,0
64.4999999999975,0,630.315679972865,0,0
64.5249999999975,0,630.315679972865,0,0
64.5499999999976,0,630.315679972865,0,0
64.5749999999976,0,630.315679972865,0,0
64.5999999999976,0,630.315679972865,0,0
64.6249999999976,0,630.315679972865,0,0
64.6499999999976,1,630.397804972865,0,0
64.6749999999976,0,630.479929972865,0,0
64.6999999999976,0,630.479929972865,0,0
64.7249999999976,0,630.479929972865,0,0
64.7499999999976,0,630.479929972865,0,0
64.7749999999976,0,630.479929972865,0,0
64.7999999999976,0,630.479929972865,0,0
64.8249999999976,0,630.479929972865,0,0
64.8499999999976,0,630.479929972865,0,0
64.8749999999976,0,630.479929972865,0,0
64.8999999999976,0,630.479929972865,0,0
64.9249999999976,0,630.479929972865,0,0
```

```
64.9499999999976,0,630.479929972865,0,0
64.9749999999977,0,630.479929972865,0,0
64.9999999999977,0,630.479929972865,0,0
65.0249999999977,0,630.479929972865,0,0
65.0499999999977,0,630.479929972865,0,0
65.0749999999977,0,630.479929972865,0,0
65.0999999999977,0,630.479929972865,0,0
65.1249999999977,0,630.479929972865,0,0
65.1499999999977,1,630.562054972865,0,0
65.1749999999977,0,630.644179972865,0,0
65.1999999999977,0,630.644179972865,0,0
65.2249999999977,1,630.726304972865,0,0
65.2499999999977,0,630.808429972865,0,0
65.2749999999977,0,630.808429972865,0,0
65.2999999999977,0,630.808429972865,0,0
65.3249999999977,0,630.808429972865,0,0
65.3499999999977,1,630.890554972865,0,0
65.3749999999977,0,630.972679972865,0,0
65.3999999999977,0,630.972679972865,0,0
65.4249999999978,0,630.972679972865,0,0
65.4499999999978,1,631.054804972865,0,0
65.4749999999978,0,631.136929972865,0,0
65.4999999999978,1,631.219054972865,0,0
65.5249999999978,0,631.301179972865,0,0
65.5499999999978,1,631.383304972865,0,0
65.5749999999978,1,631.547554972865,0,0
65.5999999999978,0,631.629679972865,0,0
65.6249999999978,1,631.711804972865,0,0
65.6499999999978,1,631.876054972865,0,0
65.6749999999978,0,631.958179972865,0,0
65.6999999999978,1,632.040304972865,0,0
65.7249999999978,0,632.122429972865,0,0
65.7499999999978,0,632.122429972865,0,0
65.7749999999978,0,632.122429972865,0,0
65.7999999999978,1,632.204554972865,0,0
65.8249999999978,0,632.286679972865,0,0
65.8499999999978,0,632.286679972865,0,0
65.8749999999979,0,632.286679972865,0,0
65.8999999999979,0,632.286679972865,0,0
65.9249999999979,0,632.286679972865,0,0
65.9499999999979,0,632.286679972865,0,0
65.9749999999979,0,632.286679972865,0,0
65.9999999999979,0,632.286679972865,0,0
66.0249999999979,0,632.286679972865,0,0
66.0499999999979,0,632.286679972865,0,0
66.0749999999979,0,632.286679972865,0,0
66.0999999999979,0,632.286679972865,0,0
66.1249999999979,1,632.368804972865,0,0
66.1499999999979,0,632.450929972865,0,0
66.1749999999979,1,632.533054972865,0,0
66.1999999999979,1,632.697304972865,0,0
66.2249999999979,1,632.861554972865,0,0
```

66.2499999999979,0,632.943679972865,0,0
66.2749999999979,0,632.943679972865,0,0
66.299999999998,0,632.943679972865,0,0
66.324999999998,0,632.943679972865,0,0
66.349999999998,0,632.943679972865,0,0
66.374999999998,0,632.943679972865,0,0
66.399999999998,0,632.943679972865,0,0
66.424999999998,0,632.943679972865,0,0
66.449999999998,0,632.943679972865,0,0
66.474999999998,0,632.943679972865,0,0
66.499999999998,0,632.943679972865,0,0
66.524999999998,0,632.943679972865,0,0
66.549999999998,0,632.943679972865,0,0
66.574999999998,0,632.943679972865,0,0
66.599999999998,1,633.025804972865,0,0
66.624999999998,1,633.190054972865,0,0
66.649999999998,0,633.272179972865,0,0
66.674999999998,0,633.272179972865,0,0
66.699999999998,0,633.272179972865,0,0
66.724999999998,1,633.354304972865,0,0
66.7499999999981,0,633.436429972865,0,0
66.7749999999981,0,633.436429972865,0,0
66.7999999999981,0,633.436429972865,0,0
66.8249999999981,0,633.436429972865,0,0
66.8499999999981,0,633.436429972865,0,0
66.8749999999981,0,633.436429972865,0,0
66.8999999999981,0,633.436429972865,0,0
66.9249999999981,0,633.436429972865,0,0
66.9499999999981,0,633.436429972865,0,0
66.9749999999981,0,633.436429972865,0,0
66.9999999999981,0,633.436429972865,0,0
67.0249999999981,0,633.436429972865,0,0
67.0499999999981,0,633.436429972865,0,0
67.0749999999981,0,633.436429972865,0,0
67.0999999999981,0,633.436429972865,0,0
67.1249999999981,0,633.436429972865,0,0
67.1499999999981,0,633.436429972865,0,0
67.1749999999982,0,633.436429972865,0,0
67.1999999999982,0,633.436429972865,0,0
67.2249999999982,0,633.436429972865,0,0
67.2499999999982,0,633.436429972865,0,0
67.2749999999982,1,633.518554972865,0,0
67.2999999999982,0,633.600679972865,0,0
67.3249999999982,0,633.600679972865,0,0
67.3499999999982,0,633.600679972865,0,0
67.3749999999982,0,633.600679972865,0,0
67.3999999999982,1,633.682804972865,0,0
67.4249999999982,0,633.764929972866,0,0
67.4499999999982,0,633.764929972866,0,0
67.4749999999982,0,633.764929972866,0,0
67.4999999999982,1,633.847054972866,0,0
67.5249999999982,0,633.929179972866,0,0

67.5499999999982,1,634.011304972866,0,0
67.5749999999982,1,634.175554972866,0,0
67.5999999999982,0,634.257679972866,0,0
67.6249999999983,3,634.399924645437,0,0
67.6499999999983,3,634.68441399058,0,0
67.6749999999983,1,634.908783663152,0,0
67.6999999999983,0,634.990908663152,0,0
67.7249999999983,2,635.107050951962,0,0
67.7499999999983,3,635.365437913343,0,0
67.7749999999983,0,635.507682585915,0,0
67.7999999999983,0,635.507682585915,0,0
67.8249999999983,0,635.507682585915,0,0
67.8499999999983,0,635.507682585915,0,0
67.8749999999983,0,635.507682585915,0,0
67.8999999999983,0,635.507682585915,0,0
67.9249999999983,0,635.507682585915,0,0
67.9499999999983,0,635.507682585915,0,0
67.9749999999983,0,635.507682585915,0,0
67.9999999999983,0,635.507682585915,0,0
68.0249999999983,0,635.507682585915,0,0
68.0499999999983,0,635.507682585915,0,0
68.0749999999984,0,635.507682585915,0,0
68.0999999999984,0,635.507682585915,0,0
68.1249999999984,0,635.507682585915,0,0
68.1499999999984,0,635.507682585915,0,0
68.1749999999984,0,635.507682585915,0,0
68.1999999999984,0,635.507682585915,0,0
68.2249999999984,0,635.507682585915,0,0
68.2499999999984,0,635.507682585915,0,0
68.2749999999984,0,635.507682585915,0,0
68.2999999999984,0,635.507682585915,0,0
68.3249999999984,1,635.589807585915,0,0
68.3499999999984,1,635.754057585915,0,0
68.3749999999984,0,635.836182585915,0,0
68.3999999999984,1,635.918307585915,0,0
68.4249999999984,0,636.000432585915,0,0
68.4499999999984,0,636.000432585915,0,0
68.4749999999984,1,636.082557585915,0,0
68.4999999999985,0,636.164682585915,0,0
68.5249999999985,0,636.164682585915,0,0
68.5499999999985,0,636.164682585915,0,0
68.5749999999985,1,636.246807585915,0,0
68.5999999999985,0,636.328932585915,0,0
68.6249999999985,1,636.411057585915,0,0
68.6499999999985,1,636.575307585915,0,0
68.6749999999985,0,636.657432585915,0,0
68.6999999999985,0,636.657432585915,0,0
68.7249999999985,1,636.739557585915,0,0
68.7499999999985,1,636.903807585915,0,0
68.7749999999985,0,636.985932585915,0,0
68.7999999999985,1,637.068057585915,0,0
68.8249999999985,0,637.150182585915,0,0

68.8499999999985,1,637.232307585915,0,0
68.8749999999985,1,637.396557585915,0,0
68.8999999999985,1,637.560807585915,0,0
68.9249999999985,1,637.725057585915,0,0
68.9499999999986,0,637.807182585915,0,0
68.9749999999986,0,637.807182585915,0,0
68.9999999999986,1,637.889307585916,0,0
69.0249999999986,0,637.971432585916,0,0
69.0499999999986,0,637.971432585916,0,0
69.0749999999986,1,638.053557585916,0,0
69.0999999999986,1,638.217807585916,0,0
69.1249999999986,0,638.299932585916,0,0
69.1499999999986,1,638.382057585916,0,0
69.1749999999986,0,638.464182585916,0,0
69.1999999999986,1,638.546307585916,0,0
69.2249999999986,0,638.628432585916,0,0
69.2499999999986,0,638.628432585916,0,0
69.2749999999986,0,638.628432585916,0,0
69.2999999999986,0,638.628432585916,0,0
69.3249999999986,0,638.628432585916,0,0
69.3499999999986,0,638.628432585916,0,0
69.3749999999987,0,638.628432585916,0,0
69.3999999999987,1,638.710557585916,0,0
69.4249999999987,0,638.792682585916,0,0
69.4499999999987,1,638.874807585916,0,0
69.4749999999987,1,639.039057585916,0,0
69.4999999999987,0,639.121182585916,0,0
69.5249999999987,0,639.121182585916,0,0
69.5499999999987,0,639.121182585916,0,0
69.5749999999987,1,639.203307585916,0,0
69.5999999999987,1,639.367557585916,0,0
69.6249999999987,1,639.531807585916,0,0
69.6499999999987,1,639.696057585916,0,0
69.6749999999987,0,639.778182585916,0,0
69.6999999999987,0,639.778182585916,0,0
69.7249999999987,0,639.778182585916,0,0
69.7499999999987,0,639.778182585916,0,0
69.7749999999987,0,639.778182585916,0,0
69.7999999999987,0,639.778182585916,0,0
69.8249999999988,1,639.860307585916,0,0
69.8499999999988,0,639.942432585916,0,0
69.8749999999988,0,639.942432585916,0,0
69.8999999999988,1,640.024557585916,0,0
69.9249999999988,0,640.106682585916,0,0
69.9499999999988,0,640.106682585916,0,0
69.9749999999988,0,640.106682585916,0,0
69.9999999999988,0,640.106682585916,0,0
70.0249999999988,0,640.106682585916,0,0
70.0499999999988,0,640.106682585916,0,0
70.0749999999988,0,640.106682585916,0,0
70.0999999999988,0,640.106682585916,0,0
70.1249999999988,0,640.106682585916,0,0

70.1499999999988,0,640.106682585916,0,0
70.1749999999988,0,640.106682585916,0,0
70.1999999999988,0,640.106682585916,0,0
70.2249999999988,0,640.106682585916,0,0
70.2499999999988,0,640.106682585916,0,0
70.2749999999989,0,640.106682585916,0,0
70.2999999999989,0,640.106682585916,0,0
70.3249999999989,0,640.106682585916,0,0
70.3499999999989,0,640.106682585916,0,0
70.3749999999989,0,640.106682585916,0,0
70.3999999999989,0,640.106682585916,0,0
70.4249999999989,0,640.106682585916,0,0
70.4499999999989,0,640.106682585916,0,0
70.4749999999989,0,640.106682585916,0,0
70.4999999999989,1,640.188807585916,0,0
70.5249999999989,0,640.270932585916,0,0
70.5499999999989,1,640.353057585916,0,0
70.5749999999989,0,640.435182585916,0,0
70.5999999999989,0,640.435182585916,0,0
70.6249999999989,1,640.517307585916,0,0
70.6499999999989,0,640.599432585916,0,0
70.6749999999989,1,640.681557585916,0,0
70.699999999999,1,640.845807585916,0,0
70.724999999999,0,640.927932585916,0,0
70.749999999999,1,641.010057585916,0,0
70.774999999999,1,641.174307585916,0,0
70.799999999999,1,641.338557585916,0,0
70.824999999999,0,641.420682585916,0,0
70.849999999999,1,641.502807585916,0,0
70.874999999999,0,641.584932585916,0,0
70.899999999999,0,641.584932585916,0,0
70.924999999999,0,641.584932585916,0,0
70.949999999999,0,641.584932585916,0,0
70.974999999999,0,641.584932585916,0,0
70.999999999999,0,641.584932585916,0,0
71.024999999999,0,641.584932585916,0,0
71.049999999999,0,641.584932585916,0,0
71.074999999999,0,641.584932585916,0,0
71.099999999999,0,641.584932585916,0,0
71.124999999999,0,641.584932585916,0,0
71.1499999999991,1,641.667057585916,0,0
71.1749999999991,0,641.749182585916,0,0
71.1999999999991,0,641.749182585916,0,0
71.2249999999991,0,641.749182585916,0,0
71.2499999999991,0,641.749182585916,0,0
71.2749999999991,0,641.749182585916,0,0
71.2999999999991,0,641.749182585916,0,0
71.3249999999991,1,641.831307585916,0,0
71.3499999999991,0,641.913432585916,0,0
71.3749999999991,0,641.913432585916,0,0
71.3999999999991,0,641.913432585916,0,0
71.4249999999991,0,641.913432585916,0,0

71.4499999999991,3,642.055677258488,0,0
71.4749999999991,4,642.36217193106,0,0
71.4999999999991,0,642.52642193106,0,0
71.5249999999991,0,642.52642193106,0,0
71.5499999999991,0,642.52642193106,0,0
71.5749999999992,0,642.52642193106,0,0
71.5999999999992,0,642.52642193106,0,0
71.6249999999992,0,642.52642193106,0,0
71.6499999999992,2,642.64256421987,0,0
71.6749999999992,5,642.942343591332,0,0
71.6999999999992,0,643.125980673984,0,0
71.7249999999992,0,643.125980673984,0,0
71.7499999999992,0,643.125980673984,0,0
71.7749999999992,0,643.125980673984,0,0
71.7999999999992,0,643.125980673984,0,0
71.8249999999992,0,643.125980673984,0,0
71.8499999999992,0,643.125980673984,0,0
71.8749999999992,0,643.125980673984,0,0
71.8999999999992,0,643.125980673984,0,0
71.9249999999992,0,643.125980673984,0,0
71.9499999999992,0,643.125980673984,0,0
71.9749999999992,1,643.208105673984,0,0
71.9999999999992,0,643.290230673984,0,0
72.0249999999993,0,643.290230673984,0,0
72.0499999999993,0,643.290230673984,0,0
72.0749999999993,1,643.372355673984,0,0
72.0999999999993,0,643.454480673984,0,0
72.1249999999993,0,643.454480673984,0,0
72.1499999999993,0,643.454480673984,0,0
72.1749999999993,0,643.454480673984,0,0
72.1999999999993,0,643.454480673984,0,0
72.2249999999993,0,643.454480673984,0,0
72.2499999999993,0,643.454480673984,0,0
72.2749999999993,0,643.454480673984,0,0
72.2999999999993,0,643.454480673984,0,0
72.3249999999993,0,643.454480673984,0,0
72.3499999999993,0,643.454480673984,0,0
72.3749999999993,0,643.454480673984,0,0
72.3999999999993,0,643.454480673984,0,0
72.4249999999993,0,643.454480673984,0,0
72.4499999999993,0,643.454480673984,0,0
72.4749999999994,0,643.454480673984,0,0
72.4999999999994,0,643.454480673984,0,0
72.5249999999994,0,643.454480673984,0,0
72.5499999999994,0,643.454480673984,0,0
72.5749999999994,0,643.454480673984,0,0
72.5999999999994,1,643.536605673984,0,0
72.6249999999994,0,643.618730673984,0,0
72.6499999999994,0,643.618730673984,0,0
72.6749999999994,0,643.618730673984,0,0
72.6999999999994,0,643.618730673984,0,0
72.7249999999994,0,643.618730673984,0,0

72.7499999999994,0,643.618730673984,0,0
72.7749999999994,1,643.700855673984,0,0
72.7999999999994,0,643.782980673984,0,0
72.8249999999994,0,643.782980673984,0,0
72.8499999999994,0,643.782980673984,0,0
72.8749999999994,0,643.782980673984,0,0
72.8999999999995,0,643.782980673984,0,0
72.9249999999995,0,643.782980673984,0,0
72.9499999999995,0,643.782980673984,0,0
72.9749999999995,0,643.782980673984,0,0
72.9999999999995,0,643.782980673984,0,0
73.0249999999995,0,643.782980673984,0,0
73.0499999999995,0,643.782980673984,0,0
73.0749999999995,0,643.782980673984,0,0
73.0999999999995,0,643.782980673984,0,0
73.1249999999995,0,643.782980673984,0,0
73.1499999999995,0,643.782980673984,0,0
73.1749999999995,1,643.865105673984,0,0
73.1999999999995,0,643.947230673984,0,0
73.2249999999995,0,643.947230673984,0,0
73.2499999999995,1,644.029355673984,0,0
73.2749999999995,0,644.111480673984,0,0
73.2999999999995,0,644.111480673984,0,0
73.3249999999995,0,644.111480673984,0,0
73.3499999999996,1,644.193605673984,0,0
73.3749999999996,0,644.275730673984,0,0
73.3999999999996,0,644.275730673984,0,0
73.4249999999996,0,644.275730673984,0,0
73.4499999999996,0,644.275730673984,0,0
73.4749999999996,0,644.275730673984,0,0
73.4999999999996,0,644.275730673984,0,0
73.5249999999996,0,644.275730673984,0,0
73.5499999999996,0,644.275730673984,0,0
73.5749999999996,0,644.275730673984,0,0
73.5999999999996,0,644.275730673984,0,0
73.6249999999996,0,644.275730673984,0,0
73.6499999999996,1,644.357855673984,0,0
73.6749999999996,0,644.439980673984,0,0
73.6999999999996,0,644.439980673984,0,0
73.7249999999996,0,644.439980673984,0,0
73.7499999999996,0,644.439980673984,0,0
73.7749999999997,0,644.439980673984,0,0
73.7999999999997,0,644.439980673984,0,0
73.8249999999997,0,644.439980673984,0,0
73.8499999999997,0,644.439980673984,0,0
73.8749999999997,0,644.439980673984,0,0
73.8999999999997,0,644.439980673984,0,0
73.9249999999997,0,644.439980673984,0,0
73.9499999999997,0,644.439980673984,0,0
73.9749999999997,0,644.439980673984,0,0
73.9999999999997,0,644.439980673984,0,0
74.0249999999997,0,644.439980673984,0,0

74.0499999999997,1,644.522105673984,0,0
74.0749999999997,0,644.604230673984,0,0
74.0999999999997,1,644.686355673984,0,0
74.1249999999997,0,644.768480673984,0,0
74.1499999999997,1,644.850605673984,0,0
74.1749999999997,1,645.014855673984,0,0
74.1999999999997,0,645.096980673984,0,0
74.2249999999998,0,645.096980673984,0,0
74.2499999999998,0,645.096980673984,0,0
74.2749999999998,1,645.179105673984,0,0
74.2999999999998,0,645.261230673984,0,0
74.3249999999998,0,645.261230673984,0,0
74.3499999999998,0,645.261230673984,0,0
74.3749999999998,0,645.261230673984,0,0
74.3999999999998,0,645.261230673984,0,0
74.4249999999998,0,645.261230673984,0,0
74.4499999999998,0,645.261230673984,0,0
74.4749999999998,0,645.261230673984,0,0
74.4999999999998,0,645.261230673984,0,0
74.5249999999998,0,645.261230673984,0,0
74.5499999999998,0,645.261230673984,0,0
74.5749999999998,0,645.261230673984,0,0
74.5999999999998,0,645.261230673984,0,0
74.6249999999998,0,645.261230673984,0,0
74.6499999999998,0,645.261230673984,0,0
74.6749999999999,0,645.261230673984,0,0
74.6999999999999,0,645.261230673984,0,0
74.7249999999999,0,645.261230673984,0,0
74.7499999999999,1,645.343355673984,0,0
74.7749999999999,0,645.425480673984,0,0
74.7999999999999,0,645.425480673984,0,0
74.8249999999999,0,645.425480673984,0,0
74.8499999999999,0,645.425480673984,0,0
74.8749999999999,0,645.425480673984,0,0
74.8999999999999,0,645.425480673984,0,0
74.9249999999999,0,645.425480673984,0,0
74.9499999999999,0,645.425480673984,0,0
74.9749999999999,0,645.425480673984,0,0
74.9999999999999,0,645.425480673984,0,0
75.0249999999999,0,645.425480673984,0,0
75.0499999999999,0,645.425480673984,0,0
75.0749999999999,0,645.425480673984,0,0
75.1,0,645.425480673984,0,0
75.125,0,645.425480673984,0,0
75.15,0,645.425480673984,0,0
75.175,4,645.589730673984,0,0
75.2,0,645.753980673984,0,0
75.225,3,645.896225346556,0,0
75.25,1,646.120595019127,0,0
75.275,0,646.202720019127,0,0
75.3,0,646.202720019127,0,0
75.325,0,646.202720019127,0,0

```
75.35,0,646.202720019127,0,0
75.375,2,646.318862307937,0,0
75.4,3,646.577249269319,0,0
75.425,0,646.71949394189,0,0
75.45,0,646.71949394189,0,0
75.475,1,646.80161894189,0,0
75.5,1,646.96586894189,0,0
75.525,0,647.04799394189,0,0
75.5500000000001,1,647.13011894189,0,0
75.5750000000001,1,647.294368941891,0,0
75.6000000000001,0,647.376493941891,0,0
75.6250000000001,0,647.376493941891,0,0
75.6500000000001,0,647.376493941891,0,0
75.6750000000001,0,647.376493941891,0,0
75.7000000000001,1,647.458618941891,0,0
75.7250000000001,0,647.540743941891,0,0
75.7500000000001,0,647.540743941891,0,0
75.7750000000001,1,647.622868941891,0,0
75.8000000000001,1,647.787118941891,0,0
75.8250000000001,0,647.869243941891,0,0
75.8500000000001,0,647.869243941891,0,0
75.8750000000001,0,647.869243941891,0,0
75.9000000000001,0,647.869243941891,0,0
75.9250000000001,0,647.869243941891,0,0
75.9500000000001,1,647.951368941891,0,0
75.9750000000002,0,648.033493941891,0,0
76.0000000000002,1,648.115618941891,0,0
76.0250000000002,0,648.197743941891,0,0
76.0500000000002,0,648.197743941891,0,0
76.0750000000002,0,648.197743941891,0,0
76.1000000000002,0,648.197743941891,0,0
76.1250000000002,0,648.197743941891,0,0
76.1500000000002,0,648.197743941891,0,0
76.1750000000002,0,648.197743941891,0,0
76.2000000000002,0,648.197743941891,0,0
76.2250000000002,0,648.197743941891,0,0
76.2500000000002,0,648.197743941891,0,0
76.2750000000002,0,648.197743941891,0,0
76.3000000000002,0,648.197743941891,0,0
76.3250000000002,0,648.197743941891,0,0
76.3500000000002,0,648.197743941891,0,0
76.3750000000002,0,648.197743941891,0,0
76.4000000000002,1,648.279868941891,0,0
76.4250000000003,0,648.361993941891,0,0
76.4500000000003,1,648.444118941891,0,0
76.4750000000003,1,648.608368941891,0,0
76.5000000000003,0,648.690493941891,0,0
76.5250000000003,1,648.772618941891,0,0
76.5500000000003,0,648.854743941891,0,0
76.5750000000003,0,648.854743941891,0,0
76.6000000000003,0,648.854743941891,0,0
76.6250000000003,0,648.854743941891,0,0
```

76.6500000000003,0,648.854743941891,0,0
76.6750000000003,1,648.936868941891,0,0
76.7000000000003,0,649.018993941891,0,0
76.7250000000003,1,649.101118941891,0,0
76.7500000000003,1,649.265368941891,0,0
76.7750000000003,0,649.347493941891,0,0
76.8000000000003,0,649.347493941891,0,0
76.8250000000003,0,649.347493941891,0,0
76.8500000000004,0,649.347493941891,0,0
76.8750000000004,0,649.347493941891,0,0
76.9000000000004,0,649.347493941891,0,0
76.9250000000004,0,649.347493941891,0,0
76.9500000000004,0,649.347493941891,0,0
76.9750000000004,0,649.347493941891,0,0
77.0000000000004,0,649.347493941891,0,0
77.0250000000004,0,649.347493941891,0,0
77.0500000000004,0,649.347493941891,0,0
77.0750000000004,0,649.347493941891,0,0
77.1000000000004,0,649.347493941891,0,0
77.1250000000004,0,649.347493941891,0,0
77.1500000000004,0,649.347493941891,0,0
77.1750000000004,0,649.347493941891,0,0
77.2000000000004,0,649.347493941891,0,0
77.2250000000004,0,649.347493941891,0,0
77.2500000000004,0,649.347493941891,0,0
77.2750000000004,0,649.347493941891,0,0
77.3000000000005,0,649.347493941891,0,0
77.3250000000005,0,649.347493941891,0,0
77.3500000000005,0,649.347493941891,0,0
77.3750000000005,0,649.347493941891,0,0
77.4000000000005,0,649.347493941891,0,0
77.4250000000005,12,649.631983287034,26.9563706011914,1
77.4500000000005,15,650.234541389485,26.9563706011914,1
77.4750000000005,15,650.870678904099,26.9563706011914,1
77.5000000000005,19,651.546722237145,26.9563706011914,1
77.5250000000005,20,652.271970978187,26.9563706011914,1
77.5500000000005,18,652.987672009921,26.9563706011914,1
77.5750000000005,17,653.674708925855,26.9563706011914,1
77.6000000000005,14,654.320602588248,26.9563706011914,1
77.6250000000005,12,654.91237554628,26.9563706011914,1
77.6500000000005,12,655.481354236566,26.9563706011914,1
77.6750000000005,13,656.061949480207,26.9563706011914,1
77.7000000000005,17,656.696665428208,26.9563706011914,1
77.7250000000005,19,657.39325005345,26.9563706011914,1
77.7500000000006,24,658.153553319441,26.9563706011914,1
77.7750000000006,25,658.966507009693,26.9563706011914,1
77.8000000000006,25,659.787757009693,26.9563706011914,1
77.8250000000006,25,660.609007009693,26.9563706011914,1
77.8500000000006,23,661.413489673545,26.9563706011914,1
77.8750000000006,22,662.192547731672,26.9563706011914,1
77.9000000000006,21,662.954092154896,26.9563706011914,1
77.9250000000006,19,663.688410759583,26.9563706011914,1

77.9500000000006,19,664.404359911059,26.9563706011914,1
77.9750000000006,16,665.090834486797,26.9563706011914,1
78.0000000000006,15,665.737403244105,26.9563706011914,1
78.0250000000006,14,666.362755614301,26.9563706011914,1
78.0500000000006,14,666.977322840078,26.9563706011914,1
78.0750000000006,17,667.623216502471,26.9563706011914,1
78.1000000000006,18,668.310253418405,26.9563706011914,1
78.1250000000006,20,669.025954450139,26.9563706011914,1
78.1500000000006,18,669.741655481873,26.9563706011914,1
78.1750000000007,16,670.418582348302,26.9563706011914,1
78.2000000000007,13,671.0431882468,26.9563706011914,1
78.2250000000007,12,671.62378349044,26.9563706011914,1
78.2500000000007,11,672.180650646492,26.9563706011914,1
78.2750000000007,10,672.712730510241,26.9563706011914,1
78.3000000000007,9,673.218807563082,26.9563706011914,1
78.3250000000007,9,673.711557563082,0,0
78.3500000000007,9,674.204307563082,0,0
78.3750000000007,8,674.682967140702,0,0
78.4000000000007,9,675.161626718322,0,0
78.4250000000007,9,675.654376718322,0,0
78.4500000000007,8,676.133036295941,0,0
78.4750000000007,7,676.582603199982,0,0
78.5000000000007,6,677.00104987153,0,0
78.5250000000007,6,677.403378561782,0,0
78.5500000000007,6,677.805707252034,0,0
78.5750000000007,7,678.224153923581,0,0
78.6000000000007,6,678.642600595128,0,0
78.6250000000008,6,679.04492928538,0,0
78.6500000000008,5,679.429730713159,0,0
78.6750000000008,5,679.797004878463,0,0
78.7000000000008,5,680.164279043767,0,0
78.7250000000008,5,680.531553209072,0,0
78.7500000000008,5,680.898827374376,0,0
78.7750000000008,4,681.246714457028,0,0
78.8000000000008,0,681.410964457028,0,0
78.8250000000008,1,681.493089457028,0,0
78.8500000000008,1,681.657339457028,0,0
78.8750000000008,0,681.739464457028,0,0
78.9000000000008,1,681.821589457028,0,0
78.9250000000008,1,681.985839457029,0,0
78.9500000000008,0,682.067964457029,0,0
78.9750000000008,2,682.184106745838,0,0
79.0000000000008,1,682.382374034648,0,0
79.0250000000008,2,682.580641323458,0,0
79.0500000000009,1,682.778908612268,0,0
79.0750000000009,0,682.861033612268,0,0
79.1000000000009,5,683.04467069492,0,0
79.1250000000009,0,683.228307777572,0,0
79.1500000000009,4,683.392557777573,0,0
79.1750000000009,5,683.740444860225,0,0
79.2000000000009,0,683.924081942877,0,0
79.2250000000009,0,683.924081942877,0,0

79.2500000000009,1,684.006206942877,0,0
79.2750000000009,0,684.088331942877,0,0
79.3000000000009,1,684.170456942877,0,0
79.3250000000009,0,684.252581942877,0,0
79.3500000000009,0,684.252581942877,0,0
79.3750000000009,0,684.252581942877,0,0
79.4000000000009,0,684.252581942877,0,0
79.4250000000009,0,684.252581942877,0,0
79.4500000000009,0,684.252581942877,0,0
79.4750000000009,0,684.252581942877,0,0
79.500000000001,0,684.252581942877,0,0
79.525000000001,0,684.252581942877,0,0
79.550000000001,0,684.252581942877,0,0
79.575000000001,0,684.252581942877,0,0
79.600000000001,0,684.252581942877,0,0
79.625000000001,0,684.252581942877,0,0
79.650000000001,0,684.252581942877,0,0
79.675000000001,0,684.252581942877,0,0
79.700000000001,0,684.252581942877,0,0
79.725000000001,0,684.252581942877,0,0
79.750000000001,0,684.252581942877,0,0
79.775000000001,0,684.252581942877,0,0
79.800000000001,0,684.252581942877,0,0
79.825000000001,0,684.252581942877,0,0
79.850000000001,0,684.252581942877,0,0
79.875000000001,0,684.252581942877,0,0
79.900000000001,0,684.252581942877,0,0
79.925000000001,0,684.252581942877,0,0
79.9500000000011,0,684.252581942877,0,0
79.9750000000011,0,684.252581942877,0,0
80.0000000000011,0,684.252581942877,0,0
80.0250000000011,0,684.252581942877,0,0
80.0500000000011,0,684.252581942877,0,0
80.0750000000011,0,684.252581942877,0,0
80.1000000000011,0,684.252581942877,0,0
80.1250000000011,0,684.252581942877,0,0
80.1500000000011,0,684.252581942877,0,0
80.1750000000011,0,684.252581942877,0,0
80.2000000000011,0,684.252581942877,0,0
80.2250000000011,0,684.252581942877,0,0
80.2500000000011,0,684.252581942877,0,0
80.2750000000011,0,684.252581942877,0,0
80.3000000000011,0,684.252581942877,0,0
80.3250000000011,0,684.252581942877,0,0
80.3500000000011,0,684.252581942877,0,0
80.3750000000012,0,684.252581942877,0,0
80.4000000000012,0,684.252581942877,0,0
80.4250000000012,0,684.252581942877,0,0
80.4500000000012,0,684.252581942877,0,0
80.4750000000012,0,684.252581942877,0,0
80.5000000000012,1,684.334706942877,0,0
80.5250000000012,0,684.416831942877,0,0

80.5500000000012,0,684.416831942877,0,0
80.5750000000012,0,684.416831942877,0,0
80.6000000000012,1,684.498956942877,0,0
80.6250000000012,1,684.663206942877,0,0
80.6500000000012,0,684.745331942877,0,0
80.6750000000012,0,684.745331942877,0,0
80.7000000000012,0,684.745331942877,0,0
80.7250000000012,0,684.745331942877,0,0
80.7500000000012,0,684.745331942877,0,0
80.7750000000012,0,684.745331942877,0,0
80.8000000000012,0,684.745331942877,0,0
80.8250000000013,0,684.745331942877,0,0
80.8500000000013,0,684.745331942877,0,0
80.8750000000013,0,684.745331942877,0,0
80.9000000000013,0,684.745331942877,0,0
80.9250000000013,0,684.745331942877,0,0
80.9500000000013,0,684.745331942877,0,0
80.9750000000013,1,684.827456942877,0,0
81.0000000000013,1,684.991706942877,0,0
81.0250000000013,1,685.155956942877,0,0
81.0500000000013,0,685.238081942877,0,0
81.0750000000013,0,685.238081942877,0,0
81.1000000000013,0,685.238081942877,0,0
81.1250000000013,0,685.238081942877,0,0
81.1500000000013,0,685.238081942877,0,0
81.1750000000013,0,685.238081942877,0,0
81.2000000000013,0,685.238081942877,0,0
81.2250000000013,0,685.238081942877,0,0
81.2500000000014,1,685.320206942877,0,0
81.2750000000014,1,685.484456942877,0,0
81.3000000000014,0,685.566581942877,0,0
81.3250000000014,1,685.648706942877,0,0
81.3500000000014,0,685.730831942877,0,0
81.3750000000014,1,685.812956942877,0,0
81.4000000000014,1,685.977206942877,0,0
81.4250000000014,1,686.141456942877,0,0
81.4500000000014,1,686.305706942877,0,0
81.4750000000014,1,686.469956942877,0,0
81.5000000000014,1,686.634206942877,0,0
81.5250000000014,1,686.798456942877,0,0
81.5500000000014,1,686.962706942878,0,0
81.5750000000014,3,687.187076615449,0,0
81.6000000000014,5,687.512958370673,0,0
81.6250000000014,7,687.913877779746,0,0
81.6500000000014,11,688.403537917075,24.4638533209457,1
81.6750000000014,14,688.983199340872,24.4638533209457,1
81.7000000000015,17,689.629093003265,24.4638533209457,1
81.7250000000015,19,690.325677628507,24.4638533209457,1
81.7500000000015,18,691.032079070675,24.4638533209457,1
81.7750000000015,20,691.747780102409,24.4638533209457,1
81.8000000000015,20,692.482328433018,24.4638533209457,1
81.8250000000015,21,693.22594662727,24.4638533209457,1

81.8500000000015,21,693.978634685167,24.4638533209457,1
81.8750000000015,24,694.757307404367,24.4638533209457,1
81.9000000000015,24,695.561964784871,24.4638533209457,1
81.9250000000015,24,696.366622165376,24.4638533209457,1
81.9500000000015,22,697.154151249903,24.4638533209457,1
81.9750000000015,22,697.924552038454,24.4638533209457,1
82.0000000000015,19,698.667727008468,24.4638533209457,1
82.0250000000015,18,699.374128450636,24.4638533209457,1
82.0500000000015,17,700.06116536657,24.4638533209457,1
82.0750000000015,16,700.728275416073,24.4638533209457,1
82.1000000000015,13,701.352881314571,24.4638533209457,1
82.1250000000015,13,701.945093111566,24.4638533209457,1
82.1500000000016,13,702.537304908561,24.4638533209457,1
82.1750000000016,12,703.117900152202,24.4638533209457,1
82.2000000000016,13,703.698495395842,24.4638533209457,1
82.2250000000016,14,704.301884907229,24.4638533209457,1
82.2500000000016,14,704.916452133006,24.4638533209457,1
82.2750000000016,14,705.531019358784,24.4638533209457,1
82.3000000000016,13,706.13440887017,24.4638533209457,1
82.3250000000016,15,706.748583525975,24.4638533209457,1
82.3500000000016,17,707.405262332786,24.4638533209457,1
82.3750000000016,16,708.07237238229,24.4638533209457,1
82.4000000000016,15,708.718941139597,24.4638533209457,1
82.4250000000016,16,709.365509896904,24.4638533209457,1
82.4500000000016,14,710.001293509793,24.4638533209457,1
82.4750000000016,12,710.593066467825,24.4638533209457,1
82.5000000000016,12,711.162045158111,24.4638533209457,1
82.5250000000016,10,711.706236556096,24.4638533209457,1
82.5500000000016,12,712.25042795408,24.4638533209457,1
82.5750000000017,9,712.781292299224,24.4638533209457,1
82.6000000000017,9,713.274042299224,24.4638533209457,1
82.6250000000017,15,713.838486056531,24.4638533209457,1
82.6500000000017,6,714.357719158964,24.4638533209457,1
82.6750000000017,7,714.776165830511,24.4638533209457,1
82.7000000000017,6,715.194612502059,24.4638533209457,1
82.7250000000017,6,715.596941192311,24.4638533209457,1
82.7500000000017,7,716.015387863858,24.4638533209457,1
82.7750000000017,8,716.464954767899,24.4638533209457,1
82.8000000000017,4,716.861489345519,24.4638533209457,1
82.8250000000017,11,717.298117156427,24.4638533209457,1
82.8500000000017,12,717.854984312478,24.4638533209457,1
82.8750000000017,8,718.371758235241,24.4638533209457,1
82.9000000000017,5,718.787679895513,0,0
82.9250000000017,9,719.217691978165,0,0
82.9500000000017,5,719.647704060817,0,0
82.9750000000017,2,719.947483432279,0,0
83.0000000000017,1,720.145750721089,0,0
83.0250000000018,3,720.370120393661,0,0
83.0500000000018,1,720.594490066232,0,0
83.0750000000018,0,720.676615066232,0,0
83.1000000000018,1,720.758740066232,0,0
83.1250000000018,1,720.922990066232,0,0

83.1500000000018,0,721.005115066232,0,0
83.1750000000018,1,721.087240066232,0,0
83.2000000000018,1,721.251490066232,0,0
83.2250000000018,1,721.415740066232,0,0
83.2500000000018,0,721.497865066232,0,0
83.2750000000018,0,721.497865066232,0,0
83.3000000000018,0,721.497865066232,0,0
83.3250000000018,0,721.497865066232,0,0
83.3500000000018,1,721.579990066232,0,0
83.3750000000018,0,721.662115066232,0,0
83.4000000000018,1,721.744240066232,0,0
83.4250000000018,1,721.908490066232,0,0
83.4500000000019,1,722.072740066232,0,0
83.4750000000019,1,722.236990066232,0,0
83.5000000000019,1,722.401240066233,0,0
83.5250000000019,1,722.565490066233,0,0
83.5500000000019,0,722.647615066233,0,0
83.5750000000019,0,722.647615066233,0,0
83.6000000000019,1,722.729740066233,0,0
83.6250000000019,1,722.893990066233,0,0
83.6500000000019,0,722.976115066233,0,0
83.6750000000019,0,722.976115066233,0,0
83.7000000000019,0,722.976115066233,0,0
83.7250000000019,0,722.976115066233,0,0
83.7500000000019,0,722.976115066233,0,0
83.7750000000019,1,723.058240066233,0,0
83.8000000000019,0,723.140365066233,0,0
83.8250000000019,0,723.140365066233,0,0
83.8500000000019,0,723.140365066233,0,0
83.8750000000019,0,723.140365066233,0,0
83.900000000002,0,723.140365066233,0,0
83.925000000002,0,723.140365066233,0,0
83.950000000002,0,723.140365066233,0,0
83.975000000002,1,723.222490066233,0,0
84.000000000002,0,723.304615066233,0,0
84.025000000002,0,723.304615066233,0,0
84.050000000002,0,723.304615066233,0,0
84.075000000002,0,723.304615066233,0,0
84.100000000002,0,723.304615066233,0,0
84.125000000002,0,723.304615066233,0,0
84.150000000002,0,723.304615066233,0,0
84.175000000002,0,723.304615066233,0,0
84.200000000002,0,723.304615066233,0,0
84.225000000002,0,723.304615066233,0,0
84.250000000002,1,723.386740066233,0,0
84.275000000002,1,723.550990066233,0,0
84.300000000002,1,723.715240066233,0,0
84.325000000002,0,723.797365066233,0,0
84.3500000000021,0,723.797365066233,0,0
84.3750000000021,1,723.879490066233,0,0
84.4000000000021,1,724.043740066233,0,0
84.4250000000021,0,724.125865066233,0,0

84.4500000000021,1,724.207990066233,0,0
84.4750000000021,1,724.372240066233,0,0
84.5000000000021,1,724.536490066233,0,0
84.5250000000021,1,724.700740066233,0,0
84.5500000000021,1,724.864990066233,0,0
84.5750000000021,1,725.029240066233,0,0
84.6000000000021,0,725.111365066233,0,0
84.6250000000021,1,725.193490066233,0,0
84.6500000000021,0,725.275615066233,0,0
84.6750000000021,1,725.357740066233,0,0
84.7000000000021,1,725.521990066233,0,0
84.7250000000021,0,725.604115066233,0,0
84.7500000000021,1,725.686240066233,0,0
84.7750000000022,0,725.768365066233,0,0
84.8000000000022,0,725.768365066233,0,0
84.8250000000022,0,725.768365066233,0,0
84.8500000000022,0,725.768365066233,0,0
84.8750000000022,0,725.768365066233,0,0
84.9000000000022,0,725.768365066233,0,0
84.9250000000022,0,725.768365066233,0,0
84.9500000000022,0,725.768365066233,0,0
84.9750000000022,1,725.850490066233,0,0
85.0000000000022,1,726.014740066233,0,0
85.0250000000022,0,726.096865066233,0,0
85.0500000000022,0,726.096865066233,0,0
85.0750000000022,0,726.096865066233,0,0
85.1000000000022,0,726.096865066233,0,0
85.1250000000022,0,726.096865066233,0,0
85.1500000000022,1,726.178990066233,0,0
85.1750000000022,1,726.343240066233,0,0
85.2000000000022,0,726.425365066233,0,0
85.2250000000023,1,726.507490066233,0,0
85.2500000000023,0,726.589615066233,0,0
85.2750000000023,0,726.589615066233,0,0
85.3000000000023,1,726.671740066234,0,0
85.3250000000023,1,726.835990066234,0,0
85.3500000000023,0,726.918115066234,0,0
85.3750000000023,0,726.918115066234,0,0
85.4000000000023,0,726.918115066234,0,0
85.4250000000023,0,726.918115066234,0,0
85.4500000000023,0,726.918115066234,0,0
85.4750000000023,0,726.918115066234,0,0
85.5000000000023,0,726.918115066234,0,0
85.5250000000023,0,726.918115066234,0,0
85.5500000000023,0,726.918115066234,0,0
85.5750000000023,0,726.918115066234,0,0
85.6000000000023,1,727.000240066234,0,0
85.6250000000023,1,727.164490066234,0,0
85.6500000000024,1,727.328740066234,0,0
85.6750000000024,0,727.410865066234,0,0
85.7000000000024,0,727.410865066234,0,0
85.7250000000024,1,727.492990066234,0,0

85.7500000000024,0,727.575115066234,0,0
85.7750000000024,1,727.657240066234,0,0
85.8000000000024,0,727.739365066234,0,0
85.8250000000024,0,727.739365066234,0,0
85.8500000000024,1,727.821490066234,0,0
85.8750000000024,0,727.903615066234,0,0
85.9000000000024,1,727.985740066234,0,0
85.9250000000024,0,728.067865066234,0,0
85.9500000000024,0,728.067865066234,0,0
85.9750000000024,0,728.067865066234,0,0
86.0000000000024,0,728.067865066234,0,0
86.0250000000024,0,728.067865066234,0,0
86.0500000000024,0,728.067865066234,0,0
86.0750000000024,0,728.067865066234,0,0
86.1000000000025,0,728.067865066234,0,0
86.1250000000025,1,728.149990066234,0,0
86.1500000000025,0,728.232115066234,0,0
86.1750000000025,0,728.232115066234,0,0
86.2000000000025,0,728.232115066234,0,0
86.2250000000025,0,728.232115066234,0,0
86.2500000000025,1,728.314240066234,0,0
86.2750000000025,0,728.396365066234,0,0
86.3000000000025,1,728.478490066234,0,0
86.3250000000025,0,728.560615066234,0,0
86.3500000000025,0,728.560615066234,0,0
86.3750000000025,2,728.676757355044,0,0
86.4000000000025,2,728.909041932664,0,0
86.4250000000025,2,729.141326510283,0,0
86.4500000000025,0,729.257468799093,0,0
86.4750000000025,0,729.257468799093,0,0
86.5000000000025,0,729.257468799093,0,0
86.5250000000025,0,729.257468799093,0,0
86.5500000000026,0,729.257468799093,0,0
86.5750000000026,1,729.339593799093,0,0
86.6000000000026,0,729.421718799093,0,0
86.6250000000026,0,729.421718799093,0,0
86.6500000000026,0,729.421718799093,0,0
86.6750000000026,0,729.421718799093,0,0
86.7000000000026,0,729.421718799093,0,0
86.7250000000026,0,729.421718799093,0,0
86.7500000000026,0,729.421718799093,0,0
86.7750000000026,0,729.421718799093,0,0
86.8000000000026,0,729.421718799093,0,0
86.8250000000026,1,729.503843799093,0,0
86.8500000000026,0,729.585968799093,0,0
86.8750000000026,0,729.585968799093,0,0
86.9000000000026,0,729.585968799093,0,0
86.9250000000026,0,729.585968799093,0,0
86.9500000000026,0,729.585968799093,0,0
86.9750000000027,1,729.668093799093,0,0
87.0000000000027,0,729.750218799093,0,0
87.0250000000027,1,729.832343799093,0,0

87.0500000000027,0,729.914468799093,0,0
87.0750000000027,0,729.914468799093,0,0
87.1000000000027,0,729.914468799093,0,0
87.1250000000027,0,729.914468799093,0,0
87.1500000000027,0,729.914468799093,0,0
87.1750000000027,0,729.914468799093,0,0
87.2000000000027,0,729.914468799093,0,0
87.2250000000027,0,729.914468799093,0,0
87.2500000000027,2,730.030611087903,0,0
87.2750000000027,4,730.311003376713,0,0
87.3000000000027,7,730.692535703134,0,0
87.3250000000027,8,731.142102607175,0,0
87.3500000000027,9,731.620762184795,0,0
87.3750000000027,9,732.113512184795,0,0
87.4000000000027,12,732.644376529938,24.7551525062979,1
87.4250000000028,14,733.23614948797,24.7551525062979,1
87.4500000000028,14,733.850716713748,24.7551525062979,1
87.4750000000028,16,734.486500326637,24.7551525062979,1
87.5000000000028,18,735.163427193066,24.7551525062979,1
87.5250000000028,18,735.860280925926,24.7551525062979,1
87.5500000000028,18,736.557134658785,24.7551525062979,1
87.5750000000028,19,737.263536100953,24.7551525062979,1
87.6000000000028,17,737.960120726195,24.7551525062979,1
87.6250000000028,17,738.637340825203,24.7551525062979,1
87.6500000000028,17,739.314560924211,24.7551525062979,1
87.6750000000028,17,739.991781023218,24.7551525062979,1
87.7000000000028,17,740.669001122226,24.7551525062979,1
87.7250000000028,17,741.346221221234,24.7551525062979,1
87.7500000000028,15,742.002900028045,24.7551525062979,1
87.7750000000028,16,742.649468785352,24.7551525062979,1
87.8000000000028,16,743.306468785352,24.7551525062979,1
87.8250000000028,16,743.963468785352,24.7551525062979,1
87.8500000000029,14,744.599252398241,24.7551525062979,1
87.8750000000029,14,745.213819624019,24.7551525062979,1
87.9000000000029,14,745.828386849796,24.7551525062979,1
87.9250000000029,14,746.442954075574,24.7551525062979,1
87.9500000000029,15,747.06830644577,24.7551525062979,1
87.9750000000029,15,747.704443960385,24.7551525062979,1
88.0000000000029,14,748.329796330581,24.7551525062979,1
88.0250000000029,14,748.944363556358,24.7551525062979,1
88.0500000000029,13,749.547753067744,24.7551525062979,1
88.0750000000029,13,750.139964864739,24.7551525062979,1
88.1000000000029,12,750.72056010838,24.7551525062979,1
88.1250000000029,11,751.277427264431,24.7551525062979,1
88.1500000000029,12,751.834294420482,24.7551525062979,1
88.1750000000029,10,752.378485818467,24.7551525062979,1
88.2000000000029,11,752.910565682216,24.7551525062979,1
88.2250000000029,15,753.501012250431,24.7551525062979,1
88.2500000000029,21,754.195425036687,24.7551525062979,1
88.2750000000029,23,754.965626729487,24.7551525062979,1
88.300000000003,23,755.753342057191,24.7551525062979,1
88.325000000003,22,756.532400115319,24.7551525062979,1

88.350000000003,20,757.284874674899,24.7551525062979,1
88.375000000003,18,758.000575706633,24.7551525062979,1
88.400000000003,17,758.687612622566,24.7551525062979,1
88.425000000003,17,759.364832721574,24.7551525062979,1
88.450000000003,16,760.031942771078,24.7551525062979,1
88.475000000003,15,760.678511528385,24.7551525062979,1
88.500000000003,15,761.314649043,24.7551525062979,1
88.525000000003,13,761.928823698804,24.7551525062979,1
88.550000000003,13,762.521035495799,24.7551525062979,1
88.575000000003,13,763.113247292794,24.7551525062979,1
88.600000000003,12,763.693842536435,24.7551525062979,1
88.625000000003,11,764.250709692486,24.7551525062979,1
88.650000000003,11,764.795465314302,24.7551525062979,1
88.675000000003,10,765.327545178051,24.7551525062979,1
88.700000000003,10,765.846949283734,24.7551525062979,1
88.7250000000031,9,766.353026336575,24.7551525062979,1
88.7500000000031,8,766.831685914195,24.7551525062979,1
88.7750000000031,8,767.296255069435,24.7551525062979,1
88.8000000000031,9,767.774914647054,24.7551525062979,1
88.8250000000031,10,768.280991699896,24.7551525062979,1
88.8500000000031,11,768.813071563645,24.7551525062979,1
88.8750000000031,10,769.345151427394,24.7551525062979,1
88.9000000000031,11,769.877231291143,24.7551525062979,1
88.9250000000031,9,770.395984102051,24.7551525062979,1
88.9500000000031,6,770.843523447177,0,0
88.9750000000031,6,771.245852137429,0,0
89.0000000000031,5,771.630653565208,0,0
89.0250000000031,4,771.97854064786,0,0
89.0500000000031,4,772.30704064786,0,0
89.0750000000031,4,772.63554064786,0,0
89.1000000000031,3,772.942035320431,0,0
89.1250000000031,3,773.226524665575,0,0
89.1500000000031,4,773.533019338146,0,0
89.1750000000032,3,773.839514010718,0,0
89.2000000000032,1,774.063883683289,0,0
89.2250000000032,1,774.228133683289,0,0
89.2500000000032,1,774.392383683289,0,0
89.2750000000032,1,774.55663368329,0,0
89.3000000000032,2,774.754900972099,0,0
89.3250000000032,2,774.987185549719,0,0
89.3500000000032,2,775.219470127339,0,0
89.3750000000032,3,775.477857088721,0,0
89.4000000000032,3,775.762346433864,0,0
89.4250000000032,3,776.046835779007,0,0
89.4500000000032,1,776.271205451579,0,0
89.4750000000032,0,776.353330451579,0,0
89.5000000000032,0,776.353330451579,0,0
89.5250000000032,0,776.353330451579,0,0
89.5500000000032,0,776.353330451579,0,0
89.5750000000032,0,776.353330451579,0,0
89.6000000000032,0,776.353330451579,0,0
89.6250000000033,1,776.435455451579,0,0

89.6500000000033,0,776.517580451579,0,0
89.6750000000033,0,776.517580451579,0,0
89.7000000000033,0,776.517580451579,0,0
89.7250000000033,0,776.517580451579,0,0
89.7500000000033,0,776.517580451579,0,0
89.7750000000033,0,776.517580451579,0,0
89.8000000000033,0,776.517580451579,0,0
89.8250000000033,0,776.517580451579,0,0
89.8500000000033,0,776.517580451579,0,0
89.8750000000033,1,776.599705451579,0,0
89.9000000000033,0,776.681830451579,0,0
89.9250000000033,0,776.681830451579,0,0
89.9500000000033,0,776.681830451579,0,0
89.9750000000033,0,776.681830451579,0,0
90.0000000000033,0,776.681830451579,0,0
90.0250000000033,0,776.681830451579,0,0
90.0500000000034,0,776.681830451579,0,0
90.0750000000034,0,776.681830451579,0,0
90.1000000000034,1,776.763955451579,0,0
90.1250000000034,0,776.846080451579,0,0
90.1500000000034,0,776.846080451579,0,0
90.1750000000034,0,776.846080451579,0,0
90.2000000000034,0,776.846080451579,0,0
90.2250000000034,0,776.846080451579,0,0
90.2500000000034,6,777.047244796705,0,0
90.2750000000034,0,777.248409141831,0,0
90.3000000000034,0,777.248409141831,0,0
90.3250000000034,0,777.248409141831,0,0
90.3500000000034,1,777.330534141831,0,0
90.3750000000034,0,777.412659141831,0,0
90.4000000000034,0,777.412659141831,0,0
90.4250000000034,0,777.412659141831,0,0
90.4500000000034,1,777.494784141831,0,0
90.4750000000034,0,777.576909141831,0,0
90.5000000000035,0,777.576909141831,0,0
90.5250000000035,0,777.576909141831,0,0
90.5500000000035,1,777.659034141831,0,0
90.5750000000035,1,777.823284141831,0,0
90.6000000000035,1,777.987534141831,0,0
90.6250000000035,0,778.069659141831,0,0
90.6500000000035,1,778.151784141831,0,0
90.6750000000035,0,778.233909141831,0,0
90.7000000000035,0,778.233909141831,0,0
90.7250000000035,0,778.233909141831,0,0
90.7500000000035,0,778.233909141831,0,0
90.7750000000035,1,778.316034141831,0,0
90.8000000000035,0,778.398159141831,0,0
90.8250000000035,0,778.398159141831,0,0
90.8500000000035,0,778.398159141831,0,0
90.8750000000035,0,778.398159141831,0,0
90.9000000000035,0,778.398159141831,0,0
90.9250000000036,0,778.398159141831,0,0

90.9500000000036,0,778.398159141831,0,0
90.9750000000036,1,778.480284141831,0,0
91.0000000000036,1,778.644534141831,0,0
91.0250000000036,1,778.808784141831,0,0
91.0500000000036,0,778.890909141831,0,0
91.0750000000036,1,778.973034141831,0,0
91.1000000000036,1,779.137284141831,0,0
91.1250000000036,1,779.301534141831,0,0
91.1500000000036,0,779.383659141831,0,0
91.1750000000036,1,779.465784141831,0,0
91.2000000000036,1,779.630034141831,0,0
91.2250000000036,0,779.712159141831,0,0
91.2500000000036,1,779.794284141831,0,0
91.2750000000036,1,779.958534141831,0,0
91.3000000000036,0,780.040659141831,0,0
91.3250000000036,1,780.122784141831,0,0
91.3500000000036,0,780.204909141831,0,0
91.3750000000037,0,780.204909141831,0,0
91.4000000000037,1,780.287034141831,0,0
91.4250000000037,1,780.451284141831,0,0
91.4500000000037,0,780.533409141832,0,0
91.4750000000037,0,780.533409141832,0,0
91.5000000000037,0,780.533409141832,0,0
91.5250000000037,1,780.615534141832,0,0
91.5500000000037,0,780.697659141832,0,0
91.5750000000037,0,780.697659141832,0,0
91.6000000000037,0,780.697659141832,0,0
91.6250000000037,0,780.697659141832,0,0
91.6500000000037,1,780.779784141832,0,0
91.6750000000037,0,780.861909141832,0,0
91.7000000000037,0,780.861909141832,0,0
91.7250000000037,0,780.861909141832,0,0
91.7500000000037,0,780.861909141832,0,0
91.7750000000037,0,780.861909141832,0,0
91.8000000000037,0,780.861909141832,0,0
91.8250000000038,1,780.944034141832,0,0
91.8500000000038,0,781.026159141832,0,0
91.8750000000038,0,781.026159141832,0,0
91.9000000000038,0,781.026159141832,0,0
91.9250000000038,1,781.108284141832,0,0
91.9500000000038,0,781.190409141832,0,0
91.9750000000038,0,781.190409141832,0,0
92.0000000000038,0,781.190409141832,0,0
92.0250000000038,1,781.272534141832,0,0
92.0500000000038,0,781.354659141832,0,0
92.0750000000038,0,781.354659141832,0,0
92.1000000000038,0,781.354659141832,0,0
92.1250000000038,0,781.354659141832,0,0
92.1500000000038,0,781.354659141832,0,0
92.1750000000038,0,781.354659141832,0,0
92.2000000000038,0,781.354659141832,0,0
92.2250000000038,0,781.354659141832,0,0

92.2500000000039,1,781.436784141832,0,0
92.2750000000039,1,781.601034141832,0,0
92.3000000000039,1,781.765284141832,0,0
92.3250000000039,1,781.929534141832,0,0
92.3500000000039,0,782.011659141832,0,0
92.3750000000039,1,782.093784141832,0,0
92.4000000000039,0,782.175909141832,0,0
92.4250000000039,1,782.258034141832,0,0
92.4500000000039,1,782.422284141832,0,0
92.4750000000039,0,782.504409141832,0,0
92.5000000000039,1,782.586534141832,0,0
92.5250000000039,1,782.750784141832,0,0
92.5500000000039,1,782.915034141832,0,0
92.5750000000039,0,782.997159141832,0,0
92.6000000000039,0,782.997159141832,0,0
92.6250000000039,1,783.079284141832,0,0
92.6500000000039,1,783.243534141832,0,0
92.6750000000039,4,783.489909141832,0,0
92.700000000004,3,783.796403814404,0,0
92.725000000004,0,783.938648486975,0,0
92.750000000004,23,784.332506150827,24.5826377647182,1
92.775000000004,28,785.160928467522,24.5826377647182,1
92.800000000004,24,785.997821810616,24.5826377647182,1
92.825000000004,19,786.758125076607,24.5826377647182,1
92.850000000004,12,787.400588997488,24.5826377647182,1
92.875000000004,13,787.981184241129,24.5826377647182,1
92.900000000004,14,788.584573752515,24.5826377647182,1
92.925000000004,14,789.199140978293,24.5826377647182,1
92.950000000004,10,789.766126644023,24.5826377647182,1
92.975000000004,10,790.285530749705,24.5826377647182,1
93.000000000004,9,790.791607802547,24.5826377647182,1
93.025000000004,9,791.284357802547,24.5826377647182,1
93.050000000004,11,791.803110613455,24.5826377647182,1
93.075000000004,9,792.321863424363,24.5826377647182,1
93.100000000004,3,792.710483096934,0,0
93.1250000000041,4,793.016977769506,0,0
93.1500000000041,2,793.297370058316,0,0
93.1750000000041,1,793.495637347126,0,0
93.2000000000041,1,793.659887347126,0,0
93.2250000000041,2,793.858154635936,0,0
93.2500000000041,2,794.090439213555,0,0
93.2750000000041,2,794.322723791175,0,0
93.3000000000041,1,794.520991079985,0,0
93.3250000000041,1,794.685241079985,0,0
93.3500000000041,3,794.909610752557,0,0
93.3750000000041,4,795.216105425128,0,0
93.4000000000041,4,795.544605425128,0,0
93.4250000000041,3,795.8511000977,0,0
93.4500000000041,5,796.176981852924,0,0
93.4750000000041,5,796.544256018228,0,0
93.5000000000041,6,796.929057446006,0,0
93.5250000000041,7,797.347504117554,0,0

93.5500000000041,7,797.782068770396,0,0
93.5750000000042,5,798.182988179469,0,0
93.6000000000042,9,798.613000262121,0,0
93.6250000000042,5,799.043012344774,0,0
93.6500000000042,7,799.443931753847,0,0
93.6750000000042,7,799.878496406689,0,0
93.7000000000042,7,800.313061059532,0,0
93.7250000000042,8,800.762627963573,0,0
93.7500000000042,11,801.2672903521,21.6244247390991,1
93.7750000000042,12,801.824157508151,21.6244247390991,1
93.8000000000042,16,802.437146853295,21.6244247390991,1
93.8250000000042,15,803.083715610602,21.6244247390991,1
93.8500000000042,15,803.719853125216,21.6244247390991,1
93.8750000000042,12,804.322411227667,21.6244247390991,1
93.9000000000042,15,804.924969330117,21.6244247390991,1
93.9250000000042,6,805.444202432551,21.6244247390991,1
93.9500000000042,10,805.905068830518,21.6244247390991,1
93.9750000000042,11,806.437148694267,21.6244247390991,1
94.0000000000042,11,806.981904316083,21.6244247390991,1
94.0250000000043,10,807.513984179832,21.6244247390991,1
94.0500000000043,10,808.033388285515,21.6244247390991,1
94.0750000000043,10,808.552792391198,21.6244247390991,1
94.1000000000043,10,809.07219649688,21.6244247390991,1
94.1250000000043,11,809.604276360629,21.6244247390991,1
94.1500000000043,10,810.136356224379,21.6244247390991,1
94.1750000000043,11,810.668436088128,21.6244247390991,1
94.2000000000043,12,811.225303244179,21.6244247390991,1
94.2250000000043,11,811.78217040023,21.6244247390991,1
94.2500000000043,10,812.314250263979,21.6244247390991,1
94.2750000000043,9,812.820327316821,21.6244247390991,1
94.3000000000043,9,813.313077316821,21.6244247390991,1
94.3250000000043,9,813.805827316821,21.6244247390991,1
94.3500000000043,9,814.298577316821,21.6244247390991,1
94.3750000000043,10,814.804654369662,21.6244247390991,1
94.4000000000043,11,815.336734233411,21.6244247390991,1
94.4250000000043,11,815.881489855227,21.6244247390991,1
94.4500000000044,11,816.426245477043,21.6244247390991,1
94.4750000000044,9,816.944998287951,21.6244247390991,1
94.5000000000044,8,817.42365786557,0,0
94.5250000000044,8,817.88822702081,0,0
94.5500000000044,8,818.35279617605,0,0
94.5750000000044,8,818.817365331289,0,0
94.6000000000044,8,819.281934486529,0,0
94.6250000000044,8,819.746503641768,0,0
94.6500000000044,7,820.196070545809,0,0
94.6750000000044,7,820.630635198652,0,0
94.7000000000044,8,821.080202102693,0,0
94.7250000000044,9,821.558861680312,0,0
94.7500000000044,10,822.064938733154,26.382798795661,1
94.7750000000044,13,822.620746684492,26.382798795661,1
94.8000000000044,16,823.24535258299,26.382798795661,1
94.8250000000044,18,823.92227944942,26.382798795661,1

94.8500000000044,19,824.628680891588,26.382798795661,1
94.8750000000044,23,825.380513131178,26.382798795661,1
94.9000000000045,22,826.159571189306,26.382798795661,1
94.9250000000045,22,826.929971977857,26.382798795661,1
94.9500000000045,21,827.69151640108,26.382798795661,1
94.9750000000045,22,828.453060824304,26.382798795661,1
95.0000000000045,22,829.223461612855,26.382798795661,1
95.0250000000045,22,829.993862401406,26.382798795661,1
95.0500000000045,22,830.764263189957,26.382798795661,1
95.0750000000045,23,831.543321248085,26.382798795661,1
95.1000000000045,24,832.339507602189,26.382798795661,1
95.1250000000045,23,833.135693956293,26.382798795661,1
95.1500000000045,22,833.914752014421,26.382798795661,1
95.1750000000045,20,834.667226574,26.382798795661,1
95.2000000000045,18,835.382927605734,26.382798795661,1
95.2250000000045,18,836.079781338594,26.382798795661,1
95.2500000000045,17,836.766818254527,26.382798795661,1
95.2750000000045,16,837.433928304031,26.382798795661,1
95.3000000000045,14,838.06971191692,26.382798795661,1
95.3250000000046,13,838.673101428306,26.382798795661,1
95.3500000000046,11,839.241585137712,26.382798795661,1
95.3750000000046,11,839.786340759528,26.382798795661,1
95.4000000000046,12,840.343207915579,26.382798795661,1
95.4250000000046,12,840.912186605865,26.382798795661,1
95.4500000000046,13,841.492781849506,26.382798795661,1
95.4750000000046,15,842.10695650531,26.382798795661,1
95.5000000000046,14,842.732308875507,26.382798795661,1
95.5250000000046,13,843.335698386893,26.382798795661,1
95.5500000000046,12,843.916293630533,26.382798795661,1
95.5750000000046,10,844.460485028518,26.382798795661,1
95.6000000000046,10,844.979889134201,26.382798795661,1
95.6250000000046,9,845.485966187042,26.382798795661,1
95.6500000000046,10,845.992043239883,26.382798795661,1
95.6750000000046,7,846.469027619146,26.382798795661,1
95.7000000000046,7,846.903592271988,0,0
95.7250000000046,7,847.33815692483,0,0
95.7500000000046,6,847.756603596378,0,0
95.7750000000047,5,848.141405024156,0,0
95.8000000000047,4,848.489292106808,0,0
95.8250000000047,4,848.817792106808,0,0
95.8500000000047,4,849.146292106808,0,0
95.8750000000047,3,849.45278677938,0,0
95.9000000000047,3,849.737276124523,0,0
95.9250000000047,2,849.995663085904,0,0
95.9500000000047,1,850.193930374714,0,0
95.9750000000047,0,850.276055374714,0,0
96.0000000000047,0,850.276055374714,0,0
96.0250000000047,0,850.276055374714,0,0
96.0500000000047,0,850.276055374714,0,0
96.0750000000047,0,850.276055374714,0,0
96.1000000000047,0,850.276055374714,0,0
96.1250000000047,1,850.358180374714,0,0

96.1500000000047,0,850.440305374714,0,0
96.1750000000047,0,850.440305374714,0,0
96.2000000000047,0,850.440305374714,0,0
96.2250000000048,0,850.440305374714,0,0
96.2500000000048,0,850.440305374714,0,0
96.2750000000048,0,850.440305374714,0,0
96.3000000000048,0,850.440305374714,0,0
96.3250000000048,0,850.440305374714,0,0
96.3500000000048,0,850.440305374714,0,0
96.3750000000048,0,850.440305374714,0,0
96.4000000000048,0,850.440305374714,0,0
96.4250000000048,0,850.440305374714,0,0
96.4500000000048,0,850.440305374714,0,0
96.4750000000048,1,850.522430374714,0,0
96.5000000000048,0,850.604555374714,0,0
96.5250000000048,1,850.686680374714,0,0
96.5500000000048,0,850.768805374714,0,0
96.5750000000048,1,850.850930374714,0,0
96.6000000000048,1,851.015180374714,0,0
96.6250000000048,1,851.179430374714,0,0
96.6500000000049,0,851.261555374714,0,0
96.6750000000049,0,851.261555374714,0,0
96.7000000000049,0,851.261555374714,0,0
96.7250000000049,1,851.343680374714,0,0
96.7500000000049,1,851.507930374715,0,0
96.7750000000049,0,851.590055374715,0,0
96.8000000000049,1,851.672180374715,0,0
96.8250000000049,1,851.836430374715,0,0
96.8500000000049,0,851.918555374715,0,0
96.8750000000049,5,852.102192457367,0,0
96.9000000000049,3,852.428074212591,0,0
96.9250000000049,0,852.570318885162,0,0
96.9500000000049,4,852.734568885162,0,0
96.9750000000049,0,852.898818885162,0,0
97.0000000000049,0,852.898818885162,0,0
97.0250000000049,1,852.980943885162,0,0
97.0500000000049,0,853.063068885162,0,0
97.0750000000049,0,853.063068885162,0,0
97.100000000005,0,853.063068885162,0,0
97.125000000005,0,853.063068885162,0,0
97.150000000005,0,853.063068885162,0,0
97.175000000005,0,853.063068885162,0,0
97.200000000005,0,853.063068885162,0,0
97.225000000005,0,853.063068885162,0,0
97.250000000005,0,853.063068885162,0,0
97.275000000005,0,853.063068885162,0,0
97.300000000005,0,853.063068885162,0,0
97.325000000005,0,853.063068885162,0,0
97.350000000005,0,853.063068885162,0,0
97.375000000005,0,853.063068885162,0,0
97.400000000005,0,853.063068885162,0,0
97.425000000005,0,853.063068885162,0,0

97.450000000005,0,853.063068885162,0,0
97.475000000005,0,853.063068885162,0,0
97.500000000005,0,853.063068885162,0,0
97.5250000000051,0,853.063068885162,0,0
97.5500000000051,0,853.063068885162,0,0
97.5750000000051,0,853.063068885162,0,0
97.6000000000051,0,853.063068885162,0,0
97.6250000000051,0,853.063068885162,0,0
97.6500000000051,0,853.063068885162,0,0
97.6750000000051,0,853.063068885162,0,0
97.7000000000051,0,853.063068885162,0,0
97.7250000000051,0,853.063068885162,0,0
97.7500000000051,0,853.063068885162,0,0
97.7750000000051,0,853.063068885162,0,0
97.8000000000051,0,853.063068885162,0,0
97.8250000000051,0,853.063068885162,0,0
97.8500000000051,0,853.063068885162,0,0
97.8750000000051,1,853.145193885162,0,0
97.9000000000051,0,853.227318885162,0,0
97.9250000000051,0,853.227318885162,0,0
97.9500000000051,0,853.227318885162,0,0
97.9750000000052,0,853.227318885162,0,0
98.0000000000052,0,853.227318885162,0,0
98.0250000000052,1,853.309443885162,0,0
98.0500000000052,0,853.391568885162,0,0
98.0750000000052,0,853.391568885162,0,0
98.1000000000052,1,853.473693885162,0,0
98.1250000000052,0,853.555818885162,0,0
98.1500000000052,0,853.555818885162,0,0
98.1750000000052,0,853.555818885162,0,0
98.2000000000052,0,853.555818885162,0,0
98.2250000000052,0,853.555818885162,0,0
98.2500000000052,0,853.555818885162,0,0
98.2750000000052,0,853.555818885162,0,0
98.3000000000052,0,853.555818885162,0,0
98.3250000000052,0,853.555818885162,0,0
98.3500000000052,0,853.555818885162,0,0
98.3750000000052,0,853.555818885162,0,0
98.4000000000052,0,853.555818885162,0,0
98.4250000000053,0,853.555818885162,0,0
98.4500000000053,0,853.555818885162,0,0
98.4750000000053,0,853.555818885162,0,0
98.5000000000053,0,853.555818885162,0,0
98.5250000000053,0,853.555818885162,0,0
98.5500000000053,0,853.555818885162,0,0
98.5750000000053,0,853.555818885162,0,0
98.6000000000053,0,853.555818885162,0,0
98.6250000000053,0,853.555818885162,0,0
98.6500000000053,0,853.555818885162,0,0
98.6750000000053,0,853.555818885162,0,0
98.7000000000053,0,853.555818885162,0,0
98.7250000000053,0,853.555818885162,0,0

98.7500000000053,0,853.555818885162,0,0
98.7750000000053,0,853.555818885162,0,0
98.8000000000053,0,853.555818885162,0,0
98.8250000000053,0,853.555818885162,0,0
98.8500000000054,0,853.555818885162,0,0
98.8750000000054,0,853.555818885162,0,0
98.9000000000054,1,853.637943885162,0,0
98.9250000000054,1,853.802193885162,0,0
98.9500000000054,0,853.884318885162,0,0
98.9750000000054,0,853.884318885162,0,0
99.0000000000054,1,853.966443885162,0,0
99.0250000000054,1,854.130693885162,0,0
99.0500000000054,0,854.212818885163,0,0
99.0750000000054,1,854.294943885163,0,0
99.1000000000054,0,854.377068885163,0,0
99.1250000000054,0,854.377068885163,0,0
99.1500000000054,0,854.377068885163,0,0
99.1750000000054,0,854.377068885163,0,0
99.2000000000054,0,854.377068885163,0,0
99.2250000000054,0,854.377068885163,0,0
99.2500000000054,0,854.377068885163,0,0
99.2750000000054,1,854.459193885163,0,0
99.3000000000055,0,854.541318885163,0,0
99.3250000000055,0,854.541318885163,0,0
99.3500000000055,0,854.541318885163,0,0
99.3750000000055,0,854.541318885163,0,0
99.4000000000055,1,854.623443885163,0,0
99.4250000000055,1,854.787693885163,0,0
99.4500000000055,1,854.951943885163,0,0
99.4750000000055,0,855.034068885163,0,0
99.5000000000055,0,855.034068885163,0,0
99.5250000000055,0,855.034068885163,0,0
99.5500000000055,0,855.034068885163,0,0
99.5750000000055,0,855.034068885163,0,0
99.6000000000055,0,855.034068885163,0,0
99.6250000000055,0,855.034068885163,0,0
99.6500000000055,0,855.034068885163,0,0
99.6750000000055,0,855.034068885163,0,0
99.7000000000055,0,855.034068885163,0,0
99.7250000000056,0,855.034068885163,0,0
99.7500000000056,0,855.034068885163,0,0
99.7750000000056,0,855.034068885163,0,0
99.8000000000056,0,855.034068885163,0,0
99.8250000000056,0,855.034068885163,0,0
99.8500000000056,0,855.034068885163,0,0
99.8750000000056,0,855.034068885163,0,0
99.9000000000056,1,855.116193885163,0,0
99.9250000000056,0,855.198318885163,0,0
99.9500000000056,0,855.198318885163,0,0
99.9750000000056,0,855.198318885163,0,0
100.000000000006,0,855.198318885163,0,0
100.025000000006,0,855.198318885163,0,0

100.050000000006,0,855.198318885163,0,0
100.075000000006,0,855.198318885163,0,0
100.100000000006,0,855.198318885163,0,0
100.125000000006,0,855.198318885163,0,0
100.150000000006,0,855.198318885163,0,0
100.175000000006,0,855.198318885163,0,0
100.200000000006,1,855.280443885163,0,0
100.225000000006,0,855.362568885163,0,0
100.250000000006,0,855.362568885163,0,0
100.275000000006,0,855.362568885163,0,0
100.300000000006,0,855.362568885163,0,0
100.325000000006,0,855.362568885163,0,0
100.350000000006,0,855.362568885163,0,0
100.375000000006,0,855.362568885163,0,0
100.400000000006,1,855.444693885163,0,0
100.425000000006,0,855.526818885163,0,0
100.450000000006,8,855.759103462783,0,0
100.475000000006,10,856.251090093244,25.5758574474481,1
100.500000000006,13,856.806898044583,25.5758574474481,1
100.525000000006,14,857.410287555969,25.5758574474481,1
100.550000000006,19,858.075545744596,25.5758574474481,1
100.575000000006,16,858.762020320334,25.5758574474481,1
100.600000000006,16,859.419020320334,25.5758574474481,1
100.625000000006,15,860.065589077641,25.5758574474481,1
100.650000000006,16,860.712157834949,25.5758574474481,1
100.675000000006,22,861.425858229224,25.5758574474481,1
100.700000000006,25,862.2216836235,25.5758574474481,1
100.725000000006,14,862.939592236389,25.5758574474481,1
100.750000000006,17,863.585485898781,25.5758574474481,1
100.775000000006,21,864.300439977233,25.5758574474481,1
100.800000000006,19,865.03475858192,25.5758574474481,1
100.825000000006,19,865.750707733396,25.5758574474481,1
100.850000000006,18,866.457109175564,25.5758574474481,1
100.875000000006,18,867.153962908424,25.5758574474481,1
100.900000000006,16,867.830889774853,25.5758574474481,1
100.925000000006,18,868.507816641283,25.5758574474481,1
100.950000000006,19,869.214218083451,25.5758574474481,1
100.975000000006,17,869.910802708693,25.5758574474481,1
101.000000000006,15,870.567481515504,25.5758574474481,1
101.025000000006,14,871.1928338857,25.5758574474481,1
101.050000000006,13,871.796223397087,25.5758574474481,1
101.075000000006,11,872.364707106492,25.5758574474481,1
101.100000000006,12,872.921574262543,25.5758574474481,1
101.125000000006,12,873.490552952829,25.5758574474481,1
101.150000000006,10,874.034744350814,25.5758574474481,1
101.175000000006,10,874.554148456497,25.5758574474481,1
101.200000000006,9,875.060225509338,25.5758574474481,1
101.225000000006,10,875.566302562179,25.5758574474481,1
101.250000000006,9,876.072379615021,25.5758574474481,1
101.275000000006,8,876.55103919264,0,0
101.300000000006,6,876.984488115386,0,0
101.325000000006,8,877.417937038132,0,0

101.350000000006,8,877.882506193371,0,0
101.375000000006,8,878.347075348611,0,0
101.400000000006,7,878.796642252652,0,0
101.425000000006,8,879.246209156693,0,0
101.450000000006,8,879.710778311932,0,0
101.475000000006,7,880.160345215973,0,0
101.500000000006,8,880.609912120014,0,0
101.525000000006,9,881.088571697634,0,0
101.550000000006,10,881.594648750475,0,1
101.575000000006,9,882.100725803317,0,0
101.600000000006,9,882.593475803317,0,0
101.625000000006,8,883.072135380936,0,0
101.650000000006,8,883.536704536176,0,0
101.675000000006,8,884.001273691416,0,0
101.700000000006,8,884.465842846655,0,0
101.725000000006,8,884.930412001895,0,0
101.750000000006,8,885.394981157134,0,0
101.775000000006,8,885.859550312374,0,0
101.800000000006,11,886.364212700902,22.0992203334403,1
101.825000000006,14,886.943874124698,22.0992203334403,1
101.850000000006,16,887.579657737587,22.0992203334403,1
101.875000000006,15,888.226226494894,22.0992203334403,1
101.900000000006,14,888.85157886509,22.0992203334403,1
101.925000000006,13,889.454968376477,22.0992203334403,1
101.950000000006,13,890.047180173472,22.0992203334403,1
101.975000000006,14,890.650569684858,22.0992203334403,1
102.000000000006,12,891.24234264289,22.0992203334403,1
102.025000000006,12,891.811321333176,22.0992203334403,1
102.050000000006,11,892.368188489227,22.0992203334403,1
102.075000000006,10,892.900268352977,22.0992203334403,1
102.100000000006,10,893.419672458659,22.0992203334403,1
102.125000000006,10,893.939076564342,22.0992203334403,1
102.150000000006,10,894.458480670025,22.0992203334403,1
102.175000000006,11,894.990560533774,22.0992203334403,1
102.200000000006,11,895.53531615559,22.0992203334403,1
102.225000000006,10,896.067396019339,22.0992203334403,1
102.250000000006,10,896.586800125021,22.0992203334403,1
102.275000000006,10,897.106204230704,22.0992203334403,1
102.300000000006,10,897.625608336387,22.0992203334403,1
102.325000000006,10,898.145012442069,22.0992203334403,1
102.350000000006,10,898.664416547752,22.0992203334403,1
102.375000000006,10,899.183820653435,22.0992203334403,1
102.400000000006,10,899.703224759117,22.0992203334403,1
102.425000000006,10,900.2226288648,22.0992203334403,1
102.450000000006,9,900.728705917641,22.0992203334403,1
102.475000000006,8,901.207365495261,0,0
102.500000000006,8,901.6719346505,0,0
102.525000000006,8,902.13650380574,0,0
102.550000000006,8,902.60107296098,0,0
102.575000000006,8,903.065642116219,0,0
102.600000000006,8,903.530211271459,0,0
102.625000000006,8,903.994780426698,0,0

102.650000000006,7,904.444347330739,0,0
102.675000000006,7,904.878911983582,0,0
102.700000000006,6,905.297358655129,0,0
102.725000000006,7,905.715805326676,0,0
102.750000000006,6,906.134251998223,0,0
102.775000000006,6,906.536580688476,0,0
102.800000000006,6,906.938909378728,0,0
102.825000000006,6,907.34123806898,0,0
102.850000000006,5,907.726039496758,0,0
102.875000000006,4,908.07392657941,0,0
102.900000000006,4,908.40242657941,0,0
102.925000000006,4,908.73092657941,0,0
102.950000000006,3,909.037421251982,0,0
102.975000000006,3,909.321910597125,0,0
103.000000000006,3,909.606399942268,0,0
103.025000000006,3,909.890889287411,0,0
103.050000000006,3,910.175378632555,0,0
103.075000000006,3,910.459867977698,0,0
103.100000000006,3,910.744357322841,0,0
103.125000000006,1,910.968726995413,0,0
103.150000000006,0,911.050851995413,0,0
103.175000000006,2,911.166994284223,0,0
103.200000000006,0,911.283136573033,0,0
103.225000000006,0,911.283136573033,0,0
103.250000000006,0,911.283136573033,0,0
103.275000000006,0,911.283136573033,0,0
103.300000000006,0,911.283136573033,0,0
103.325000000006,1,911.365261573033,0,0
103.350000000006,1,911.529511573033,0,0
103.375000000006,0,911.611636573033,0,0
103.400000000006,1,911.693761573033,0,0
103.425000000006,0,911.775886573033,0,0
103.450000000006,0,911.775886573033,0,0
103.475000000006,0,911.775886573033,0,0
103.500000000006,0,911.775886573033,0,0
103.525000000006,0,911.775886573033,0,0
103.550000000006,1,911.858011573033,0,0
103.575000000006,0,911.940136573033,0,0
103.600000000006,0,911.940136573033,0,0
103.625000000006,0,911.940136573033,0,0
103.650000000006,0,911.940136573033,0,0
103.675000000006,0,911.940136573033,0,0
103.700000000006,0,911.940136573033,0,0
103.725000000006,0,911.940136573033,0,0
103.750000000006,1,912.022261573033,0,0
103.775000000006,0,912.104386573033,0,0
103.800000000006,1,912.186511573033,0,0
103.825000000006,1,912.350761573033,0,0
103.850000000006,0,912.432886573033,0,0
103.875000000006,1,912.515011573033,0,0
103.900000000007,0,912.597136573033,0,0
103.925000000007,0,912.597136573033,0,0

103.950000000007,0,912.597136573033,0,0
103.975000000007,0,912.597136573033,0,0
104.000000000007,1,912.679261573033,0,0
104.025000000007,0,912.761386573033,0,0
104.050000000007,0,912.761386573033,0,0
104.075000000007,0,912.761386573033,0,0
104.100000000007,0,912.761386573033,0,0
104.125000000007,1,912.843511573033,0,0
104.150000000007,0,912.925636573033,0,0
104.175000000007,0,912.925636573033,0,0
104.200000000007,0,912.925636573033,0,0
104.225000000007,0,912.925636573033,0,0
104.250000000007,1,913.007761573033,0,0
104.275000000007,0,913.089886573033,0,0
104.300000000007,1,913.172011573033,0,0
104.325000000007,0,913.254136573033,0,0
104.350000000007,0,913.254136573033,0,0
104.375000000007,0,913.254136573033,0,0
104.400000000007,4,913.418386573033,0,0
104.425000000007,0,913.582636573033,0,0
104.450000000007,0,913.582636573033,0,0
104.475000000007,0,913.582636573033,0,0
104.500000000007,0,913.582636573033,0,0
104.525000000007,0,913.582636573033,0,0
104.550000000007,1,913.664761573033,0,0
104.575000000007,1,913.829011573033,0,0
104.600000000007,1,913.993261573033,0,0
104.625000000007,1,914.157511573033,0,0
104.650000000007,1,914.321761573033,0,0
104.675000000007,1,914.486011573033,0,0
104.700000000007,1,914.650261573033,0,0
104.725000000007,1,914.814511573033,0,0
104.750000000007,1,914.978761573033,0,0
104.775000000007,0,915.060886573033,0,0
104.800000000007,1,915.143011573034,0,0
104.825000000007,0,915.225136573034,0,0
104.850000000007,0,915.225136573034,0,0
104.875000000007,1,915.307261573034,0,0
104.900000000007,1,915.471511573034,0,0
104.925000000007,0,915.553636573034,0,0
104.950000000007,0,915.553636573034,0,0
104.975000000007,1,915.635761573034,0,0
105.000000000007,0,915.717886573034,0,0
105.025000000007,0,915.717886573034,0,0
105.050000000007,0,915.717886573034,0,0
105.075000000007,0,915.717886573034,0,0
105.100000000007,1,915.800011573034,0,0
105.125000000007,0,915.882136573034,0,0
105.150000000007,0,915.882136573034,0,0
105.175000000007,1,915.964261573034,0,0
105.200000000007,1,916.128511573034,0,0
105.225000000007,0,916.210636573034,0,0

105.250000000007,0,916.210636573034,0,0
105.275000000007,0,916.210636573034,0,0
105.300000000007,0,916.210636573034,0,0
105.325000000007,0,916.210636573034,0,0
105.350000000007,0,916.210636573034,0,0
105.375000000007,0,916.210636573034,0,0
105.400000000007,0,916.210636573034,0,0
105.425000000007,0,916.210636573034,0,0
105.450000000007,0,916.210636573034,0,0
105.475000000007,0,916.210636573034,0,0
105.500000000007,0,916.210636573034,0,0
105.525000000007,0,916.210636573034,0,0
105.550000000007,0,916.210636573034,0,0
105.575000000007,1,916.292761573034,0,0
105.600000000007,1,916.457011573034,0,0
105.625000000007,0,916.539136573034,0,0
105.650000000007,0,916.539136573034,0,0
105.675000000007,0,916.539136573034,0,0
105.700000000007,1,916.621261573034,0,0
105.725000000007,0,916.703386573034,0,0
105.750000000007,0,916.703386573034,0,0
105.775000000007,0,916.703386573034,0,0
105.800000000007,0,916.703386573034,0,0
105.825000000007,0,916.703386573034,0,0
105.850000000007,0,916.703386573034,0,0
105.875000000007,0,916.703386573034,0,0
105.900000000007,0,916.703386573034,0,0
105.925000000007,1,916.785511573034,0,0
105.950000000007,0,916.867636573034,0,0
105.975000000007,0,916.867636573034,0,0
106.000000000007,0,916.867636573034,0,0
106.025000000007,1,916.949761573034,0,0
106.050000000007,0,917.031886573034,0,0
106.075000000007,0,917.031886573034,0,0
106.100000000007,0,917.031886573034,0,0
106.125000000007,0,917.031886573034,0,0
106.150000000007,1,917.114011573034,0,0
106.175000000007,0,917.196136573034,0,0
106.200000000007,0,917.196136573034,0,0
106.225000000007,1,917.278261573034,0,0
106.250000000007,1,917.442511573034,0,0
106.275000000007,0,917.524636573034,0,0
106.300000000007,0,917.524636573034,0,0
106.325000000007,0,917.524636573034,0,0
106.350000000007,0,917.524636573034,0,0
106.375000000007,0,917.524636573034,0,0
106.400000000007,0,917.524636573034,0,0
106.425000000007,0,917.524636573034,0,0
106.450000000007,0,917.524636573034,0,0
106.475000000007,1,917.606761573034,0,0
106.500000000007,0,917.688886573034,0,0
106.525000000007,0,917.688886573034,0,0

106.550000000007,0,917.688886573034,0,0
106.575000000007,0,917.688886573034,0,0
106.600000000007,0,917.688886573034,0,0
106.625000000007,0,917.688886573034,0,0
106.650000000007,0,917.688886573034,0,0
106.675000000007,1,917.771011573034,0,0
106.700000000007,1,917.935261573034,0,0
106.725000000007,0,918.017386573034,0,0
106.750000000007,1,918.099511573034,0,0
106.775000000007,0,918.181636573034,0,0
106.800000000007,0,918.181636573034,0,0
106.825000000007,0,918.181636573034,0,0
106.850000000007,0,918.181636573034,0,0
106.875000000007,0,918.181636573034,0,0
106.900000000007,1,918.263761573034,0,0
106.925000000007,1,918.428011573034,0,0
106.950000000007,1,918.592261573034,0,0
106.975000000007,0,918.674386573034,0,0
107.000000000007,0,918.674386573034,0,0
107.025000000007,0,918.674386573034,0,0
107.050000000007,1,918.756511573034,0,0
107.075000000007,0,918.838636573034,0,0
107.100000000007,1,918.920761573034,0,0
107.125000000007,1,919.085011573034,0,0
107.150000000007,0,919.167136573034,0,0
107.175000000007,0,919.167136573034,0,0
107.200000000007,0,919.167136573034,0,0
107.225000000007,0,919.167136573034,0,0
107.250000000007,0,919.167136573034,0,0
107.275000000007,1,919.249261573034,0,0
107.300000000007,0,919.331386573034,0,0
107.325000000007,1,919.413511573035,0,0
107.350000000007,0,919.495636573035,0,0
107.375000000007,1,919.577761573035,0,0
107.400000000007,1,919.742011573035,0,0
107.425000000007,0,919.824136573035,0,0
107.450000000007,0,919.824136573035,0,0
107.475000000007,0,919.824136573035,0,0
107.500000000007,0,919.824136573035,0,0
107.525000000007,0,919.824136573035,0,0
107.550000000007,0,919.824136573035,0,0
107.575000000007,0,919.824136573035,0,0
107.600000000007,0,919.824136573035,0,0
107.625000000007,0,919.824136573035,0,0
107.650000000007,0,919.824136573035,0,0
107.675000000007,0,919.824136573035,0,0
107.700000000007,0,919.824136573035,0,0
107.725000000007,0,919.824136573035,0,0
107.750000000007,0,919.824136573035,0,0
107.775000000007,0,919.824136573035,0,0
107.800000000007,0,919.824136573035,0,0
107.825000000007,0,919.824136573035,0,0

```
107.850000000007,0,919.824136573035,0,0
107.875000000007,0,919.824136573035,0,0
107.900000000007,0,919.824136573035,0,0
107.925000000007,1,919.906261573035,0,0
107.950000000007,9,920.234761573035,0,0
107.975000000007,24,920.883465263287,45.8572546233531,1
108.000000000007,40,921.805198059221,45.8572546233531,1
108.025000000007,51,922.911091974598,45.8572546233531,1
108.050000000007,55,924.106637085045,45.8572546233531,1
108.075000000007,53,925.313571410455,45.8572546233531,1
108.100000000007,51,926.497940244806,45.8572546233531,1
108.125000000007,52,927.676641851495,45.8572546233531,1
108.150000000007,51,928.855343458184,45.8572546233531,1
108.175000000007,47,930.004853901936,45.8572546233531,1
108.200000000007,45,931.118785783951,45.8572546233531,1
108.225000000007,51,932.256186841601,45.8572546233531,1
108.250000000007,52,933.43488844829,45.8572546233531,1
108.275000000007,46,934.584099095149,45.8572546233531,1
108.300000000008,53,935.738976969671,45.8572546233531,1
108.325000000008,59,936.967670088872,45.8572546233531,1
108.350000000008,63,938.250331162679,45.8572546233531,1
108.375000000008,65,939.564291059521,45.8572546233531,1
108.400000000008,71,940.918401527223,45.8572546233531,1
108.425000000008,89,942.385164777815,45.8572546233531,1
108.450000000008,106,944.005459103614,45.8572546233531,1
108.475000000008,123,945.761798039531,45.8572546233531,1
108.500000000008,119,947.568486332534,45.8572546233531,1
108.525000000008,117,949.352682010441,45.8572546233531,1
108.550000000008,110,951.102333972494,45.8572546233531,1
108.575000000008,103,952.797145958837,45.8572546233531,1
108.600000000008,87,954.396634683355,45.8572546233531,1
108.625000000008,83,955.910840045777,45.8572546233531,1
108.650000000008,73,957.360710711048,45.8572546233531,1
108.675000000008,63,958.714233997896,45.8572546233531,1
108.700000000008,57,959.986111130156,45.8572546233531,1
108.725000000008,48,961.175119973439,45.8572546233531,1
108.750000000008,43,962.282628302534,45.8572546233531,1
108.775000000008,37,963.320704814143,45.8572546233531,1
108.800000000008,31,964.277504335241,45.8572546233531,1
108.825000000008,27,965.161491001253,45.8572546233531,1
108.850000000008,24,965.99055370922,45.8572546233531,1
108.875000000008,20,966.760156564776,45.8572546233531,1
108.900000000008,17,967.466040779585,45.8572546233531,1
108.925000000008,13,968.100756727586,45.8572546233531,1
108.950000000008,11,968.669240436991,45.8572546233531,1
108.975000000008,12,969.226107593042,45.8572546233531,1
109.000000000008,11,969.782974749094,45.8572546233531,1
109.025000000008,10,970.315054612843,45.8572546233531,1
109.050000000008,10,970.834458718525,45.8572546233531,1
109.075000000008,8,971.326445348987,45.8572546233531,1
109.100000000008,7,971.776012253027,0,0
109.125000000008,7,972.21057690587,0,0
```

109.150000000008,6,972.629023577417,0,0
109.175000000008,6,973.031352267669,0,0
109.200000000008,5,973.416153695448,0,0
109.225000000008,5,973.783427860752,0,0
109.250000000008,5,974.150702026056,0,0
109.275000000008,4,974.498589108708,0,0
109.300000000008,4,974.827089108708,0,0
109.325000000008,2,975.107481397518,0,0
109.350000000008,0,975.223623686328,0,0
109.375000000008,0,975.223623686328,0,0
109.400000000008,1,975.305748686328,0,0
109.425000000008,1,975.469998686328,0,0
109.450000000008,0,975.552123686328,0,0
109.475000000008,0,975.552123686328,0,0
109.500000000008,0,975.552123686328,0,0
109.525000000008,0,975.552123686328,0,0
109.550000000008,0,975.552123686328,0,0
109.575000000008,0,975.552123686328,0,0
109.600000000008,0,975.552123686328,0,0
109.625000000008,0,975.552123686328,0,0
109.650000000008,0,975.552123686328,0,0
109.675000000008,0,975.552123686328,0,0
109.700000000008,0,975.552123686328,0,0
109.725000000008,0,975.552123686328,0,0
109.750000000008,0,975.552123686328,0,0
109.775000000008,0,975.552123686328,0,0
109.800000000008,0,975.552123686328,0,0
109.825000000008,0,975.552123686328,0,0
109.850000000008,0,975.552123686328,0,0
109.875000000008,0,975.552123686328,0,0
109.900000000008,0,975.552123686328,0,0
109.925000000008,0,975.552123686328,0,0
109.950000000008,0,975.552123686328,0,0
109.975000000008,0,975.552123686328,0,0
110.000000000008,0,975.552123686328,0,0
110.025000000008,0,975.552123686328,0,0
110.050000000008,0,975.552123686328,0,0
110.075000000008,0,975.552123686328,0,0
110.100000000008,1,975.634248686328,0,0
110.125000000008,0,975.716373686328,0,0
110.150000000008,0,975.716373686328,0,0
110.175000000008,0,975.716373686328,0,0
110.200000000008,0,975.716373686328,0,0
110.225000000008,0,975.716373686328,0,0
110.250000000008,0,975.716373686328,0,0
110.275000000008,0,975.716373686328,0,0
110.300000000008,0,975.716373686328,0,0
110.325000000008,0,975.716373686328,0,0
110.350000000008,0,975.716373686328,0,0
110.375000000008,0,975.716373686328,0,0
110.400000000008,0,975.716373686328,0,0
110.425000000008,0,975.716373686328,0,0

110.450000000008,0,975.716373686328,0,0
110.475000000008,0,975.716373686328,0,0
110.500000000008,0,975.716373686328,0,0
110.525000000008,0,975.716373686328,0,0
110.550000000008,0,975.716373686328,0,0
110.575000000008,0,975.716373686328,0,0
110.600000000008,0,975.716373686328,0,0
110.625000000008,0,975.716373686328,0,0
110.650000000008,0,975.716373686328,0,0
110.675000000008,0,975.716373686328,0,0
110.700000000008,0,975.716373686328,0,0
110.725000000008,0,975.716373686328,0,0
110.750000000008,0,975.716373686328,0,0
110.775000000008,0,975.716373686328,0,0
110.800000000008,0,975.716373686328,0,0
110.825000000008,0,975.716373686328,0,0
110.850000000008,0,975.716373686328,0,0
110.875000000008,0,975.716373686328,0,0
110.900000000008,0,975.716373686328,0,0
110.925000000008,0,975.716373686328,0,0
110.950000000008,0,975.716373686328,0,0
110.975000000008,0,975.716373686328,0,0
111.000000000008,0,975.716373686328,0,0
111.025000000008,0,975.716373686328,0,0
111.050000000008,0,975.716373686328,0,0
111.075000000008,0,975.716373686328,0,0
111.100000000008,0,975.716373686328,0,0
111.125000000008,1,975.798498686328,0,0
111.150000000008,0,975.880623686328,0,0
111.175000000008,0,975.880623686328,0,0
111.200000000008,0,975.880623686328,0,0
111.225000000008,0,975.880623686328,0,0
111.250000000008,0,975.880623686328,0,0
111.275000000008,0,975.880623686328,0,0
111.300000000008,0,975.880623686328,0,0
111.325000000008,0,975.880623686328,0,0
111.350000000008,0,975.880623686328,0,0
111.375000000008,0,975.880623686328,0,0
111.400000000008,0,975.880623686328,0,0
111.425000000008,0,975.880623686328,0,0
111.450000000008,0,975.880623686328,0,0
111.475000000008,0,975.880623686328,0,0
111.500000000008,0,975.880623686328,0,0
111.525000000008,0,975.880623686328,0,0
111.550000000008,0,975.880623686328,0,0
111.575000000008,0,975.880623686328,0,0
111.600000000008,1,975.962748686328,0,0
111.625000000008,5,976.228510768981,0,0
111.650000000008,1,976.494272851633,0,0
111.675000000008,0,976.576397851633,0,0
111.700000000008,0,976.576397851633,0,0
111.725000000008,0,976.576397851633,0,0

111.750000000008,0,976.576397851633,0,0
111.775000000008,0,976.576397851633,0,0
111.800000000008,3,976.718642524204,0,0
111.825000000008,2,976.977029485586,0,0
111.850000000008,1,977.175296774396,0,0
111.875000000008,0,977.257421774396,0,0
111.900000000008,1,977.339546774396,0,0
111.925000000008,0,977.421671774396,0,0
111.950000000008,2,977.537814063206,0,0
111.975000000008,5,977.837593434668,0,0
112.000000000008,0,978.02123051732,0,0
112.025000000008,0,978.02123051732,0,0
112.050000000008,0,978.02123051732,0,0
112.075000000008,0,978.02123051732,0,0
112.100000000008,0,978.02123051732,0,0
112.125000000008,0,978.02123051732,0,0
112.150000000008,0,978.02123051732,0,0
112.175000000008,0,978.02123051732,0,0
112.200000000008,0,978.02123051732,0,0
112.225000000008,0,978.02123051732,0,0
112.250000000008,0,978.02123051732,0,0
112.275000000008,0,978.02123051732,0,0
112.300000000008,0,978.02123051732,0,0
112.325000000008,0,978.02123051732,0,0
112.350000000008,0,978.02123051732,0,0
112.375000000008,1,978.10335551732,0,0
112.400000000008,1,978.26760551732,0,0
112.425000000008,0,978.34973051732,0,0
112.450000000008,1,978.43185551732,0,0
112.475000000008,0,978.51398051732,0,0
112.500000000008,0,978.51398051732,0,0
112.525000000008,0,978.51398051732,0,0
112.550000000008,0,978.51398051732,0,0
112.575000000008,0,978.51398051732,0,0
112.600000000008,0,978.51398051732,0,0
112.625000000008,0,978.51398051732,0,0
112.650000000008,0,978.51398051732,0,0
112.675000000009,0,978.51398051732,0,0
112.700000000009,0,978.51398051732,0,0
112.725000000009,0,978.51398051732,0,0
112.750000000009,0,978.51398051732,0,0
112.775000000009,0,978.51398051732,0,0
112.800000000009,0,978.51398051732,0,0
112.825000000009,0,978.51398051732,0,0
112.850000000009,0,978.51398051732,0,0
112.875000000009,0,978.51398051732,0,0
112.900000000009,0,978.51398051732,0,0
112.925000000009,0,978.51398051732,0,0
112.950000000009,0,978.51398051732,0,0
112.975000000009,0,978.51398051732,0,0
113.000000000009,0,978.51398051732,0,0
113.025000000009,0,978.51398051732,0,0

113.050000000009,0,978.51398051732,0,0
113.075000000009,0,978.51398051732,0,0
113.100000000009,0,978.51398051732,0,0
113.125000000009,2,978.63012280613,0,0
113.150000000009,0,978.74626509494,0,0
113.175000000009,0,978.74626509494,0,0
113.200000000009,0,978.74626509494,0,0
113.225000000009,0,978.74626509494,0,0
113.250000000009,0,978.74626509494,0,0
113.275000000009,0,978.74626509494,0,0
113.300000000009,0,978.74626509494,0,0
113.325000000009,0,978.74626509494,0,0
113.350000000009,0,978.74626509494,0,0
113.375000000009,0,978.74626509494,0,0
113.400000000009,0,978.74626509494,0,0
113.425000000009,1,978.82839009494,0,0
113.450000000009,0,978.91051509494,0,0
113.475000000009,0,978.91051509494,0,0
113.500000000009,0,978.91051509494,0,0
113.525000000009,1,978.99264009494,0,0
113.550000000009,2,979.19090738375,0,0
113.575000000009,0,979.30704967256,0,0
113.600000000009,1,979.38917467256,0,0
113.625000000009,0,979.47129967256,0,0
113.650000000009,0,979.47129967256,0,0
113.675000000009,0,979.47129967256,0,0
113.700000000009,0,979.47129967256,0,0
113.725000000009,0,979.47129967256,0,0
113.750000000009,0,979.47129967256,0,0
113.775000000009,0,979.47129967256,0,0
113.800000000009,0,979.47129967256,0,0
113.825000000009,0,979.47129967256,0,0
113.850000000009,0,979.47129967256,0,0
113.875000000009,0,979.47129967256,0,0
113.900000000009,0,979.47129967256,0,0
113.925000000009,0,979.47129967256,0,0
113.950000000009,0,979.47129967256,0,0
113.975000000009,0,979.47129967256,0,0
114.000000000009,0,979.47129967256,0,0
114.025000000009,0,979.47129967256,0,0
114.050000000009,0,979.47129967256,0,0
114.075000000009,0,979.47129967256,0,0
114.100000000009,0,979.47129967256,0,0
114.125000000009,0,979.47129967256,0,0
114.150000000009,0,979.47129967256,0,0
114.175000000009,0,979.47129967256,0,0
114.200000000009,0,979.47129967256,0,0
114.225000000009,0,979.47129967256,0,0
114.250000000009,0,979.47129967256,0,0
114.275000000009,0,979.47129967256,0,0
114.300000000009,0,979.47129967256,0,0
114.325000000009,0,979.47129967256,0,0

114.350000000009,0,979.47129967256,0,0
114.375000000009,0,979.47129967256,0,0
114.400000000009,0,979.47129967256,0,0
114.425000000009,0,979.47129967256,0,0
114.450000000009,0,979.47129967256,0,0
114.475000000009,0,979.47129967256,0,0
114.500000000009,0,979.47129967256,0,0
114.525000000009,1,979.55342467256,0,0
114.550000000009,0,979.63554967256,0,0
114.575000000009,0,979.63554967256,0,0
114.600000000009,0,979.63554967256,0,0
114.625000000009,0,979.63554967256,0,0
114.650000000009,0,979.63554967256,0,0
114.675000000009,0,979.63554967256,0,0
114.700000000009,0,979.63554967256,0,0
114.725000000009,0,979.63554967256,0,0
114.750000000009,0,979.63554967256,0,0
114.775000000009,0,979.63554967256,0,0
114.800000000009,0,979.63554967256,0,0
114.825000000009,0,979.63554967256,0,0
114.850000000009,0,979.63554967256,0,0
114.875000000009,0,979.63554967256,0,0
114.900000000009,0,979.63554967256,0,0
114.925000000009,0,979.63554967256,0,0
114.950000000009,0,979.63554967256,0,0
114.975000000009,0,979.63554967256,0,0
115.000000000009,0,979.63554967256,0,0
115.025000000009,0,979.63554967256,0,0
115.050000000009,0,979.63554967256,0,0
115.075000000009,0,979.63554967256,0,0
115.100000000009,0,979.63554967256,0,0
115.125000000009,0,979.63554967256,0,0
115.150000000009,1,979.71767467256,0,0
115.175000000009,0,979.79979967256,0,0
115.200000000009,0,979.79979967256,0,0
115.225000000009,1,979.88192467256,0,0
115.250000000009,0,979.96404967256,0,0
115.275000000009,0,979.96404967256,0,0
115.300000000009,0,979.96404967256,0,0
115.325000000009,0,979.96404967256,0,0
115.350000000009,0,979.96404967256,0,0
115.375000000009,0,979.96404967256,0,0
115.400000000009,2,980.08019196137,0,0
115.425000000009,0,980.196334250179,0,0
115.450000000009,1,980.278459250179,0,0
115.475000000009,0,980.36058425018,0,0
115.500000000009,0,980.36058425018,0,0
115.525000000009,0,980.36058425018,0,0
115.550000000009,0,980.36058425018,0,0
115.575000000009,0,980.36058425018,0,0
115.600000000009,0,980.36058425018,0,0
115.625000000009,0,980.36058425018,0,0

115.650000000009,0,980.36058425018,0,0
115.675000000009,0,980.36058425018,0,0
115.700000000009,0,980.36058425018,0,0
115.725000000009,0,980.36058425018,0,0
115.750000000009,4,980.52483425018,0,0
115.775000000009,0,980.68908425018,0,0
115.800000000009,0,980.68908425018,0,0
115.825000000009,0,980.68908425018,0,0
115.850000000009,1,980.77120925018,0,0
115.875000000009,0,980.85333425018,0,0
115.900000000009,0,980.85333425018,0,0
115.925000000009,0,980.85333425018,0,0
115.950000000009,0,980.85333425018,0,0
115.975000000009,0,980.85333425018,0,0
116.000000000009,0,980.85333425018,0,0
116.025000000009,0,980.85333425018,0,0
116.050000000009,0,980.85333425018,0,0
116.075000000009,0,980.85333425018,0,0
116.100000000009,0,980.85333425018,0,0
116.125000000009,0,980.85333425018,0,0
116.150000000009,0,980.85333425018,0,0
116.175000000009,0,980.85333425018,0,0
116.200000000009,0,980.85333425018,0,0
116.225000000009,0,980.85333425018,0,0
116.250000000009,0,980.85333425018,0,0
116.275000000009,0,980.85333425018,0,0
116.300000000009,0,980.85333425018,0,0
116.325000000009,0,980.85333425018,0,0
116.350000000009,0,980.85333425018,0,0
116.375000000009,0,980.85333425018,0,0
116.400000000009,0,980.85333425018,0,0
116.425000000009,0,980.85333425018,0,0
116.450000000009,0,980.85333425018,0,0
116.475000000009,0,980.85333425018,0,0
116.500000000009,0,980.85333425018,0,0
116.525000000009,0,980.85333425018,0,0
116.550000000009,0,980.85333425018,0,0
116.575000000009,0,980.85333425018,0,0
116.600000000009,0,980.85333425018,0,0
116.625000000009,0,980.85333425018,0,0
116.650000000009,0,980.85333425018,0,0
116.675000000009,0,980.85333425018,0,0
116.700000000009,0,980.85333425018,0,0
116.725000000009,0,980.85333425018,0,0
116.750000000009,0,980.85333425018,0,0
116.775000000009,0,980.85333425018,0,0
116.800000000009,0,980.85333425018,0,0
116.825000000009,0,980.85333425018,0,0
116.850000000009,0,980.85333425018,0,0
116.875000000009,0,980.85333425018,0,0
116.900000000009,0,980.85333425018,0,0
116.925000000009,0,980.85333425018,0,0

116.950000000009,0,980.85333425018,0,0
116.975000000009,0,980.85333425018,0,0
117.000000000009,0,980.85333425018,0,0
117.025000000009,0,980.85333425018,0,0
117.050000000009,1,980.93545925018,0,0
117.07500000001,0,981.01758425018,0,0
117.10000000001,1,981.09970925018,0,0
117.12500000001,0,981.18183425018,0,0
117.15000000001,0,981.18183425018,0,0
117.17500000001,0,981.18183425018,0,0
117.20000000001,0,981.18183425018,0,0
117.22500000001,0,981.18183425018,0,0
117.25000000001,0,981.18183425018,0,0
117.27500000001,0,981.18183425018,0,0
117.30000000001,0,981.18183425018,0,0
117.32500000001,0,981.18183425018,0,0
117.35000000001,0,981.18183425018,0,0
117.37500000001,0,981.18183425018,0,0
117.40000000001,0,981.18183425018,0,0
117.42500000001,1,981.26395925018,0,0
117.45000000001,0,981.34608425018,0,0
117.47500000001,0,981.34608425018,0,0
117.50000000001,0,981.34608425018,0,0
117.52500000001,0,981.34608425018,0,0
117.55000000001,0,981.34608425018,0,0
117.57500000001,0,981.34608425018,0,0
117.60000000001,0,981.34608425018,0,0
117.62500000001,0,981.34608425018,0,0
117.65000000001,0,981.34608425018,0,0
117.67500000001,0,981.34608425018,0,0
117.70000000001,0,981.34608425018,0,0
117.72500000001,0,981.34608425018,0,0
117.75000000001,0,981.34608425018,0,0
117.77500000001,0,981.34608425018,0,0
117.80000000001,0,981.34608425018,0,0
117.82500000001,0,981.34608425018,0,0
117.85000000001,0,981.34608425018,0,0
117.87500000001,0,981.34608425018,0,0
117.90000000001,0,981.34608425018,0,0
117.92500000001,0,981.34608425018,0,0
117.95000000001,0,981.34608425018,0,0
117.97500000001,0,981.34608425018,0,0
118.00000000001,0,981.34608425018,0,0
118.02500000001,0,981.34608425018,0,0
118.05000000001,0,981.34608425018,0,0
118.07500000001,0,981.34608425018,0,0
118.10000000001,0,981.34608425018,0,0
118.12500000001,0,981.34608425018,0,0
118.15000000001,1,981.42820925018,0,0
118.17500000001,1,981.59245925018,0,0
118.20000000001,1,981.75670925018,0,0
118.22500000001,0,981.83883425018,0,0

118.25000000001,0,981.83883425018,0,0
118.27500000001,0,981.83883425018,0,0
118.30000000001,0,981.83883425018,0,0
118.32500000001,0,981.83883425018,0,0
118.35000000001,0,981.83883425018,0,0
118.37500000001,0,981.83883425018,0,0
118.40000000001,0,981.83883425018,0,0
118.42500000001,0,981.83883425018,0,0
118.45000000001,0,981.83883425018,0,0
118.47500000001,0,981.83883425018,0,0
118.50000000001,0,981.83883425018,0,0
118.52500000001,0,981.83883425018,0,0
118.55000000001,0,981.83883425018,0,0
118.57500000001,0,981.83883425018,0,0
118.60000000001,0,981.83883425018,0,0
118.62500000001,0,981.83883425018,0,0
118.65000000001,0,981.83883425018,0,0
118.67500000001,0,981.83883425018,0,0
118.70000000001,0,981.83883425018,0,0
118.72500000001,0,981.83883425018,0,0
118.75000000001,0,981.83883425018,0,0
118.77500000001,0,981.83883425018,0,0
118.80000000001,1,981.92095925018,0,0
118.82500000001,0,982.00308425018,0,0
118.85000000001,0,982.00308425018,0,0
118.87500000001,0,982.00308425018,0,0
118.90000000001,0,982.00308425018,0,0
118.92500000001,0,982.00308425018,0,0
118.95000000001,0,982.00308425018,0,0
118.97500000001,0,982.00308425018,0,0
119.00000000001,0,982.00308425018,0,0
119.02500000001,0,982.00308425018,0,0
119.05000000001,1,982.08520925018,0,0
119.07500000001,0,982.16733425018,0,0
119.10000000001,0,982.16733425018,0,0
119.12500000001,1,982.24945925018,0,0
119.15000000001,2,982.44772653899,0,0
119.17500000001,0,982.5638688278,0,0
119.20000000001,0,982.5638688278,0,0
119.22500000001,0,982.5638688278,0,0
119.25000000001,1,982.6459938278,0,0
119.27500000001,2,982.84426111661,0,0
119.30000000001,0,982.96040340542,0,0
119.32500000001,0,982.96040340542,0,0
119.35000000001,0,982.96040340542,0,0
119.37500000001,2,983.076545694229,0,0
119.40000000001,0,983.192687983039,0,0
119.42500000001,6,983.393852328165,0,0
119.45000000001,1,983.677141673291,0,0
119.47500000001,0,983.759266673291,0,0
119.50000000001,1,983.841391673291,0,0
119.52500000001,1,984.005641673292,0,0

119.55000000001,0,984.087766673292,0,0
119.57500000001,0,984.087766673292,0,0
119.60000000001,1,984.169891673292,0,0
119.62500000001,0,984.252016673292,0,0
119.65000000001,0,984.252016673292,0,0
119.67500000001,0,984.252016673292,0,0
119.70000000001,0,984.252016673292,0,0
119.72500000001,0,984.252016673292,0,0
119.75000000001,0,984.252016673292,0,0
119.77500000001,0,984.252016673292,0,0
119.80000000001,1,984.334141673292,0,0
119.82500000001,0,984.416266673292,0,0
119.85000000001,0,984.416266673292,0,0
119.87500000001,0,984.416266673292,0,0
119.90000000001,0,984.416266673292,0,0
119.92500000001,0,984.416266673292,0,0
119.95000000001,0,984.416266673292,0,0
119.97500000001,0,984.416266673292,0,0
120.00000000001,0,984.416266673292,0,0
120.02500000001,0,984.416266673292,0,0
120.05000000001,0,984.416266673292,0,0
120.07500000001,0,984.416266673292,0,0
120.10000000001,0,984.416266673292,0,0
120.12500000001,0,984.416266673292,0,0
120.15000000001,0,984.416266673292,0,0
120.17500000001,0,984.416266673292,0,0
120.20000000001,0,984.416266673292,0,0
120.22500000001,0,984.416266673292,0,0
120.25000000001,0,984.416266673292,0,0
120.27500000001,0,984.416266673292,0,0
120.30000000001,0,984.416266673292,0,0
120.32500000001,0,984.416266673292,0,0
120.35000000001,0,984.416266673292,0,0
120.37500000001,0,984.416266673292,0,0
120.40000000001,0,984.416266673292,0,0
120.42500000001,0,984.416266673292,0,0
120.45000000001,0,984.416266673292,0,0
120.47500000001,0,984.416266673292,0,0
120.50000000001,0,984.416266673292,0,0
120.52500000001,0,984.416266673292,0,0
120.55000000001,0,984.416266673292,0,0
120.57500000001,0,984.416266673292,0,0
120.60000000001,0,984.416266673292,0,0
120.62500000001,0,984.416266673292,0,0
120.65000000001,0,984.416266673292,0,0
120.67500000001,0,984.416266673292,0,0
120.70000000001,0,984.416266673292,0,0
120.72500000001,0,984.416266673292,0,0
120.75000000001,0,984.416266673292,0,0
120.77500000001,0,984.416266673292,0,0
120.80000000001,0,984.416266673292,0,0
120.82500000001,0,984.416266673292,0,0

120.85000000001,0,984.416266673292,0,0
120.87500000001,0,984.416266673292,0,0
120.90000000001,0,984.416266673292,0,0
120.92500000001,0,984.416266673292,0,0
120.95000000001,0,984.416266673292,0,0
120.97500000001,0,984.416266673292,0,0
121.00000000001,1,984.498391673292,0,0
121.02500000001,0,984.580516673292,0,0
121.05000000001,0,984.580516673292,0,0
121.07500000001,0,984.580516673292,0,0
121.10000000001,0,984.580516673292,0,0
121.12500000001,0,984.580516673292,0,0
121.15000000001,0,984.580516673292,0,0
121.17500000001,0,984.580516673292,0,0
121.20000000001,0,984.580516673292,0,0
121.22500000001,1,984.662641673292,0,0
121.25000000001,0,984.744766673292,0,0
121.27500000001,0,984.744766673292,0,0
121.30000000001,0,984.744766673292,0,0
121.32500000001,0,984.744766673292,0,0
121.35000000001,0,984.744766673292,0,0
121.37500000001,0,984.744766673292,0,0
121.40000000001,0,984.744766673292,0,0
121.42500000001,0,984.744766673292,0,0
121.45000000001,0,984.744766673292,0,0
121.475000000011,0,984.744766673292,0,0
121.500000000011,0,984.744766673292,0,0
121.525000000011,0,984.744766673292,0,0
121.550000000011,0,984.744766673292,0,0
121.575000000011,0,984.744766673292,0,0
121.600000000011,0,984.744766673292,0,0
121.625000000011,0,984.744766673292,0,0
121.650000000011,0,984.744766673292,0,0
121.675000000011,0,984.744766673292,0,0
121.700000000011,0,984.744766673292,0,0
121.725000000011,0,984.744766673292,0,0
121.750000000011,0,984.744766673292,0,0
121.775000000011,0,984.744766673292,0,0
121.800000000011,0,984.744766673292,0,0
121.825000000011,0,984.744766673292,0,0
121.850000000011,0,984.744766673292,0,0
121.875000000011,0,984.744766673292,0,0
121.900000000011,0,984.744766673292,0,0
121.925000000011,0,984.744766673292,0,0
121.950000000011,0,984.744766673292,0,0
121.975000000011,0,984.744766673292,0,0
122.000000000011,0,984.744766673292,0,0
122.025000000011,0,984.744766673292,0,0
122.050000000011,0,984.744766673292,0,0
122.075000000011,0,984.744766673292,0,0
122.100000000011,0,984.744766673292,0,0
122.125000000011,0,984.744766673292,0,0

122.150000000011,0,984.744766673292,0,0
122.175000000011,0,984.744766673292,0,0
122.200000000011,0,984.744766673292,0,0
122.225000000011,0,984.744766673292,0,0
122.250000000011,0,984.744766673292,0,0
122.275000000011,0,984.744766673292,0,0
122.300000000011,0,984.744766673292,0,0
122.325000000011,0,984.744766673292,0,0
122.350000000011,0,984.744766673292,0,0
122.375000000011,0,984.744766673292,0,0
122.400000000011,0,984.744766673292,0,0
122.425000000011,0,984.744766673292,0,0
122.450000000011,0,984.744766673292,0,0
122.475000000011,0,984.744766673292,0,0
122.500000000011,0,984.744766673292,0,0
122.525000000011,0,984.744766673292,0,0
122.550000000011,0,984.744766673292,0,0
122.575000000011,1,984.826891673292,0,0
122.600000000011,0,984.909016673292,0,0
122.625000000011,0,984.909016673292,0,0
122.650000000011,0,984.909016673292,0,0
122.675000000011,0,984.909016673292,0,0
122.700000000011,0,984.909016673292,0,0
122.725000000011,0,984.909016673292,0,0
122.750000000011,0,984.909016673292,0,0
122.775000000011,0,984.909016673292,0,0
122.800000000011,0,984.909016673292,0,0
122.825000000011,0,984.909016673292,0,0
122.850000000011,0,984.909016673292,0,0
122.875000000011,4,985.073266673292,0,0
122.900000000011,0,985.237516673292,0,0
122.925000000011,0,985.237516673292,0,0
122.950000000011,3,985.379761345863,0,0
122.975000000011,0,985.522006018435,0,0
123.000000000011,1,985.604131018435,0,0
123.025000000011,0,985.686256018435,0,0
123.050000000011,5,985.869893101087,0,0
123.075000000011,2,986.169672472549,0,0
123.100000000011,0,986.285814761359,0,0
123.125000000011,2,986.401957050169,0,0
123.150000000011,0,986.518099338979,0,0
123.175000000011,0,986.518099338979,0,0
123.200000000011,0,986.518099338979,0,0
123.225000000011,2,986.634241627789,0,0
123.250000000011,0,986.750383916599,0,0
123.275000000011,0,986.750383916599,0,0
123.300000000011,0,986.750383916599,0,0
123.325000000011,1,986.832508916599,0,0
123.350000000011,3,987.05687858917,0,0
123.375000000011,4,987.363373261742,0,0
123.400000000011,6,987.728787606868,0,0
123.425000000011,8,988.162236529614,0,0

123.450000000011,9,988.640896107233,0,0
123.475000000011,11,989.159648918141,24.0963172114887,1
123.500000000011,11,989.704404539957,24.0963172114887,1
123.525000000011,13,990.272888249363,24.0963172114887,1
123.550000000011,11,990.841371958768,24.0963172114887,1
123.575000000011,10,991.373451822517,24.0963172114887,1
123.600000000011,11,991.905531686267,24.0963172114887,1
123.625000000011,12,992.462398842318,24.0963172114887,1
123.650000000011,12,993.031377532604,24.0963172114887,1
123.675000000011,13,993.611972776245,24.0963172114887,1
123.700000000011,14,994.215362287631,24.0963172114887,1
123.725000000011,15,994.840714657827,24.0963172114887,1
123.750000000011,16,995.487283415134,24.0963172114887,1
123.775000000011,18,996.164210281564,24.0963172114887,1
123.800000000011,20,996.879911313298,24.0963172114887,1
123.825000000011,19,997.605160054341,24.0963172114887,1
123.850000000011,18,998.311561496508,24.0963172114887,1
123.875000000011,20,999.027262528243,24.0963172114887,1
123.900000000011,19,999.752511269285,24.0963172114887,1
123.925000000011,18,1000.45891271145,24.0963172114887,1
123.950000000011,17,1001.14594962739,24.0963172114887,1
123.975000000011,16,1001.81305967689,24.0963172114887,1
124.000000000011,16,1002.47005967689,24.0963172114887,1
124.025000000011,15,1003.1166284342,24.0963172114887,1
124.050000000011,15,1003.75276594881,24.0963172114887,1
124.075000000011,13,1004.36694060462,24.0963172114887,1
124.100000000011,13,1004.95915240161,24.0963172114887,1
124.125000000011,14,1005.562541913,24.0963172114887,1
124.150000000011,14,1006.17710913878,24.0963172114887,1
124.175000000011,13,1006.78049865016,24.0963172114887,1
124.200000000011,13,1007.37271044716,24.0963172114887,1
124.225000000011,13,1007.96492224415,24.0963172114887,1
124.250000000011,13,1008.55713404115,24.0963172114887,1
124.275000000011,12,1009.13772928479,24.0963172114887,1
124.300000000011,12,1009.70670797507,24.0963172114887,1
124.325000000011,12,1010.27568666536,24.0963172114887,1
124.350000000011,11,1010.83255382141,24.0963172114887,1
124.375000000011,11,1011.37730944323,24.0963172114887,1
124.400000000011,11,1011.92206506504,24.0963172114887,1
124.425000000011,11,1012.46682068686,24.0963172114887,1
124.450000000011,10,1012.99890055061,24.0963172114887,1
124.475000000011,10,1013.51830465629,24.0963172114887,1
124.500000000011,10,1014.03770876197,24.0963172114887,1
124.525000000011,10,1014.55711286766,24.0963172114887,1
124.550000000011,9,1015.0631899205,24.0963172114887,1
124.575000000011,8,1015.54184949812,0,0
124.600000000011,8,1016.00641865336,0,0
124.625000000011,7,1016.4559855574,0,0
124.650000000011,6,1016.87443222895,0,0
124.675000000011,6,1017.2767609192,0,0
124.700000000011,5,1017.66156234698,0,0
124.725000000011,4,1018.00944942963,0,0

124.750000000011,2,1018.28984171844,0,0
124.775000000011,2,1018.52212629606,0,0
124.800000000011,1,1018.72039358487,0,0
124.825000000011,1,1018.88464358487,0,0
124.850000000011,1,1019.04889358487,0,0
124.875000000011,0,1019.13101858487,0,0
124.900000000011,1,1019.21314358487,0,0
124.925000000011,0,1019.29526858487,0,0
124.950000000011,0,1019.29526858487,0,0
124.975000000011,1,1019.37739358487,0,0
125.000000000011,1,1019.54164358487,0,0
125.025000000011,1,1019.70589358487,0,0
125.050000000011,0,1019.78801858487,0,0
125.075000000011,1,1019.87014358487,0,0
125.100000000011,1,1020.03439358487,0,0
125.125000000011,0,1020.11651858487,0,0
125.150000000011,0,1020.11651858487,0,0
125.175000000011,0,1020.11651858487,0,0
125.200000000011,0,1020.11651858487,0,0
125.225000000011,0,1020.11651858487,0,0
125.250000000011,0,1020.11651858487,0,0
125.275000000011,0,1020.11651858487,0,0
125.300000000011,0,1020.11651858487,0,0
125.325000000011,0,1020.11651858487,0,0
125.350000000011,0,1020.11651858487,0,0
125.375000000011,0,1020.11651858487,0,0
125.400000000011,1,1020.19864358487,0,0
125.425000000011,0,1020.28076858487,0,0
125.450000000011,1,1020.36289358487,0,0
125.475000000011,1,1020.52714358487,0,0
125.500000000011,0,1020.60926858487,0,0
125.525000000011,0,1020.60926858487,0,0
125.550000000011,0,1020.60926858487,0,0
125.575000000011,1,1020.69139358487,0,0
125.600000000011,0,1020.77351858487,0,0
125.625000000011,0,1020.77351858487,0,0
125.650000000011,1,1020.85564358487,0,0
125.675000000011,0,1020.93776858487,0,0
125.700000000011,0,1020.93776858487,0,0
125.725000000011,0,1020.93776858487,0,0
125.750000000011,1,1021.01989358487,0,0
125.775000000011,0,1021.10201858487,0,0
125.800000000011,1,1021.18414358487,0,0
125.825000000011,0,1021.26626858487,0,0
125.850000000011,1,1021.34839358487,0,0
125.875000000012,1,1021.51264358487,0,0
125.900000000012,0,1021.59476858487,0,0
125.925000000012,0,1021.59476858487,0,0
125.950000000012,0,1021.59476858487,0,0
125.975000000012,0,1021.59476858487,0,0
126.000000000012,0,1021.59476858487,0,0
126.025000000012,0,1021.59476858487,0,0

126.050000000012,0,1021.59476858487,0,0
126.075000000012,0,1021.59476858487,0,0
126.100000000012,0,1021.59476858487,0,0
126.125000000012,0,1021.59476858487,0,0
126.150000000012,0,1021.59476858487,0,0
126.175000000012,0,1021.59476858487,0,0
126.200000000012,0,1021.59476858487,0,0
126.225000000012,0,1021.59476858487,0,0
126.250000000012,0,1021.59476858487,0,0
126.275000000012,0,1021.59476858487,0,0
126.300000000012,0,1021.59476858487,0,0
126.325000000012,0,1021.59476858487,0,0
126.350000000012,1,1021.67689358487,0,0
126.375000000012,0,1021.75901858487,0,0
126.400000000012,0,1021.75901858487,0,0
126.425000000012,1,1021.84114358487,0,0
126.450000000012,1,1022.00539358487,0,0
126.475000000012,1,1022.16964358487,0,0
126.500000000012,0,1022.25176858487,0,0
126.525000000012,0,1022.25176858487,0,0
126.550000000012,0,1022.25176858487,0,0
126.575000000012,0,1022.25176858487,0,0
126.600000000012,0,1022.25176858487,0,0
126.625000000012,3,1022.39401325744,0,0
126.650000000012,2,1022.65240021882,0,0
126.675000000012,0,1022.76854250763,0,0
126.700000000012,0,1022.76854250763,0,0
126.725000000012,1,1022.85066750763,0,0
126.750000000012,2,1023.04893479644,0,0
126.775000000012,5,1023.3487141679,0,0
126.800000000012,3,1023.67459592313,0,0
126.825000000012,0,1023.8168405957,0,0
126.850000000012,0,1023.8168405957,0,0
126.875000000012,0,1023.8168405957,0,0
126.900000000012,0,1023.8168405957,0,0
126.925000000012,0,1023.8168405957,0,0
126.950000000012,0,1023.8168405957,0,0
126.975000000012,0,1023.8168405957,0,0
127.000000000012,0,1023.8168405957,0,0
127.025000000012,0,1023.8168405957,0,0
127.050000000012,0,1023.8168405957,0,0
127.075000000012,0,1023.8168405957,0,0
127.100000000012,2,1023.93298288451,0,0
127.125000000012,0,1024.04912517332,0,0
127.150000000012,0,1024.04912517332,0,0
127.175000000012,0,1024.04912517332,0,0
127.200000000012,0,1024.04912517332,0,0
127.225000000012,0,1024.04912517332,0,0
127.250000000012,0,1024.04912517332,0,0
127.275000000012,0,1024.04912517332,0,0
127.300000000012,0,1024.04912517332,0,0
127.325000000012,0,1024.04912517332,0,0

127.350000000012,0,1024.04912517332,0,0
127.375000000012,0,1024.04912517332,0,0
127.400000000012,0,1024.04912517332,0,0
127.425000000012,0,1024.04912517332,0,0
127.450000000012,0,1024.04912517332,0,0
127.475000000012,0,1024.04912517332,0,0
127.500000000012,0,1024.04912517332,0,0
127.525000000012,0,1024.04912517332,0,0
127.550000000012,2,1024.16526746213,0,0
127.575000000012,0,1024.28140975094,0,0
127.600000000012,0,1024.28140975094,0,0
127.625000000012,1,1024.36353475094,0,0
127.650000000012,0,1024.44565975094,0,0
127.675000000012,0,1024.44565975094,0,0
127.700000000012,0,1024.44565975094,0,0
127.725000000012,0,1024.44565975094,0,0
127.750000000012,0,1024.44565975094,0,0
127.775000000012,0,1024.44565975094,0,0
127.800000000012,1,1024.52778475094,0,0
127.825000000012,0,1024.60990975094,0,0
127.850000000012,0,1024.60990975094,0,0
127.875000000012,0,1024.60990975094,0,0
127.900000000012,0,1024.60990975094,0,0
127.925000000012,0,1024.60990975094,0,0
127.950000000012,0,1024.60990975094,0,0
127.975000000012,0,1024.60990975094,0,0
128.000000000012,0,1024.60990975094,0,0
128.025000000012,0,1024.60990975094,0,0
128.050000000012,0,1024.60990975094,0,0
128.075000000012,0,1024.60990975094,0,0
128.100000000012,0,1024.60990975094,0,0
128.125000000012,0,1024.60990975094,0,0
128.150000000012,0,1024.60990975094,0,0
128.175000000012,0,1024.60990975094,0,0
128.200000000012,1,1024.69203475094,0,0
128.225000000012,0,1024.77415975094,0,0
128.250000000012,1,1024.85628475094,0,0
128.275000000012,0,1024.93840975094,0,0
128.300000000012,0,1024.93840975094,0,0
128.325000000012,0,1024.93840975094,0,0
128.350000000012,0,1024.93840975094,0,0
128.375000000012,0,1024.93840975094,0,0
128.400000000012,0,1024.93840975094,0,0
128.425000000012,0,1024.93840975094,0,0
128.450000000012,0,1024.93840975094,0,0
128.475000000012,0,1024.93840975094,0,0
128.500000000012,2,1025.05455203975,0,0
128.525000000012,1,1025.25281932856,0,0
128.550000000012,1,1025.41706932856,0,0
128.575000000012,0,1025.49919432856,0,0
128.600000000012,0,1025.49919432856,0,0
128.625000000012,0,1025.49919432856,0,0

128.650000000012,1,1025.58131932856,0,0
128.675000000012,0,1025.66344432856,0,0
128.700000000012,0,1025.66344432856,0,0
128.725000000012,0,1025.66344432856,0,0
128.750000000012,0,1025.66344432856,0,0
128.775000000012,0,1025.66344432856,0,0
128.800000000012,0,1025.66344432856,0,0
128.825000000012,0,1025.66344432856,0,0
128.850000000012,0,1025.66344432856,0,0
128.875000000012,0,1025.66344432856,0,0
128.900000000012,0,1025.66344432856,0,0
128.925000000012,0,1025.66344432856,0,0
128.950000000012,0,1025.66344432856,0,0
128.975000000012,0,1025.66344432856,0,0
129.000000000012,0,1025.66344432856,0,0
129.025000000012,0,1025.66344432856,0,0
129.050000000012,0,1025.66344432856,0,0
129.075000000012,0,1025.66344432856,0,0
129.100000000012,0,1025.66344432856,0,0
129.125000000012,0,1025.66344432856,0,0
129.150000000012,0,1025.66344432856,0,0
129.175000000012,0,1025.66344432856,0,0
129.200000000012,0,1025.66344432856,0,0
129.225000000012,0,1025.66344432856,0,0
129.250000000012,0,1025.66344432856,0,0
129.275000000012,0,1025.66344432856,0,0
129.300000000012,0,1025.66344432856,0,0
129.325000000012,0,1025.66344432856,0,0
129.350000000012,0,1025.66344432856,0,0
129.375000000012,0,1025.66344432856,0,0
129.400000000012,0,1025.66344432856,0,0
129.425000000012,0,1025.66344432856,0,0
129.450000000012,0,1025.66344432856,0,0
129.475000000012,0,1025.66344432856,0,0
129.500000000012,0,1025.66344432856,0,0
129.525000000012,0,1025.66344432856,0,0
129.550000000012,0,1025.66344432856,0,0
129.575000000012,0,1025.66344432856,0,0
129.600000000012,1,1025.74556932856,0,0
129.625000000012,0,1025.82769432856,0,0
129.650000000012,0,1025.82769432856,0,0
129.675000000012,0,1025.82769432856,0,0
129.700000000012,0,1025.82769432856,0,0
129.725000000012,0,1025.82769432856,0,0
129.750000000012,0,1025.82769432856,0,0
129.775000000012,0,1025.82769432856,0,0
129.800000000012,1,1025.90981932856,0,0
129.825000000012,0,1025.99194432856,0,0
129.850000000012,0,1025.99194432856,0,0
129.875000000012,0,1025.99194432856,0,0
129.900000000012,0,1025.99194432856,0,0
129.925000000012,0,1025.99194432856,0,0

```
129.950000000012,0,1025.99194432856,0,0
129.975000000012,0,1025.99194432856,0,0
130.000000000012,0,1025.99194432856,0,0
130.025000000012,0,1025.99194432856,0,0
130.050000000012,0,1025.99194432856,0,0
130.075000000012,0,1025.99194432856,0,0
130.100000000012,0,1025.99194432856,0,0
130.125000000012,0,1025.99194432856,0,0
130.150000000012,0,1025.99194432856,0,0
130.175000000012,0,1025.99194432856,0,0
130.200000000012,0,1025.99194432856,0,0
130.225000000012,0,1025.99194432856,0,0
130.250000000012,0,1025.99194432856,0,0
130.275000000012,0,1025.99194432856,0,0
130.300000000012,1,1026.07406932856,0,0
130.325000000012,0,1026.15619432856,0,0
130.350000000013,0,1026.15619432856,0,0
130.375000000013,0,1026.15619432856,0,0
130.400000000013,0,1026.15619432856,0,0
130.425000000013,0,1026.15619432856,0,0
130.450000000013,0,1026.15619432856,0,0
130.475000000013,0,1026.15619432856,0,0
130.500000000013,1,1026.23831932856,0,0
130.525000000013,0,1026.32044432856,0,0
130.550000000013,0,1026.32044432856,0,0
130.575000000013,0,1026.32044432856,0,0
130.600000000013,0,1026.32044432856,0,0
130.625000000013,0,1026.32044432856,0,0
130.650000000013,0,1026.32044432856,0,0
130.675000000013,0,1026.32044432856,0,0
130.700000000013,0,1026.32044432856,0,0
130.725000000013,0,1026.32044432856,0,0
130.750000000013,0,1026.32044432856,0,0
130.775000000013,0,1026.32044432856,0,0
130.800000000013,0,1026.32044432856,0,0
130.825000000013,0,1026.32044432856,0,0
130.850000000013,0,1026.32044432856,0,0
130.875000000013,1,1026.40256932856,0,0
130.900000000013,1,1026.56681932856,0,0
130.925000000013,0,1026.64894432856,0,0
130.950000000013,1,1026.73106932856,0,0
130.975000000013,0,1026.81319432856,0,0
131.000000000013,0,1026.81319432856,0,0
131.025000000013,0,1026.81319432856,0,0
131.050000000013,0,1026.81319432856,0,0
131.075000000013,0,1026.81319432856,0,0
131.100000000013,0,1026.81319432856,0,0
131.125000000013,0,1026.81319432856,0,0
131.150000000013,0,1026.81319432856,0,0
131.175000000013,0,1026.81319432856,0,0
131.200000000013,0,1026.81319432856,0,0
131.225000000013,0,1026.81319432856,0,0
```

131.250000000013,0,1026.81319432856,0,0
131.275000000013,0,1026.81319432856,0,0
131.300000000013,0,1026.81319432856,0,0
131.325000000013,0,1026.81319432856,0,0
131.350000000013,0,1026.81319432856,0,0
131.375000000013,0,1026.81319432856,0,0
131.400000000013,0,1026.81319432856,0,0
131.425000000013,0,1026.81319432856,0,0
131.450000000013,0,1026.81319432856,0,0
131.475000000013,0,1026.81319432856,0,0
131.500000000013,0,1026.81319432856,0,0
131.525000000013,0,1026.81319432856,0,0
131.550000000013,0,1026.81319432856,0,0
131.575000000013,0,1026.81319432856,0,0
131.600000000013,0,1026.81319432856,0,0
131.625000000013,0,1026.81319432856,0,0
131.650000000013,0,1026.81319432856,0,0
131.675000000013,0,1026.81319432856,0,0
131.700000000013,0,1026.81319432856,0,0
131.725000000013,0,1026.81319432856,0,0
131.750000000013,0,1026.81319432856,0,0
131.775000000013,1,1026.89531932856,0,0
131.800000000013,1,1027.05956932856,0,0
131.825000000013,1,1027.22381932856,0,0
131.850000000013,1,1027.38806932856,0,0
131.875000000013,0,1027.47019432856,0,0
131.900000000013,0,1027.47019432856,0,0
131.925000000013,0,1027.47019432856,0,0
131.950000000013,0,1027.47019432856,0,0
131.975000000013,0,1027.47019432856,0,0
132.000000000013,0,1027.47019432856,0,0
132.025000000013,0,1027.47019432856,0,0
132.050000000013,0,1027.47019432856,0,0
132.075000000013,0,1027.47019432856,0,0
132.100000000013,0,1027.47019432856,0,0
132.125000000013,0,1027.47019432856,0,0
132.150000000013,0,1027.47019432856,0,0
132.175000000013,0,1027.47019432856,0,0
132.200000000013,0,1027.47019432856,0,0
132.225000000013,0,1027.47019432856,0,0
132.250000000013,3,1027.61243900113,0,0
132.275000000013,0,1027.7546836737,0,0
132.300000000013,0,1027.7546836737,0,0
132.325000000013,0,1027.7546836737,0,0
132.350000000013,0,1027.7546836737,0,0
132.375000000013,0,1027.7546836737,0,0
132.400000000013,0,1027.7546836737,0,0
132.425000000013,0,1027.7546836737,0,0
132.450000000013,0,1027.7546836737,0,0
132.475000000013,0,1027.7546836737,0,0
132.500000000013,0,1027.7546836737,0,0
132.525000000013,0,1027.7546836737,0,0

132.550000000013,0,1027.7546836737,0,0
132.575000000013,0,1027.7546836737,0,0
132.600000000013,0,1027.7546836737,0,0
132.625000000013,0,1027.7546836737,0,0
132.650000000013,0,1027.7546836737,0,0
132.675000000013,0,1027.7546836737,0,0
132.700000000013,0,1027.7546836737,0,0
132.725000000013,0,1027.7546836737,0,0
132.750000000013,0,1027.7546836737,0,0
132.775000000013,0,1027.7546836737,0,0
132.800000000013,0,1027.7546836737,0,0
132.825000000013,0,1027.7546836737,0,0
132.850000000013,0,1027.7546836737,0,0
132.875000000013,0,1027.7546836737,0,0
132.900000000013,0,1027.7546836737,0,0
132.925000000013,0,1027.7546836737,0,0
132.950000000013,0,1027.7546836737,0,0
132.975000000013,0,1027.7546836737,0,0
133.000000000013,0,1027.7546836737,0,0
133.025000000013,0,1027.7546836737,0,0
133.050000000013,0,1027.7546836737,0,0
133.075000000013,0,1027.7546836737,0,0
133.100000000013,0,1027.7546836737,0,0
133.125000000013,0,1027.7546836737,0,0
133.150000000013,0,1027.7546836737,0,0
133.175000000013,2,1027.87082596251,0,0
133.200000000013,0,1027.98696825132,0,0
133.225000000013,0,1027.98696825132,0,0
133.250000000013,1,1028.06909325132,0,0
133.275000000013,0,1028.15121825132,0,0
133.300000000013,0,1028.15121825132,0,0
133.325000000013,1,1028.23334325132,0,0
133.350000000013,0,1028.31546825132,0,0
133.375000000013,0,1028.31546825132,0,0
133.400000000013,0,1028.31546825132,0,0
133.425000000013,0,1028.31546825132,0,0
133.450000000013,0,1028.31546825132,0,0
133.475000000013,0,1028.31546825132,0,0
133.500000000013,0,1028.31546825132,0,0
133.525000000013,0,1028.31546825132,0,0
133.550000000013,0,1028.31546825132,0,0
133.575000000013,0,1028.31546825132,0,0
133.600000000013,0,1028.31546825132,0,0
133.625000000013,0,1028.31546825132,0,0
133.650000000013,0,1028.31546825132,0,0
133.675000000013,0,1028.31546825132,0,0
133.700000000013,1,1028.39759325132,0,0
133.725000000013,1,1028.56184325132,0,0
133.750000000013,1,1028.72609325132,0,0
133.775000000013,1,1028.89034325132,0,0
133.800000000013,1,1029.05459325132,0,0
133.825000000013,1,1029.21884325132,0,0

133.850000000013,0,1029.30096825132,0,0
133.875000000013,0,1029.30096825132,0,0
133.900000000013,0,1029.30096825132,0,0
133.925000000013,1,1029.38309325132,0,0
133.950000000013,3,1029.60746292389,0,0
133.975000000013,7,1029.96698992288,0,0
134.000000000013,14,1030.49155586219,30.657132106076,1
134.025000000013,22,1031.18403986936,30.657132106076,1
134.050000000013,24,1031.97156895388,30.657132106076,1
134.075000000013,27,1032.80063166185,30.657132106076,1
134.100000000013,28,1033.66193033241,30.657132106076,1
134.125000000013,27,1034.52322900297,30.657132106076,1
134.150000000013,25,1035.36058802068,30.657132106076,1
134.175000000013,24,1036.17354171093,30.657132106076,1
134.200000000013,25,1036.98649540118,30.657132106076,1
134.225000000013,23,1037.79097806504,30.657132106076,1
134.250000000013,26,1038.60359270644,30.657132106076,1
134.275000000013,25,1039.432974684,30.657132106076,1
134.300000000013,31,1040.30085233229,30.657132106076,1
134.325000000013,32,1041.22267413583,30.657132106076,1
134.350000000013,32,1042.15181244631,30.657132106076,1
134.375000000013,29,1043.05863826134,30.657132106076,1
134.400000000013,33,1043.97266712847,30.657132106076,1
134.425000000013,27,1044.87117335353,30.657132106076,1
134.450000000013,30,1045.7477245216,30.657132106076,1
134.475000000013,32,1046.66211082719,30.657132106076,1
134.500000000013,27,1047.55341400014,30.657132106076,1
134.525000000013,31,1048.43740066615,30.657132106076,1
134.550000000013,26,1049.313410292,30.657132106076,1
134.575000000013,27,1050.15890128727,30.657132106076,1
134.600000000013,26,1051.00439228254,30.657132106076,1
134.625000000013,23,1051.81700692395,30.657132106076,1
134.650000000013,23,1052.60472225165,30.657132106076,1
134.675000000013,22,1053.38378030978,30.657132106076,1
134.700000000013,22,1054.15418109833,30.657132106076,1
134.725000000013,20,1054.90665565791,30.657132106076,1
134.750000000014,20,1055.64120398852,30.657132106076,1
134.775000000014,20,1056.37575231913,30.657132106076,1
134.800000000014,20,1057.11030064974,30.657132106076,1
134.825000000014,18,1057.82600168147,30.657132106076,1
134.850000000014,16,1058.5029285479,30.657132106076,1
134.875000000014,15,1059.14949730521,30.657132106076,1
134.900000000014,15,1059.78563481982,30.657132106076,1
134.925000000014,14,1060.41098719002,30.657132106076,1
134.950000000014,12,1061.00276014805,30.657132106076,1
134.975000000014,12,1061.57173883834,30.657132106076,1
135.000000000014,12,1062.14071752862,30.657132106076,1
135.025000000014,11,1062.69758468467,30.657132106076,1
135.050000000014,9,1063.21633749558,30.657132106076,1
135.075000000014,10,1063.72241454842,30.657132106076,1
135.100000000014,8,1064.21440117888,30.657132106076,1
135.125000000014,7,1064.66396808292,0,0

135.150000000014,7,1065.09853273577,0,0
135.175000000014,8,1065.54809963981,0,0
135.200000000014,8,1066.01266879505,0,0
135.225000000014,7,1066.46223569909,0,0
135.250000000014,6,1066.88068237064,0,0
135.275000000014,5,1067.26548379841,0,0
135.300000000014,5,1067.63275796372,0,0
135.325000000014,5,1068.00003212902,0,0
135.350000000014,5,1068.36730629433,0,0
135.375000000014,5,1068.73458045963,0,0
135.400000000014,5,1069.10185462494,0,0
135.425000000014,4,1069.44974170759,0,0
135.450000000014,5,1069.79762879024,0,0
135.475000000014,4,1070.14551587289,0,0
135.500000000014,4,1070.47401587289,0,0
135.525000000014,2,1070.7544081617,0,0
135.550000000014,3,1071.01279512308,0,0
135.575000000014,2,1071.27118208447,0,0
135.600000000014,2,1071.50346666208,0,0
135.625000000014,1,1071.70173395089,0,0
135.650000000014,1,1071.86598395089,0,0
135.675000000014,1,1072.03023395089,0,0
135.700000000014,1,1072.19448395089,0,0
135.725000000014,0,1072.27660895089,0,0
135.750000000014,1,1072.35873395089,0,0
135.775000000014,1,1072.52298395089,0,0
135.800000000014,0,1072.60510895089,0,0
135.825000000014,1,1072.68723395089,0,0
135.850000000014,0,1072.76935895089,0,0
135.875000000014,0,1072.76935895089,0,0
135.900000000014,0,1072.76935895089,0,0
135.925000000014,0,1072.76935895089,0,0
135.950000000014,0,1072.76935895089,0,0
135.975000000014,0,1072.76935895089,0,0
136.000000000014,0,1072.76935895089,0,0
136.025000000014,0,1072.76935895089,0,0
136.050000000014,0,1072.76935895089,0,0
136.075000000014,0,1072.76935895089,0,0
136.100000000014,0,1072.76935895089,0,0
136.125000000014,0,1072.76935895089,0,0
136.150000000014,0,1072.76935895089,0,0
136.175000000014,0,1072.76935895089,0,0
136.200000000014,0,1072.76935895089,0,0
136.225000000014,0,1072.76935895089,0,0
136.250000000014,0,1072.76935895089,0,0
136.275000000014,0,1072.76935895089,0,0
136.300000000014,0,1072.76935895089,0,0
136.325000000014,0,1072.76935895089,0,0
136.350000000014,1,1072.85148395089,0,0
136.375000000014,0,1072.93360895089,0,0
136.400000000014,1,1073.01573395089,0,0
136.425000000014,0,1073.09785895089,0,0

136.450000000014,0,1073.09785895089,0,0
136.475000000014,1,1073.17998395089,0,0
136.500000000014,0,1073.26210895089,0,0
136.525000000014,1,1073.34423395089,0,0
136.550000000014,1,1073.50848395089,0,0
136.575000000014,1,1073.67273395089,0,0
136.600000000014,1,1073.83698395089,0,0
136.625000000014,1,1074.00123395089,0,0
136.650000000014,1,1074.16548395089,0,0
136.675000000014,1,1074.32973395089,0,0
136.700000000014,0,1074.41185895089,0,0
136.725000000014,1,1074.49398395089,0,0
136.750000000014,1,1074.65823395089,0,0
136.775000000014,1,1074.82248395089,0,0
136.800000000014,1,1074.98673395089,0,0
136.825000000014,1,1075.15098395089,0,0
136.850000000014,1,1075.31523395089,0,0
136.875000000014,1,1075.47948395089,0,0
136.900000000014,1,1075.64373395089,0,0
136.925000000014,1,1075.80798395089,0,0
136.950000000014,0,1075.89010895089,0,0
136.975000000014,0,1075.89010895089,0,0
137.000000000014,0,1075.89010895089,0,0
137.025000000014,0,1075.89010895089,0,0
137.050000000014,0,1075.89010895089,0,0
137.075000000014,0,1075.89010895089,0,0
137.100000000014,0,1075.89010895089,0,0
137.125000000014,0,1075.89010895089,0,0
137.150000000014,0,1075.89010895089,0,0
137.175000000014,0,1075.89010895089,0,0
137.200000000014,0,1075.89010895089,0,0
137.225000000014,0,1075.89010895089,0,0
137.250000000014,0,1075.89010895089,0,0
137.275000000014,0,1075.89010895089,0,0
137.300000000014,0,1075.89010895089,0,0
137.325000000014,0,1075.89010895089,0,0
137.350000000014,1,1075.97223395089,0,0
137.375000000014,1,1076.13648395089,0,0
137.400000000014,0,1076.21860895089,0,0
137.425000000014,0,1076.21860895089,0,0
137.450000000014,0,1076.21860895089,0,0
137.475000000014,0,1076.21860895089,0,0
137.500000000014,0,1076.21860895089,0,0
137.525000000014,1,1076.30073395089,0,0
137.550000000014,0,1076.38285895089,0,0
137.575000000014,0,1076.38285895089,0,0
137.600000000014,0,1076.38285895089,0,0
137.625000000014,0,1076.38285895089,0,0
137.650000000014,0,1076.38285895089,0,0
137.675000000014,0,1076.38285895089,0,0
137.700000000014,0,1076.38285895089,0,0
137.725000000014,0,1076.38285895089,0,0

137.750000000014,0,1076.38285895089,0,0
137.775000000014,1,1076.46498395089,0,0
137.800000000014,0,1076.54710895089,0,0
137.825000000014,0,1076.54710895089,0,0
137.850000000014,0,1076.54710895089,0,0
137.875000000014,0,1076.54710895089,0,0
137.900000000014,0,1076.54710895089,0,0
137.925000000014,0,1076.54710895089,0,0
137.950000000014,0,1076.54710895089,0,0
137.975000000014,0,1076.54710895089,0,0
138.000000000014,1,1076.62923395089,0,0
138.025000000014,2,1076.8275012397,0,0
138.050000000014,0,1076.94364352851,0,0
138.075000000014,0,1076.94364352851,0,0
138.100000000014,1,1077.02576852851,0,0
138.125000000014,0,1077.10789352851,0,0
138.150000000014,0,1077.10789352851,0,0
138.175000000014,0,1077.10789352851,0,0
138.200000000014,0,1077.10789352851,0,0
138.225000000014,2,1077.22403581732,0,0
138.250000000014,2,1077.45632039494,0,0
138.275000000014,0,1077.57246268375,0,0
138.300000000014,0,1077.57246268375,0,0
138.325000000014,0,1077.57246268375,0,0
138.350000000014,0,1077.57246268375,0,0
138.375000000014,0,1077.57246268375,0,0
138.400000000014,0,1077.57246268375,0,0
138.425000000014,0,1077.57246268375,0,0
138.450000000014,0,1077.57246268375,0,0
138.475000000014,0,1077.57246268375,0,0
138.500000000014,0,1077.57246268375,0,0
138.525000000014,0,1077.57246268375,0,0
138.550000000014,0,1077.57246268375,0,0
138.575000000014,0,1077.57246268375,0,0
138.600000000014,0,1077.57246268375,0,0
138.625000000014,0,1077.57246268375,0,0
138.650000000014,0,1077.57246268375,0,0
138.675000000014,0,1077.57246268375,0,0
138.700000000014,0,1077.57246268375,0,0
138.725000000014,0,1077.57246268375,0,0
138.750000000014,0,1077.57246268375,0,0
138.775000000014,0,1077.57246268375,0,0
138.800000000014,0,1077.57246268375,0,0
138.825000000014,0,1077.57246268375,0,0
138.850000000014,0,1077.57246268375,0,0
138.875000000014,0,1077.57246268375,0,0
138.900000000014,0,1077.57246268375,0,0
138.925000000014,0,1077.57246268375,0,0
138.950000000014,0,1077.57246268375,0,0
138.975000000014,0,1077.57246268375,0,0
139.000000000014,0,1077.57246268375,0,0
139.025000000014,1,1077.65458768375,0,0

139.050000000014,1,1077.81883768375,0,0
139.075000000014,1,1077.98308768375,0,0
139.100000000014,1,1078.14733768375,0,0
139.125000000015,1,1078.31158768375,0,0
139.150000000015,1,1078.47583768375,0,0
139.175000000015,0,1078.55796268375,0,0
139.200000000015,0,1078.55796268375,0,0
139.225000000015,1,1078.64008768375,0,0
139.250000000015,0,1078.72221268375,0,0
139.275000000015,1,1078.80433768375,0,0
139.300000000015,0,1078.88646268375,0,0
139.325000000015,0,1078.88646268375,0,0
139.350000000015,0,1078.88646268375,0,0
139.375000000015,0,1078.88646268375,0,0
139.400000000015,0,1078.88646268375,0,0
139.425000000015,0,1078.88646268375,0,0
139.450000000015,0,1078.88646268375,0,0
139.475000000015,0,1078.88646268375,0,0
139.500000000015,0,1078.88646268375,0,0
139.525000000015,0,1078.88646268375,0,0
139.550000000015,0,1078.88646268375,0,0
139.575000000015,0,1078.88646268375,0,0
139.600000000015,0,1078.88646268375,0,0
139.625000000015,0,1078.88646268375,0,0
139.650000000015,0,1078.88646268375,0,0
139.675000000015,0,1078.88646268375,0,0
139.700000000015,0,1078.88646268375,0,0
139.725000000015,0,1078.88646268375,0,0
139.750000000015,0,1078.88646268375,0,0
139.775000000015,0,1078.88646268375,0,0
139.800000000015,0,1078.88646268375,0,0
139.825000000015,1,1078.96858768375,0,0
139.850000000015,0,1079.05071268375,0,0
139.875000000015,1,1079.13283768375,0,0
139.900000000015,0,1079.21496268375,0,0
139.925000000015,0,1079.21496268375,0,0
139.950000000015,1,1079.29708768375,0,0
139.975000000015,0,1079.37921268375,0,0
140.000000000015,0,1079.37921268375,0,0
140.025000000015,1,1079.46133768375,0,0
140.050000000015,0,1079.54346268375,0,0
140.075000000015,0,1079.54346268375,0,0
140.100000000015,0,1079.54346268375,0,0
140.125000000015,1,1079.62558768375,0,0
140.150000000015,1,1079.78983768375,0,0
140.175000000015,1,1079.95408768375,0,0
140.200000000015,0,1080.03621268375,0,0
140.225000000015,1,1080.11833768375,0,0
140.250000000015,0,1080.20046268375,0,0
140.275000000015,0,1080.20046268375,0,0
140.300000000015,0,1080.20046268375,0,0
140.325000000015,0,1080.20046268375,0,0

140.350000000015,0,1080.20046268375,0,0
140.375000000015,0,1080.20046268375,0,0
140.400000000015,0,1080.20046268375,0,0
140.425000000015,0,1080.20046268375,0,0
140.450000000015,0,1080.20046268375,0,0
140.475000000015,0,1080.20046268375,0,0
140.500000000015,0,1080.20046268375,0,0
140.525000000015,0,1080.20046268375,0,0
140.550000000015,0,1080.20046268375,0,0
140.575000000015,0,1080.20046268375,0,0
140.600000000015,0,1080.20046268375,0,0
140.625000000015,0,1080.20046268375,0,0
140.650000000015,0,1080.20046268375,0,0
140.675000000015,1,1080.28258768375,0,0
140.700000000015,1,1080.44683768375,0,0
140.725000000015,1,1080.61108768375,0,0
140.750000000015,0,1080.69321268375,0,0
140.775000000015,0,1080.69321268375,0,0
140.800000000015,0,1080.69321268375,0,0
140.825000000015,0,1080.69321268375,0,0
140.850000000015,0,1080.69321268375,0,0
140.875000000015,0,1080.69321268375,0,0
140.900000000015,0,1080.69321268375,0,0
140.925000000015,0,1080.69321268375,0,0
140.950000000015,0,1080.69321268375,0,0
140.975000000015,0,1080.69321268375,0,0
141.000000000015,0,1080.69321268375,0,0
141.025000000015,0,1080.69321268375,0,0
141.050000000015,0,1080.69321268375,0,0
141.075000000015,1,1080.77533768375,0,0
141.100000000015,0,1080.85746268375,0,0
141.125000000015,0,1080.85746268375,0,0
141.150000000015,0,1080.85746268375,0,0
141.175000000015,0,1080.85746268375,0,0
141.200000000015,0,1080.85746268375,0,0
141.225000000015,1,1080.93958768375,0,0
141.250000000015,0,1081.02171268375,0,0
141.275000000015,0,1081.02171268375,0,0
141.300000000015,0,1081.02171268375,0,0
141.325000000015,0,1081.02171268375,0,0
141.350000000015,0,1081.02171268375,0,0
141.375000000015,0,1081.02171268375,0,0
141.400000000015,0,1081.02171268375,0,0
141.425000000015,0,1081.02171268375,0,0
141.450000000015,0,1081.02171268375,0,0
141.475000000015,0,1081.02171268375,0,0
141.500000000015,0,1081.02171268375,0,0
141.525000000015,0,1081.02171268375,0,0
141.550000000015,0,1081.02171268375,0,0
141.575000000015,0,1081.02171268375,0,0
141.600000000015,0,1081.02171268375,0,0
141.625000000015,1,1081.10383768375,0,0

141.650000000015,0,1081.18596268375,0,0
141.675000000015,7,1081.40324501017,0,0
141.700000000015,0,1081.62052733659,0,0
141.725000000015,0,1081.62052733659,0,0
141.750000000015,3,1081.76277200916,0,0
141.775000000015,0,1081.90501668174,0,0
141.800000000015,0,1081.90501668174,0,0
141.825000000015,2,1082.02115897055,0,0
141.850000000015,5,1082.32093834201,0,0
141.875000000015,0,1082.50457542466,0,0
141.900000000015,1,1082.58670042466,0,0
141.925000000015,0,1082.66882542466,0,0
141.950000000015,2,1082.78496771347,0,0
141.975000000015,0,1082.90111000228,0,0
142.000000000015,0,1082.90111000228,0,0
142.025000000015,0,1082.90111000228,0,0
142.050000000015,0,1082.90111000228,0,0
142.075000000015,0,1082.90111000228,0,0
142.100000000015,1,1082.98323500228,0,0
142.125000000015,0,1083.06536000228,0,0
142.150000000015,0,1083.06536000228,0,0
142.175000000015,0,1083.06536000228,0,0
142.200000000015,0,1083.06536000228,0,0
142.225000000015,0,1083.06536000228,0,0
142.250000000015,0,1083.06536000228,0,0
142.275000000015,0,1083.06536000228,0,0
142.300000000015,0,1083.06536000228,0,0
142.325000000015,0,1083.06536000228,0,0
142.350000000015,0,1083.06536000228,0,0
142.375000000015,0,1083.06536000228,0,0
142.400000000015,0,1083.06536000228,0,0
142.425000000015,0,1083.06536000228,0,0
142.450000000015,0,1083.06536000228,0,0
142.475000000015,1,1083.14748500228,0,0
142.500000000015,0,1083.22961000228,0,0
142.525000000015,0,1083.22961000228,0,0
142.550000000015,3,1083.37185467485,0,0
142.575000000015,0,1083.51409934742,0,0
142.600000000015,0,1083.51409934742,0,0
142.625000000015,0,1083.51409934742,0,0
142.650000000015,0,1083.51409934742,0,0
142.675000000015,0,1083.51409934742,0,0
142.700000000015,0,1083.51409934742,0,0
142.725000000015,0,1083.51409934742,0,0
142.750000000015,0,1083.51409934742,0,0
142.775000000015,0,1083.51409934742,0,0
142.800000000015,0,1083.51409934742,0,0
142.825000000015,1,1083.59622434742,0,0
142.850000000015,0,1083.67834934742,0,0
142.875000000015,0,1083.67834934742,0,0
142.900000000015,0,1083.67834934742,0,0
142.925000000015,0,1083.67834934742,0,0

142.950000000015,0,1083.67834934742,0,0
142.975000000015,0,1083.67834934742,0,0
143.000000000015,0,1083.67834934742,0,0
143.025000000015,1,1083.76047434742,0,0
143.050000000015,0,1083.84259934742,0,0
143.075000000015,0,1083.84259934742,0,0
143.100000000015,0,1083.84259934742,0,0
143.125000000015,0,1083.84259934742,0,0
143.150000000015,0,1083.84259934742,0,0
143.175000000015,0,1083.84259934742,0,0
143.200000000015,0,1083.84259934742,0,0
143.225000000015,0,1083.84259934742,0,0
143.250000000015,0,1083.84259934742,0,0
143.275000000015,1,1083.92472434742,0,0
143.300000000015,0,1084.00684934742,0,0
143.325000000015,1,1084.08897434742,0,0
143.350000000015,0,1084.17109934742,0,0
143.375000000015,0,1084.17109934742,0,0
143.400000000015,0,1084.17109934742,0,0
143.425000000015,0,1084.17109934742,0,0
143.450000000015,0,1084.17109934742,0,0
143.475000000015,0,1084.17109934742,0,0
143.500000000015,0,1084.17109934742,0,0
143.525000000016,0,1084.17109934742,0,0
143.550000000016,0,1084.17109934742,0,0
143.575000000016,0,1084.17109934742,0,0
143.600000000016,1,1084.25322434742,0,0
143.625000000016,0,1084.33534934742,0,0
143.650000000016,0,1084.33534934742,0,0
143.675000000016,0,1084.33534934742,0,0
143.700000000016,0,1084.33534934742,0,0
143.725000000016,0,1084.33534934742,0,0
143.750000000016,0,1084.33534934742,0,0
143.775000000016,0,1084.33534934742,0,0
143.800000000016,0,1084.33534934742,0,0
143.825000000016,0,1084.33534934742,0,0
143.850000000016,0,1084.33534934742,0,0
143.875000000016,0,1084.33534934742,0,0
143.900000000016,0,1084.33534934742,0,0
143.925000000016,0,1084.33534934742,0,0
143.950000000016,0,1084.33534934742,0,0
143.975000000016,0,1084.33534934742,0,0
144.000000000016,0,1084.33534934742,0,0
144.025000000016,0,1084.33534934742,0,0
144.050000000016,0,1084.33534934742,0,0
144.075000000016,0,1084.33534934742,0,0
144.100000000016,0,1084.33534934742,0,0
144.125000000016,0,1084.33534934742,0,0
144.150000000016,0,1084.33534934742,0,0
144.175000000016,0,1084.33534934742,0,0
144.200000000016,1,1084.41747434742,0,0
144.225000000016,0,1084.49959934742,0,0

144.250000000016,0,1084.49959934742,0,0
144.275000000016,0,1084.49959934742,0,0
144.300000000016,0,1084.49959934742,0,0
144.325000000016,0,1084.49959934742,0,0
144.350000000016,0,1084.49959934742,0,0
144.375000000016,1,1084.58172434742,0,0
144.400000000016,0,1084.66384934742,0,0
144.425000000016,0,1084.66384934742,0,0
144.450000000016,0,1084.66384934742,0,0
144.475000000016,1,1084.74597434742,0,0
144.500000000016,0,1084.82809934742,0,0
144.525000000016,0,1084.82809934742,0,0
144.550000000016,0,1084.82809934742,0,0
144.575000000016,0,1084.82809934742,0,0
144.600000000016,0,1084.82809934742,0,0
144.625000000016,1,1084.91022434742,0,0
144.650000000016,0,1084.99234934742,0,0
144.675000000016,0,1084.99234934742,0,0
144.700000000016,0,1084.99234934742,0,0
144.725000000016,0,1084.99234934742,0,0
144.750000000016,0,1084.99234934742,0,0
144.775000000016,0,1084.99234934742,0,0
144.800000000016,0,1084.99234934742,0,0
144.825000000016,0,1084.99234934742,0,0
144.850000000016,0,1084.99234934742,0,0
144.875000000016,0,1084.99234934742,0,0
144.900000000016,0,1084.99234934742,0,0
144.925000000016,0,1084.99234934742,0,0
144.950000000016,0,1084.99234934742,0,0
144.975000000016,0,1084.99234934742,0,0
145.000000000016,0,1084.99234934742,0,0
145.025000000016,0,1084.99234934742,0,0
145.050000000016,0,1084.99234934742,0,0
145.075000000016,0,1084.99234934742,0,0
145.100000000016,0,1084.99234934742,0,0
145.125000000016,0,1084.99234934742,0,0
145.150000000016,0,1084.99234934742,0,0
145.175000000016,0,1084.99234934742,0,0
145.200000000016,0,1084.99234934742,0,0
145.225000000016,0,1084.99234934742,0,0
145.250000000016,0,1084.99234934742,0,0
145.275000000016,0,1084.99234934742,0,0
145.300000000016,0,1084.99234934742,0,0
145.325000000016,0,1084.99234934742,0,0
145.350000000016,0,1084.99234934742,0,0
145.375000000016,1,1085.07447434742,0,0
145.400000000016,0,1085.15659934742,0,0
145.425000000016,0,1085.15659934742,0,0
145.450000000016,0,1085.15659934742,0,0
145.475000000016,3,1085.29884401999,0,0
145.500000000016,0,1085.44108869256,0,0
145.525000000016,0,1085.44108869256,0,0

145.550000000016,7,1085.65837101899,0,0
145.575000000016,6,1086.07681769053,0,0
145.600000000016,2,1086.39412432447,0,0
145.625000000016,0,1086.51026661328,0,0
145.650000000016,0,1086.51026661328,0,0
145.675000000016,4,1086.67451661328,0,0
145.700000000016,0,1086.83876661328,0,0
145.725000000016,2,1086.95490890209,0,0
145.750000000016,1,1087.1531761909,0,0
145.775000000016,0,1087.2353011909,0,0
145.800000000016,0,1087.2353011909,0,0
145.825000000016,0,1087.2353011909,0,0
145.850000000016,0,1087.2353011909,0,0
145.875000000016,0,1087.2353011909,0,0
145.900000000016,0,1087.2353011909,0,0
145.925000000016,0,1087.2353011909,0,0
145.950000000016,0,1087.2353011909,0,0
145.975000000016,0,1087.2353011909,0,0
146.000000000016,0,1087.2353011909,0,0
146.025000000016,1,1087.3174261909,0,0
146.050000000016,0,1087.3995511909,0,0
146.075000000016,0,1087.3995511909,0,0
146.100000000016,0,1087.3995511909,0,0
146.125000000016,0,1087.3995511909,0,0
146.150000000016,0,1087.3995511909,0,0
146.175000000016,0,1087.3995511909,0,0
146.200000000016,0,1087.3995511909,0,0
146.225000000016,0,1087.3995511909,0,0
146.250000000016,0,1087.3995511909,0,0
146.275000000016,0,1087.3995511909,0,0
146.300000000016,1,1087.4816761909,0,0
146.325000000016,0,1087.5638011909,0,0
146.350000000016,0,1087.5638011909,0,0
146.375000000016,0,1087.5638011909,0,0
146.400000000016,0,1087.5638011909,0,0
146.425000000016,0,1087.5638011909,0,0
146.450000000016,0,1087.5638011909,0,0
146.475000000016,0,1087.5638011909,0,0
146.500000000016,0,1087.5638011909,0,0
146.525000000016,0,1087.5638011909,0,0
146.550000000016,0,1087.5638011909,0,0
146.575000000016,0,1087.5638011909,0,0
146.600000000016,0,1087.5638011909,0,0
146.625000000016,0,1087.5638011909,0,0
146.650000000016,0,1087.5638011909,0,0
146.675000000016,0,1087.5638011909,0,0
146.700000000016,0,1087.5638011909,0,0
146.725000000016,0,1087.5638011909,0,0
146.750000000016,0,1087.5638011909,0,0
146.775000000016,0,1087.5638011909,0,0
146.800000000016,0,1087.5638011909,0,0
146.825000000016,0,1087.5638011909,0,0

146.850000000016,0,1087.5638011909,0,0
146.875000000016,0,1087.5638011909,0,0
146.900000000016,1,1087.6459261909,0,0
146.925000000016,0,1087.7280511909,0,0
146.950000000016,0,1087.7280511909,0,0
146.975000000016,0,1087.7280511909,0,0
147.000000000016,0,1087.7280511909,0,0
147.025000000016,0,1087.7280511909,0,0
147.050000000016,0,1087.7280511909,0,0
147.075000000016,0,1087.7280511909,0,0
147.100000000016,0,1087.7280511909,0,0
147.125000000016,0,1087.7280511909,0,0
147.150000000016,0,1087.7280511909,0,0
147.175000000016,0,1087.7280511909,0,0
147.200000000016,0,1087.7280511909,0,0
147.225000000016,0,1087.7280511909,0,0
147.250000000016,0,1087.7280511909,0,0
147.275000000016,0,1087.7280511909,0,0
147.300000000016,0,1087.7280511909,0,0
147.325000000016,0,1087.7280511909,0,0
147.350000000016,0,1087.7280511909,0,0
147.375000000016,0,1087.7280511909,0,0
147.400000000016,0,1087.7280511909,0,0
147.425000000016,0,1087.7280511909,0,0
147.450000000016,0,1087.7280511909,0,0
147.475000000016,0,1087.7280511909,0,0
147.500000000016,0,1087.7280511909,0,0
147.525000000016,0,1087.7280511909,0,0
147.550000000016,0,1087.7280511909,0,0
147.575000000016,0,1087.7280511909,0,0
147.600000000016,1,1087.8101761909,0,0
147.625000000016,0,1087.8923011909,0,0
147.650000000016,0,1087.8923011909,0,0
147.675000000016,0,1087.8923011909,0,0
147.700000000016,0,1087.8923011909,0,0
147.725000000016,0,1087.8923011909,0,0
147.750000000016,1,1087.9744261909,0,0
147.775000000016,0,1088.0565511909,0,0
147.800000000016,0,1088.0565511909,0,0
147.825000000016,1,1088.1386761909,0,0
147.850000000016,0,1088.2208011909,0,0
147.875000000016,1,1088.3029261909,0,0
147.900000000016,0,1088.3850511909,0,0
147.925000000017,0,1088.3850511909,0,0
147.950000000017,0,1088.3850511909,0,0
147.975000000017,0,1088.3850511909,0,0
148.000000000017,0,1088.3850511909,0,0
148.025000000017,5,1088.56868827355,0,0
148.050000000017,16,1089.0808253562,29.7252573426603,1
148.075000000017,26,1089.82808233375,29.7252573426603,1
148.100000000017,27,1090.67357332902,29.7252573426603,1
148.125000000017,24,1091.50263603699,29.7252573426603,1

148.150000000017,24,1092.30729341749,29.7252573426603,1
148.175000000017,21,1093.08596613669,29.7252573426603,1
148.200000000017,18,1093.81073703207,29.7252573426603,1
148.225000000017,15,1094.47723265581,29.7252573426603,1
148.250000000017,17,1095.13391146262,29.7252573426603,1
148.275000000017,17,1095.81113156163,29.7252573426603,1
148.300000000017,19,1096.50771618687,29.7252573426603,1
148.325000000017,22,1097.25089115688,29.7252573426603,1
148.350000000017,25,1098.04671655116,29.7252573426603,1
148.375000000017,25,1098.86796655116,29.7252573426603,1
148.400000000017,26,1099.69734852871,29.7252573426603,1
148.425000000017,26,1100.53486248382,29.7252573426603,1
148.450000000017,27,1101.38035347909,29.7252573426603,1
148.475000000017,26,1102.22584447436,29.7252573426603,1
148.500000000017,26,1103.06335842947,29.7252573426603,1
148.525000000017,28,1103.91668005986,29.7252573426603,1
148.550000000017,28,1104.78580936555,29.7252573426603,1
148.575000000017,30,1105.67019116874,29.7252573426603,1
148.600000000017,30,1106.56982546944,29.7252573426603,1
148.625000000017,30,1107.46945977015,29.7252573426603,1
148.650000000017,34,1108.39814384486,29.7252573426603,1
148.675000000017,32,1109.34157992446,29.7252573426603,1
148.700000000017,32,1110.27071823494,29.7252573426603,1
148.725000000017,35,1111.22114544237,29.7252573426603,1
148.750000000017,34,1112.18587041892,29.7252573426603,1
148.775000000017,36,1113.15748734329,29.7252573426603,1
148.800000000017,34,1114.12910426765,29.7252573426603,1
148.825000000017,32,1115.07254034725,29.7252573426603,1
148.850000000017,30,1115.98692665284,29.7252573426603,1
148.875000000017,31,1116.89399645149,29.7252573426603,1
148.900000000017,31,1117.80850174809,29.7252573426603,1
148.925000000017,30,1118.71557154674,29.7252573426603,1
148.950000000017,31,1119.62264134538,29.7252573426603,1
148.975000000017,26,1120.49865097124,29.7252573426603,1
149.000000000017,24,1121.31973663904,29.7252573426603,1
149.025000000017,22,1122.10726572357,29.7252573426603,1
149.050000000017,18,1122.84089298427,29.7252573426603,1
149.075000000017,13,1123.4854257492,29.7252573426603,1
149.100000000017,15,1124.09960040501,29.7252573426603,1
149.125000000017,17,1124.75627921182,29.7252573426603,1
149.150000000017,18,1125.44331612775,29.7252573426603,1
149.175000000017,16,1126.12024299418,29.7252573426603,1
149.200000000017,23,1126.84260065803,29.7252573426603,1
149.225000000017,19,1127.59443289762,29.7252573426603,1
149.250000000017,23,1128.34626513721,29.7252573426603,1
149.275000000017,28,1129.17468745391,29.7252573426603,1
149.300000000017,25,1130.01987710675,29.7252573426603,1
149.325000000017,22,1130.81570250103,29.7252573426603,1
149.350000000017,23,1131.59476055915,29.7252573426603,1
149.375000000017,20,1132.35589238831,29.7252573426603,1
149.400000000017,21,1133.09951058256,29.7252573426603,1
149.425000000017,18,1133.82428147794,29.7252573426603,1

149.450000000017,20,1134.53998250967,29.7252573426603,1
149.475000000017,17,1135.24586672448,29.7252573426603,1
149.500000000017,15,1135.90254553129,29.7252573426603,1
149.525000000017,15,1136.53868304591,29.7252573426603,1
149.550000000017,14,1137.1640354161,29.7252573426603,1
149.575000000017,14,1137.77860264188,29.7252573426603,1
149.600000000017,14,1138.39316986766,29.7252573426603,1
149.625000000017,13,1138.99655937905,29.7252573426603,1
149.650000000017,12,1139.57715462269,29.7252573426603,1
149.675000000017,10,1140.12134602067,29.7252573426603,1
149.700000000017,9,1140.62742307351,29.7252573426603,1
149.725000000017,9,1141.12017307351,29.7252573426603,1
149.750000000017,9,1141.61292307351,29.7252573426603,1
149.775000000017,10,1142.11900012635,29.7252573426603,1
149.800000000017,10,1142.63840423204,29.7252573426603,1
149.825000000017,12,1143.18259563002,29.7252573426603,1
149.850000000017,14,1143.77436858805,29.7252573426603,1
149.875000000017,16,1144.41015220094,29.7252573426603,1
149.900000000017,17,1145.07726225045,29.7252573426603,1
149.925000000017,19,1145.77384687569,29.7252573426603,1
149.950000000017,19,1146.48979602716,29.7252573426603,1
149.975000000017,21,1147.22411463185,29.7252573426603,1
150.000000000017,21,1147.97680268975,29.7252573426603,1
150.025000000017,20,1148.720420884,29.7252573426603,1
150.050000000017,18,1149.43612191573,29.7252573426603,1
150.075000000017,19,1150.1425233579,29.7252573426603,1
150.100000000017,19,1150.85847250938,29.7252573426603,1
150.125000000017,20,1151.58372125042,29.7252573426603,1
150.150000000017,20,1152.31826958103,29.7252573426603,1
150.175000000017,19,1153.04351832207,29.7252573426603,1
150.200000000017,19,1153.75946747355,29.7252573426603,1
150.225000000017,19,1154.47541662502,29.7252573426603,1
150.250000000017,18,1155.18181806719,29.7252573426603,1
150.275000000017,20,1155.89751909893,29.7252573426603,1
150.300000000017,19,1156.62276783997,29.7252573426603,1
150.325000000017,19,1157.33871699145,29.7252573426603,1
150.350000000017,20,1158.06396573249,29.7252573426603,1
150.375000000017,19,1158.78921447353,29.7252573426603,1
150.400000000017,21,1159.52353307822,29.7252573426603,1
150.425000000017,20,1160.26715127247,29.7252573426603,1
150.450000000017,21,1161.01076946672,29.7252573426603,1
150.475000000017,19,1161.74508807141,29.7252573426603,1
150.500000000017,19,1162.46103722289,29.7252573426603,1
150.525000000017,18,1163.16743866505,29.7252573426603,1
150.550000000017,19,1163.87384010722,29.7252573426603,1
150.575000000017,20,1164.59908884826,29.7252573426603,1
150.600000000017,16,1165.29486301357,29.7252573426603,1
150.625000000017,0,1165.62336301357,29.7252573426603,1
150.650000000017,0,1165.62336301357,0,0
150.675000000017,0,1165.62336301357,0,0
150.700000000017,0,1165.62336301357,0,0
150.725000000017,0,1165.62336301357,0,0

150.750000000017,0,1165.62336301357,0,0
150.775000000017,0,1165.62336301357,0,0
150.800000000017,0,1165.62336301357,0,0
150.825000000017,0,1165.62336301357,0,0
150.850000000017,1,1165.70548801357,0,0
150.875000000017,0,1165.78761301357,0,0
150.900000000017,0,1165.78761301357,0,0
150.925000000017,0,1165.78761301357,0,0
150.950000000017,0,1165.78761301357,0,0
150.975000000017,0,1165.78761301357,0,0
151.000000000017,0,1165.78761301357,0,0
151.025000000017,0,1165.78761301357,0,0
151.050000000017,0,1165.78761301357,0,0
151.075000000017,0,1165.78761301357,0,0
151.100000000017,0,1165.78761301357,0,0
151.125000000017,0,1165.78761301357,0,0
151.150000000017,0,1165.78761301357,0,0
151.175000000017,0,1165.78761301357,0,0
151.200000000017,0,1165.78761301357,0,0
151.225000000017,0,1165.78761301357,0,0
151.250000000017,0,1165.78761301357,0,0
151.275000000017,0,1165.78761301357,0,0
151.300000000017,0,1165.78761301357,0,0
151.325000000017,0,1165.78761301357,0,0
151.350000000017,0,1165.78761301357,0,0
151.375000000017,0,1165.78761301357,0,0
151.400000000017,0,1165.78761301357,0,0
151.425000000017,0,1165.78761301357,0,0
151.450000000017,0,1165.78761301357,0,0
151.475000000017,0,1165.78761301357,0,0
151.500000000017,1,1165.86973801357,0,0
151.525000000017,0,1165.95186301357,0,0
151.550000000017,0,1165.95186301357,0,0
151.575000000017,0,1165.95186301357,0,0
151.600000000017,0,1165.95186301357,0,0
151.625000000017,0,1165.95186301357,0,0
151.650000000017,1,1166.03398801357,0,0
151.675000000017,0,1166.11611301357,0,0
151.700000000017,0,1166.11611301357,0,0
151.725000000017,0,1166.11611301357,0,0
151.750000000017,0,1166.11611301357,0,0
151.775000000017,0,1166.11611301357,0,0
151.800000000017,0,1166.11611301357,0,0
151.825000000017,0,1166.11611301357,0,0
151.850000000017,0,1166.11611301357,0,0
151.875000000017,0,1166.11611301357,0,0
151.900000000017,0,1166.11611301357,0,0
151.925000000017,0,1166.11611301357,0,0
151.950000000017,0,1166.11611301357,0,0
151.975000000017,0,1166.11611301357,0,0
152.000000000017,0,1166.11611301357,0,0
152.025000000017,0,1166.11611301357,0,0

152.050000000017,0,1166.11611301357,0,0
152.075000000017,0,1166.11611301357,0,0
152.100000000017,0,1166.11611301357,0,0
152.125000000017,0,1166.11611301357,0,0
152.150000000017,0,1166.11611301357,0,0
152.175000000017,0,1166.11611301357,0,0
152.200000000017,0,1166.11611301357,0,0
152.225000000017,0,1166.11611301357,0,0
152.250000000017,0,1166.11611301357,0,0
152.275000000017,0,1166.11611301357,0,0
152.300000000017,0,1166.11611301357,0,0
152.325000000018,0,1166.11611301357,0,0
152.350000000018,0,1166.11611301357,0,0
152.375000000018,0,1166.11611301357,0,0
152.400000000018,2,1166.23225530238,0,0
152.425000000018,0,1166.34839759119,0,0
152.450000000018,0,1166.34839759119,0,0
152.475000000018,1,1166.43052259119,0,0
152.500000000018,0,1166.51264759119,0,0
152.525000000018,0,1166.51264759119,0,0
152.550000000018,0,1166.51264759119,0,0
152.575000000018,0,1166.51264759119,0,0
152.600000000018,0,1166.51264759119,0,0
152.625000000018,0,1166.51264759119,0,0
152.650000000018,0,1166.51264759119,0,0
152.675000000018,0,1166.51264759119,0,0
152.700000000018,1,1166.59477259119,0,0
152.725000000018,0,1166.67689759119,0,0
152.750000000018,0,1166.67689759119,0,0
152.775000000018,1,1166.75902259119,0,0
152.800000000018,0,1166.84114759119,0,0
152.825000000018,0,1166.84114759119,0,0
152.850000000018,0,1166.84114759119,0,0
152.875000000018,5,1167.02478467384,0,0
152.900000000018,1,1167.29054675649,0,0
152.925000000018,0,1167.37267175649,0,0
152.950000000018,0,1167.37267175649,0,0
152.975000000018,0,1167.37267175649,0,0
153.000000000018,0,1167.37267175649,0,0
153.025000000018,1,1167.45479675649,0,0
153.050000000018,0,1167.53692175649,0,0
153.075000000018,7,1167.75420408291,0,0
153.100000000018,0,1167.97148640933,0,0
153.125000000018,0,1167.97148640933,0,0
153.150000000018,0,1167.97148640933,0,0
153.175000000018,0,1167.97148640933,0,0
153.200000000018,0,1167.97148640933,0,0
153.225000000018,0,1167.97148640933,0,0
153.250000000018,0,1167.97148640933,0,0
153.275000000018,1,1168.05361140933,0,0
153.300000000018,0,1168.13573640933,0,0
153.325000000018,0,1168.13573640933,0,0

153.350000000018,0,1168.13573640933,0,0
153.375000000018,0,1168.13573640933,0,0
153.400000000018,0,1168.13573640933,0,0
153.425000000018,0,1168.13573640933,0,0
153.450000000018,0,1168.13573640933,0,0
153.475000000018,0,1168.13573640933,0,0
153.500000000018,0,1168.13573640933,0,0
153.525000000018,0,1168.13573640933,0,0
153.550000000018,0,1168.13573640933,0,0
153.575000000018,0,1168.13573640933,0,0
153.600000000018,0,1168.13573640933,0,0
153.625000000018,0,1168.13573640933,0,0
153.650000000018,0,1168.13573640933,0,0
153.675000000018,0,1168.13573640933,0,0
153.700000000018,1,1168.21786140933,0,0
153.725000000018,0,1168.29998640933,0,0
153.750000000018,0,1168.29998640933,0,0
153.775000000018,1,1168.38211140933,0,0
153.800000000018,1,1168.54636140933,0,0
153.825000000018,0,1168.62848640933,0,0
153.850000000018,0,1168.62848640933,0,0
153.875000000018,0,1168.62848640933,0,0
153.900000000018,0,1168.62848640933,0,0
153.925000000018,0,1168.62848640933,0,0
153.950000000018,0,1168.62848640933,0,0
153.975000000018,0,1168.62848640933,0,0
154.000000000018,0,1168.62848640933,0,0
154.025000000018,0,1168.62848640933,0,0
154.050000000018,0,1168.62848640933,0,0
154.075000000018,0,1168.62848640933,0,0
154.100000000018,0,1168.62848640933,0,0
154.125000000018,0,1168.62848640933,0,0
154.150000000018,0,1168.62848640933,0,0
154.175000000018,0,1168.62848640933,0,0
154.200000000018,0,1168.62848640933,0,0
154.225000000018,0,1168.62848640933,0,0
154.250000000018,0,1168.62848640933,0,0
154.275000000018,0,1168.62848640933,0,0
154.300000000018,0,1168.62848640933,0,0
154.325000000018,0,1168.62848640933,0,0
154.350000000018,0,1168.62848640933,0,0
154.375000000018,0,1168.62848640933,0,0
154.400000000018,0,1168.62848640933,0,0
154.425000000018,0,1168.62848640933,0,0
154.450000000018,0,1168.62848640933,0,0
154.475000000018,0,1168.62848640933,0,0
154.500000000018,0,1168.62848640933,0,0
154.525000000018,0,1168.62848640933,0,0
154.550000000018,0,1168.62848640933,0,0
154.575000000018,0,1168.62848640933,0,0
154.600000000018,0,1168.62848640933,0,0
154.625000000018,0,1168.62848640933,0,0

154.650000000018,0,1168.62848640933,0,0
154.675000000018,0,1168.62848640933,0,0
154.700000000018,0,1168.62848640933,0,0
154.725000000018,0,1168.62848640933,0,0
154.750000000018,0,1168.62848640933,0,0
154.775000000018,0,1168.62848640933,0,0
154.800000000018,0,1168.62848640933,0,0
154.825000000018,1,1168.71061140933,0,0
154.850000000018,0,1168.79273640933,0,0
154.875000000018,0,1168.79273640933,0,0
154.900000000018,0,1168.79273640933,0,0
154.925000000018,0,1168.79273640933,0,0
154.950000000018,0,1168.79273640933,0,0
154.975000000018,0,1168.79273640933,0,0
155.000000000018,0,1168.79273640933,0,0
155.025000000018,0,1168.79273640933,0,0
155.050000000018,0,1168.79273640933,0,0
155.075000000018,0,1168.79273640933,0,0
155.100000000018,0,1168.79273640933,0,0
155.125000000018,0,1168.79273640933,0,0
155.150000000018,0,1168.79273640933,0,0
155.175000000018,0,1168.79273640933,0,0
155.200000000018,0,1168.79273640933,0,0
155.225000000018,0,1168.79273640933,0,0
155.250000000018,0,1168.79273640933,0,0
155.275000000018,0,1168.79273640933,0,0
155.300000000018,0,1168.79273640933,0,0
155.325000000018,0,1168.79273640933,0,0
155.350000000018,0,1168.79273640933,0,0
155.375000000018,0,1168.79273640933,0,0
155.400000000018,0,1168.79273640933,0,0
155.425000000018,0,1168.79273640933,0,0
155.450000000018,0,1168.79273640933,0,0
155.475000000018,0,1168.79273640933,0,0
155.500000000018,0,1168.79273640933,0,0
155.525000000018,0,1168.79273640933,0,0
155.550000000018,0,1168.79273640933,0,0
155.575000000018,0,1168.79273640933,0,0
155.600000000018,0,1168.79273640933,0,0
155.625000000018,0,1168.79273640933,0,0
155.650000000018,0,1168.79273640933,0,0
155.675000000018,0,1168.79273640933,0,0
155.700000000018,0,1168.79273640933,0,0
155.725000000018,0,1168.79273640933,0,0
155.750000000018,0,1168.79273640933,0,0
155.775000000018,0,1168.79273640933,0,0
155.800000000018,0,1168.79273640933,0,0
155.825000000018,0,1168.79273640933,0,0
155.850000000018,0,1168.79273640933,0,0
155.875000000018,0,1168.79273640933,0,0
155.900000000018,0,1168.79273640933,0,0
155.925000000018,0,1168.79273640933,0,0

155.950000000018,0,1168.79273640933,0,0
155.975000000018,0,1168.79273640933,0,0
156.000000000018,0,1168.79273640933,0,0
156.025000000018,0,1168.79273640933,0,0
156.050000000018,0,1168.79273640933,0,0
156.075000000018,0,1168.79273640933,0,0
156.100000000018,0,1168.79273640933,0,0
156.125000000018,0,1168.79273640933,0,0
156.150000000018,0,1168.79273640933,0,0
156.175000000018,0,1168.79273640933,0,0
156.200000000018,0,1168.79273640933,0,0
156.225000000018,0,1168.79273640933,0,0
156.250000000018,0,1168.79273640933,0,0
156.275000000018,0,1168.79273640933,0,0
156.300000000018,0,1168.79273640933,0,0
156.325000000018,0,1168.79273640933,0,0
156.350000000018,0,1168.79273640933,0,0
156.375000000018,0,1168.79273640933,0,0
156.400000000018,1,1168.87486140933,0,0
156.425000000018,0,1168.95698640933,0,0
156.450000000018,0,1168.95698640933,0,0
156.475000000018,0,1168.95698640933,0,0
156.500000000018,0,1168.95698640933,0,0
156.525000000018,0,1168.95698640933,0,0
156.550000000018,0,1168.95698640933,0,0
156.575000000018,0,1168.95698640933,0,0
156.600000000018,0,1168.95698640933,0,0
156.625000000018,0,1168.95698640933,0,0
156.650000000018,2,1169.07312869814,0,0
156.675000000018,3,1169.33151565952,0,0
156.700000000018,0,1169.4737603321,0,0
156.725000000019,0,1169.4737603321,0,0
156.750000000019,0,1169.4737603321,0,0
156.775000000019,2,1169.58990262091,0,0
156.800000000019,1,1169.78816990972,0,0
156.825000000019,1,1169.95241990972,0,0
156.850000000019,0,1170.03454490972,0,0
156.875000000019,1,1170.11666990972,0,0
156.900000000019,1,1170.28091990972,0,0
156.925000000019,0,1170.36304490972,0,0
156.950000000019,0,1170.36304490972,0,0
156.975000000019,0,1170.36304490972,0,0
157.000000000019,0,1170.36304490972,0,0
157.025000000019,1,1170.44516990972,0,0
157.050000000019,0,1170.52729490972,0,0
157.075000000019,0,1170.52729490972,0,0
157.100000000019,1,1170.60941990972,0,0
157.125000000019,0,1170.69154490972,0,0
157.150000000019,0,1170.69154490972,0,0
157.175000000019,0,1170.69154490972,0,0
157.200000000019,0,1170.69154490972,0,0
157.225000000019,0,1170.69154490972,0,0

157.250000000019,0,1170.69154490972,0,0
157.275000000019,0,1170.69154490972,0,0
157.300000000019,0,1170.69154490972,0,0
157.325000000019,0,1170.69154490972,0,0
157.350000000019,0,1170.69154490972,0,0
157.375000000019,0,1170.69154490972,0,0
157.400000000019,0,1170.69154490972,0,0
157.425000000019,0,1170.69154490972,0,0
157.450000000019,0,1170.69154490972,0,0
157.475000000019,0,1170.69154490972,0,0
157.500000000019,0,1170.69154490972,0,0
157.525000000019,0,1170.69154490972,0,0
157.550000000019,0,1170.69154490972,0,0
157.575000000019,0,1170.69154490972,0,0
157.600000000019,0,1170.69154490972,0,0
157.625000000019,0,1170.69154490972,0,0
157.650000000019,0,1170.69154490972,0,0
157.675000000019,0,1170.69154490972,0,0
157.700000000019,0,1170.69154490972,0,0
157.725000000019,0,1170.69154490972,0,0
157.750000000019,0,1170.69154490972,0,0
157.775000000019,0,1170.69154490972,0,0
157.800000000019,0,1170.69154490972,0,0
157.825000000019,0,1170.69154490972,0,0
157.850000000019,0,1170.69154490972,0,0
157.875000000019,0,1170.69154490972,0,0
157.900000000019,0,1170.69154490972,0,0
157.925000000019,0,1170.69154490972,0,0
157.950000000019,0,1170.69154490972,0,0
157.975000000019,0,1170.69154490972,0,0
158.000000000019,0,1170.69154490972,0,0
158.025000000019,0,1170.69154490972,0,0
158.050000000019,0,1170.69154490972,0,0
158.075000000019,0,1170.69154490972,0,0
158.100000000019,0,1170.69154490972,0,0
158.125000000019,0,1170.69154490972,0,0
158.150000000019,0,1170.69154490972,0,0
158.175000000019,0,1170.69154490972,0,0
158.200000000019,0,1170.69154490972,0,0
158.225000000019,0,1170.69154490972,0,0
158.250000000019,0,1170.69154490972,0,0
158.275000000019,0,1170.69154490972,0,0
158.300000000019,1,1170.77366990972,0,0
158.325000000019,0,1170.85579490971,0,0
158.350000000019,0,1170.85579490971,0,0
158.375000000019,0,1170.85579490971,0,0
158.400000000019,0,1170.85579490971,0,0
158.425000000019,0,1170.85579490971,0,0
158.450000000019,0,1170.85579490971,0,0
158.475000000019,0,1170.85579490971,0,0
158.500000000019,0,1170.85579490971,0,0
158.525000000019,0,1170.85579490971,0,0

158.550000000019,0,1170.85579490971,0,0
158.575000000019,0,1170.85579490971,0,0
158.600000000019,0,1170.85579490971,0,0
158.625000000019,0,1170.85579490971,0,0
158.650000000019,0,1170.85579490971,0,0
158.675000000019,1,1170.93791990971,0,0
158.700000000019,0,1171.02004490971,0,0
158.725000000019,0,1171.02004490971,0,0
158.750000000019,0,1171.02004490971,0,0
158.775000000019,0,1171.02004490971,0,0
158.800000000019,0,1171.02004490971,0,0
158.825000000019,0,1171.02004490971,0,0
158.850000000019,0,1171.02004490971,0,0
158.875000000019,0,1171.02004490971,0,0
158.900000000019,0,1171.02004490971,0,0
158.925000000019,0,1171.02004490971,0,0
158.950000000019,0,1171.02004490971,0,0
158.975000000019,0,1171.02004490971,0,0
159.000000000019,0,1171.02004490971,0,0
159.025000000019,0,1171.02004490971,0,0
159.050000000019,0,1171.02004490971,0,0
159.075000000019,0,1171.02004490971,0,0
159.100000000019,0,1171.02004490971,0,0
159.125000000019,0,1171.02004490971,0,0
159.150000000019,0,1171.02004490971,0,0
159.175000000019,0,1171.02004490971,0,0
159.200000000019,0,1171.02004490971,0,0
159.225000000019,0,1171.02004490971,0,0
159.250000000019,0,1171.02004490971,0,0
159.275000000019,0,1171.02004490971,0,0
159.300000000019,1,1171.10216990971,0,0
159.325000000019,0,1171.18429490971,0,0
159.350000000019,0,1171.18429490971,0,0
159.375000000019,0,1171.18429490971,0,0
159.400000000019,0,1171.18429490971,0,0
159.425000000019,1,1171.26641990971,0,0
159.450000000019,0,1171.34854490971,0,0
159.475000000019,0,1171.34854490971,0,0
159.500000000019,0,1171.34854490971,0,0
159.525000000019,0,1171.34854490971,0,0
159.550000000019,0,1171.34854490971,0,0
159.575000000019,0,1171.34854490971,0,0
159.600000000019,0,1171.34854490971,0,0
159.625000000019,0,1171.34854490971,0,0
159.650000000019,0,1171.34854490971,0,0
159.675000000019,0,1171.34854490971,0,0
159.700000000019,0,1171.34854490971,0,0
159.725000000019,1,1171.43066990971,0,0
159.750000000019,0,1171.51279490971,0,0
159.775000000019,0,1171.51279490971,0,0
159.800000000019,0,1171.51279490971,0,0
159.825000000019,0,1171.51279490971,0,0

159.850000000019,0,1171.51279490971,0,0
159.875000000019,0,1171.51279490971,0,0
159.900000000019,0,1171.51279490971,0,0
159.925000000019,0,1171.51279490971,0,0
159.950000000019,0,1171.51279490971,0,0
159.975000000019,0,1171.51279490971,0,0
160.000000000019,0,1171.51279490971,0,0
160.025000000019,0,1171.51279490971,0,0
160.050000000019,0,1171.51279490971,0,0
160.075000000019,0,1171.51279490971,0,0
160.100000000019,0,1171.51279490971,0,0
160.125000000019,0,1171.51279490971,0,0
160.150000000019,1,1171.59491990971,0,0
160.175000000019,0,1171.67704490971,0,0
160.200000000019,0,1171.67704490971,0,0
160.225000000019,0,1171.67704490971,0,0
160.250000000019,0,1171.67704490971,0,0
160.275000000019,0,1171.67704490971,0,0
160.300000000019,0,1171.67704490971,0,0
160.325000000019,1,1171.75916990971,0,0
160.350000000019,0,1171.84129490971,0,0
160.375000000019,0,1171.84129490971,0,0
160.400000000019,0,1171.84129490971,0,0
160.425000000019,0,1171.84129490971,0,0
160.450000000019,8,1172.07357948733,0,0
160.475000000019,0,1172.30586406495,0,0
160.500000000019,1,1172.38798906495,0,0
160.525000000019,0,1172.47011406495,0,0
160.550000000019,0,1172.47011406495,0,0
160.575000000019,1,1172.55223906495,0,0
160.600000000019,0,1172.63436406495,0,0
160.625000000019,0,1172.63436406495,0,0
160.650000000019,0,1172.63436406495,0,0
160.675000000019,1,1172.71648906495,0,0
160.700000000019,5,1172.98225114761,0,0
160.725000000019,1,1173.24801323026,0,0
160.750000000019,2,1173.44628051907,0,0
160.775000000019,0,1173.56242280788,0,0
160.800000000019,0,1173.56242280788,0,0
160.825000000019,0,1173.56242280788,0,0
160.850000000019,0,1173.56242280788,0,0
160.875000000019,0,1173.56242280788,0,0
160.900000000019,0,1173.56242280788,0,0
160.925000000019,0,1173.56242280788,0,0
160.950000000019,0,1173.56242280788,0,0
160.975000000019,0,1173.56242280788,0,0
161.000000000019,0,1173.56242280788,0,0
161.025000000019,0,1173.56242280788,0,0
161.050000000019,0,1173.56242280788,0,0
161.075000000019,0,1173.56242280788,0,0
161.100000000019,0,1173.56242280788,0,0
161.12500000002,0,1173.56242280788,0,0

161.15000000002,0,1173.56242280788,0,0
161.17500000002,0,1173.56242280788,0,0
161.20000000002,0,1173.56242280788,0,0
161.22500000002,0,1173.56242280788,0,0
161.25000000002,0,1173.56242280788,0,0
161.27500000002,0,1173.56242280788,0,0
161.30000000002,2,1173.67856509669,0,0
161.32500000002,0,1173.7947073855,0,0
161.35000000002,0,1173.7947073855,0,0
161.37500000002,0,1173.7947073855,0,0
161.40000000002,0,1173.7947073855,0,0
161.42500000002,0,1173.7947073855,0,0
161.45000000002,0,1173.7947073855,0,0
161.47500000002,0,1173.7947073855,0,0
161.50000000002,0,1173.7947073855,0,0
161.52500000002,0,1173.7947073855,0,0
161.55000000002,0,1173.7947073855,0,0
161.57500000002,0,1173.7947073855,0,0
161.60000000002,0,1173.7947073855,0,0
161.62500000002,0,1173.7947073855,0,0
161.65000000002,0,1173.7947073855,0,0
161.67500000002,0,1173.7947073855,0,0
161.70000000002,0,1173.7947073855,0,0
161.72500000002,0,1173.7947073855,0,0
161.75000000002,0,1173.7947073855,0,0
161.77500000002,0,1173.7947073855,0,0
161.80000000002,0,1173.7947073855,0,0
161.82500000002,0,1173.7947073855,0,0
161.85000000002,0,1173.7947073855,0,0
161.87500000002,0,1173.7947073855,0,0
161.90000000002,0,1173.7947073855,0,0
161.92500000002,0,1173.7947073855,0,0
161.95000000002,0,1173.7947073855,0,0
161.97500000002,0,1173.7947073855,0,0
162.00000000002,0,1173.7947073855,0,0
162.02500000002,0,1173.7947073855,0,0
162.05000000002,0,1173.7947073855,0,0
162.07500000002,0,1173.7947073855,0,0
162.10000000002,0,1173.7947073855,0,0
162.12500000002,0,1173.7947073855,0,0
162.15000000002,0,1173.7947073855,0,0
162.17500000002,0,1173.7947073855,0,0
162.20000000002,0,1173.7947073855,0,0
162.22500000002,0,1173.7947073855,0,0
162.25000000002,0,1173.7947073855,0,0
162.27500000002,0,1173.7947073855,0,0
162.30000000002,0,1173.7947073855,0,0
162.32500000002,0,1173.7947073855,0,0
162.35000000002,0,1173.7947073855,0,0
162.37500000002,0,1173.7947073855,0,0
162.40000000002,0,1173.7947073855,0,0
162.42500000002,0,1173.7947073855,0,0

```
162.45000000002,0,1173.7947073855,0,0
162.47500000002,0,1173.7947073855,0,0
162.50000000002,0,1173.7947073855,0,0
162.52500000002,0,1173.7947073855,0,0
162.55000000002,0,1173.7947073855,0,0
162.57500000002,0,1173.7947073855,0,0
162.60000000002,0,1173.7947073855,0,0
162.62500000002,0,1173.7947073855,0,0
162.65000000002,0,1173.7947073855,0,0
162.67500000002,0,1173.7947073855,0,0
162.70000000002,0,1173.7947073855,0,0
162.72500000002,0,1173.7947073855,0,0
162.75000000002,0,1173.7947073855,0,0
162.77500000002,0,1173.7947073855,0,0
162.80000000002,1,1173.8768323855,0,0
162.82500000002,0,1173.9589573855,0,0
162.85000000002,0,1173.9589573855,0,0
162.87500000002,0,1173.9589573855,0,0
162.90000000002,0,1173.9589573855,0,0
162.92500000002,1,1174.0410823855,0,0
162.95000000002,0,1174.1232073855,0,0
162.97500000002,0,1174.1232073855,0,0
163.00000000002,0,1174.1232073855,0,0
163.02500000002,0,1174.1232073855,0,0
163.05000000002,1,1174.2053323855,0,0
163.07500000002,3,1174.42970205807,0,0
163.10000000002,4,1174.73619673064,0,0
163.12500000002,5,1175.08408381329,0,0
163.15000000002,6,1175.46888524107,0,0
163.17500000002,6,1175.87121393132,0,0
163.20000000002,11,1176.34475608736,26.0067147467872,1
163.22500000002,12,1176.90162324341,26.0067147467872,1
163.25000000002,12,1177.47060193369,26.0067147467872,1
163.27500000002,11,1178.02746908974,26.0067147467872,1
163.30000000002,9,1178.54622190065,26.0067147467872,1
163.32500000002,9,1179.03897190065,26.0067147467872,1
163.35000000002,9,1179.53172190065,26.0067147467872,1
163.37500000002,10,1180.03779895349,26.0067147467872,1
163.40000000002,10,1180.55720305918,26.0067147467872,1
163.42500000002,11,1181.08928292293,26.0067147467872,1
163.45000000002,11,1181.63403854474,26.0067147467872,1
163.47500000002,13,1182.20252225415,26.0067147467872,1
163.50000000002,15,1182.81669690995,26.0067147467872,1
163.52500000002,16,1183.46326566726,26.0067147467872,1
163.55000000002,16,1184.12026566726,26.0067147467872,1
163.57500000002,16,1184.77726566726,26.0067147467872,1
163.60000000002,16,1185.43426566726,26.0067147467872,1
163.62500000002,16,1186.09126566726,26.0067147467872,1
163.65000000002,16,1186.74826566726,26.0067147467872,1
163.67500000002,15,1187.39483442457,26.0067147467872,1
163.70000000002,13,1188.00900908037,26.0067147467872,1
163.72500000002,13,1188.60122087737,26.0067147467872,1
```

163.75000000002,13,1189.19343267436,26.0067147467872,1
163.77500000002,12,1189.774027918,26.0067147467872,1
163.80000000002,13,1190.35462316164,26.0067147467872,1
163.82500000002,14,1190.95801267303,26.0067147467872,1
163.85000000002,15,1191.58336504322,26.0067147467872,1
163.87500000002,16,1192.22993380053,26.0067147467872,1
163.90000000002,16,1192.88693380053,26.0067147467872,1
163.92500000002,15,1193.53350255784,26.0067147467872,1
163.95000000002,16,1194.18007131515,26.0067147467872,1
163.97500000002,13,1194.80467721364,26.0067147467872,1
164.00000000002,10,1195.36048516498,26.0067147467872,1
164.02500000002,10,1195.87988927067,26.0067147467872,1
164.05000000002,11,1196.41196913441,26.0067147467872,1
164.07500000002,13,1196.98045284382,26.0067147467872,1
164.10000000002,14,1197.58384235521,26.0067147467872,1
164.12500000002,16,1198.21962596809,26.0067147467872,1
164.15000000002,16,1198.87662596809,26.0067147467872,1
164.17500000002,18,1199.55355283452,26.0067147467872,1
164.20000000002,23,1200.29583736481,26.0067147467872,1
164.22500000002,18,1201.03812189509,26.0067147467872,1
164.25000000002,15,1201.70461751882,26.0067147467872,1
164.27500000002,16,1202.35118627613,26.0067147467872,1
164.30000000002,19,1203.03766085187,26.0067147467872,1
164.32500000002,20,1203.76290959291,26.0067147467872,1
164.35000000002,28,1204.56474841106,26.0067147467872,1
164.37500000002,19,1205.35728763964,26.0067147467872,1
164.40000000002,20,1206.08253638068,26.0067147467872,1
164.42500000002,20,1206.81708471129,26.0067147467872,1
164.45000000002,24,1207.58668756685,26.0067147467872,1
164.47500000002,19,1208.34699083284,26.0067147467872,1
164.50000000002,24,1209.10729409883,26.0067147467872,1
164.52500000002,25,1209.92024778908,26.0067147467872,1
164.55000000002,23,1210.72473045293,26.0067147467872,1
164.57500000002,23,1211.51244578064,26.0067147467872,1
164.60000000002,21,1212.28264747344,26.0067147467872,1
164.62500000002,21,1213.03533553133,26.0067147467872,1
164.65000000002,22,1213.79687995456,26.0067147467872,1
164.67500000002,20,1214.54935451414,26.0067147467872,1
164.70000000002,21,1215.29297270839,26.0067147467872,1
164.72500000002,21,1216.04566076629,26.0067147467872,1
164.75000000002,21,1216.79834882418,26.0067147467872,1
164.77500000002,19,1217.53266742887,26.0067147467872,1
164.80000000002,19,1218.24861658035,26.0067147467872,1
164.82500000002,18,1218.95501802251,26.0067147467872,1
164.85000000002,18,1219.65187175537,26.0067147467872,1
164.87500000002,18,1220.34872548823,26.0067147467872,1
164.90000000002,18,1221.04557922109,26.0067147467872,1
164.92500000002,16,1221.72250608752,26.0067147467872,1
164.95000000002,15,1222.36907484483,26.0067147467872,1
164.97500000002,15,1223.00521235944,26.0067147467872,1
165.00000000002,13,1223.61938701525,26.0067147467872,1
165.02500000002,15,1224.23356167105,26.0067147467872,1

```
165.05000000002,13,1224.84773632686,26.0067147467872,1
165.07500000002,13,1225.43994812385,26.0067147467872,1
165.10000000002,11,1226.00843183326,26.0067147467872,1
165.12500000002,11,1226.55318745507,26.0067147467872,1
165.15000000002,8,1227.0578498436,26.0067147467872,1
165.17500000002,5,1227.47377150387,0,0
165.20000000002,3,1227.7996532591,0,0
165.22500000002,2,1228.05804022048,0,0
165.25000000002,1,1228.25630750929,0,0
165.27500000002,1,1228.42055750929,0,0
165.30000000002,0,1228.50268250929,0,0
165.32500000002,0,1228.50268250929,0,0
165.35000000002,0,1228.50268250929,0,0
165.37500000002,0,1228.50268250929,0,0
165.40000000002,0,1228.50268250929,0,0
165.42500000002,0,1228.50268250929,0,0
165.45000000002,0,1228.50268250929,0,0
165.47500000002,0,1228.50268250929,0,0
165.50000000002,0,1228.50268250929,0,0
165.525000000021,0,1228.50268250929,0,0
165.550000000021,0,1228.50268250929,0,0
165.575000000021,0,1228.50268250929,0,0
165.600000000021,0,1228.50268250929,0,0
165.625000000021,0,1228.50268250929,0,0
165.650000000021,0,1228.50268250929,0,0
165.675000000021,0,1228.50268250929,0,0
165.700000000021,0,1228.50268250929,0,0
165.725000000021,0,1228.50268250929,0,0
165.750000000021,0,1228.50268250929,0,0
165.775000000021,0,1228.50268250929,0,0
165.800000000021,0,1228.50268250929,0,0
165.825000000021,0,1228.50268250929,0,0
165.850000000021,0,1228.50268250929,0,0
165.875000000021,0,1228.50268250929,0,0
165.900000000021,0,1228.50268250929,0,0
165.925000000021,0,1228.50268250929,0,0
165.950000000021,1,1228.58480750929,0,0
165.975000000021,0,1228.66693250929,0,0
166.000000000021,0,1228.66693250929,0,0
166.025000000021,0,1228.66693250929,0,0
166.050000000021,0,1228.66693250929,0,0
166.075000000021,0,1228.66693250929,0,0
166.100000000021,0,1228.66693250929,0,0
166.125000000021,0,1228.66693250929,0,0
166.150000000021,0,1228.66693250929,0,0
166.175000000021,0,1228.66693250929,0,0
166.200000000021,0,1228.66693250929,0,0
166.225000000021,0,1228.66693250929,0,0
166.250000000021,1,1228.74905750929,0,0
166.275000000021,0,1228.83118250929,0,0
166.300000000021,0,1228.83118250929,0,0
166.325000000021,0,1228.83118250929,0,0
```

166.350000000021,0,1228.83118250929,0,0
166.375000000021,0,1228.83118250929,0,0
166.400000000021,0,1228.83118250929,0,0
166.425000000021,0,1228.83118250929,0,0
166.450000000021,0,1228.83118250929,0,0
166.475000000021,0,1228.83118250929,0,0
166.500000000021,0,1228.83118250929,0,0
166.525000000021,0,1228.83118250929,0,0
166.550000000021,0,1228.83118250929,0,0
166.575000000021,0,1228.83118250929,0,0
166.600000000021,0,1228.83118250929,0,0
166.625000000021,1,1228.91330750929,0,0
166.650000000021,0,1228.99543250929,0,0
166.675000000021,0,1228.99543250929,0,0
166.700000000021,0,1228.99543250929,0,0
166.725000000021,0,1228.99543250929,0,0
166.750000000021,0,1228.99543250929,0,0
166.775000000021,0,1228.99543250929,0,0
166.800000000021,0,1228.99543250929,0,0
166.825000000021,0,1228.99543250929,0,0
166.850000000021,0,1228.99543250929,0,0
166.875000000021,0,1228.99543250929,0,0
166.900000000021,0,1228.99543250929,0,0
166.925000000021,0,1228.99543250929,0,0
166.950000000021,0,1228.99543250929,0,0
166.975000000021,0,1228.99543250929,0,0
167.000000000021,0,1228.99543250929,0,0
167.025000000021,0,1228.99543250929,0,0
167.050000000021,0,1228.99543250929,0,0
167.075000000021,0,1228.99543250929,0,0
167.100000000021,0,1228.99543250929,0,0
167.125000000021,0,1228.99543250929,0,0
167.150000000021,0,1228.99543250929,0,0
167.175000000021,0,1228.99543250929,0,0
167.200000000021,0,1228.99543250929,0,0
167.225000000021,0,1228.99543250929,0,0
167.250000000021,0,1228.99543250929,0,0
167.275000000021,1,1229.07755750929,0,0
167.300000000021,1,1229.24180750929,0,0
167.325000000021,1,1229.40605750929,0,0
167.350000000021,3,1229.63042718186,0,0
167.375000000021,5,1229.95630893708,0,0
167.400000000021,7,1230.35722834616,0,0
167.425000000021,10,1230.83421272542,23.3219474047261,1
167.450000000021,11,1231.36629258917,23.3219474047261,1
167.475000000021,11,1231.91104821099,23.3219474047261,1
167.500000000021,13,1232.47953192039,23.3219474047261,1
167.525000000021,14,1233.08292143178,23.3219474047261,1
167.550000000021,15,1233.70827380197,23.3219474047261,1
167.575000000021,18,1234.37476942571,23.3219474047261,1
167.600000000021,21,1235.09954032109,23.3219474047261,1
167.625000000021,19,1235.83385892577,23.3219474047261,1

167.650000000021,20,1236.55910766682,23.3219474047261,1
167.675000000021,21,1237.30272586107,23.3219474047261,1
167.700000000021,19,1238.03704446576,23.3219474047261,1
167.725000000021,18,1238.74344590792,23.3219474047261,1
167.750000000021,17,1239.43048282386,23.3219474047261,1
167.775000000021,13,1240.06519877186,23.3219474047261,1
167.800000000021,11,1240.63368248126,23.3219474047261,1
167.825000000021,10,1241.16576234501,23.3219474047261,1
167.850000000021,10,1241.6851664507,23.3219474047261,1
167.875000000021,7,1242.16215082996,23.3219474047261,1
167.900000000021,12,1242.66392250152,23.3219474047261,1
167.925000000021,14,1243.25569545956,23.3219474047261,1
167.950000000021,12,1243.84746841759,23.3219474047261,1
167.975000000021,8,1244.36424234035,23.3219474047261,1
168.000000000021,12,1244.88101626311,23.3219474047261,1
168.025000000021,11,1245.43788341916,23.3219474047261,1
168.050000000021,12,1245.99475057522,23.3219474047261,1
168.075000000021,11,1246.55161773127,23.3219474047261,1
168.100000000021,11,1247.09637335308,23.3219474047261,1
168.125000000021,6,1247.56991550912,23.3219474047261,1
168.150000000021,9,1248.01745485424,23.3219474047261,1
168.175000000021,7,1248.48111218066,23.3219474047261,1
168.200000000021,13,1248.99450040558,23.3219474047261,1
168.225000000021,6,1249.49177064921,23.3219474047261,1
168.250000000021,5,1249.87657207698,0,0
168.275000000021,8,1250.29249373726,0,0
168.300000000021,8,1250.7570628925,0,0
168.325000000021,8,1251.22163204773,0,0
168.350000000021,6,1251.65508097048,0,0
168.375000000021,7,1252.07352764203,0,0
168.400000000021,7,1252.50809229487,0,0
168.425000000021,6,1252.92653896642,0,0
168.450000000021,6,1253.32886765667,0,0
168.475000000021,5,1253.71366908445,0,0
168.500000000021,4,1254.0615561671,0,0
168.525000000021,4,1254.3900561671,0,0
168.550000000021,4,1254.7185561671,0,0
168.575000000021,4,1255.0470561671,0,0
168.600000000021,3,1255.35355083967,0,0
168.625000000021,4,1255.66004551224,0,0
168.650000000021,4,1255.98854551224,0,0
168.675000000021,5,1256.3364325949,0,0
168.700000000021,6,1256.72123402267,0,0
168.725000000021,7,1257.13968069422,0,0
168.750000000021,7,1257.57424534706,0,0
168.775000000021,6,1257.99269201861,0,0
168.800000000021,4,1258.35810636374,0,0
168.825000000021,6,1258.72352070886,0,0
168.850000000021,6,1259.12584939911,0,0
168.875000000021,6,1259.52817808937,0,0
168.900000000021,6,1259.93050677962,0,0
168.925000000021,6,1260.33283546987,0,0

168.950000000021,7,1260.75128214142,0,0
168.975000000021,10,1261.22826652068,21.4652008681193,1
169.000000000021,11,1261.76034638443,21.4652008681193,1
169.025000000021,11,1262.30510200625,21.4652008681193,1
169.050000000021,11,1262.84985762806,21.4652008681193,1
169.075000000021,11,1263.39461324988,21.4652008681193,1
169.100000000021,10,1263.92669311363,21.4652008681193,1
169.125000000021,11,1264.45877297738,21.4652008681193,1
169.150000000021,11,1265.00352859919,21.4652008681193,1
169.175000000021,11,1265.54828422101,21.4652008681193,1
169.200000000021,11,1266.09303984282,21.4652008681193,1
169.225000000021,10,1266.62511970657,21.4652008681193,1
169.250000000021,9,1267.13119675941,21.4652008681193,1
169.275000000021,7,1267.59485408584,0,0
169.300000000021,7,1268.02941873868,0,0
169.325000000021,7,1268.46398339152,0,0
169.350000000021,7,1268.89854804436,0,0
169.375000000021,8,1269.3481149484,0,0
169.400000000021,8,1269.81268410364,0,0
169.425000000021,8,1270.27725325888,0,0
169.450000000021,8,1270.74182241412,0,0
169.475000000021,9,1271.22048199174,0,0
169.500000000021,8,1271.69914156936,0,0
169.525000000021,8,1272.1637107246,0,0
169.550000000021,9,1272.64237030222,0,0
169.575000000021,9,1273.13512030222,0,0
169.600000000021,10,1273.64119735506,22.4358240133015,1
169.625000000021,11,1274.17327721881,22.4358240133015,1
169.650000000021,12,1274.73014437486,22.4358240133015,1
169.675000000021,13,1275.3107396185,22.4358240133015,1
169.700000000021,12,1275.89133486214,22.4358240133015,1
169.725000000021,13,1276.47193010578,22.4358240133015,1
169.750000000021,12,1277.05252534942,22.4358240133015,1
169.775000000021,12,1277.62150403971,22.4358240133015,1
169.800000000021,12,1278.19048273,22.4358240133015,1
169.825000000021,12,1278.75946142028,22.4358240133015,1
169.850000000021,13,1279.34005666392,22.4358240133015,1
169.875000000021,13,1279.93226846092,22.4358240133015,1
169.900000000021,13,1280.52448025791,22.4358240133015,1
169.925000000022,14,1281.1278697693,22.4358240133015,1
169.950000000022,13,1281.73125928069,22.4358240133015,1
169.975000000022,13,1282.32347107768,22.4358240133015,1
170.000000000022,12,1282.90406632132,22.4358240133015,1
170.025000000022,12,1283.47304501161,22.4358240133015,1
170.050000000022,12,1284.0420237019,22.4358240133015,1
170.075000000022,12,1284.61100239218,22.4358240133015,1
170.100000000022,10,1285.15519379017,22.4358240133015,1
170.125000000022,10,1285.67459789585,22.4358240133015,1
170.150000000022,10,1286.19400200153,22.4358240133015,1
170.175000000022,9,1286.70007905437,22.4358240133015,1
170.200000000022,10,1287.20615610721,22.4358240133015,1
170.225000000022,10,1287.7255602129,22.4358240133015,1

170.250000000022,10,1288.24496431858,22.4358240133015,1
170.275000000022,12,1288.78915571656,22.4358240133015,1
170.300000000022,13,1289.36975096021,22.4358240133015,1
170.325000000022,14,1289.97314047159,22.4358240133015,1
170.350000000022,12,1290.56491342962,22.4358240133015,1
170.375000000022,12,1291.13389211991,22.4358240133015,1
170.400000000022,12,1291.7028708102,22.4358240133015,1
170.425000000022,10,1292.24706220818,22.4358240133015,1
170.450000000022,10,1292.76646631386,22.4358240133015,1
170.475000000022,9,1293.2725433667,22.4358240133015,1
170.500000000022,8,1293.75120294432,0,0
170.525000000022,9,1294.22986252194,0,0
170.550000000022,9,1294.72261252194,0,0
170.575000000022,8,1295.20127209956,0,0
170.600000000022,8,1295.6658412548,0,0
170.625000000022,8,1296.13041041004,0,0
170.650000000022,8,1296.59497956528,0,0
170.675000000022,7,1297.04454646932,0,0
170.700000000022,8,1297.49411337336,0,0
170.725000000022,8,1297.9586825286,0,0
170.750000000022,8,1298.42325168384,0,0
170.775000000022,6,1298.85670060659,0,0
170.800000000022,6,1299.25902929684,0,0
170.825000000022,3,1299.60243831454,0,0
170.850000000022,1,1299.82680798711,0,0
170.875000000022,1,1299.99105798711,0,0
170.900000000022,0,1300.07318298711,0,0
170.925000000022,0,1300.07318298711,0,0
170.950000000022,0,1300.07318298711,0,0
170.975000000022,0,1300.07318298711,0,0
171.000000000022,0,1300.07318298711,0,0
171.025000000022,0,1300.07318298711,0,0
171.050000000022,0,1300.07318298711,0,0
171.075000000022,0,1300.07318298711,0,0
171.100000000022,0,1300.07318298711,0,0
171.125000000022,0,1300.07318298711,0,0
171.150000000022,0,1300.07318298711,0,0
171.175000000022,0,1300.07318298711,0,0
171.200000000022,0,1300.07318298711,0,0
171.225000000022,0,1300.07318298711,0,0
171.250000000022,1,1300.15530798711,0,0
171.275000000022,0,1300.23743298711,0,0
171.300000000022,0,1300.23743298711,0,0
171.325000000022,0,1300.23743298711,0,0
171.350000000022,0,1300.23743298711,0,0
171.375000000022,1,1300.31955798711,0,0
171.400000000022,0,1300.40168298711,0,0
171.425000000022,0,1300.40168298711,0,0
171.450000000022,0,1300.40168298711,0,0
171.475000000022,1,1300.48380798711,0,0
171.500000000022,0,1300.56593298711,0,0
171.525000000022,0,1300.56593298711,0,0

171.550000000022,0,1300.56593298711,0,0
171.575000000022,0,1300.56593298711,0,0
171.600000000022,0,1300.56593298711,0,0
171.625000000022,2,1300.68207527592,0,0
171.650000000022,0,1300.79821756473,0,0
171.675000000022,1,1300.88034256473,0,0
171.700000000022,0,1300.96246756473,0,0
171.725000000022,0,1300.96246756473,0,0
171.750000000022,0,1300.96246756473,0,0
171.775000000022,0,1300.96246756473,0,0
171.800000000022,0,1300.96246756473,0,0
171.825000000022,0,1300.96246756473,0,0
171.850000000022,0,1300.96246756473,0,0
171.875000000022,0,1300.96246756473,0,0
171.900000000022,0,1300.96246756473,0,0
171.925000000022,2,1301.07860985354,0,0
171.950000000022,0,1301.19475214235,0,0
171.975000000022,0,1301.19475214235,0,0
172.000000000022,0,1301.19475214235,0,0
172.025000000022,1,1301.27687714235,0,0
172.050000000022,0,1301.35900214235,0,0
172.075000000022,0,1301.35900214235,0,0
172.100000000022,0,1301.35900214235,0,0
172.125000000022,0,1301.35900214235,0,0
172.150000000022,0,1301.35900214235,0,0
172.175000000022,0,1301.35900214235,0,0
172.200000000022,0,1301.35900214235,0,0
172.225000000022,0,1301.35900214235,0,0
172.250000000022,0,1301.35900214235,0,0
172.275000000022,0,1301.35900214235,0,0
172.300000000022,2,1301.47514443116,0,0
172.325000000022,5,1301.77492380262,0,0
172.350000000022,9,1302.20493588527,0,0
172.375000000022,12,1302.73580023042,27.188444176574,1
172.400000000022,20,1303.38756374086,27.188444176574,1
172.425000000022,20,1304.12211207147,27.188444176574,1
172.450000000022,19,1304.84736081252,27.188444176574,1
172.475000000022,20,1305.57260955356,27.188444176574,1
172.500000000022,21,1306.31622774781,27.188444176574,1
172.525000000022,21,1307.06891580571,27.188444176574,1
172.550000000022,24,1307.84758852491,27.188444176574,1
172.575000000022,23,1308.64377487901,27.188444176574,1
172.600000000022,23,1309.43149020672,27.188444176574,1
172.625000000022,23,1310.21920553442,27.188444176574,1
172.650000000022,20,1310.98033736358,27.188444176574,1
172.675000000022,18,1311.69603839531,27.188444176574,1
172.700000000022,17,1312.38307531124,27.188444176574,1
172.725000000022,16,1313.05018536075,27.188444176574,1
172.750000000022,14,1313.68596897364,27.188444176574,1
172.775000000022,12,1314.27774193167,27.188444176574,1
172.800000000022,13,1314.85833717531,27.188444176574,1
172.825000000022,11,1315.42682088472,27.188444176574,1

172.850000000022,11,1315.97157650653,27.188444176574,1
172.875000000022,10,1316.50365637028,27.188444176574,1
172.900000000022,9,1317.00973342312,27.188444176574,1
172.925000000022,8,1317.48839300074,0,0
172.950000000022,8,1317.95296215598,0,0
172.975000000022,7,1318.40252906002,0,0
173.000000000022,7,1318.83709371286,0,0
173.025000000022,7,1319.27165836571,0,0
173.050000000022,6,1319.69010503725,0,0
173.075000000022,5,1320.07490646503,0,0
173.100000000022,4,1320.42279354768,0,0
173.125000000022,4,1320.75129354768,0,0
173.150000000022,5,1321.09918063034,0,0
173.175000000022,6,1321.48398205811,0,0
173.200000000022,7,1321.90242872966,0,0
173.225000000022,7,1322.3369933825,0,0
173.250000000022,7,1322.77155803535,0,0
173.275000000022,7,1323.20612268819,0,0
173.300000000022,7,1323.64068734103,0,0
173.325000000022,7,1324.07525199387,0,0
173.350000000022,8,1324.52481889791,0,0
173.375000000022,8,1324.98938805315,0,0
173.400000000022,7,1325.43895495719,0,0
173.425000000022,8,1325.88852186124,0,0
173.450000000022,8,1326.35309101648,0,0
173.475000000022,8,1326.81766017171,0,0
173.500000000022,9,1327.29631974933,0,0
173.525000000022,9,1327.78906974933,0,0
173.550000000022,9,1328.28181974933,0,0
173.575000000022,9,1328.77456974933,0,0
173.600000000022,9,1329.26731974933,0,0
173.625000000022,9,1329.76006974933,0,0
173.650000000022,8,1330.23872932695,0,0
173.675000000022,8,1330.70329848219,0,0
173.700000000022,9,1331.18195805981,0,0
173.725000000022,9,1331.67470805981,0,0
173.750000000022,9,1332.16745805981,0,0
173.775000000022,9,1332.66020805981,0,0
173.800000000022,8,1333.13886763743,0,0
173.825000000022,9,1333.61752721505,0,0
173.850000000022,8,1334.09618679267,0,0
173.875000000022,7,1334.54575369671,0,0
173.900000000022,7,1334.98031834955,0,0
173.925000000022,8,1335.4298852536,0,0
173.950000000022,8,1335.89445440884,0,0
173.975000000022,8,1336.35902356408,0,0
174.000000000022,7,1336.80859046812,0,0
174.025000000022,7,1337.24315512096,0,0
174.050000000022,7,1337.6777197738,0,0
174.075000000022,8,1338.12728667784,0,0
174.100000000022,9,1338.60594625546,0,0
174.125000000022,9,1339.09869625546,0,0

174.150000000022,9,1339.59144625546,0,0
174.175000000022,9,1340.08419625546,0,0
174.200000000022,8,1340.56285583308,0,0
174.225000000022,8,1341.02742498832,0,0
174.250000000022,8,1341.49199414356,0,0
174.275000000022,8,1341.9565632988,0,0
174.300000000022,9,1342.43522287642,0,0
174.325000000023,8,1342.91388245404,0,0
174.350000000023,8,1343.37845160928,0,0
174.375000000023,8,1343.84302076452,0,0
174.400000000023,9,1344.32168034214,0,0
174.425000000023,7,1344.78533766856,0,0
174.450000000023,10,1345.26232204782,0,1
174.475000000023,9,1345.76839910066,0,0
174.500000000023,8,1346.24705867828,0,0
174.525000000023,8,1346.71162783352,0,0
174.550000000023,8,1347.17619698876,0,0
174.575000000023,7,1347.6257638928,0,0
174.600000000023,6,1348.04421056435,0,0
174.625000000023,6,1348.4465392546,0,0
174.650000000023,6,1348.84886794485,0,0
174.675000000023,6,1349.25119663511,0,0
174.700000000023,6,1349.65352532536,0,0
174.725000000023,6,1350.05585401561,0,0
174.750000000023,7,1350.47430068716,0,0
174.775000000023,7,1350.90886534,0,0
174.800000000023,8,1351.35843224404,0,0
174.825000000023,8,1351.82300139928,0,0
174.850000000023,7,1352.27256830332,0,0
174.875000000023,6,1352.69101497487,0,0
174.900000000023,6,1353.09334366512,0,0
174.925000000023,6,1353.49567235537,0,0
174.950000000023,6,1353.89800104562,0,0
174.975000000023,7,1354.31644771717,0,0
175.000000000023,7,1354.75101237001,0,0
175.025000000023,7,1355.18557702286,0,0
175.050000000023,7,1355.6201416757,0,0
175.075000000023,7,1356.05470632854,0,0
175.100000000023,7,1356.48927098138,0,0
175.125000000023,7,1356.92383563422,0,0
175.150000000023,6,1357.34228230577,0,0
175.175000000023,6,1357.74461099602,0,0
175.200000000023,5,1358.1294124238,0,0
175.225000000023,5,1358.49668658911,0,0
175.250000000023,5,1358.86396075441,0,0
175.275000000023,5,1359.23123491971,0,0
175.300000000023,5,1359.59850908502,0,0
175.325000000023,5,1359.96578325032,0,0
175.350000000023,5,1360.33305741563,0,0
175.375000000023,8,1360.7489790759,0,0
175.400000000023,5,1361.16490073617,0,0
175.425000000023,1,1361.43066281882,0,0

175.450000000023,8,1361.74507239644,0,0
175.475000000023,1,1362.05948197406,0,0
175.500000000023,7,1362.35888930048,0,0
175.525000000023,2,1362.69231391572,0,0
175.550000000023,3,1362.9507008771,0,0
175.575000000023,4,1363.25719554967,0,0
175.600000000023,3,1363.56369022224,0,0
175.625000000023,6,1363.90709923994,0,0
175.650000000023,12,1364.39275293021,21.6894138820351,1
175.675000000023,14,1364.98452588824,21.6894138820351,1
175.700000000023,11,1365.56418731204,21.6894138820351,1
175.725000000023,9,1366.08294012294,21.6894138820351,1
175.750000000023,15,1366.64738388025,21.6894138820351,1
175.775000000023,9,1367.21182763756,21.6894138820351,1
175.800000000023,10,1367.7179046904,21.6894138820351,1
175.825000000023,11,1368.24998455415,21.6894138820351,1
175.850000000023,11,1368.79474017596,21.6894138820351,1
175.875000000023,10,1369.32682003971,21.6894138820351,1
175.900000000023,10,1369.8462241454,21.6894138820351,1
175.925000000023,11,1370.37830400915,21.6894138820351,1
175.950000000023,11,1370.92305963096,21.6894138820351,1
175.975000000023,9,1371.44181244187,21.6894138820351,1
176.000000000023,9,1371.93456244187,0,0
176.025000000023,9,1372.42731244187,0,0
176.050000000023,9,1372.92006244187,0,0
176.075000000023,8,1373.39872201949,0,0
176.100000000023,8,1373.86329117473,0,0
176.125000000023,8,1374.32786032997,0,0
176.150000000023,8,1374.79242948521,0,0
176.175000000023,7,1375.24199638925,0,0
176.200000000023,7,1375.67656104209,0,0
176.225000000023,7,1376.11112569493,0,0
176.250000000023,7,1376.54569034778,0,0
176.275000000023,6,1376.96413701932,0,0
176.300000000023,9,1377.41167636445,0,0
176.325000000023,4,1377.82230136445,0,0
176.350000000023,3,1378.12879603702,0,0
176.375000000023,2,1378.3871829984,0,0
176.400000000023,1,1378.58545028721,0,0
176.425000000023,0,1378.66757528721,0,0
176.450000000023,0,1378.66757528721,0,0
176.475000000023,0,1378.66757528721,0,0
176.500000000023,0,1378.66757528721,0,0
176.525000000023,0,1378.66757528721,0,0
176.550000000023,0,1378.66757528721,0,0
176.575000000023,0,1378.66757528721,0,0
176.600000000023,0,1378.66757528721,0,0
176.625000000023,0,1378.66757528721,0,0
176.650000000023,0,1378.66757528721,0,0
176.675000000023,0,1378.66757528721,0,0
176.700000000023,0,1378.66757528721,0,0
176.725000000023,0,1378.66757528721,0,0

176.750000000023,0,1378.66757528721,0,0
176.775000000023,0,1378.66757528721,0,0
176.800000000023,0,1378.66757528721,0,0
176.825000000023,0,1378.66757528721,0,0
176.850000000023,0,1378.66757528721,0,0
176.875000000023,0,1378.66757528721,0,0
176.900000000023,0,1378.66757528721,0,0
176.925000000023,0,1378.66757528721,0,0
176.950000000023,0,1378.66757528721,0,0
176.975000000023,0,1378.66757528721,0,0
177.000000000023,0,1378.66757528721,0,0
177.025000000023,0,1378.66757528721,0,0
177.050000000023,0,1378.66757528721,0,0
177.075000000023,0,1378.66757528721,0,0
177.100000000023,0,1378.66757528721,0,0
177.125000000023,0,1378.66757528721,0,0
177.150000000023,0,1378.66757528721,0,0
177.175000000023,0,1378.66757528721,0,0
177.200000000023,0,1378.66757528721,0,0
177.225000000023,0,1378.66757528721,0,0
177.250000000023,0,1378.66757528721,0,0
177.275000000023,0,1378.66757528721,0,0
177.300000000023,0,1378.66757528721,0,0
177.325000000023,0,1378.66757528721,0,0
177.350000000023,0,1378.66757528721,0,0
177.375000000023,0,1378.66757528721,0,0
177.400000000023,0,1378.66757528721,0,0
177.425000000023,0,1378.66757528721,0,0
177.450000000023,0,1378.66757528721,0,0
177.475000000023,0,1378.66757528721,0,0
177.500000000023,0,1378.66757528721,0,0
177.525000000023,0,1378.66757528721,0,0
177.550000000023,0,1378.66757528721,0,0
177.575000000023,0,1378.66757528721,0,0
177.600000000023,0,1378.66757528721,0,0
177.625000000023,0,1378.66757528721,0,0
177.650000000023,0,1378.66757528721,0,0
177.675000000023,0,1378.66757528721,0,0
177.700000000023,0,1378.66757528721,0,0
177.725000000023,0,1378.66757528721,0,0
177.750000000023,0,1378.66757528721,0,0
177.775000000023,0,1378.66757528721,0,0
177.800000000023,0,1378.66757528721,0,0
177.825000000023,0,1378.66757528721,0,0
177.850000000023,0,1378.66757528721,0,0
177.875000000023,0,1378.66757528721,0,0
177.900000000023,0,1378.66757528721,0,0
177.925000000023,0,1378.66757528721,0,0
177.950000000023,0,1378.66757528721,0,0
177.975000000023,0,1378.66757528721,0,0
178.000000000023,0,1378.66757528721,0,0
178.025000000023,0,1378.66757528721,0,0

178.050000000023,0,1378.66757528721,0,0
178.075000000023,0,1378.66757528721,0,0
178.100000000023,0,1378.66757528721,0,0
178.125000000023,0,1378.66757528721,0,0
178.150000000023,0,1378.66757528721,0,0
178.175000000023,2,1378.78371757602,0,0
178.200000000023,0,1378.89985986483,0,0
178.225000000023,0,1378.89985986483,0,0
178.250000000023,0,1378.89985986483,0,0
178.275000000023,0,1378.89985986483,0,0
178.300000000023,0,1378.89985986483,0,0
178.325000000023,0,1378.89985986483,0,0
178.350000000023,0,1378.89985986483,0,0
178.375000000023,0,1378.89985986483,0,0
178.400000000023,0,1378.89985986483,0,0
178.425000000023,0,1378.89985986483,0,0
178.450000000023,0,1378.89985986483,0,0
178.475000000023,0,1378.89985986483,0,0
178.500000000023,0,1378.89985986483,0,0
178.525000000023,0,1378.89985986483,0,0
178.550000000023,0,1378.89985986483,0,0
178.575000000023,0,1378.89985986483,0,0
178.600000000023,0,1378.89985986483,0,0
178.625000000023,0,1378.89985986483,0,0
178.650000000023,0,1378.89985986483,0,0
178.675000000023,0,1378.89985986483,0,0
178.700000000023,0,1378.89985986483,0,0
178.725000000024,0,1378.89985986483,0,0
178.750000000024,0,1378.89985986483,0,0
178.775000000024,0,1378.89985986483,0,0
178.800000000024,0,1378.89985986483,0,0
178.825000000024,0,1378.89985986483,0,0
178.850000000024,0,1378.89985986483,0,0
178.875000000024,0,1378.89985986483,0,0
178.900000000024,0,1378.89985986483,0,0
178.925000000024,0,1378.89985986483,0,0
178.950000000024,0,1378.89985986483,0,0
178.975000000024,0,1378.89985986483,0,0
179.000000000024,0,1378.89985986483,0,0
179.025000000024,0,1378.89985986483,0,0
179.050000000024,0,1378.89985986483,0,0
179.075000000024,0,1378.89985986483,0,0
179.100000000024,0,1378.89985986483,0,0
179.125000000024,0,1378.89985986483,0,0
179.150000000024,1,1378.98198486483,0,0
179.175000000024,0,1379.06410986483,0,0
179.200000000024,0,1379.06410986483,0,0
179.225000000024,0,1379.06410986483,0,0
179.250000000024,1,1379.14623486483,0,0
179.275000000024,0,1379.22835986483,0,0
179.300000000024,5,1379.41199694748,0,0
179.325000000024,3,1379.73787870271,0,0

179.350000000024,4,1380.04437337528,0,0
179.375000000024,1,1380.29074837528,0,0
179.400000000024,1,1380.45499837528,0,0
179.425000000024,5,1380.72076045793,0,0
179.450000000024,0,1380.90439754058,0,0
179.475000000024,0,1380.90439754058,0,0
179.500000000024,5,1381.08803462324,0,0
179.525000000024,0,1381.27167170589,0,0
179.550000000024,0,1381.27167170589,0,0
179.575000000024,0,1381.27167170589,0,0
179.600000000024,0,1381.27167170589,0,0
179.625000000024,0,1381.27167170589,0,0
179.650000000024,0,1381.27167170589,0,0
179.675000000024,0,1381.27167170589,0,0
179.700000000024,0,1381.27167170589,0,0
179.725000000024,0,1381.27167170589,0,0
179.750000000024,0,1381.27167170589,0,0
179.775000000024,0,1381.27167170589,0,0
179.800000000024,0,1381.27167170589,0,0
179.825000000024,0,1381.27167170589,0,0
179.850000000024,0,1381.27167170589,0,0
179.875000000024,0,1381.27167170589,0,0
179.900000000024,0,1381.27167170589,0,0
179.925000000024,0,1381.27167170589,0,0
179.950000000024,0,1381.27167170589,0,0
179.975000000024,0,1381.27167170589,0,0
180.000000000024,0,1381.27167170589,0,0
180.025000000024,0,1381.27167170589,0,0
180.050000000024,0,1381.27167170589,0,0
180.075000000024,0,1381.27167170589,0,0
180.100000000024,0,1381.27167170589,0,0
180.125000000024,0,1381.27167170589,0,0
180.150000000024,0,1381.27167170589,0,0
180.175000000024,0,1381.27167170589,0,0
180.200000000024,0,1381.27167170589,0,0
180.225000000024,0,1381.27167170589,0,0
180.250000000024,0,1381.27167170589,0,0
180.275000000024,0,1381.27167170589,0,0
180.300000000024,0,1381.27167170589,0,0
180.325000000024,0,1381.27167170589,0,0
180.350000000024,1,1381.35379670589,0,0
180.375000000024,1,1381.51804670589,0,0
180.400000000024,1,1381.68229670589,0,0
180.425000000024,0,1381.76442170589,0,0
180.450000000024,1,1381.84654670589,0,0
180.475000000024,0,1381.92867170589,0,0
180.500000000024,0,1381.92867170589,0,0
180.525000000024,0,1381.92867170589,0,0
180.550000000024,1,1382.01079670589,0,0
180.575000000024,0,1382.09292170589,0,0
180.600000000024,0,1382.09292170589,0,0
180.625000000024,0,1382.09292170589,0,0

180.650000000024,0,1382.09292170589,0,0
180.675000000024,0,1382.09292170589,0,0
180.700000000024,0,1382.09292170589,0,0
180.725000000024,0,1382.09292170589,0,0
180.750000000024,0,1382.09292170589,0,0
180.775000000024,0,1382.09292170589,0,0
180.800000000024,0,1382.09292170589,0,0
180.825000000024,0,1382.09292170589,0,0
180.850000000024,0,1382.09292170589,0,0
180.875000000024,0,1382.09292170589,0,0
180.900000000024,0,1382.09292170589,0,0
180.925000000024,0,1382.09292170589,0,0
180.950000000024,0,1382.09292170589,0,0
180.975000000024,0,1382.09292170589,0,0
181.000000000024,0,1382.09292170589,0,0
181.025000000024,0,1382.09292170589,0,0
181.050000000024,0,1382.09292170589,0,0
181.075000000024,0,1382.09292170589,0,0
181.100000000024,0,1382.09292170589,0,0
181.125000000024,0,1382.09292170589,0,0
181.150000000024,0,1382.09292170589,0,0
181.175000000024,0,1382.09292170589,0,0
181.200000000024,0,1382.09292170589,0,0
181.225000000024,0,1382.09292170589,0,0
181.250000000024,0,1382.09292170589,0,0
181.275000000024,0,1382.09292170589,0,0
181.300000000024,0,1382.09292170589,0,0
181.325000000024,0,1382.09292170589,0,0
181.350000000024,0,1382.09292170589,0,0
181.375000000024,0,1382.09292170589,0,0
181.400000000024,0,1382.09292170589,0,0
181.425000000024,0,1382.09292170589,0,0
181.450000000024,0,1382.09292170589,0,0
181.475000000024,0,1382.09292170589,0,0
181.500000000024,0,1382.09292170589,0,0
181.525000000024,0,1382.09292170589,0,0
181.550000000024,0,1382.09292170589,0,0
181.575000000024,0,1382.09292170589,0,0
181.600000000024,0,1382.09292170589,0,0
181.625000000024,0,1382.09292170589,0,0
181.650000000024,0,1382.09292170589,0,0
181.675000000024,0,1382.09292170589,0,0
181.700000000024,0,1382.09292170589,0,0
181.725000000024,0,1382.09292170589,0,0
181.750000000024,0,1382.09292170589,0,0
181.775000000024,0,1382.09292170589,0,0
181.800000000024,0,1382.09292170589,0,0
181.825000000024,0,1382.09292170589,0,0
181.850000000024,0,1382.09292170589,0,0
181.875000000024,0,1382.09292170589,0,0
181.900000000024,0,1382.09292170589,0,0
181.925000000024,0,1382.09292170589,0,0

```
181.950000000024,0,1382.09292170589,0,0
181.975000000024,0,1382.09292170589,0,0
182.000000000024,0,1382.09292170589,0,0
182.025000000024,0,1382.09292170589,0,0
182.050000000024,0,1382.09292170589,0,0
182.075000000024,0,1382.09292170589,0,0
182.100000000024,0,1382.09292170589,0,0
182.125000000024,0,1382.09292170589,0,0
182.150000000024,0,1382.09292170589,0,0
182.175000000024,0,1382.09292170589,0,0
182.200000000024,0,1382.09292170589,0,0
182.225000000024,0,1382.09292170589,0,0
182.250000000024,0,1382.09292170589,0,0
182.275000000024,0,1382.09292170589,0,0
182.300000000024,0,1382.09292170589,0,0
182.325000000024,0,1382.09292170589,0,0
182.350000000024,0,1382.09292170589,0,0
182.375000000024,0,1382.09292170589,0,0
182.400000000024,0,1382.09292170589,0,0
182.425000000024,0,1382.09292170589,0,0
182.450000000024,0,1382.09292170589,0,0
182.475000000024,3,1382.23516637846,0,0
182.500000000024,0,1382.37741105103,0,0
182.525000000024,1,1382.45953605103,0,0
182.550000000024,0,1382.54166105103,0,0
182.575000000024,0,1382.54166105103,0,0
182.600000000024,0,1382.54166105103,0,0
182.625000000024,4,1382.70591105103,0,0
182.650000000024,0,1382.87016105103,0,0
182.675000000024,1,1382.95228605103,0,0
182.700000000024,0,1383.03441105103,0,0
182.725000000024,1,1383.11653605103,0,0
182.750000000024,0,1383.19866105103,0,0
182.775000000024,0,1383.19866105103,0,0
182.800000000024,0,1383.19866105103,0,0
182.825000000024,0,1383.19866105103,0,0
182.850000000024,0,1383.19866105103,0,0
182.875000000024,4,1383.36291105103,0,0
182.900000000024,0,1383.52716105103,0,0
182.925000000024,0,1383.52716105103,0,0
182.950000000024,0,1383.52716105103,0,0
182.975000000024,0,1383.52716105103,0,0
183.000000000024,2,1383.64330333984,0,0
183.025000000024,0,1383.75944562865,0,0
183.050000000024,0,1383.75944562865,0,0
183.075000000024,0,1383.75944562865,0,0
183.100000000024,0,1383.75944562865,0,0
183.125000000025,0,1383.75944562865,0,0
183.150000000025,0,1383.75944562865,0,0
183.175000000025,0,1383.75944562865,0,0
183.200000000025,0,1383.75944562865,0,0
183.225000000025,0,1383.75944562865,0,0
```

183.250000000025,0,1383.75944562865,0,0
183.275000000025,0,1383.75944562865,0,0
183.300000000025,0,1383.75944562865,0,0
183.325000000025,0,1383.75944562865,0,0
183.350000000025,1,1383.84157062865,0,0
183.375000000025,0,1383.92369562865,0,0
183.400000000025,0,1383.92369562865,0,0
183.425000000025,0,1383.92369562865,0,0
183.450000000025,0,1383.92369562865,0,0
183.475000000025,0,1383.92369562865,0,0
183.500000000025,0,1383.92369562865,0,0
183.525000000025,0,1383.92369562865,0,0
183.550000000025,0,1383.92369562865,0,0
183.575000000025,0,1383.92369562865,0,0
183.600000000025,0,1383.92369562865,0,0
183.625000000025,0,1383.92369562865,0,0
183.650000000025,0,1383.92369562865,0,0
183.675000000025,0,1383.92369562865,0,0
183.700000000025,0,1383.92369562865,0,0
183.725000000025,0,1383.92369562865,0,0
183.750000000025,0,1383.92369562865,0,0
183.775000000025,0,1383.92369562865,0,0
183.800000000025,0,1383.92369562865,0,0
183.825000000025,0,1383.92369562865,0,0
183.850000000025,0,1383.92369562865,0,0
183.875000000025,0,1383.92369562865,0,0
183.900000000025,0,1383.92369562865,0,0
183.925000000025,0,1383.92369562865,0,0
183.950000000025,0,1383.92369562865,0,0
183.975000000025,0,1383.92369562865,0,0
184.000000000025,0,1383.92369562865,0,0
184.025000000025,0,1383.92369562865,0,0
184.050000000025,0,1383.92369562865,0,0
184.075000000025,0,1383.92369562865,0,0
184.100000000025,0,1383.92369562865,0,0
184.125000000025,0,1383.92369562865,0,0
184.150000000025,0,1383.92369562865,0,0
184.175000000025,0,1383.92369562865,0,0
184.200000000025,0,1383.92369562865,0,0
184.225000000025,0,1383.92369562865,0,0
184.250000000025,0,1383.92369562865,0,0
184.275000000025,1,1384.00582062865,0,0
184.300000000025,0,1384.08794562865,0,0
184.325000000025,0,1384.08794562865,0,0
184.350000000025,0,1384.08794562865,0,0
184.375000000025,0,1384.08794562865,0,0
184.400000000025,0,1384.08794562865,0,0
184.425000000025,0,1384.08794562865,0,0
184.450000000025,0,1384.08794562865,0,0
184.475000000025,0,1384.08794562865,0,0
184.500000000025,0,1384.08794562865,0,0
184.525000000025,0,1384.08794562865,0,0

184.550000000025,0,1384.08794562865,0,0
184.575000000025,0,1384.08794562865,0,0
184.600000000025,0,1384.08794562865,0,0
184.625000000025,0,1384.08794562865,0,0
184.650000000025,0,1384.08794562865,0,0
184.675000000025,0,1384.08794562865,0,0
184.700000000025,0,1384.08794562865,0,0
184.725000000025,0,1384.08794562865,0,0
184.750000000025,0,1384.08794562865,0,0
184.775000000025,0,1384.08794562865,0,0
184.800000000025,0,1384.08794562865,0,0
184.825000000025,0,1384.08794562865,0,0
184.850000000025,0,1384.08794562865,0,0
184.875000000025,0,1384.08794562865,0,0
184.900000000025,0,1384.08794562865,0,0
184.925000000025,0,1384.08794562865,0,0
184.950000000025,0,1384.08794562865,0,0
184.975000000025,0,1384.08794562865,0,0
185.000000000025,0,1384.08794562865,0,0
185.025000000025,0,1384.08794562865,0,0
185.050000000025,0,1384.08794562865,0,0
185.075000000025,0,1384.08794562865,0,0
185.100000000025,0,1384.08794562865,0,0
185.125000000025,0,1384.08794562865,0,0
185.150000000025,0,1384.08794562865,0,0
185.175000000025,0,1384.08794562865,0,0
185.200000000025,0,1384.08794562865,0,0
185.225000000025,0,1384.08794562865,0,0
185.250000000025,0,1384.08794562865,0,0
185.275000000025,0,1384.08794562865,0,0
185.300000000025,0,1384.08794562865,0,0
185.325000000025,0,1384.08794562865,0,0
185.350000000025,0,1384.08794562865,0,0
185.375000000025,0,1384.08794562865,0,0
185.400000000025,0,1384.08794562865,0,0
185.425000000025,0,1384.08794562865,0,0
185.450000000025,0,1384.08794562865,0,0
185.475000000025,0,1384.08794562865,0,0
185.500000000025,0,1384.08794562865,0,0
185.525000000025,0,1384.08794562865,0,0
185.550000000025,0,1384.08794562865,0,0
185.575000000025,0,1384.08794562865,0,0
185.600000000025,0,1384.08794562865,0,0
185.625000000025,0,1384.08794562865,0,0
185.650000000025,0,1384.08794562865,0,0
185.675000000025,0,1384.08794562865,0,0
185.700000000025,0,1384.08794562865,0,0
185.725000000025,0,1384.08794562865,0,0
185.750000000025,6,1384.28910997378,0,0
185.775000000025,0,1384.4902743189,0,0
185.800000000025,0,1384.4902743189,0,0
185.825000000025,0,1384.4902743189,0,0

185.850000000025,1,1384.5723993189,0,0
185.875000000025,0,1384.6545243189,0,0
185.900000000025,0,1384.6545243189,0,0
185.925000000025,0,1384.6545243189,0,0
185.950000000025,0,1384.6545243189,0,0
185.975000000025,0,1384.6545243189,0,0
186.000000000025,0,1384.6545243189,0,0
186.025000000025,0,1384.6545243189,0,0
186.050000000025,4,1384.8187743189,0,0
186.075000000025,1,1385.0651493189,0,0
186.100000000025,1,1385.2293993189,0,0
186.125000000025,0,1385.3115243189,0,0
186.150000000025,0,1385.3115243189,0,0
186.175000000025,4,1385.4757743189,0,0
186.200000000025,0,1385.6400243189,0,0
186.225000000025,0,1385.6400243189,0,0
186.250000000025,0,1385.6400243189,0,0
186.275000000025,0,1385.6400243189,0,0
186.300000000025,0,1385.6400243189,0,0
186.325000000025,0,1385.6400243189,0,0
186.350000000025,0,1385.6400243189,0,0
186.375000000025,0,1385.6400243189,0,0
186.400000000025,0,1385.6400243189,0,0
186.425000000025,0,1385.6400243189,0,0
186.450000000025,0,1385.6400243189,0,0
186.475000000025,0,1385.6400243189,0,0
186.500000000025,0,1385.6400243189,0,0
186.525000000025,0,1385.6400243189,0,0
186.550000000025,0,1385.6400243189,0,0
186.575000000025,0,1385.6400243189,0,0
186.600000000025,0,1385.6400243189,0,0
186.625000000025,0,1385.6400243189,0,0
186.650000000025,0,1385.6400243189,0,0
186.675000000025,0,1385.6400243189,0,0
186.700000000025,0,1385.6400243189,0,0
186.725000000025,0,1385.6400243189,0,0
186.750000000025,0,1385.6400243189,0,0
186.775000000025,0,1385.6400243189,0,0
186.800000000025,0,1385.6400243189,0,0
186.825000000025,1,1385.7221493189,0,0
186.850000000025,0,1385.8042743189,0,0
186.875000000025,0,1385.8042743189,0,0
186.900000000025,0,1385.8042743189,0,0
186.925000000025,0,1385.8042743189,0,0
186.950000000025,0,1385.8042743189,0,0
186.975000000025,0,1385.8042743189,0,0
187.000000000025,0,1385.8042743189,0,0
187.025000000025,0,1385.8042743189,0,0
187.050000000025,0,1385.8042743189,0,0
187.075000000025,0,1385.8042743189,0,0
187.100000000025,0,1385.8042743189,0,0
187.125000000025,0,1385.8042743189,0,0

187.150000000025,0,1385.8042743189,0,0
187.175000000025,0,1385.8042743189,0,0
187.200000000025,0,1385.8042743189,0,0
187.225000000025,0,1385.8042743189,0,0
187.250000000025,0,1385.8042743189,0,0
187.275000000025,0,1385.8042743189,0,0
187.300000000025,0,1385.8042743189,0,0
187.325000000025,0,1385.8042743189,0,0
187.350000000025,0,1385.8042743189,0,0
187.375000000025,0,1385.8042743189,0,0
187.400000000025,0,1385.8042743189,0,0
187.425000000025,0,1385.8042743189,0,0
187.450000000025,0,1385.8042743189,0,0
187.475000000025,0,1385.8042743189,0,0
187.500000000025,0,1385.8042743189,0,0
187.525000000026,0,1385.8042743189,0,0
187.550000000026,0,1385.8042743189,0,0
187.575000000026,0,1385.8042743189,0,0
187.600000000026,0,1385.8042743189,0,0
187.625000000026,0,1385.8042743189,0,0
187.650000000026,0,1385.8042743189,0,0
187.675000000026,0,1385.8042743189,0,0
187.700000000026,0,1385.8042743189,0,0
187.725000000026,0,1385.8042743189,0,0
187.750000000026,0,1385.8042743189,0,0
187.775000000026,0,1385.8042743189,0,0
187.800000000026,0,1385.8042743189,0,0
187.825000000026,0,1385.8042743189,0,0
187.850000000026,0,1385.8042743189,0,0
187.875000000026,1,1385.8863993189,0,0
187.900000000026,0,1385.9685243189,0,0
187.925000000026,0,1385.9685243189,0,0
187.950000000026,0,1385.9685243189,0,0
187.975000000026,0,1385.9685243189,0,0
188.000000000026,0,1385.9685243189,0,0
188.025000000026,0,1385.9685243189,0,0
188.050000000026,0,1385.9685243189,0,0
188.075000000026,0,1385.9685243189,0,0
188.100000000026,0,1385.9685243189,0,0
188.125000000026,0,1385.9685243189,0,0
188.150000000026,0,1385.9685243189,0,0
188.175000000026,0,1385.9685243189,0,0
188.200000000026,0,1385.9685243189,0,0
188.225000000026,0,1385.9685243189,0,0
188.250000000026,0,1385.9685243189,0,0
188.275000000026,0,1385.9685243189,0,0
188.300000000026,0,1385.9685243189,0,0
188.325000000026,0,1385.9685243189,0,0
188.350000000026,0,1385.9685243189,0,0
188.375000000026,0,1385.9685243189,0,0
188.400000000026,0,1385.9685243189,0,0
188.425000000026,0,1385.9685243189,0,0

188.450000000026,0,1385.9685243189,0,0
188.475000000026,0,1385.9685243189,0,0
188.500000000026,0,1385.9685243189,0,0
188.525000000026,0,1385.9685243189,0,0
188.550000000026,0,1385.9685243189,0,0
188.575000000026,0,1385.9685243189,0,0
188.600000000026,0,1385.9685243189,0,0
188.625000000026,0,1385.9685243189,0,0
188.650000000026,0,1385.9685243189,0,0
188.675000000026,0,1385.9685243189,0,0
188.700000000026,0,1385.9685243189,0,0
188.725000000026,0,1385.9685243189,0,0
188.750000000026,0,1385.9685243189,0,0
188.775000000026,0,1385.9685243189,0,0
188.800000000026,1,1386.0506493189,0,0
188.825000000026,0,1386.1327743189,0,0
188.850000000026,0,1386.1327743189,0,0
188.875000000026,0,1386.1327743189,0,0
188.900000000026,0,1386.1327743189,0,0
188.925000000026,0,1386.1327743189,0,0
188.950000000026,0,1386.1327743189,0,0
188.975000000026,1,1386.2148993189,0,0
189.000000000026,0,1386.2970243189,0,0
189.025000000026,0,1386.2970243189,0,0
189.050000000026,0,1386.2970243189,0,0
189.075000000026,0,1386.2970243189,0,0
189.100000000026,0,1386.2970243189,0,0
189.125000000026,0,1386.2970243189,0,0
189.150000000026,0,1386.2970243189,0,0
189.175000000026,0,1386.2970243189,0,0
189.200000000026,0,1386.2970243189,0,0
189.225000000026,0,1386.2970243189,0,0
189.250000000026,0,1386.2970243189,0,0
189.275000000026,0,1386.2970243189,0,0
189.300000000026,0,1386.2970243189,0,0
189.325000000026,0,1386.2970243189,0,0
189.350000000026,0,1386.2970243189,0,0
189.375000000026,0,1386.2970243189,0,0
189.400000000026,0,1386.2970243189,0,0
189.425000000026,1,1386.3791493189,0,0
189.450000000026,5,1386.64491140155,0,0
189.475000000026,0,1386.8285484842,0,0
189.500000000026,0,1386.8285484842,0,0
189.525000000026,5,1387.01218556686,0,0
189.550000000026,7,1387.41310497593,0,0
189.575000000026,1,1387.71251230235,0,0
189.600000000026,0,1387.79463730235,0,0
189.625000000026,0,1387.79463730235,0,0
189.650000000026,0,1387.79463730235,0,0
189.675000000026,1,1387.87676230235,0,0
189.700000000026,0,1387.95888730235,0,0
189.725000000026,0,1387.95888730235,0,0

189.750000000026,0,1387.95888730235,0,0
189.775000000026,0,1387.95888730235,0,0
189.800000000026,0,1387.95888730235,0,0
189.825000000026,0,1387.95888730235,0,0
189.850000000026,0,1387.95888730235,0,0
189.875000000026,0,1387.95888730235,0,0
189.900000000026,0,1387.95888730235,0,0
189.925000000026,0,1387.95888730235,0,0
189.950000000026,0,1387.95888730235,0,0
189.975000000026,0,1387.95888730235,0,0
190.000000000026,0,1387.95888730235,0,0
190.025000000026,0,1387.95888730235,0,0
190.050000000026,0,1387.95888730235,0,0
190.075000000026,0,1387.95888730235,0,0
190.100000000026,1,1388.04101230235,0,0
190.125000000026,0,1388.12313730235,0,0
190.150000000026,0,1388.12313730235,0,0
190.175000000026,0,1388.12313730235,0,0
190.200000000026,0,1388.12313730235,0,0
190.225000000026,0,1388.12313730235,0,0
190.250000000026,0,1388.12313730235,0,0
190.275000000026,0,1388.12313730235,0,0
190.300000000026,0,1388.12313730235,0,0
190.325000000026,0,1388.12313730235,0,0
190.350000000026,0,1388.12313730235,0,0
190.375000000026,0,1388.12313730235,0,0
190.400000000026,0,1388.12313730235,0,0
190.425000000026,0,1388.12313730235,0,0
190.450000000026,0,1388.12313730235,0,0
190.475000000026,0,1388.12313730235,0,0
190.500000000026,0,1388.12313730235,0,0
190.525000000026,0,1388.12313730235,0,0
190.550000000026,0,1388.12313730235,0,0
190.575000000026,0,1388.12313730235,0,0
190.600000000026,0,1388.12313730235,0,0
190.625000000026,0,1388.12313730235,0,0
190.650000000026,0,1388.12313730235,0,0
190.675000000026,0,1388.12313730235,0,0
190.700000000026,0,1388.12313730235,0,0
190.725000000026,0,1388.12313730235,0,0
190.750000000026,0,1388.12313730235,0,0
190.775000000026,0,1388.12313730235,0,0
190.800000000026,0,1388.12313730235,0,0
190.825000000026,0,1388.12313730235,0,0
190.850000000026,2,1388.23927959116,0,0
190.875000000026,0,1388.35542187997,0,0
190.900000000026,0,1388.35542187997,0,0
190.925000000026,0,1388.35542187997,0,0
190.950000000026,0,1388.35542187997,0,0
190.975000000026,0,1388.35542187997,0,0
191.000000000026,0,1388.35542187997,0,0
191.025000000026,0,1388.35542187997,0,0

191.050000000026,0,1388.35542187997,0,0
191.075000000026,0,1388.35542187997,0,0
191.100000000026,0,1388.35542187997,0,0
191.125000000026,0,1388.35542187997,0,0
191.150000000026,0,1388.35542187997,0,0
191.175000000026,0,1388.35542187997,0,0
191.200000000026,0,1388.35542187997,0,0
191.225000000026,0,1388.35542187997,0,0
191.250000000026,0,1388.35542187997,0,0
191.275000000026,0,1388.35542187997,0,0
191.300000000026,0,1388.35542187997,0,0
191.325000000026,0,1388.35542187997,0,0
191.350000000026,0,1388.35542187997,0,0
191.375000000026,0,1388.35542187997,0,0
191.400000000026,0,1388.35542187997,0,0
191.425000000026,0,1388.35542187997,0,0
191.450000000026,0,1388.35542187997,0,0
191.475000000026,0,1388.35542187997,0,0
191.500000000026,0,1388.35542187997,0,0
191.525000000026,0,1388.35542187997,0,0
191.550000000026,0,1388.35542187997,0,0
191.575000000026,0,1388.35542187997,0,0
191.600000000026,0,1388.35542187997,0,0
191.625000000026,0,1388.35542187997,0,0
191.650000000026,0,1388.35542187997,0,0
191.675000000026,0,1388.35542187997,0,0
191.700000000026,0,1388.35542187997,0,0
191.725000000026,0,1388.35542187997,0,0
191.750000000026,0,1388.35542187997,0,0
191.775000000026,0,1388.35542187997,0,0
191.800000000026,0,1388.35542187997,0,0
191.825000000026,0,1388.35542187997,0,0
191.850000000026,0,1388.35542187997,0,0
191.875000000026,0,1388.35542187997,0,0
191.900000000026,0,1388.35542187997,0,0
191.925000000027,0,1388.35542187997,0,0
191.950000000027,0,1388.35542187997,0,0
191.975000000027,0,1388.35542187997,0,0
192.000000000027,0,1388.35542187997,0,0
192.025000000027,0,1388.35542187997,0,0
192.050000000027,0,1388.35542187997,0,0
192.075000000027,0,1388.35542187997,0,0
192.100000000027,0,1388.35542187997,0,0
192.125000000027,0,1388.35542187997,0,0
192.150000000027,0,1388.35542187997,0,0
192.175000000027,0,1388.35542187997,0,0
192.200000000027,0,1388.35542187997,0,0
192.225000000027,0,1388.35542187997,0,0
192.250000000027,1,1388.43754687997,0,0
192.275000000027,0,1388.51967187997,0,0
192.300000000027,0,1388.51967187997,0,0
192.325000000027,0,1388.51967187997,0,0

192.350000000027,0,1388.51967187997,0,0
192.375000000027,0,1388.51967187997,0,0
192.400000000027,0,1388.51967187997,0,0
192.425000000027,0,1388.51967187997,0,0
192.450000000027,0,1388.51967187997,0,0
192.475000000027,0,1388.51967187997,0,0
192.500000000027,0,1388.51967187997,0,0
192.525000000027,0,1388.51967187997,0,0
192.550000000027,0,1388.51967187997,0,0
192.575000000027,0,1388.51967187997,0,0
192.600000000027,0,1388.51967187997,0,0
192.625000000027,0,1388.51967187997,0,0
192.650000000027,0,1388.51967187997,0,0
192.675000000027,0,1388.51967187997,0,0
192.700000000027,0,1388.51967187997,0,0
192.725000000027,0,1388.51967187997,0,0
192.750000000027,0,1388.51967187997,0,0
192.775000000027,0,1388.51967187997,0,0
192.800000000027,0,1388.51967187997,0,0
192.825000000027,0,1388.51967187997,0,0
192.850000000027,0,1388.51967187997,0,0
192.875000000027,0,1388.51967187997,0,0
192.900000000027,0,1388.51967187997,0,0
192.925000000027,0,1388.51967187997,0,0
192.950000000027,0,1388.51967187997,0,0
192.975000000027,0,1388.51967187997,0,0
193.000000000027,0,1388.51967187997,0,0
193.025000000027,1,1388.60179687997,0,0
193.050000000027,0,1388.68392187997,0,0
193.075000000027,0,1388.68392187997,0,0
193.100000000027,0,1388.68392187997,0,0
193.125000000027,0,1388.68392187997,0,0
193.150000000027,0,1388.68392187997,0,0
193.175000000027,3,1388.82616655254,0,0
193.200000000027,0,1388.96841122511,0,0
193.225000000027,0,1388.96841122511,0,0
193.250000000027,2,1389.08455351392,0,0
193.275000000027,0,1389.20069580273,0,0
193.300000000027,5,1389.38433288539,0,0
193.325000000027,0,1389.56796996804,0,0
193.350000000027,0,1389.56796996804,0,0
193.375000000027,6,1389.76913431316,0,0
193.400000000027,0,1389.97029865829,0,0
193.425000000027,0,1389.97029865829,0,0
193.450000000027,0,1389.97029865829,0,0
193.475000000027,2,1390.0864409471,0,0
193.500000000027,0,1390.20258323591,0,0
193.525000000027,0,1390.20258323591,0,0
193.550000000027,0,1390.20258323591,0,0
193.575000000027,0,1390.20258323591,0,0
193.600000000027,0,1390.20258323591,0,0
193.625000000027,1,1390.28470823591,0,0

193.650000000027,0,1390.36683323591,0,0
193.675000000027,0,1390.36683323591,0,0
193.700000000027,0,1390.36683323591,0,0
193.725000000027,0,1390.36683323591,0,0
193.750000000027,0,1390.36683323591,0,0
193.775000000027,0,1390.36683323591,0,0
193.800000000027,1,1390.44895823591,0,0
193.825000000027,0,1390.53108323591,0,0
193.850000000027,0,1390.53108323591,0,0
193.875000000027,0,1390.53108323591,0,0
193.900000000027,0,1390.53108323591,0,0
193.925000000027,0,1390.53108323591,0,0
193.950000000027,0,1390.53108323591,0,0
193.975000000027,0,1390.53108323591,0,0
194.000000000027,0,1390.53108323591,0,0
194.025000000027,0,1390.53108323591,0,0
194.050000000027,0,1390.53108323591,0,0
194.075000000027,0,1390.53108323591,0,0
194.100000000027,0,1390.53108323591,0,0
194.125000000027,0,1390.53108323591,0,0
194.150000000027,0,1390.53108323591,0,0
194.175000000027,0,1390.53108323591,0,0
194.200000000027,0,1390.53108323591,0,0
194.225000000027,0,1390.53108323591,0,0
194.250000000027,0,1390.53108323591,0,0
194.275000000027,0,1390.53108323591,0,0
194.300000000027,1,1390.61320823591,0,0
194.325000000027,0,1390.69533323591,0,0
194.350000000027,0,1390.69533323591,0,0
194.375000000027,0,1390.69533323591,0,0
194.400000000027,0,1390.69533323591,0,0
194.425000000027,0,1390.69533323591,0,0
194.450000000027,0,1390.69533323591,0,0
194.475000000027,0,1390.69533323591,0,0
194.500000000027,0,1390.69533323591,0,0
194.525000000027,0,1390.69533323591,0,0
194.550000000027,0,1390.69533323591,0,0
194.575000000027,0,1390.69533323591,0,0
194.600000000027,1,1390.77745823591,0,0
194.625000000027,0,1390.85958323591,0,0
194.650000000027,0,1390.85958323591,0,0
194.675000000027,0,1390.85958323591,0,0
194.700000000027,1,1390.94170823591,0,0
194.725000000027,0,1391.02383323591,0,0
194.750000000027,0,1391.02383323591,0,0
194.775000000027,0,1391.02383323591,0,0
194.800000000027,0,1391.02383323591,0,0
194.825000000027,0,1391.02383323591,0,0
194.850000000027,0,1391.02383323591,0,0
194.875000000027,1,1391.10595823591,0,0
194.900000000027,0,1391.18808323591,0,0
194.925000000027,0,1391.18808323591,0,0

194.950000000027,0,1391.18808323591,0,0
194.975000000027,0,1391.18808323591,0,0
195.000000000027,0,1391.18808323591,0,0
195.025000000027,0,1391.18808323591,0,0
195.050000000027,0,1391.18808323591,0,0
195.075000000027,0,1391.18808323591,0,0
195.100000000027,0,1391.18808323591,0,0
195.125000000027,0,1391.18808323591,0,0
195.150000000027,0,1391.18808323591,0,0
195.175000000027,0,1391.18808323591,0,0
195.200000000027,0,1391.18808323591,0,0
195.225000000027,0,1391.18808323591,0,0
195.250000000027,0,1391.18808323591,0,0
195.275000000027,0,1391.18808323591,0,0
195.300000000027,0,1391.18808323591,0,0
195.325000000027,0,1391.18808323591,0,0
195.350000000027,0,1391.18808323591,0,0
195.375000000027,0,1391.18808323591,0,0
195.400000000027,0,1391.18808323591,0,0
195.425000000027,0,1391.18808323591,0,0
195.450000000027,0,1391.18808323591,0,0
195.475000000027,0,1391.18808323591,0,0
195.500000000027,1,1391.27020823591,0,0
195.525000000027,0,1391.35233323591,0,0
195.550000000027,0,1391.35233323591,0,0
195.575000000027,0,1391.35233323591,0,0
195.600000000027,0,1391.35233323591,0,0
195.625000000027,0,1391.35233323591,0,0
195.650000000027,0,1391.35233323591,0,0
195.675000000027,0,1391.35233323591,0,0
195.700000000027,0,1391.35233323591,0,0
195.725000000027,1,1391.43445823591,0,0
195.750000000027,0,1391.51658323591,0,0
195.775000000027,0,1391.51658323591,0,0
195.800000000027,0,1391.51658323591,0,0
195.825000000027,0,1391.51658323591,0,0
195.850000000027,0,1391.51658323591,0,0
195.875000000027,0,1391.51658323591,0,0
195.900000000027,0,1391.51658323591,0,0
195.925000000027,0,1391.51658323591,0,0
195.950000000027,0,1391.51658323591,0,0
195.975000000027,0,1391.51658323591,0,0
196.000000000027,0,1391.51658323591,0,0
196.025000000027,0,1391.51658323591,0,0
196.050000000027,0,1391.51658323591,0,0
196.075000000027,0,1391.51658323591,0,0
196.100000000027,0,1391.51658323591,0,0
196.125000000027,0,1391.51658323591,0,0
196.150000000027,0,1391.51658323591,0,0
196.175000000027,0,1391.51658323591,0,0
196.200000000027,0,1391.51658323591,0,0
196.225000000027,0,1391.51658323591,0,0

196.250000000027,0,1391.51658323591,0,0
196.275000000027,0,1391.51658323591,0,0
196.300000000028,0,1391.51658323591,0,0
196.325000000028,0,1391.51658323591,0,0
196.350000000028,0,1391.51658323591,0,0
196.375000000028,0,1391.51658323591,0,0
196.400000000028,0,1391.51658323591,0,0
196.425000000028,0,1391.51658323591,0,0
196.450000000028,0,1391.51658323591,0,0
196.475000000028,0,1391.51658323591,0,0
196.500000000028,0,1391.51658323591,0,0
196.525000000028,0,1391.51658323591,0,0
196.550000000028,0,1391.51658323591,0,0
196.575000000028,0,1391.51658323591,0,0
196.600000000028,0,1391.51658323591,0,0
196.625000000028,0,1391.51658323591,0,0
196.650000000028,0,1391.51658323591,0,0
196.675000000028,0,1391.51658323591,0,0
196.700000000028,0,1391.51658323591,0,0
196.725000000028,0,1391.51658323591,0,0
196.750000000028,0,1391.51658323591,0,0
196.775000000028,0,1391.51658323591,0,0
196.800000000028,0,1391.51658323591,0,0
196.825000000028,0,1391.51658323591,0,0
196.850000000028,0,1391.51658323591,0,0
196.875000000028,0,1391.51658323591,0,0
196.900000000028,0,1391.51658323591,0,0
196.925000000028,0,1391.51658323591,0,0
196.950000000028,0,1391.51658323591,0,0
196.975000000028,0,1391.51658323591,0,0
197.000000000028,0,1391.51658323591,0,0
197.025000000028,3,1391.65882790848,0,0
197.050000000028,0,1391.80107258105,0,0
197.075000000028,4,1391.96532258105,0,0
197.100000000028,0,1392.12957258105,0,0
197.125000000028,0,1392.12957258105,0,0
197.150000000028,1,1392.21169758105,0,0
197.175000000028,0,1392.29382258105,0,0
197.200000000028,1,1392.37594758105,0,0
197.225000000028,1,1392.54019758105,0,0
197.250000000028,5,1392.8059596637,0,0
197.275000000028,1,1393.07172174636,0,0
197.300000000028,0,1393.15384674636,0,0
197.325000000028,0,1393.15384674636,0,0
197.350000000028,1,1393.23597174636,0,0
197.375000000028,0,1393.31809674636,0,0
197.400000000028,1,1393.40022174636,0,0
197.425000000028,0,1393.48234674636,0,0
197.450000000028,0,1393.48234674636,0,0
197.475000000028,0,1393.48234674636,0,0
197.500000000028,0,1393.48234674636,0,0
197.525000000028,0,1393.48234674636,0,0

197.550000000028,0,1393.48234674636,0,0
197.575000000028,0,1393.48234674636,0,0
197.600000000028,0,1393.48234674636,0,0
197.625000000028,0,1393.48234674636,0,0
197.650000000028,0,1393.48234674636,0,0
197.675000000028,0,1393.48234674636,0,0
197.700000000028,0,1393.48234674636,0,0
197.725000000028,0,1393.48234674636,0,0
197.750000000028,0,1393.48234674636,0,0
197.775000000028,1,1393.56447174636,0,0
197.800000000028,0,1393.64659674636,0,0
197.825000000028,0,1393.64659674636,0,0
197.850000000028,0,1393.64659674636,0,0
197.875000000028,0,1393.64659674636,0,0
197.900000000028,0,1393.64659674636,0,0
197.925000000028,0,1393.64659674636,0,0
197.950000000028,0,1393.64659674636,0,0
197.975000000028,0,1393.64659674636,0,0
198.000000000028,0,1393.64659674636,0,0
198.025000000028,0,1393.64659674636,0,0
198.050000000028,0,1393.64659674636,0,0
198.075000000028,0,1393.64659674636,0,0
198.100000000028,0,1393.64659674636,0,0
198.125000000028,0,1393.64659674636,0,0
198.150000000028,0,1393.64659674636,0,0
198.175000000028,0,1393.64659674636,0,0
198.200000000028,0,1393.64659674636,0,0
198.225000000028,0,1393.64659674636,0,0
198.250000000028,0,1393.64659674636,0,0
198.275000000028,0,1393.64659674636,0,0
198.300000000028,1,1393.72872174635,0,0
198.325000000028,0,1393.81084674635,0,0
198.350000000028,0,1393.81084674635,0,0
198.375000000028,0,1393.81084674635,0,0
198.400000000028,0,1393.81084674635,0,0
198.425000000028,0,1393.81084674635,0,0
198.450000000028,0,1393.81084674635,0,0
198.475000000028,0,1393.81084674635,0,0
198.500000000028,0,1393.81084674635,0,0
198.525000000028,0,1393.81084674635,0,0
198.550000000028,0,1393.81084674635,0,0
198.575000000028,0,1393.81084674635,0,0
198.600000000028,0,1393.81084674635,0,0
198.625000000028,13,1394.10695264485,25.8221033086748,1
198.650000000028,11,1394.67543635426,25.8221033086748,1
198.675000000028,11,1395.22019197607,25.8221033086748,1
198.700000000028,14,1395.79985339987,25.8221033086748,1
198.725000000028,20,1396.47441117806,25.8221033086748,1
198.750000000028,21,1397.21802937232,25.8221033086748,1
198.775000000028,23,1397.98823106512,25.8221033086748,1
198.800000000028,16,1398.71058872897,25.8221033086748,1
198.825000000028,8,1399.27137330659,25.8221033086748,1

198.850000000028,4,1399.66790788421,0,0
198.875000000028,3,1399.97440255678,0,0
198.900000000028,2,1400.23278951816,0,0
198.925000000028,2,1400.46507409578,0,0
198.950000000028,2,1400.6973586734,0,0
198.975000000028,3,1400.95574563478,0,0
199.000000000028,1,1401.18011530735,0,0
199.025000000028,1,1401.34436530735,0,0
199.050000000028,1,1401.50861530735,0,0
199.075000000028,1,1401.67286530735,0,0
199.100000000028,1,1401.83711530735,0,0
199.125000000028,2,1402.03538259616,0,0
199.150000000028,2,1402.26766717378,0,0
199.175000000028,3,1402.52605413517,0,0
199.200000000028,4,1402.83254880774,0,0
199.225000000028,8,1403.22908338536,0,0
199.250000000028,15,1403.77943672028,25.3852949064878,1
199.275000000028,24,1404.49983416784,25.3852949064878,1
199.300000000028,30,1405.35198000845,25.3852949064878,1
199.325000000028,29,1406.24405381858,25.3852949064878,1
199.350000000028,29,1407.12856713816,25.3852949064878,1
199.375000000028,26,1407.98958077549,25.3852949064878,1
199.400000000028,22,1408.79353814732,25.3852949064878,1
199.425000000028,16,1409.5072385416,25.3852949064878,1
199.450000000028,16,1410.1642385416,25.3852949064878,1
199.475000000028,14,1410.80002215449,25.3852949064878,1
199.500000000028,10,1411.36700782022,25.3852949064878,1
199.525000000028,10,1411.8864119259,25.3852949064878,1
199.550000000028,8,1412.37839855636,25.3852949064878,1
199.575000000028,8,1412.8429677116,25.3852949064878,1
199.600000000028,8,1413.30753686684,25.3852949064878,1
199.625000000028,9,1413.78619644446,25.3852949064878,1
199.650000000028,11,1414.30494925537,25.3852949064878,1
199.675000000028,13,1414.87343296477,25.3852949064878,1
199.700000000028,14,1415.47682247616,25.3852949064878,1
199.725000000028,15,1416.10217484636,25.3852949064878,1
199.750000000028,15,1416.73831236097,25.3852949064878,1
199.775000000028,14,1417.36366473117,25.3852949064878,1
199.800000000028,13,1417.96705424255,25.3852949064878,1
199.825000000028,14,1418.57044375394,25.3852949064878,1
199.850000000028,12,1419.16221671197,25.3852949064878,1
199.875000000028,11,1419.71908386802,25.3852949064878,1
199.900000000028,11,1420.26383948984,25.3852949064878,1
199.925000000028,12,1420.82070664589,25.3852949064878,1
199.950000000028,13,1421.40130188953,25.3852949064878,1
199.975000000028,13,1421.99351368653,25.3852949064878,1
200.000000000028,12,1422.57410893017,25.3852949064878,1
200.025000000028,11,1423.13097608622,25.3852949064878,1
200.050000000028,12,1423.68784324227,25.3852949064878,1
200.075000000028,12,1424.25682193256,25.3852949064878,1
200.100000000028,12,1424.82580062284,25.3852949064878,1
200.125000000028,12,1425.39477931313,25.3852949064878,1

200.150000000028,12,1425.96375800341,25.3852949064878,1
200.175000000028,12,1426.5327366937,25.3852949064878,1
200.200000000028,11,1427.08960384975,25.3852949064878,1
200.225000000028,11,1427.63435947157,25.3852949064878,1
200.250000000028,11,1428.17911509338,25.3852949064878,1
200.275000000028,12,1428.73598224944,25.3852949064878,1
200.300000000028,12,1429.30496093972,25.3852949064878,1
200.325000000028,13,1429.88555618336,25.3852949064878,1
200.350000000028,12,1430.466151427,25.3852949064878,1
200.375000000028,13,1431.04674667064,25.3852949064878,1
200.400000000028,12,1431.62734191428,25.3852949064878,1
200.425000000028,11,1432.18420907034,25.3852949064878,1
200.450000000028,10,1432.71628893408,25.3852949064878,1
200.475000000028,10,1433.23569303977,25.3852949064878,1
200.500000000028,9,1433.74177009261,25.3852949064878,1
200.525000000028,9,1434.23452009261,25.3852949064878,1
200.550000000028,9,1434.72727009261,25.3852949064878,1
200.575000000028,10,1435.23334714545,25.3852949064878,1
200.600000000028,11,1435.7654270092,25.3852949064878,1
200.625000000028,12,1436.32229416525,25.3852949064878,1
200.650000000028,15,1436.9248522677,25.3852949064878,1
200.675000000028,19,1437.60089560075,25.3852949064878,1
200.700000000029,20,1438.32614434179,25.3852949064878,1
200.725000000029,24,1439.09574719735,25.3852949064878,1
200.750000000029,27,1439.92480990531,25.3852949064878,1
200.775000000029,26,1440.77030090058,25.3852949064878,1
200.800000000029,23,1441.58291554199,25.3852949064878,1
200.825000000029,22,1442.36197360011,25.3852949064878,1
200.850000000029,22,1443.13237438866,25.3852949064878,1
200.875000000029,30,1443.96739193329,25.3852949064878,1
200.900000000029,25,1444.82783408364,25.3852949064878,1
200.925000000029,23,1445.63231674749,25.3852949064878,1
200.950000000029,24,1446.4285031016,25.3852949064878,1
200.975000000029,19,1447.18880636759,25.3852949064878,1
201.000000000029,23,1447.94063860718,25.3852949064878,1
201.025000000029,22,1448.71969666531,25.3852949064878,1
201.050000000029,22,1449.49009745386,25.3852949064878,1
201.075000000029,20,1450.24257201344,25.3852949064878,1
201.100000000029,18,1450.95827304517,25.3852949064878,1
201.125000000029,18,1451.65512677803,25.3852949064878,1
201.150000000029,16,1452.33205364446,25.3852949064878,1
201.175000000029,15,1452.97862240177,25.3852949064878,1
201.200000000029,14,1453.60397477196,25.3852949064878,1
201.225000000029,14,1454.21854199774,25.3852949064878,1
201.250000000029,13,1454.82193150913,25.3852949064878,1
201.275000000029,11,1455.39041521853,25.3852949064878,1
201.300000000029,11,1455.93517084035,25.3852949064878,1
201.325000000029,9,1456.45392365126,25.3852949064878,1
201.350000000029,8,1456.93258322888,0,0
201.375000000029,7,1457.38215013292,0,0
201.400000000029,6,1457.80059680447,0,0
201.425000000029,5,1458.18539823224,0,0

201.450000000029,5,1458.55267239755,0,0
201.475000000029,5,1458.91994656285,0,0
201.500000000029,4,1459.2678336455,0,0
201.525000000029,1,1459.5142086455,0,0
201.550000000029,0,1459.5963336455,0,0
201.575000000029,0,1459.5963336455,0,0
201.600000000029,1,1459.6784586455,0,0
201.625000000029,0,1459.7605836455,0,0
201.650000000029,0,1459.7605836455,0,0
201.675000000029,0,1459.7605836455,0,0
201.700000000029,0,1459.7605836455,0,0
201.725000000029,0,1459.7605836455,0,0
201.750000000029,0,1459.7605836455,0,0
201.775000000029,0,1459.7605836455,0,0
201.800000000029,0,1459.7605836455,0,0
201.825000000029,0,1459.7605836455,0,0
201.850000000029,0,1459.7605836455,0,0
201.875000000029,0,1459.7605836455,0,0
201.900000000029,0,1459.7605836455,0,0
201.925000000029,0,1459.7605836455,0,0
201.950000000029,0,1459.7605836455,0,0
201.975000000029,0,1459.7605836455,0,0
202.000000000029,0,1459.7605836455,0,0
202.025000000029,0,1459.7605836455,0,0
202.050000000029,0,1459.7605836455,0,0
202.075000000029,0,1459.7605836455,0,0
202.100000000029,0,1459.7605836455,0,0
202.125000000029,0,1459.7605836455,0,0
202.150000000029,0,1459.7605836455,0,0
202.175000000029,0,1459.7605836455,0,0
202.200000000029,0,1459.7605836455,0,0
202.225000000029,0,1459.7605836455,0,0
202.250000000029,0,1459.7605836455,0,0
202.275000000029,0,1459.7605836455,0,0
202.300000000029,0,1459.7605836455,0,0
202.325000000029,0,1459.7605836455,0,0
202.350000000029,0,1459.7605836455,0,0
202.375000000029,1,1459.8427086455,0,0
202.400000000029,0,1459.9248336455,0,0
202.425000000029,0,1459.9248336455,0,0
202.450000000029,0,1459.9248336455,0,0
202.475000000029,0,1459.9248336455,0,0
202.500000000029,0,1459.9248336455,0,0
202.525000000029,0,1459.9248336455,0,0
202.550000000029,1,1460.0069586455,0,0
202.575000000029,0,1460.0890836455,0,0
202.600000000029,0,1460.0890836455,0,0
202.625000000029,0,1460.0890836455,0,0
202.650000000029,0,1460.0890836455,0,0
202.675000000029,0,1460.0890836455,0,0
202.700000000029,0,1460.0890836455,0,0
202.725000000029,0,1460.0890836455,0,0

202.750000000029,0,1460.0890836455,0,0
202.775000000029,0,1460.0890836455,0,0
202.800000000029,0,1460.0890836455,0,0
202.825000000029,0,1460.0890836455,0,0
202.850000000029,1,1460.1712086455,0,0
202.875000000029,0,1460.2533336455,0,0
202.900000000029,0,1460.2533336455,0,0
202.925000000029,0,1460.2533336455,0,0
202.950000000029,0,1460.2533336455,0,0
202.975000000029,0,1460.2533336455,0,0
203.000000000029,0,1460.2533336455,0,0
203.025000000029,0,1460.2533336455,0,0
203.050000000029,0,1460.2533336455,0,0
203.075000000029,0,1460.2533336455,0,0
203.100000000029,0,1460.2533336455,0,0
203.125000000029,0,1460.2533336455,0,0
203.150000000029,0,1460.2533336455,0,0
203.175000000029,0,1460.2533336455,0,0
203.200000000029,0,1460.2533336455,0,0
203.225000000029,0,1460.2533336455,0,0
203.250000000029,0,1460.2533336455,0,0
203.275000000029,0,1460.2533336455,0,0
203.300000000029,0,1460.2533336455,0,0
203.325000000029,0,1460.2533336455,0,0
203.350000000029,1,1460.3354586455,0,0
203.375000000029,0,1460.4175836455,0,0
203.400000000029,0,1460.4175836455,0,0
203.425000000029,0,1460.4175836455,0,0
203.450000000029,0,1460.4175836455,0,0
203.475000000029,0,1460.4175836455,0,0
203.500000000029,0,1460.4175836455,0,0
203.525000000029,0,1460.4175836455,0,0
203.550000000029,0,1460.4175836455,0,0
203.575000000029,0,1460.4175836455,0,0
203.600000000029,0,1460.4175836455,0,0
203.625000000029,0,1460.4175836455,0,0
203.650000000029,0,1460.4175836455,0,0
203.675000000029,1,1460.4997086455,0,0
203.700000000029,0,1460.5818336455,0,0
203.725000000029,0,1460.5818336455,0,0
203.750000000029,0,1460.5818336455,0,0
203.775000000029,0,1460.5818336455,0,0
203.800000000029,0,1460.5818336455,0,0
203.825000000029,0,1460.5818336455,0,0
203.850000000029,0,1460.5818336455,0,0
203.875000000029,0,1460.5818336455,0,0
203.900000000029,0,1460.5818336455,0,0
203.925000000029,0,1460.5818336455,0,0
203.950000000029,0,1460.5818336455,0,0
203.975000000029,0,1460.5818336455,0,0
204.000000000029,0,1460.5818336455,0,0
204.025000000029,0,1460.5818336455,0,0

204.050000000029,0,1460.5818336455,0,0
204.075000000029,0,1460.5818336455,0,0
204.100000000029,0,1460.5818336455,0,0
204.125000000029,0,1460.5818336455,0,0
204.150000000029,0,1460.5818336455,0,0
204.175000000029,0,1460.5818336455,0,0
204.200000000029,0,1460.5818336455,0,0
204.225000000029,0,1460.5818336455,0,0
204.250000000029,0,1460.5818336455,0,0
204.275000000029,0,1460.5818336455,0,0
204.300000000029,0,1460.5818336455,0,0
204.325000000029,0,1460.5818336455,0,0
204.350000000029,0,1460.5818336455,0,0
204.375000000029,0,1460.5818336455,0,0
204.400000000029,0,1460.5818336455,0,0
204.425000000029,0,1460.5818336455,0,0
204.450000000029,1,1460.6639586455,0,0
204.475000000029,1,1460.8282086455,0,0
204.500000000029,0,1460.9103336455,0,0
204.525000000029,0,1460.9103336455,0,0
204.550000000029,3,1461.05257831807,0,0
204.575000000029,0,1461.19482299065,0,0
204.600000000029,0,1461.19482299065,0,0
204.625000000029,0,1461.19482299065,0,0
204.650000000029,2,1461.31096527946,0,0
204.675000000029,0,1461.42710756827,0,0
204.700000000029,0,1461.42710756827,0,0
204.725000000029,1,1461.50923256827,0,0
204.750000000029,5,1461.77499465092,0,0
204.775000000029,0,1461.95863173357,0,0
204.800000000029,2,1462.07477402238,0,0
204.825000000029,0,1462.19091631119,0,0
204.850000000029,0,1462.19091631119,0,0
204.875000000029,0,1462.19091631119,0,0
204.900000000029,1,1462.27304131119,0,0
204.925000000029,0,1462.35516631119,0,0
204.950000000029,1,1462.43729131119,0,0
204.975000000029,1,1462.60154131119,0,0
205.000000000029,0,1462.68366631119,0,0
205.025000000029,0,1462.68366631119,0,0
205.050000000029,0,1462.68366631119,0,0
205.075000000029,0,1462.68366631119,0,0
205.10000000003,0,1462.68366631119,0,0
205.12500000003,0,1462.68366631119,0,0
205.15000000003,0,1462.68366631119,0,0
205.17500000003,0,1462.68366631119,0,0
205.20000000003,0,1462.68366631119,0,0
205.22500000003,0,1462.68366631119,0,0
205.25000000003,0,1462.68366631119,0,0
205.27500000003,0,1462.68366631119,0,0
205.30000000003,0,1462.68366631119,0,0
205.32500000003,0,1462.68366631119,0,0

205.35000000003,0,1462.68366631119,0,0
205.37500000003,2,1462.7998086,0,0
205.40000000003,0,1462.91595088881,0,0
205.42500000003,0,1462.91595088881,0,0
205.45000000003,0,1462.91595088881,0,0
205.47500000003,0,1462.91595088881,0,0
205.50000000003,0,1462.91595088881,0,0
205.52500000003,0,1462.91595088881,0,0
205.55000000003,0,1462.91595088881,0,0
205.57500000003,0,1462.91595088881,0,0
205.60000000003,0,1462.91595088881,0,0
205.62500000003,0,1462.91595088881,0,0
205.65000000003,0,1462.91595088881,0,0
205.67500000003,0,1462.91595088881,0,0
205.70000000003,0,1462.91595088881,0,0
205.72500000003,0,1462.91595088881,0,0
205.75000000003,0,1462.91595088881,0,0
205.77500000003,1,1462.99807588881,0,0
205.80000000003,0,1463.08020088881,0,0
205.82500000003,0,1463.08020088881,0,0
205.85000000003,0,1463.08020088881,0,0
205.87500000003,0,1463.08020088881,0,0
205.90000000003,0,1463.08020088881,0,0
205.92500000003,0,1463.08020088881,0,0
205.95000000003,0,1463.08020088881,0,0
205.97500000003,0,1463.08020088881,0,0
206.00000000003,0,1463.08020088881,0,0
206.02500000003,0,1463.08020088881,0,0
206.05000000003,0,1463.08020088881,0,0
206.07500000003,0,1463.08020088881,0,0
206.10000000003,0,1463.08020088881,0,0
206.12500000003,0,1463.08020088881,0,0
206.15000000003,0,1463.08020088881,0,0
206.17500000003,0,1463.08020088881,0,0
206.20000000003,0,1463.08020088881,0,0
206.22500000003,0,1463.08020088881,0,0
206.25000000003,0,1463.08020088881,0,0
206.27500000003,0,1463.08020088881,0,0
206.30000000003,0,1463.08020088881,0,0
206.32500000003,0,1463.08020088881,0,0
206.35000000003,0,1463.08020088881,0,0
206.37500000003,0,1463.08020088881,0,0
206.40000000003,0,1463.08020088881,0,0
206.42500000003,0,1463.08020088881,0,0
206.45000000003,0,1463.08020088881,0,0
206.47500000003,0,1463.08020088881,0,0
206.50000000003,1,1463.16232588881,0,0
206.52500000003,0,1463.24445088881,0,0
206.55000000003,0,1463.24445088881,0,0
206.57500000003,1,1463.32657588881,0,0
206.60000000003,1,1463.49082588881,0,0
206.62500000003,0,1463.57295088881,0,0

206.65000000003,0,1463.57295088881,0,0
206.67500000003,0,1463.57295088881,0,0
206.70000000003,0,1463.57295088881,0,0
206.72500000003,0,1463.57295088881,0,0
206.75000000003,0,1463.57295088881,0,0
206.77500000003,1,1463.65507588881,0,0
206.80000000003,0,1463.73720088881,0,0
206.82500000003,0,1463.73720088881,0,0
206.85000000003,1,1463.81932588881,0,0
206.87500000003,0,1463.90145088881,0,0
206.90000000003,0,1463.90145088881,0,0
206.92500000003,0,1463.90145088881,0,0
206.95000000003,0,1463.90145088881,0,0
206.97500000003,0,1463.90145088881,0,0
207.00000000003,0,1463.90145088881,0,0
207.02500000003,0,1463.90145088881,0,0
207.05000000003,0,1463.90145088881,0,0
207.07500000003,0,1463.90145088881,0,0
207.10000000003,0,1463.90145088881,0,0
207.12500000003,0,1463.90145088881,0,0
207.15000000003,0,1463.90145088881,0,0
207.17500000003,0,1463.90145088881,0,0
207.20000000003,0,1463.90145088881,0,0
207.22500000003,0,1463.90145088881,0,0
207.25000000003,0,1463.90145088881,0,0
207.27500000003,0,1463.90145088881,0,0
207.30000000003,0,1463.90145088881,0,0
207.32500000003,0,1463.90145088881,0,0
207.35000000003,0,1463.90145088881,0,0
207.37500000003,0,1463.90145088881,0,0
207.40000000003,0,1463.90145088881,0,0
207.42500000003,0,1463.90145088881,0,0
207.45000000003,0,1463.90145088881,0,0
207.47500000003,0,1463.90145088881,0,0
207.50000000003,0,1463.90145088881,0,0
207.52500000003,0,1463.90145088881,0,0
207.55000000003,0,1463.90145088881,0,0
207.57500000003,0,1463.90145088881,0,0
207.60000000003,0,1463.90145088881,0,0
207.62500000003,1,1463.98357588881,0,0
207.65000000003,0,1464.06570088881,0,0
207.67500000003,0,1464.06570088881,0,0
207.70000000003,0,1464.06570088881,0,0
207.72500000003,0,1464.06570088881,0,0
207.75000000003,0,1464.06570088881,0,0
207.77500000003,0,1464.06570088881,0,0
207.80000000003,0,1464.06570088881,0,0
207.82500000003,0,1464.06570088881,0,0
207.85000000003,0,1464.06570088881,0,0
207.87500000003,0,1464.06570088881,0,0
207.90000000003,0,1464.06570088881,0,0
207.92500000003,0,1464.06570088881,0,0

207.95000000003,0,1464.06570088881,0,0
207.97500000003,0,1464.06570088881,0,0
208.00000000003,0,1464.06570088881,0,0
208.02500000003,0,1464.06570088881,0,0
208.05000000003,0,1464.06570088881,0,0
208.07500000003,0,1464.06570088881,0,0
208.10000000003,0,1464.06570088881,0,0
208.12500000003,0,1464.06570088881,0,0
208.15000000003,0,1464.06570088881,0,0
208.17500000003,0,1464.06570088881,0,0
208.20000000003,0,1464.06570088881,0,0
208.22500000003,0,1464.06570088881,0,0
208.25000000003,1,1464.14782588881,0,0
208.27500000003,2,1464.34609317762,0,0
208.30000000003,0,1464.46223546643,0,0
208.32500000003,1,1464.54436046643,0,0
208.35000000003,0,1464.62648546643,0,0
208.37500000003,1,1464.70861046643,0,0
208.40000000003,0,1464.79073546643,0,0
208.42500000003,1,1464.87286046643,0,0
208.45000000003,0,1464.95498546643,0,0
208.47500000003,0,1464.95498546643,0,0
208.50000000003,0,1464.95498546643,0,0
208.52500000003,0,1464.95498546643,0,0
208.55000000003,0,1464.95498546643,0,0
208.57500000003,0,1464.95498546643,0,0
208.60000000003,0,1464.95498546643,0,0
208.62500000003,0,1464.95498546643,0,0
208.65000000003,0,1464.95498546643,0,0
208.67500000003,0,1464.95498546643,0,0
208.70000000003,0,1464.95498546643,0,0
208.72500000003,0,1464.95498546643,0,0
208.75000000003,0,1464.95498546643,0,0
208.77500000003,0,1464.95498546643,0,0
208.80000000003,0,1464.95498546643,0,0
208.82500000003,0,1464.95498546643,0,0
208.85000000003,0,1464.95498546643,0,0
208.87500000003,0,1464.95498546643,0,0
208.90000000003,1,1465.03711046643,0,0
208.92500000003,0,1465.11923546643,0,0
208.95000000003,0,1465.11923546643,0,0
208.97500000003,0,1465.11923546643,0,0
209.00000000003,0,1465.11923546643,0,0
209.02500000003,0,1465.11923546643,0,0
209.05000000003,0,1465.11923546643,0,0
209.07500000003,0,1465.11923546643,0,0
209.10000000003,0,1465.11923546643,0,0
209.12500000003,0,1465.11923546643,0,0
209.15000000003,0,1465.11923546643,0,0
209.17500000003,0,1465.11923546643,0,0
209.20000000003,0,1465.11923546643,0,0
209.22500000003,0,1465.11923546643,0,0

209.25000000003,0,1465.11923546643,0,0
209.27500000003,0,1465.11923546643,0,0
209.30000000003,0,1465.11923546643,0,0
209.32500000003,0,1465.11923546643,0,0
209.35000000003,0,1465.11923546643,0,0
209.37500000003,0,1465.11923546643,0,0
209.40000000003,0,1465.11923546643,0,0
209.42500000003,0,1465.11923546643,0,0
209.45000000003,13,1465.41534136493,0,1
209.47500000003,17,1466.05005731293,0,1
209.500000000031,16,1466.71716736243,0,1
209.525000000031,13,1467.34177326093,0,1
209.550000000031,12,1467.92236850457,0,1
209.575000000031,11,1468.47923566062,0,1
209.600000000031,11,1469.02399128244,0,1
209.625000000031,7,1469.51365141976,0,0
209.650000000031,7,1469.94821607261,0,0
209.675000000031,6,1470.36666274415,0,0
209.700000000031,3,1470.71007176185,0,0
209.725000000031,3,1470.99456110699,0,0
209.750000000031,2,1471.25294806838,0,0
209.775000000031,2,1471.485232646,0,0
209.800000000031,2,1471.71751722362,0,0
209.825000000031,2,1471.94980180124,0,0
209.850000000031,2,1472.18208637886,0,0
209.875000000031,2,1472.41437095648,0,0
209.900000000031,3,1472.67275791786,0,0
209.925000000031,3,1472.957247263,0,0
209.950000000031,3,1473.24173660814,0,0
209.975000000031,4,1473.54823128071,0,0
210.000000000031,5,1473.89611836337,0,0
210.025000000031,6,1474.28091979115,0,0
210.050000000031,9,1474.72845913627,0,0
210.075000000031,7,1475.19211646269,0,0
210.100000000031,8,1475.64168336673,0,0
210.125000000031,9,1476.12034294435,0,0
210.150000000031,10,1476.62641999719,23.2157781786209,1
210.175000000031,10,1477.14582410288,23.2157781786209,1
210.200000000031,11,1477.67790396663,23.2157781786209,1
210.225000000031,10,1478.20998383038,23.2157781786209,1
210.250000000031,9,1478.71606088322,23.2157781786209,1
210.275000000031,10,1479.22213793606,23.2157781786209,1
210.300000000031,11,1479.75421779981,23.2157781786209,1
210.325000000031,12,1480.31108495586,23.2157781786209,1
210.350000000031,14,1480.90285791389,23.2157781786209,1
210.375000000031,16,1481.53864152678,23.2157781786209,1
210.400000000031,18,1482.21556839321,23.2157781786209,1
210.425000000031,16,1482.89249525964,23.2157781786209,1
210.450000000031,17,1483.55960530914,23.2157781786209,1
210.475000000031,17,1484.23682540815,23.2157781786209,1
210.500000000031,18,1484.92386232408,23.2157781786209,1
210.525000000031,17,1485.61089924002,23.2157781786209,1

210.550000000031,13,1486.24561518802,23.2157781786209,1
210.575000000031,11,1486.81409889742,23.2157781786209,1
210.600000000031,11,1487.35885451924,23.2157781786209,1
210.625000000031,10,1487.89093438299,23.2157781786209,1
210.650000000031,8,1488.38292101345,23.2157781786209,1
210.675000000031,6,1488.8163699362,23.2157781786209,1
210.700000000031,5,1489.20117136397,23.2157781786209,1
210.725000000031,5,1489.56844552928,23.2157781786209,1
210.750000000031,5,1489.93571969458,23.2157781786209,1
210.775000000031,6,1490.32052112236,23.2157781786209,1
210.800000000031,7,1490.73896779391,23.2157781786209,1
210.825000000031,10,1491.21595217317,23.2157781786209,1
210.850000000031,11,1491.74803203692,23.2157781786209,1
210.875000000031,12,1492.30489919297,23.2157781786209,1
210.900000000031,13,1492.88549443661,23.2157781786209,1
210.925000000031,16,1493.51010033511,23.2157781786209,1
210.950000000031,15,1494.15666909242,23.2157781786209,1
210.975000000031,16,1494.80323784972,23.2157781786209,1
211.000000000031,16,1495.46023784972,23.2157781786209,1
211.025000000031,17,1496.12734789923,23.2157781786209,1
211.050000000031,17,1496.80456799824,23.2157781786209,1
211.075000000031,18,1497.49160491417,23.2157781786209,1
211.100000000031,18,1498.18845864703,23.2157781786209,1
211.125000000031,18,1498.88531237989,23.2157781786209,1
211.150000000031,18,1499.58216611275,23.2157781786209,1
211.175000000031,18,1500.27901984561,23.2157781786209,1
211.200000000031,18,1500.97587357847,23.2157781786209,1
211.225000000031,15,1501.6423692022,23.2157781786209,1
211.250000000031,14,1502.2677215724,23.2157781786209,1
211.275000000031,14,1502.88228879818,23.2157781786209,1
211.300000000031,14,1503.49685602395,23.2157781786209,1
211.325000000031,13,1504.10024553534,23.2157781786209,1
211.350000000031,13,1504.69245733234,23.2157781786209,1
211.375000000031,12,1505.27305257598,23.2157781786209,1
211.400000000031,12,1505.84203126626,23.2157781786209,1
211.425000000031,11,1506.39889842231,23.2157781786209,1
211.450000000031,11,1506.94365404413,23.2157781786209,1
211.475000000031,10,1507.47573390788,23.2157781786209,1
211.500000000031,8,1507.96772053834,23.2157781786209,1
211.525000000031,7,1508.41728744238,0,0
211.550000000031,6,1508.83573411393,0,0
211.575000000031,3,1509.17914313163,0,0
211.600000000031,1,1509.4035128042,0,0
211.625000000031,0,1509.4856378042,0,0
211.650000000031,0,1509.4856378042,0,0
211.675000000031,0,1509.4856378042,0,0
211.700000000031,0,1509.4856378042,0,0
211.725000000031,0,1509.4856378042,0,0
211.750000000031,0,1509.4856378042,0,0
211.775000000031,0,1509.4856378042,0,0
211.800000000031,0,1509.4856378042,0,0
211.825000000031,0,1509.4856378042,0,0

211.850000000031,0,1509.4856378042,0,0
211.875000000031,0,1509.4856378042,0,0
211.900000000031,0,1509.4856378042,0,0
211.925000000031,0,1509.4856378042,0,0
211.950000000031,0,1509.4856378042,0,0
211.975000000031,1,1509.5677628042,0,0
212.000000000031,0,1509.6498878042,0,0
212.025000000031,0,1509.6498878042,0,0
212.050000000031,0,1509.6498878042,0,0
212.075000000031,1,1509.7320128042,0,0
212.100000000031,0,1509.8141378042,0,0
212.125000000031,0,1509.8141378042,0,0
212.150000000031,0,1509.8141378042,0,0
212.175000000031,0,1509.8141378042,0,0
212.200000000031,0,1509.8141378042,0,0
212.225000000031,0,1509.8141378042,0,0
212.250000000031,0,1509.8141378042,0,0
212.275000000031,0,1509.8141378042,0,0
212.300000000031,0,1509.8141378042,0,0
212.325000000031,0,1509.8141378042,0,0
212.350000000031,0,1509.8141378042,0,0
212.375000000031,0,1509.8141378042,0,0
212.400000000031,0,1509.8141378042,0,0
212.425000000031,0,1509.8141378042,0,0
212.450000000031,0,1509.8141378042,0,0
212.475000000031,0,1509.8141378042,0,0
212.500000000031,1,1509.8962628042,0,0
212.525000000031,0,1509.9783878042,0,0
212.550000000031,0,1509.9783878042,0,0
212.575000000031,0,1509.9783878042,0,0
212.600000000031,0,1509.9783878042,0,0
212.625000000031,0,1509.9783878042,0,0
212.650000000031,0,1509.9783878042,0,0
212.675000000031,0,1509.9783878042,0,0
212.700000000031,0,1509.9783878042,0,0
212.725000000031,0,1509.9783878042,0,0
212.750000000031,0,1509.9783878042,0,0
212.775000000031,0,1509.9783878042,0,0
212.800000000031,0,1509.9783878042,0,0
212.825000000031,0,1509.9783878042,0,0
212.850000000031,0,1509.9783878042,0,0
212.875000000031,0,1509.9783878042,0,0
212.900000000031,0,1509.9783878042,0,0
212.925000000031,0,1509.9783878042,0,0
212.950000000031,0,1509.9783878042,0,0
212.975000000031,0,1509.9783878042,0,0
213.000000000031,0,1509.9783878042,0,0
213.025000000031,0,1509.9783878042,0,0
213.050000000031,0,1509.9783878042,0,0
213.075000000031,0,1509.9783878042,0,0
213.100000000031,0,1509.9783878042,0,0
213.125000000031,0,1509.9783878042,0,0

213.150000000031,0,1509.9783878042,0,0
213.175000000031,1,1510.0605128042,0,0
213.200000000031,0,1510.1426378042,0,0
213.225000000031,0,1510.1426378042,0,0
213.250000000031,0,1510.1426378042,0,0
213.275000000031,0,1510.1426378042,0,0
213.300000000031,1,1510.2247628042,0,0
213.325000000031,1,1510.3890128042,0,0
213.350000000031,1,1510.5532628042,0,0
213.375000000031,0,1510.6353878042,0,0
213.400000000031,0,1510.6353878042,0,0
213.425000000031,0,1510.6353878042,0,0
213.450000000031,0,1510.6353878042,0,0
213.475000000031,0,1510.6353878042,0,0
213.500000000031,0,1510.6353878042,0,0
213.525000000031,0,1510.6353878042,0,0
213.550000000031,0,1510.6353878042,0,0
213.575000000031,0,1510.6353878042,0,0
213.600000000031,0,1510.6353878042,0,0
213.625000000031,0,1510.6353878042,0,0
213.650000000031,0,1510.6353878042,0,0
213.675000000031,0,1510.6353878042,0,0
213.700000000031,0,1510.6353878042,0,0
213.725000000031,0,1510.6353878042,0,0
213.750000000031,0,1510.6353878042,0,0
213.775000000031,0,1510.6353878042,0,0
213.800000000031,0,1510.6353878042,0,0
213.825000000031,1,1510.7175128042,0,0
213.850000000031,0,1510.7996378042,0,0
213.875000000031,1,1510.8817628042,0,0
213.900000000032,0,1510.9638878042,0,0
213.925000000032,0,1510.9638878042,0,0
213.950000000032,1,1511.0460128042,0,0
213.975000000032,1,1511.2102628042,0,0
214.000000000032,1,1511.3745128042,0,0
214.025000000032,1,1511.5387628042,0,0
214.050000000032,1,1511.7030128042,0,0
214.075000000032,0,1511.7851378042,0,0
214.100000000032,0,1511.7851378042,0,0
214.125000000032,0,1511.7851378042,0,0
214.150000000032,0,1511.7851378042,0,0
214.175000000032,1,1511.8672628042,0,0
214.200000000032,1,1512.0315128042,0,0
214.225000000032,0,1512.1136378042,0,0
214.250000000032,1,1512.1957628042,0,0
214.275000000032,0,1512.2778878042,0,0
214.300000000032,0,1512.2778878042,0,0
214.325000000032,1,1512.3600128042,0,0
214.350000000032,0,1512.4421378042,0,0
214.375000000032,0,1512.4421378042,0,0
214.400000000032,0,1512.4421378042,0,0
214.425000000032,0,1512.4421378042,0,0

214.450000000032,0,1512.4421378042,0,0
214.475000000032,0,1512.4421378042,0,0
214.500000000032,0,1512.4421378042,0,0
214.525000000032,0,1512.4421378042,0,0
214.550000000032,0,1512.4421378042,0,0
214.575000000032,0,1512.4421378042,0,0
214.600000000032,0,1512.4421378042,0,0
214.625000000032,0,1512.4421378042,0,0
214.650000000032,0,1512.4421378042,0,0
214.675000000032,0,1512.4421378042,0,0
214.700000000032,0,1512.4421378042,0,0
214.725000000032,0,1512.4421378042,0,0
214.750000000032,0,1512.4421378042,0,0
214.775000000032,0,1512.4421378042,0,0
214.800000000032,0,1512.4421378042,0,0
214.825000000032,0,1512.4421378042,0,0
214.850000000032,0,1512.4421378042,0,0
214.875000000032,0,1512.4421378042,0,0
214.900000000032,0,1512.4421378042,0,0
214.925000000032,0,1512.4421378042,0,0
214.950000000032,0,1512.4421378042,0,0
214.975000000032,0,1512.4421378042,0,0
215.000000000032,0,1512.4421378042,0,0
215.025000000032,0,1512.4421378042,0,0
215.050000000032,0,1512.4421378042,0,0
215.075000000032,0,1512.4421378042,0,0
215.100000000032,0,1512.4421378042,0,0
215.125000000032,0,1512.4421378042,0,0
215.150000000032,0,1512.4421378042,0,0
215.175000000032,0,1512.4421378042,0,0
215.200000000032,0,1512.4421378042,0,0
215.225000000032,0,1512.4421378042,0,0
215.250000000032,0,1512.4421378042,0,0
215.275000000032,0,1512.4421378042,0,0
215.300000000032,0,1512.4421378042,0,0
215.325000000032,0,1512.4421378042,0,0
215.350000000032,0,1512.4421378042,0,0
215.375000000032,0,1512.4421378042,0,0
215.400000000032,0,1512.4421378042,0,0
215.425000000032,0,1512.4421378042,0,0
215.450000000032,0,1512.4421378042,0,0
215.475000000032,0,1512.4421378042,0,0
215.500000000032,0,1512.4421378042,0,0
215.525000000032,1,1512.52426280419,0,0
215.550000000032,1,1512.68851280419,0,0
215.575000000032,0,1512.77063780419,0,0
215.600000000032,0,1512.77063780419,0,0
215.625000000032,0,1512.77063780419,0,0
215.650000000032,1,1512.85276280419,0,0
215.675000000032,0,1512.93488780419,0,0
215.700000000032,0,1512.93488780419,0,0
215.725000000032,0,1512.93488780419,0,0

215.750000000032,0,1512.93488780419,0,0
215.775000000032,0,1512.93488780419,0,0
215.800000000032,0,1512.93488780419,0,0
215.825000000032,0,1512.93488780419,0,0
215.850000000032,0,1512.93488780419,0,0
215.875000000032,5,1513.11852488685,0,0
215.900000000032,0,1513.3021619695,0,0
215.925000000032,0,1513.3021619695,0,0
215.950000000032,1,1513.3842869695,0,0
215.975000000032,1,1513.5485369695,0,0
216.000000000032,0,1513.6306619695,0,0
216.025000000032,0,1513.6306619695,0,0
216.050000000032,0,1513.6306619695,0,0
216.075000000032,2,1513.74680425831,0,0
216.100000000032,0,1513.86294654712,0,0
216.125000000032,0,1513.86294654712,0,0
216.150000000032,1,1513.94507154712,0,0
216.175000000032,0,1514.02719654712,0,0
216.200000000032,1,1514.10932154712,0,0
216.225000000032,1,1514.27357154712,0,0
216.250000000032,0,1514.35569654712,0,0
216.275000000032,1,1514.43782154712,0,0
216.300000000032,1,1514.60207154712,0,0
216.325000000032,0,1514.68419654712,0,0
216.350000000032,0,1514.68419654712,0,0
216.375000000032,0,1514.68419654712,0,0
216.400000000032,0,1514.68419654712,0,0
216.425000000032,0,1514.68419654712,0,0
216.450000000032,0,1514.68419654712,0,0
216.475000000032,0,1514.68419654712,0,0
216.500000000032,1,1514.76632154712,0,0
216.525000000032,0,1514.84844654712,0,0
216.550000000032,1,1514.93057154712,0,0
216.575000000032,0,1515.01269654712,0,0
216.600000000032,0,1515.01269654712,0,0
216.625000000032,0,1515.01269654712,0,0
216.650000000032,1,1515.09482154712,0,0
216.675000000032,1,1515.25907154712,0,0
216.700000000032,0,1515.34119654712,0,0
216.725000000032,0,1515.34119654712,0,0
216.750000000032,0,1515.34119654712,0,0
216.775000000032,0,1515.34119654712,0,0
216.800000000032,0,1515.34119654712,0,0
216.825000000032,0,1515.34119654712,0,0
216.850000000032,0,1515.34119654712,0,0
216.875000000032,0,1515.34119654712,0,0
216.900000000032,0,1515.34119654712,0,0
216.925000000032,1,1515.42332154712,0,0
216.950000000032,0,1515.50544654712,0,0
216.975000000032,0,1515.50544654712,0,0
217.000000000032,0,1515.50544654712,0,0
217.025000000032,0,1515.50544654712,0,0

217.050000000032,0,1515.50544654712,0,0
217.075000000032,1,1515.58757154712,0,0
217.100000000032,1,1515.75182154712,0,0
217.125000000032,1,1515.91607154712,0,0
217.150000000032,0,1515.99819654712,0,0
217.175000000032,0,1515.99819654712,0,0
217.200000000032,0,1515.99819654712,0,0
217.225000000032,1,1516.08032154712,0,0
217.250000000032,0,1516.16244654712,0,0
217.275000000032,0,1516.16244654712,0,0
217.300000000032,0,1516.16244654712,0,0
217.325000000032,0,1516.16244654712,0,0
217.350000000032,0,1516.16244654712,0,0
217.375000000032,0,1516.16244654712,0,0
217.400000000032,0,1516.16244654712,0,0
217.425000000032,0,1516.16244654712,0,0
217.450000000032,1,1516.24457154712,0,0
217.475000000032,0,1516.32669654712,0,0
217.500000000032,0,1516.32669654712,0,0
217.525000000032,0,1516.32669654712,0,0
217.550000000032,0,1516.32669654712,0,0
217.575000000032,0,1516.32669654712,0,0
217.600000000032,0,1516.32669654712,0,0
217.625000000032,0,1516.32669654712,0,0
217.650000000032,0,1516.32669654712,0,0
217.675000000032,1,1516.40882154712,0,0
217.700000000032,0,1516.49094654712,0,0
217.725000000032,1,1516.57307154712,0,0
217.750000000032,0,1516.65519654712,0,0
217.775000000032,0,1516.65519654712,0,0
217.800000000032,0,1516.65519654712,0,0
217.825000000032,1,1516.73732154712,0,0
217.850000000032,0,1516.81944654712,0,0
217.875000000032,0,1516.81944654712,0,0
217.900000000032,0,1516.81944654712,0,0
217.925000000032,0,1516.81944654712,0,0
217.950000000032,0,1516.81944654712,0,0
217.975000000032,0,1516.81944654712,0,0
218.000000000032,0,1516.81944654712,0,0
218.025000000032,0,1516.81944654712,0,0
218.050000000032,0,1516.81944654712,0,0
218.075000000032,0,1516.81944654712,0,0
218.100000000032,0,1516.81944654712,0,0
218.125000000032,0,1516.81944654712,0,0
218.150000000032,0,1516.81944654712,0,0
218.175000000032,0,1516.81944654712,0,0
218.200000000032,0,1516.81944654712,0,0
218.225000000032,0,1516.81944654712,0,0
218.250000000032,0,1516.81944654712,0,0
218.275000000032,0,1516.81944654712,0,0
218.300000000033,0,1516.81944654712,0,0
218.325000000033,0,1516.81944654712,0,0

218.350000000033,1,1516.90157154712,0,0
218.375000000033,0,1516.98369654712,0,0
218.400000000033,0,1516.98369654712,0,0
218.425000000033,0,1516.98369654712,0,0
218.450000000033,0,1516.98369654712,0,0
218.475000000033,0,1516.98369654712,0,0
218.500000000033,0,1516.98369654712,0,0
218.525000000033,0,1516.98369654712,0,0
218.550000000033,0,1516.98369654712,0,0
218.575000000033,0,1516.98369654712,0,0
218.600000000033,0,1516.98369654712,0,0
218.625000000033,0,1516.98369654712,0,0
218.650000000033,0,1516.98369654712,0,0
218.675000000033,0,1516.98369654712,0,0
218.700000000033,0,1516.98369654712,0,0
218.725000000033,0,1516.98369654712,0,0
218.750000000033,0,1516.98369654712,0,0
218.775000000033,0,1516.98369654712,0,0
218.800000000033,0,1516.98369654712,0,0
218.825000000033,0,1516.98369654712,0,0
218.850000000033,0,1516.98369654712,0,0
218.875000000033,0,1516.98369654712,0,0
218.900000000033,0,1516.98369654712,0,0
218.925000000033,0,1516.98369654712,0,0
218.950000000033,0,1516.98369654712,0,0
218.975000000033,0,1516.98369654712,0,0
219.000000000033,0,1516.98369654712,0,0
219.025000000033,0,1516.98369654712,0,0
219.050000000033,0,1516.98369654712,0,0
219.075000000033,0,1516.98369654712,0,0
219.100000000033,0,1516.98369654712,0,0
219.125000000033,0,1516.98369654712,0,0
219.150000000033,0,1516.98369654712,0,0
219.175000000033,0,1516.98369654712,0,0
219.200000000033,1,1517.06582154712,0,0
219.225000000033,2,1517.26408883593,0,0
219.250000000033,6,1517.58139546986,0,0
219.275000000033,9,1518.02893481499,0,0
219.300000000033,10,1518.53501186783,21.4415109000953,1
219.325000000033,12,1519.07920326581,21.4415109000953,1
219.350000000033,13,1519.65979850945,21.4415109000953,1
219.375000000033,13,1520.25201030645,21.4415109000953,1
219.400000000033,12,1520.83260555009,21.4415109000953,1
219.425000000033,10,1521.37679694807,21.4415109000953,1
219.450000000033,12,1521.92098834606,21.4415109000953,1
219.475000000033,10,1522.46517974404,21.4415109000953,1
219.500000000033,10,1522.98458384973,21.4415109000953,1
219.525000000033,12,1523.52877524771,21.4415109000953,1
219.550000000033,1,1523.89538959285,21.4415109000953,1
219.575000000033,5,1524.16115167551,0,0
219.600000000033,5,1524.52842584081,0,0
219.625000000033,2,1524.82820521227,0,0

219.650000000033,4,1525.10859750108,0,0
219.675000000033,7,1525.4901298275,0,0
219.700000000033,2,1525.82355444274,0,0
219.725000000033,4,1526.10394673155,0,0
219.750000000033,1,1526.35032173154,0,0
219.775000000033,2,1526.54858902035,0,0
219.800000000033,3,1526.80697598174,0,0
219.825000000033,2,1527.06536294312,0,0
219.850000000033,2,1527.29764752074,0,0
219.875000000033,2,1527.52993209836,0,0
219.900000000033,1,1527.72819938717,0,0
219.925000000033,0,1527.81032438717,0,0
219.950000000033,1,1527.89244938717,0,0
219.975000000033,1,1528.05669938717,0,0
220.000000000033,1,1528.22094938717,0,0
220.025000000033,0,1528.30307438717,0,0
220.050000000033,0,1528.30307438717,0,0
220.075000000033,0,1528.30307438717,0,0
220.100000000033,0,1528.30307438717,0,0
220.125000000033,0,1528.30307438717,0,0
220.150000000033,0,1528.30307438717,0,0
220.175000000033,0,1528.30307438717,0,0
220.200000000033,0,1528.30307438717,0,0
220.225000000033,0,1528.30307438717,0,0
220.250000000033,0,1528.30307438717,0,0
220.275000000033,0,1528.30307438717,0,0
220.300000000033,1,1528.38519938717,0,0
220.325000000033,0,1528.46732438717,0,0
220.350000000033,0,1528.46732438717,0,0
220.375000000033,0,1528.46732438717,0,0
220.400000000033,0,1528.46732438717,0,0
220.425000000033,0,1528.46732438717,0,0
220.450000000033,0,1528.46732438717,0,0
220.475000000033,0,1528.46732438717,0,0
220.500000000033,0,1528.46732438717,0,0
220.525000000033,0,1528.46732438717,0,0
220.550000000033,0,1528.46732438717,0,0
220.575000000033,0,1528.46732438717,0,0
220.600000000033,0,1528.46732438717,0,0
220.625000000033,0,1528.46732438717,0,0
220.650000000033,0,1528.46732438717,0,0
220.675000000033,0,1528.46732438717,0,0
220.700000000033,0,1528.46732438717,0,0
220.725000000033,0,1528.46732438717,0,0
220.750000000033,0,1528.46732438717,0,0
220.775000000033,0,1528.46732438717,0,0
220.800000000033,0,1528.46732438717,0,0
220.825000000033,0,1528.46732438717,0,0
220.850000000033,0,1528.46732438717,0,0
220.875000000033,0,1528.46732438717,0,0
220.900000000033,0,1528.46732438717,0,0
220.925000000033,0,1528.46732438717,0,0

220.950000000033,0,1528.46732438717,0,0
220.975000000033,0,1528.46732438717,0,0
221.000000000033,0,1528.46732438717,0,0
221.025000000033,0,1528.46732438717,0,0
221.050000000033,0,1528.46732438717,0,0
221.075000000033,0,1528.46732438717,0,0
221.100000000033,0,1528.46732438717,0,0
221.125000000033,0,1528.46732438717,0,0
221.150000000033,0,1528.46732438717,0,0
221.175000000033,0,1528.46732438717,0,0
221.200000000033,1,1528.54944938717,0,0
221.225000000033,0,1528.63157438717,0,0
221.250000000033,0,1528.63157438717,0,0
221.275000000033,0,1528.63157438717,0,0
221.300000000033,1,1528.71369938717,0,0
221.325000000033,0,1528.79582438717,0,0
221.350000000033,0,1528.79582438717,0,0
221.375000000033,0,1528.79582438717,0,0
221.400000000033,0,1528.79582438717,0,0
221.425000000033,0,1528.79582438717,0,0
221.450000000033,0,1528.79582438717,0,0
221.475000000033,1,1528.87794938717,0,0
221.500000000033,0,1528.96007438717,0,0
221.525000000033,0,1528.96007438717,0,0
221.550000000033,0,1528.96007438717,0,0
221.575000000033,0,1528.96007438717,0,0
221.600000000033,0,1528.96007438717,0,0
221.625000000033,0,1528.96007438717,0,0
221.650000000033,0,1528.96007438717,0,0
221.675000000033,0,1528.96007438717,0,0
221.700000000033,0,1528.96007438717,0,0
221.725000000033,0,1528.96007438717,0,0
221.750000000033,0,1528.96007438717,0,0
221.775000000033,0,1528.96007438717,0,0
221.800000000033,0,1528.96007438717,0,0
221.825000000033,0,1528.96007438717,0,0
221.850000000033,1,1529.04219938717,0,0
221.875000000033,0,1529.12432438717,0,0
221.900000000033,0,1529.12432438717,0,0
221.925000000033,0,1529.12432438717,0,0
221.950000000033,1,1529.20644938717,0,0
221.975000000033,0,1529.28857438717,0,0
222.000000000033,0,1529.28857438717,0,0
222.025000000033,0,1529.28857438717,0,0
222.050000000033,1,1529.37069938717,0,0
222.075000000033,0,1529.45282438717,0,0
222.100000000033,0,1529.45282438717,0,0
222.125000000033,0,1529.45282438717,0,0
222.150000000033,0,1529.45282438717,0,0
222.175000000033,0,1529.45282438717,0,0
222.200000000033,0,1529.45282438717,0,0
222.225000000033,0,1529.45282438717,0,0

222.250000000033,0,1529.45282438717,0,0
222.275000000033,0,1529.45282438717,0,0
222.300000000033,0,1529.45282438717,0,0
222.325000000033,0,1529.45282438717,0,0
222.350000000033,0,1529.45282438717,0,0
222.375000000033,0,1529.45282438717,0,0
222.400000000033,0,1529.45282438717,0,0
222.425000000033,0,1529.45282438717,0,0
222.450000000033,0,1529.45282438717,0,0
222.475000000033,0,1529.45282438717,0,0
222.500000000033,0,1529.45282438717,0,0
222.525000000033,0,1529.45282438717,0,0
222.550000000033,0,1529.45282438717,0,0
222.575000000033,0,1529.45282438717,0,0
222.600000000033,0,1529.45282438717,0,0
222.625000000033,0,1529.45282438717,0,0
222.650000000033,0,1529.45282438717,0,0
222.675000000033,0,1529.45282438717,0,0
222.700000000034,0,1529.45282438717,0,0
222.725000000034,0,1529.45282438717,0,0
222.750000000034,0,1529.45282438717,0,0
222.775000000034,0,1529.45282438717,0,0
222.800000000034,0,1529.45282438717,0,0
222.825000000034,0,1529.45282438717,0,0
222.850000000034,0,1529.45282438717,0,0
222.875000000034,0,1529.45282438717,0,0
222.900000000034,0,1529.45282438717,0,0
222.925000000034,0,1529.45282438717,0,0
222.950000000034,0,1529.45282438717,0,0
222.975000000034,0,1529.45282438717,0,0
223.000000000034,0,1529.45282438717,0,0
223.025000000034,0,1529.45282438717,0,0
223.050000000034,0,1529.45282438717,0,0
223.075000000034,0,1529.45282438717,0,0
223.100000000034,0,1529.45282438717,0,0
223.125000000034,0,1529.45282438717,0,0
223.150000000034,0,1529.45282438717,0,0
223.175000000034,0,1529.45282438717,0,0
223.200000000034,0,1529.45282438717,0,0
223.225000000034,1,1529.53494938717,0,0
223.250000000034,0,1529.61707438717,0,0
223.275000000034,0,1529.61707438717,0,0
223.300000000034,0,1529.61707438717,0,0
223.325000000034,0,1529.61707438717,0,0
223.350000000034,1,1529.69919938717,0,0
223.375000000034,0,1529.78132438717,0,0
223.400000000034,0,1529.78132438717,0,0
223.425000000034,0,1529.78132438717,0,0
223.450000000034,0,1529.78132438717,0,0
223.475000000034,1,1529.86344938717,0,0
223.500000000034,0,1529.94557438717,0,0
223.525000000034,0,1529.94557438717,0,0

223.550000000034,6,1530.14673873229,0,0
223.575000000034,1,1530.43002807742,0,0
223.600000000034,1,1530.59427807742,0,0
223.625000000034,0,1530.67640307742,0,0
223.650000000034,0,1530.67640307742,0,0
223.675000000034,0,1530.67640307742,0,0
223.700000000034,0,1530.67640307742,0,0
223.725000000034,1,1530.75852807742,0,0
223.750000000034,0,1530.84065307742,0,0
223.775000000034,0,1530.84065307742,0,0
223.800000000034,0,1530.84065307742,0,0
223.825000000034,0,1530.84065307742,0,0
223.850000000034,0,1530.84065307742,0,0
223.875000000034,0,1530.84065307742,0,0
223.900000000034,0,1530.84065307742,0,0
223.925000000034,0,1530.84065307742,0,0
223.950000000034,0,1530.84065307742,0,0
223.975000000034,0,1530.84065307742,0,0
224.000000000034,0,1530.84065307742,0,0
224.025000000034,0,1530.84065307742,0,0
224.050000000034,0,1530.84065307742,0,0
224.075000000034,0,1530.84065307742,0,0
224.100000000034,0,1530.84065307742,0,0
224.125000000034,0,1530.84065307742,0,0
224.150000000034,0,1530.84065307742,0,0
224.175000000034,0,1530.84065307742,0,0
224.200000000034,0,1530.84065307742,0,0
224.225000000034,0,1530.84065307742,0,0
224.250000000034,0,1530.84065307742,0,0
224.275000000034,0,1530.84065307742,0,0
224.300000000034,0,1530.84065307742,0,0
224.325000000034,0,1530.84065307742,0,0
224.350000000034,0,1530.84065307742,0,0
224.375000000034,0,1530.84065307742,0,0
224.400000000034,0,1530.84065307742,0,0
224.425000000034,0,1530.84065307742,0,0
224.450000000034,0,1530.84065307742,0,0
224.475000000034,0,1530.84065307742,0,0
224.500000000034,0,1530.84065307742,0,0
224.525000000034,0,1530.84065307742,0,0
224.550000000034,0,1530.84065307742,0,0
224.575000000034,0,1530.84065307742,0,0
224.600000000034,0,1530.84065307742,0,0
224.625000000034,0,1530.84065307742,0,0
224.650000000034,0,1530.84065307742,0,0
224.675000000034,0,1530.84065307742,0,0
224.700000000034,0,1530.84065307742,0,0
224.725000000034,0,1530.84065307742,0,0
224.750000000034,0,1530.84065307742,0,0
224.775000000034,0,1530.84065307742,0,0
224.800000000034,0,1530.84065307742,0,0
224.825000000034,0,1530.84065307742,0,0

224.850000000034,0,1530.84065307742,0,0
224.875000000034,0,1530.84065307742,0,0
224.900000000034,0,1530.84065307742,0,0
224.925000000034,0,1530.84065307742,0,0
224.950000000034,0,1530.84065307742,0,0
224.975000000034,0,1530.84065307742,0,0
225.000000000034,0,1530.84065307742,0,0
225.025000000034,0,1530.84065307742,0,0
225.050000000034,0,1530.84065307742,0,0
225.075000000034,0,1530.84065307742,0,0
225.100000000034,0,1530.84065307742,0,0
225.125000000034,0,1530.84065307742,0,0
225.150000000034,0,1530.84065307742,0,0
225.175000000034,0,1530.84065307742,0,0
225.200000000034,0,1530.84065307742,0,0
225.225000000034,0,1530.84065307742,0,0
225.250000000034,0,1530.84065307742,0,0
225.275000000034,0,1530.84065307742,0,0
225.300000000034,0,1530.84065307742,0,0
225.325000000034,0,1530.84065307742,0,0
225.350000000034,0,1530.84065307742,0,0
225.375000000034,0,1530.84065307742,0,0
225.400000000034,0,1530.84065307742,0,0
225.425000000034,0,1530.84065307742,0,0
225.450000000034,0,1530.84065307742,0,0
225.475000000034,0,1530.84065307742,0,0
225.500000000034,0,1530.84065307742,0,0
225.525000000034,0,1530.84065307742,0,0
225.550000000034,0,1530.84065307742,0,0
225.575000000034,0,1530.84065307742,0,0
225.600000000034,0,1530.84065307742,0,0
225.625000000034,0,1530.84065307742,0,0
225.650000000034,0,1530.84065307742,0,0
225.675000000034,0,1530.84065307742,0,0
225.700000000034,0,1530.84065307742,0,0
225.725000000034,0,1530.84065307742,0,0
225.750000000034,0,1530.84065307742,0,0
225.775000000034,0,1530.84065307742,0,0
225.800000000034,0,1530.84065307742,0,0
225.825000000034,0,1530.84065307742,0,0
225.850000000034,0,1530.84065307742,0,0
225.875000000034,0,1530.84065307742,0,0
225.900000000034,0,1530.84065307742,0,0
225.925000000034,0,1530.84065307742,0,0
225.950000000034,0,1530.84065307742,0,0
225.975000000034,0,1530.84065307742,0,0
226.000000000034,0,1530.84065307742,0,0
226.025000000034,0,1530.84065307742,0,0
226.050000000034,0,1530.84065307742,0,0
226.075000000034,0,1530.84065307742,0,0
226.100000000034,0,1530.84065307742,0,0
226.125000000034,0,1530.84065307742,0,0

226.150000000034,0,1530.84065307742,0,0
226.175000000034,0,1530.84065307742,0,0
226.200000000034,0,1530.84065307742,0,0
226.225000000034,0,1530.84065307742,0,0
226.250000000034,0,1530.84065307742,0,0
226.275000000034,0,1530.84065307742,0,0
226.300000000034,0,1530.84065307742,0,0
226.325000000034,0,1530.84065307742,0,0
226.350000000034,0,1530.84065307742,0,0
226.375000000034,0,1530.84065307742,0,0
226.400000000034,0,1530.84065307742,0,0
226.425000000034,0,1530.84065307742,0,0
226.450000000034,0,1530.84065307742,0,0
226.475000000034,0,1530.84065307742,0,0
226.500000000034,0,1530.84065307742,0,0
226.525000000034,0,1530.84065307742,0,0
226.550000000034,0,1530.84065307742,0,0
226.575000000034,0,1530.84065307742,0,0
226.600000000034,0,1530.84065307742,0,0
226.625000000034,0,1530.84065307742,0,0
226.650000000034,0,1530.84065307742,0,0
226.675000000034,0,1530.84065307742,0,0
226.700000000034,0,1530.84065307742,0,0
226.725000000034,0,1530.84065307742,0,0
226.750000000034,0,1530.84065307742,0,0
226.775000000034,0,1530.84065307742,0,0
226.800000000034,0,1530.84065307742,0,0
226.825000000034,0,1530.84065307742,0,0
226.850000000034,0,1530.84065307742,0,0
226.875000000034,0,1530.84065307742,0,0
226.900000000034,0,1530.84065307742,0,0
226.925000000034,0,1530.84065307742,0,0
226.950000000034,0,1530.84065307742,0,0
226.975000000034,1,1530.92277807742,0,0
227.000000000034,0,1531.00490307742,0,0
227.025000000034,1,1531.08702807742,0,0
227.050000000034,0,1531.16915307742,0,0
227.075000000034,0,1531.16915307742,0,0
227.100000000035,0,1531.16915307742,0,0
227.125000000035,4,1531.33340307742,0,0
227.150000000035,0,1531.49765307742,0,0
227.175000000035,0,1531.49765307742,0,0
227.200000000035,5,1531.68129016007,0,0
227.225000000035,0,1531.86492724272,0,0
227.250000000035,0,1531.86492724272,0,0
227.275000000035,0,1531.86492724272,0,0
227.300000000035,2,1531.98106953153,0,0
227.325000000035,0,1532.09721182034,0,0
227.350000000035,0,1532.09721182034,0,0
227.375000000035,0,1532.09721182034,0,0
227.400000000035,0,1532.09721182034,0,0
227.425000000035,0,1532.09721182034,0,0

227.450000000035,0,1532.09721182034,0,0
227.475000000035,0,1532.09721182034,0,0
227.500000000035,0,1532.09721182034,0,0
227.525000000035,0,1532.09721182034,0,0
227.550000000035,0,1532.09721182034,0,0
227.575000000035,0,1532.09721182034,0,0
227.600000000035,0,1532.09721182034,0,0
227.625000000035,1,1532.17933682034,0,0
227.650000000035,0,1532.26146182034,0,0
227.675000000035,0,1532.26146182034,0,0
227.700000000035,0,1532.26146182034,0,0
227.725000000035,0,1532.26146182034,0,0
227.750000000035,0,1532.26146182034,0,0
227.775000000035,0,1532.26146182034,0,0
227.800000000035,0,1532.26146182034,0,0
227.825000000035,0,1532.26146182034,0,0
227.850000000035,0,1532.26146182034,0,0
227.875000000035,0,1532.26146182034,0,0
227.900000000035,0,1532.26146182034,0,0
227.925000000035,0,1532.26146182034,0,0
227.950000000035,0,1532.26146182034,0,0
227.975000000035,0,1532.26146182034,0,0
228.000000000035,0,1532.26146182034,0,0
228.025000000035,0,1532.26146182034,0,0
228.050000000035,0,1532.26146182034,0,0
228.075000000035,0,1532.26146182034,0,0
228.100000000035,0,1532.26146182034,0,0
228.125000000035,0,1532.26146182034,0,0
228.150000000035,0,1532.26146182034,0,0
228.175000000035,0,1532.26146182034,0,0
228.200000000035,0,1532.26146182034,0,0
228.225000000035,0,1532.26146182034,0,0
228.250000000035,0,1532.26146182034,0,0
228.275000000035,0,1532.26146182034,0,0
228.300000000035,0,1532.26146182034,0,0
228.325000000035,0,1532.26146182034,0,0
228.350000000035,0,1532.26146182034,0,0
228.375000000035,0,1532.26146182034,0,0
228.400000000035,0,1532.26146182034,0,0
228.425000000035,0,1532.26146182034,0,0
228.450000000035,0,1532.26146182034,0,0
228.475000000035,0,1532.26146182034,0,0
228.500000000035,0,1532.26146182034,0,0
228.525000000035,0,1532.26146182034,0,0
228.550000000035,0,1532.26146182034,0,0
228.575000000035,0,1532.26146182034,0,0
228.600000000035,0,1532.26146182034,0,0
228.625000000035,0,1532.26146182034,0,0
228.650000000035,0,1532.26146182034,0,0
228.675000000035,0,1532.26146182034,0,0
228.700000000035,0,1532.26146182034,0,0
228.725000000035,0,1532.26146182034,0,0

228.750000000035,0,1532.26146182034,0,0
228.775000000035,0,1532.26146182034,0,0
228.800000000035,1,1532.34358682034,0,0
228.825000000035,0,1532.42571182034,0,0
228.850000000035,0,1532.42571182034,0,0
228.875000000035,0,1532.42571182034,0,0
228.900000000035,0,1532.42571182034,0,0
228.925000000035,0,1532.42571182034,0,0
228.950000000035,0,1532.42571182034,0,0
228.975000000035,0,1532.42571182034,0,0
229.000000000035,0,1532.42571182034,0,0
229.025000000035,0,1532.42571182034,0,0
229.050000000035,0,1532.42571182034,0,0
229.075000000035,0,1532.42571182034,0,0
229.100000000035,0,1532.42571182034,0,0
229.125000000035,0,1532.42571182034,0,0
229.150000000035,0,1532.42571182034,0,0
229.175000000035,0,1532.42571182034,0,0
229.200000000035,0,1532.42571182034,0,0
229.225000000035,0,1532.42571182034,0,0
229.250000000035,0,1532.42571182034,0,0
229.275000000035,0,1532.42571182034,0,0
229.300000000035,0,1532.42571182034,0,0
229.325000000035,0,1532.42571182034,0,0
229.350000000035,0,1532.42571182034,0,0
229.375000000035,0,1532.42571182034,0,0
229.400000000035,0,1532.42571182034,0,0
229.425000000035,1,1532.50783682034,0,0
229.450000000035,0,1532.58996182034,0,0
229.475000000035,0,1532.58996182034,0,0
229.500000000035,0,1532.58996182034,0,0
229.525000000035,0,1532.58996182034,0,0
229.550000000035,0,1532.58996182034,0,0
229.575000000035,0,1532.58996182034,0,0
229.600000000035,0,1532.58996182034,0,0
229.625000000035,0,1532.58996182034,0,0
229.650000000035,0,1532.58996182034,0,0
229.675000000035,0,1532.58996182034,0,0
229.700000000035,0,1532.58996182034,0,0
229.725000000035,0,1532.58996182034,0,0
229.750000000035,1,1532.67208682034,0,0
229.775000000035,0,1532.75421182034,0,0
229.800000000035,0,1532.75421182034,0,0
229.825000000035,0,1532.75421182034,0,0
229.850000000035,0,1532.75421182034,0,0
229.875000000035,0,1532.75421182034,0,0
229.900000000035,0,1532.75421182034,0,0
229.925000000035,0,1532.75421182034,0,0
229.950000000035,0,1532.75421182034,0,0
229.975000000035,0,1532.75421182034,0,0
230.000000000035,0,1532.75421182034,0,0
230.025000000035,0,1532.75421182034,0,0

230.050000000035,1,1532.83633682034,0,0
230.075000000035,0,1532.91846182034,0,0
230.100000000035,0,1532.91846182034,0,0
230.125000000035,0,1532.91846182034,0,0
230.150000000035,0,1532.91846182034,0,0
230.175000000035,0,1532.91846182034,0,0
230.200000000035,0,1532.91846182034,0,0
230.225000000035,2,1533.03460410915,0,0
230.250000000035,0,1533.15074639796,0,0
230.275000000035,0,1533.15074639796,0,0
230.300000000035,0,1533.15074639796,0,0
230.325000000035,0,1533.15074639796,0,0
230.350000000035,0,1533.15074639796,0,0
230.375000000035,1,1533.23287139796,0,0
230.400000000035,0,1533.31499639796,0,0
230.425000000035,0,1533.31499639796,0,0
230.450000000035,0,1533.31499639796,0,0
230.475000000035,0,1533.31499639796,0,0
230.500000000035,0,1533.31499639796,0,0
230.525000000035,0,1533.31499639796,0,0
230.550000000035,0,1533.31499639796,0,0
230.575000000035,0,1533.31499639796,0,0
230.600000000035,0,1533.31499639796,0,0
230.625000000035,0,1533.31499639796,0,0
230.650000000035,0,1533.31499639796,0,0
230.675000000035,2,1533.43113868677,0,0
230.700000000035,0,1533.54728097558,0,0
230.725000000035,0,1533.54728097558,0,0
230.750000000035,4,1533.71153097558,0,0
230.775000000035,0,1533.87578097558,0,0
230.800000000035,3,1534.01802564815,0,0
230.825000000035,3,1534.30251499329,0,0
230.850000000035,0,1534.44475966587,0,0
230.875000000035,0,1534.44475966587,0,0
230.900000000035,2,1534.56090195468,0,0
230.925000000035,0,1534.67704424349,0,0
230.950000000035,2,1534.7931865323,0,0
230.975000000035,5,1535.09296590376,0,0
231.000000000035,0,1535.27660298641,0,0
231.025000000035,0,1535.27660298641,0,0
231.050000000035,0,1535.27660298641,0,0
231.075000000035,1,1535.35872798641,0,0
231.100000000035,1,1535.52297798641,0,0
231.125000000035,2,1535.72124527522,0,0
231.150000000035,1,1535.91951256403,0,0
231.175000000035,4,1536.16588756403,0,0
231.200000000035,3,1536.4723822366,0,0
231.225000000035,4,1536.77887690917,0,0
231.250000000035,5,1537.12676399182,0,0
231.275000000035,6,1537.5115654196,0,0
231.300000000035,6,1537.91389410986,0,0
231.325000000035,6,1538.31622280011,0,0

231.350000000035,5,1538.70102422789,0,0
231.375000000035,6,1539.08582565566,0,0
231.400000000035,5,1539.47062708344,0,0
231.425000000035,5,1539.83790124875,0,0
231.450000000035,5,1540.20517541405,0,0
231.475000000035,5,1540.57244957935,0,0
231.500000000036,4,1540.92033666201,0,0
231.525000000036,5,1541.26822374466,0,0
231.550000000036,5,1541.63549790996,0,0
231.575000000036,5,1542.00277207527,0,0
231.600000000036,4,1542.35065915792,0,0
231.625000000036,3,1542.65715383049,0,0
231.650000000036,2,1542.91554079187,0,0
231.675000000036,1,1543.11380808068,0,0
231.700000000036,0,1543.19593308068,0,0
231.725000000036,0,1543.19593308068,0,0
231.750000000036,0,1543.19593308068,0,0
231.775000000036,0,1543.19593308068,0,0
231.800000000036,0,1543.19593308068,0,0
231.825000000036,0,1543.19593308068,0,0
231.850000000036,0,1543.19593308068,0,0
231.875000000036,0,1543.19593308068,0,0
231.900000000036,0,1543.19593308068,0,0
231.925000000036,0,1543.19593308068,0,0
231.950000000036,0,1543.19593308068,0,0
231.975000000036,0,1543.19593308068,0,0
232.000000000036,0,1543.19593308068,0,0
232.025000000036,0,1543.19593308068,0,0
232.050000000036,0,1543.19593308068,0,0
232.075000000036,0,1543.19593308068,0,0
232.100000000036,0,1543.19593308068,0,0
232.125000000036,0,1543.19593308068,0,0
232.150000000036,0,1543.19593308068,0,0
232.175000000036,0,1543.19593308068,0,0
232.200000000036,0,1543.19593308068,0,0
232.225000000036,0,1543.19593308068,0,0
232.250000000036,0,1543.19593308068,0,0
232.275000000036,0,1543.19593308068,0,0
232.300000000036,0,1543.19593308068,0,0
232.325000000036,0,1543.19593308068,0,0
232.350000000036,0,1543.19593308068,0,0
232.375000000036,0,1543.19593308068,0,0
232.400000000036,0,1543.19593308068,0,0
232.425000000036,0,1543.19593308068,0,0
232.450000000036,0,1543.19593308068,0,0
232.475000000036,0,1543.19593308068,0,0
232.500000000036,1,1543.27805808068,0,0
232.525000000036,0,1543.36018308068,0,0
232.550000000036,1,1543.44230808068,0,0
232.575000000036,0,1543.52443308068,0,0
232.600000000036,0,1543.52443308068,0,0
232.625000000036,0,1543.52443308068,0,0

232.650000000036,0,1543.52443308068,0,0
232.675000000036,0,1543.52443308068,0,0
232.700000000036,0,1543.52443308068,0,0
232.725000000036,0,1543.52443308068,0,0
232.750000000036,0,1543.52443308068,0,0
232.775000000036,0,1543.52443308068,0,0
232.800000000036,0,1543.52443308068,0,0
232.825000000036,0,1543.52443308068,0,0
232.850000000036,0,1543.52443308068,0,0
232.875000000036,0,1543.52443308068,0,0
232.900000000036,0,1543.52443308068,0,0
232.925000000036,0,1543.52443308068,0,0
232.950000000036,0,1543.52443308068,0,0
232.975000000036,0,1543.52443308068,0,0
233.000000000036,0,1543.52443308068,0,0
233.025000000036,0,1543.52443308068,0,0
233.050000000036,0,1543.52443308068,0,0
233.075000000036,0,1543.52443308068,0,0
233.100000000036,0,1543.52443308068,0,0
233.125000000036,1,1543.60655808068,0,0
233.150000000036,0,1543.68868308068,0,0
233.175000000036,0,1543.68868308068,0,0
233.200000000036,0,1543.68868308068,0,0
233.225000000036,0,1543.68868308068,0,0
233.250000000036,0,1543.68868308068,0,0
233.275000000036,0,1543.68868308068,0,0
233.300000000036,0,1543.68868308068,0,0
233.325000000036,0,1543.68868308068,0,0
233.350000000036,0,1543.68868308068,0,0
233.375000000036,0,1543.68868308068,0,0
233.400000000036,0,1543.68868308068,0,0
233.425000000036,0,1543.68868308068,0,0
233.450000000036,0,1543.68868308068,0,0
233.475000000036,0,1543.68868308068,0,0
233.500000000036,0,1543.68868308068,0,0
233.525000000036,1,1543.77080808068,0,0
233.550000000036,0,1543.85293308068,0,0
233.575000000036,0,1543.85293308068,0,0
233.600000000036,0,1543.85293308068,0,0
233.625000000036,1,1543.93505808068,0,0
233.650000000036,0,1544.01718308068,0,0
233.675000000036,0,1544.01718308068,0,0
233.700000000036,0,1544.01718308068,0,0
233.725000000036,0,1544.01718308068,0,0
233.750000000036,0,1544.01718308068,0,0
233.775000000036,0,1544.01718308068,0,0
233.800000000036,0,1544.01718308068,0,0
233.825000000036,0,1544.01718308068,0,0
233.850000000036,0,1544.01718308068,0,0
233.875000000036,0,1544.01718308068,0,0
233.900000000036,0,1544.01718308068,0,0
233.925000000036,0,1544.01718308068,0,0

233.950000000036,0,1544.01718308068,0,0
233.975000000036,1,1544.09930808068,0,0
234.000000000036,0,1544.18143308068,0,0
234.025000000036,0,1544.18143308068,0,0
234.050000000036,0,1544.18143308068,0,0
234.075000000036,0,1544.18143308068,0,0
234.100000000036,1,1544.26355808068,0,0
234.125000000036,0,1544.34568308068,0,0
234.150000000036,0,1544.34568308068,0,0
234.175000000036,0,1544.34568308068,0,0
234.200000000036,0,1544.34568308068,0,0
234.225000000036,0,1544.34568308068,0,0
234.250000000036,0,1544.34568308068,0,0
234.275000000036,0,1544.34568308068,0,0
234.300000000036,0,1544.34568308068,0,0
234.325000000036,0,1544.34568308068,0,0
234.350000000036,0,1544.34568308068,0,0
234.375000000036,0,1544.34568308068,0,0
234.400000000036,0,1544.34568308068,0,0
234.425000000036,1,1544.42780808068,0,0
234.450000000036,3,1544.65217775325,0,0
234.475000000036,1,1544.87654742582,0,0
234.500000000036,1,1545.04079742582,0,0
234.525000000036,0,1545.12292242582,0,0
234.550000000036,0,1545.12292242582,0,0
234.575000000036,0,1545.12292242582,0,0
234.600000000036,0,1545.12292242582,0,0
234.625000000036,0,1545.12292242582,0,0
234.650000000036,2,1545.23906471463,0,0
234.675000000036,1,1545.43733200344,0,0
234.700000000036,0,1545.51945700344,0,0
234.725000000036,0,1545.51945700344,0,0
234.750000000036,1,1545.60158200344,0,0
234.775000000036,0,1545.68370700344,0,0
234.800000000036,1,1545.76583200344,0,0
234.825000000036,6,1546.04912134857,0,0
234.850000000036,0,1546.2502856937,0,0
234.875000000036,1,1546.3324106937,0,0
234.900000000036,0,1546.4145356937,0,0
234.925000000036,0,1546.4145356937,0,0
234.950000000036,0,1546.4145356937,0,0
234.975000000036,0,1546.4145356937,0,0
235.000000000036,0,1546.4145356937,0,0
235.025000000036,0,1546.4145356937,0,0
235.050000000036,0,1546.4145356937,0,0
235.075000000036,0,1546.4145356937,0,0
235.100000000036,0,1546.4145356937,0,0
235.125000000036,0,1546.4145356937,0,0
235.150000000036,0,1546.4145356937,0,0
235.175000000036,0,1546.4145356937,0,0
235.200000000036,0,1546.4145356937,0,0
235.225000000036,0,1546.4145356937,0,0

235.250000000036,0,1546.4145356937,0,0
235.275000000036,0,1546.4145356937,0,0
235.300000000036,0,1546.4145356937,0,0
235.325000000036,0,1546.4145356937,0,0
235.350000000036,1,1546.4966606937,0,0
235.375000000036,1,1546.6609106937,0,0
235.400000000036,0,1546.7430356937,0,0
235.425000000036,0,1546.7430356937,0,0
235.450000000036,0,1546.7430356937,0,0
235.475000000036,0,1546.7430356937,0,0
235.500000000036,0,1546.7430356937,0,0
235.525000000036,0,1546.7430356937,0,0
235.550000000036,0,1546.7430356937,0,0
235.575000000036,0,1546.7430356937,0,0
235.600000000036,0,1546.7430356937,0,0
235.625000000036,0,1546.7430356937,0,0
235.650000000036,0,1546.7430356937,0,0
235.675000000036,0,1546.7430356937,0,0
235.700000000036,0,1546.7430356937,0,0
235.725000000036,0,1546.7430356937,0,0
235.750000000036,0,1546.7430356937,0,0
235.775000000036,0,1546.7430356937,0,0
235.800000000036,0,1546.7430356937,0,0
235.825000000036,0,1546.7430356937,0,0
235.850000000036,0,1546.7430356937,0,0
235.875000000036,0,1546.7430356937,0,0
235.900000000037,0,1546.7430356937,0,0
235.925000000037,0,1546.7430356937,0,0
235.950000000037,0,1546.7430356937,0,0
235.975000000037,0,1546.7430356937,0,0
236.000000000037,0,1546.7430356937,0,0
236.025000000037,0,1546.7430356937,0,0
236.050000000037,0,1546.7430356937,0,0
236.075000000037,0,1546.7430356937,0,0
236.100000000037,0,1546.7430356937,0,0
236.125000000037,0,1546.7430356937,0,0
236.150000000037,0,1546.7430356937,0,0
236.175000000037,0,1546.7430356937,0,0
236.200000000037,0,1546.7430356937,0,0
236.225000000037,1,1546.8251606937,0,0
236.250000000037,4,1547.0715356937,0,0
236.275000000037,7,1547.45306802012,0,0
236.300000000037,13,1547.96645624504,48.7377057704474,1
236.325000000037,17,1548.60117219304,48.7377057704474,1
236.350000000037,20,1549.30705640784,48.7377057704474,1
236.375000000037,22,1550.05953096742,48.7377057704474,1
236.400000000037,22,1550.82993175598,48.7377057704474,1
236.425000000037,23,1551.6089898141,48.7377057704474,1
236.450000000037,20,1552.37012164326,48.7377057704474,1
236.475000000037,18,1553.08582267499,48.7377057704474,1
236.500000000037,17,1553.77285959093,48.7377057704474,1
236.525000000037,17,1554.45007968993,48.7377057704474,1

236.550000000037,19,1555.14666431518,48.7377057704474,1
236.575000000037,20,1555.87191305622,48.7377057704474,1
236.600000000037,18,1556.58761408795,48.7377057704474,1
236.625000000037,14,1557.24332456727,48.7377057704474,1
236.650000000037,12,1557.8350975253,48.7377057704474,1
236.675000000037,13,1558.41569276894,48.7377057704474,1
236.700000000037,16,1559.04029866744,48.7377057704474,1
236.725000000037,21,1559.74514269639,48.7377057704474,1
236.750000000037,33,1560.59325893269,48.7377057704474,1
236.775000000037,55,1561.67408644079,48.7377057704474,1
236.800000000037,72,1562.9799954744,48.7377057704474,1
236.825000000037,90,1564.45595536578,48.7377057704474,1
236.850000000037,109,1566.09247169635,48.7377057704474,1
236.875000000037,115,1567.83057437823,48.7377057704474,1
236.900000000037,124,1569.62577218465,48.7377057704474,1
236.925000000037,135,1571.49448375317,48.7377057704474,1
236.950000000037,134,1573.39935563073,48.7377057704474,1
236.975000000037,125,1575.26820664964,48.7377057704474,1
237.000000000037,110,1577.04772632946,48.7377057704474,1
237.025000000037,102,1578.73848243908,48.7377057704474,1
237.050000000037,94,1580.36413569872,48.7377057704474,1
237.075000000037,99,1581.97750054803,48.7377057704474,1
237.100000000037,95,1583.59508946632,48.7377057704474,1
237.125000000037,94,1585.19177636847,48.7377057704474,1
237.150000000037,92,1586.77572311275,48.7377057704474,1
237.175000000037,89,1588.33820414092,48.7377057704474,1
237.200000000037,88,1589.88337062995,48.7377057704474,1
237.225000000037,85,1591.41092650705,48.7377057704474,1
237.250000000037,79,1592.89802456213,48.7377057704474,1
237.275000000037,72,1594.32482126151,48.7377057704474,1
237.300000000037,66,1595.68886164835,48.7377057704474,1
237.325000000037,64,1597.01304830233,48.7377057704474,1
237.350000000037,63,1598.32189528159,48.7377057704474,1
237.375000000037,69,1599.6559237456,48.7377057704474,1
237.400000000037,70,1601.02521227713,48.7377057704474,1
237.425000000037,72,1602.40917305678,48.7377057704474,1
237.450000000037,71,1603.79802433977,48.7377057704474,1
237.475000000037,73,1605.19169819747,48.7377057704474,1
237.500000000037,70,1606.58048155185,48.7377057704474,1
237.525000000037,74,1607.97405456119,48.7377057704474,1
237.550000000037,73,1609.38219683133,48.7377057704474,1
237.575000000037,70,1610.77098018571,48.7377057704474,1
237.600000000037,76,1612.17403638398,48.7377057704474,1
237.625000000037,71,1613.58198308558,48.7377057704474,1
237.650000000037,75,1614.98520399856,48.7377057704474,1
237.675000000037,74,1616.40289332397,48.7377057704474,1
237.700000000037,77,1617.83000323684,48.7377057704474,1
237.725000000037,77,1619.27129113747,48.7377057704474,1
237.750000000037,73,1620.69361139537,48.7377057704474,1
237.775000000037,71,1622.08728525307,48.7377057704474,1
237.800000000037,70,1623.46638984999,48.7377057704474,1
237.825000000037,65,1624.81560981436,48.7377057704474,1

237.850000000037,60,1626.11386024655,48.7377057704474,1
237.875000000037,56,1627.36456498694,48.7377057704474,1
237.900000000037,57,1628.59916236572,48.7377057704474,1
237.925000000037,56,1629.83375974449,48.7377057704474,1
237.950000000037,59,1631.07914106481,48.7377057704474,1
237.975000000037,53,1632.30783418401,48.7377057704474,1
238.000000000037,50,1633.48642465272,48.7377057704474,1
238.025000000037,48,1634.63611478706,48.7377057704474,1
238.050000000037,42,1635.7373243072,48.7377057704474,1
238.075000000037,42,1636.80178596691,48.7377057704474,1
238.100000000037,39,1637.84688725739,48.7377057704474,1
238.125000000037,36,1638.85250771801,48.7377057704474,1
238.150000000037,28,1639.77982237085,48.7377057704474,1
238.175000000037,21,1640.59073105264,48.7377057704474,1
238.200000000037,17,1641.30568513109,48.7377057704474,1
238.225000000037,3,1641.78653985317,48.7377057704474,1
238.250000000037,2,1642.04492681455,0,0
238.275000000037,0,1642.16106910336,0,0
238.300000000037,0,1642.16106910336,0,0
238.325000000037,0,1642.16106910336,0,0
238.350000000037,0,1642.16106910336,0,0
238.375000000037,0,1642.16106910336,0,0
238.400000000037,0,1642.16106910336,0,0
238.425000000037,0,1642.16106910336,0,0
238.450000000037,0,1642.16106910336,0,0
238.475000000037,3,1642.30331377593,0,0
238.500000000037,0,1642.4455584485,0,0
238.525000000037,0,1642.4455584485,0,0
238.550000000037,0,1642.4455584485,0,0
238.575000000037,7,1642.66284077492,0,0
238.600000000037,0,1642.88012310134,0,0
238.625000000037,1,1642.96224810134,0,0
238.650000000037,1,1643.12649810134,0,0
238.675000000037,2,1643.32476539015,0,0
238.700000000037,1,1643.52303267896,0,0
238.725000000037,1,1643.68728267896,0,0
238.750000000037,2,1643.88554996777,0,0
238.775000000037,1,1644.08381725658,0,0
238.800000000037,1,1644.24806725658,0,0
238.825000000037,0,1644.33019225658,0,0
238.850000000037,0,1644.33019225658,0,0
238.875000000037,0,1644.33019225658,0,0
238.900000000037,0,1644.33019225658,0,0
238.925000000037,0,1644.33019225658,0,0
238.950000000037,0,1644.33019225658,0,0
238.975000000037,0,1644.33019225658,0,0
239.000000000037,0,1644.33019225658,0,0
239.025000000037,0,1644.33019225658,0,0
239.050000000037,0,1644.33019225658,0,0
239.075000000037,0,1644.33019225658,0,0
239.100000000037,0,1644.33019225658,0,0
239.125000000037,0,1644.33019225658,0,0

239.150000000037,0,1644.33019225658,0,0
239.175000000037,0,1644.33019225658,0,0
239.200000000037,0,1644.33019225658,0,0
239.225000000037,0,1644.33019225658,0,0
239.250000000037,0,1644.33019225658,0,0
239.275000000037,0,1644.33019225658,0,0
239.300000000037,0,1644.33019225658,0,0
239.325000000037,0,1644.33019225658,0,0
239.350000000037,0,1644.33019225658,0,0
239.375000000037,0,1644.33019225658,0,0
239.400000000037,0,1644.33019225658,0,0
239.425000000037,0,1644.33019225658,0,0
239.450000000037,0,1644.33019225658,0,0
239.475000000037,0,1644.33019225658,0,0
239.500000000037,0,1644.33019225658,0,0
239.525000000037,0,1644.33019225658,0,0
239.550000000037,0,1644.33019225658,0,0
239.575000000037,0,1644.33019225658,0,0
239.600000000037,0,1644.33019225658,0,0
239.625000000037,0,1644.33019225658,0,0
239.650000000037,1,1644.41231725658,0,0
239.675000000037,0,1644.49444225658,0,0
239.700000000037,0,1644.49444225658,0,0
239.725000000037,1,1644.57656725658,0,0
239.750000000037,0,1644.65869225658,0,0
239.775000000037,0,1644.65869225658,0,0
239.800000000037,0,1644.65869225658,0,0
239.825000000037,0,1644.65869225658,0,0
239.850000000037,0,1644.65869225658,0,0
239.875000000037,0,1644.65869225658,0,0
239.900000000037,0,1644.65869225658,0,0
239.925000000037,0,1644.65869225658,0,0
239.950000000037,0,1644.65869225658,0,0
239.975000000037,0,1644.65869225658,0,0
240.000000000037,0,1644.65869225658,0,0
240.025000000037,0,1644.65869225658,0,0
240.050000000037,0,1644.65869225658,0,0
240.075000000037,0,1644.65869225658,0,0
240.100000000037,0,1644.65869225658,0,0
240.125000000037,0,1644.65869225658,0,0
240.150000000037,0,1644.65869225658,0,0
240.175000000037,0,1644.65869225658,0,0
240.200000000037,0,1644.65869225658,0,0
240.225000000037,0,1644.65869225658,0,0
240.250000000037,0,1644.65869225658,0,0
240.275000000037,0,1644.65869225658,0,0
240.300000000038,0,1644.65869225658,0,0
240.325000000038,0,1644.65869225658,0,0
240.350000000038,0,1644.65869225658,0,0
240.375000000038,1,1644.74081725658,0,0
240.400000000038,0,1644.82294225658,0,0
240.425000000038,0,1644.82294225658,0,0

240.450000000038,0,1644.82294225658,0,0
