# Supplementary material for: Electronic Cigarette Topography in the Natural Environment
Source: PLoS One. 2015 Jun 8;10(6):e0129296. doi: 10.1371/journal.pone.0129296 (PMC4460076; doi:10.1371/journal.pone.0129296)
Supplement: S4 File — This file contains the data used to generate Fig 5, Panel B, describing Subject 7, Puffing Session 16. (PDF) [file pone.0129296.s004.pdf]

RelativeTime,CleanData,AccumulatedVolume,MeanPuffFlowRate,PuffFlag
0,1,0.16425,0,0
0.025,1,0.3285,0,0
0.05,1,0.49275,0,0
0.075,1,0.657,0,0
0.1,1,0.82125,0,0
0.125,1,0.9855,0,0
0.15,1,1.14975,0,0
0.175,1,1.314,0,0
0.2,1,1.47825,0,0
0.225,1,1.6425,0,0
0.25,1,1.80675,0,0
0.275,1,1.971,0,0
0.3,1,2.13525,0,0
0.325,1,2.2995,0,0
0.35,1,2.46375,0,0
0.375,1,2.628,0,0
0.4,1,2.79225,0,0
0.425,0,2.874375,0,0
0.45,1,2.9565,0,0
0.475,1,3.12075,0,0
0.5,1,3.285,0,0
0.525,1,3.44925,0,0
0.55,0,3.531375,0,0
0.575,1,3.6135,0,0
0.6,1,3.77775,0,0
0.625,0,3.859875,0,0
0.65,1,3.942,0,0
0.675,1,4.10625,0,0
0.7,0,4.188375,0,0
0.725,1,4.2705,0,0
0.75,0,4.352625,0,0
0.775,1,4.43475,0,0
0.8,1,4.599,0,0
0.825,1,4.76325,0,0
0.85,0,4.845375,0,0
0.875,1,4.9275,0,0
0.9,1,5.09175,0,0
0.925,1,5.256,0,0
0.950000000000001,1,5.42025,0,0
0.975000000000001,2,5.61851728880989,0,0
1,5,5.91829666027195,0,0
1.025,8,6.3342183205439,0,0
1.05,11,6.83888070907162,50.4659034191732,1
1.075,16,7.43975851997956,50.4659034191732,1
1.1,21,8.1446025489278,50.4659034191732,1
1.125,25,8.93157157787605,50.4659034191732,1
1.15,29,9.78445323766197,50.4659034191732,1
1.175,31,10.6839625457453,50.4659034191732,1
1.2,33,11.6129874013897,50.4659034191732,1
1.225,36,12.5775096087366,50.4659034191732,1

1.25,39,13.5831300693551,50.4659034191732,1
1.275,42,14.6282313598303,50.4659034191732,1
1.3,44,15.7052178115029,50.4659034191732,1
1.325,48,16.8189521236052,50.4659034191732,1
1.35,50,17.968642257941,50.4659034191732,1
1.375,53,19.1472327266476,50.4659034191732,1
1.4,57,20.3651419043014,50.4659034191732,1
1.425,55,21.5942273580507,50.4659034191732,1
1.45,59,22.8340967533472,50.4659034191732,1
1.475,60,24.1010483625054,50.4659034191732,1
1.5,60,25.3733233917346,50.4659034191732,1
1.525,64,26.6664609063491,50.4659034191732,1
1.55,63,27.9753078856127,50.4659034191732,1
1.575,63,29.2790018441397,50.4659034191732,1
1.6,67,30.6030709197933,50.4659034191732,1
1.625,64,31.9322930161833,50.4659034191732,1
1.65,63,33.2411399954469,50.4659034191732,1
1.675,62,34.5396398713636,50.4659034191732,1
1.7,67,35.8585148644069,50.4659034191732,1
1.725,64,37.1877369607969,50.4659034191732,1
1.75,68,38.5219570598046,50.4659034191732,1
1.775,67,39.8713992552023,50.4659034191732,1
1.8,65,41.2057342691714,50.4659034191732,1
1.825,69,42.5500286714925,50.4659034191732,1
1.85,67,43.9044322526247,50.4659034191732,1
1.875,66,45.2438410029955,50.4659034191732,1
1.9,69,46.5932091417183,50.4659034191732,1
1.925,66,47.9425772804412,50.4659034191732,1
1.95,65,49.271876852001,50.4659034191732,1
1.975,64,50.59098976958,50.4659034191732,1
2,65,51.910102687159,50.4659034191732,1
2.025,63,53.2240625840016,50.4659034191732,1
2.05,66,54.5430962172458,50.4659034191732,1
2.075,65,55.8723957888056,50.4659034191732,1
2.1,71,57.2265062565067,50.4659034191732,1
2.125,65,58.5806167242078,50.4659034191732,1
2.15,69,59.924911126529,50.4659034191732,1
2.175,69,61.2892740960133,50.4659034191732,1
2.2,71,62.6634531308775,50.4659034191732,1
2.225,71,64.0474482311218,50.4659034191732,1
2.25,68,65.4166658802516,50.4659034191732,1
2.275,69,66.7760674640014,50.4659034191732,1
2.3,63,68.1100959280071,50.4659034191732,1
2.325,67,69.4341650036607,50.4659034191732,1
2.35,67,70.7786091964407,50.4659034191732,1
2.375,67,72.1230533892208,50.4659034191732,1
2.4,69,73.4774569703529,50.4659034191732,1
2.425,69,74.8418199398372,50.4659034191732,1
2.45,73,76.2256777321636,50.4659034191732,1
2.475,71,77.6193515898699,50.4659034191732,1
2.5,68,78.9885692389997,50.4659034191732,1
2.525,70,80.3528963847985,50.4659034191732,1

2.54999999999999,70,81.7271104783807,50.4659034191732,1
2.57499999999999,66,83.0814041791526,50.4659034191732,1
2.59999999999999,67,84.4208129295233,50.4659034191732,1
2.62499999999999,70,85.7801420727044,50.4659034191732,1
2.64999999999999,67,87.1394712158856,50.4659034191732,1
2.67499999999999,67,88.4839154086656,50.4659034191732,1
2.69999999999999,69,89.8383189897978,50.4659034191732,1
2.72499999999999,69,91.2026819592821,50.4659034191732,1
2.74999999999999,70,92.5719704908153,50.4659034191732,1
2.77499999999999,71,93.9510750877286,50.4659034191732,1
2.79999999999999,75,95.3542960007086,50.4659034191732,1
2.82499999999999,75,96.7767427264246,50.4659034191732,1
2.84999999999999,75,98.1991894521405,50.4659034191732,1
2.87499999999999,76,99.626361966475,50.4659034191732,1
2.89999999999999,79,101.072254084474,50.4659034191732,1
2.92499999999999,76,102.518146202472,50.4659034191732,1
2.94999999999999,78,103.959403715096,50.4659034191732,1
2.97499999999999,77,105.405356026558,50.4659034191732,1
2.99999999999999,73,106.827676284457,50.4659034191732,1
3.02499999999999,75,108.240575954899,50.4659034191732,1
3.04999999999999,76,109.667748469233,50.4659034191732,1
3.07499999999999,72,111.080551353569,50.4659034191732,1
3.09999999999999,71,112.469402636551,50.4659034191732,1
3.12499999999999,68,113.83862028568,50.4659034191732,1
3.14999999999999,72,115.212694117547,50.4659034191732,1
3.17499999999999,68,116.586767949414,50.4659034191732,1
3.19999999999999,68,117.94120814743,50.4659034191732,1
3.22499999999999,71,119.31042579656,50.4659034191732,1
3.24999999999999,70,120.689530393473,50.4659034191732,1
3.27499999999999,69,122.058818925006,50.4659034191732,1
3.29999999999999,74,123.447466372304,50.4659034191732,1
3.32499999999999,78,124.879240696007,50.4659034191732,1
3.34999999999999,73,126.306225364739,50.4659034191732,1
3.37499999999999,71,127.699899222445,50.4659034191732,1
3.39999999999999,73,129.093573080151,50.4659034191732,1
3.42499999999999,72,130.492103120595,50.4659034191732,1
3.44999999999999,71,131.880954403576,50.4659034191732,1
3.47499999999999,70,133.260059000489,50.4659034191732,1
3.49999999999999,61,134.588582801914,50.4659034191732,1
3.52499999999999,61,135.871416311182,50.4659034191732,1
3.54999999999999,64,137.169833065816,50.4659034191732,1
3.57499999999999,63,138.478680045079,50.4659034191732,1
3.59999999999999,62,139.777179920996,50.4659034191732,1
3.62499999999999,65,141.085945735228,50.4659034191732,1
3.64999999999999,62,142.394711549461,50.4659034191732,1
3.67499999999999,67,143.713586542504,50.4659034191732,1
3.69999999999999,65,145.047921556473,50.4659034191732,1
3.72499999999999,59,146.340848568596,50.4659034191732,1
3.74999999999999,63,147.623509642403,50.4659034191732,1
3.77499999999999,57,148.895386774663,50.4659034191732,1
3.79999999999999,55,150.124472228412,50.4659034191732,1
3.82499999999999,64,151.390527529165,50.4659034191732,1

3.84999999999999,56,152.662094754943,50.4659034191732,1
3.87499999999999,64,153.93366198072,50.4659034191732,1
3.89999999999999,64,155.24766198072,50.4659034191732,1
3.92499999999999,60,156.540799495335,50.4659034191732,1
3.94999999999999,61,157.818353764583,50.4659034191732,1
3.97499999999999,61,159.101187273851,50.4659034191732,1
3.99999999999999,63,160.394451007748,50.4659034191732,1
4.02499999999999,64,161.703297987012,50.4659034191732,1
4.04999999999999,72,163.057151719871,50.4659034191732,1
4.07499999999999,66,164.421192106711,50.4659034191732,1
4.09999999999999,69,165.770560245434,50.4659034191732,1
4.12499999999999,68,167.129961829184,50.4659034191732,1
4.14999999999999,67,168.479404024582,50.4659034191732,1
4.17499999999999,65,169.813739038551,50.4659034191732,1
4.19999999999999,69,171.158033440872,50.4659034191732,1
4.22499999999999,63,172.492061904878,50.4659034191732,1
4.24999999999999,65,173.80602180172,50.4659034191732,1
4.27499999999999,71,175.160132269421,50.4659034191732,1
4.29999999999999,66,176.519316473524,50.4659034191732,1
4.32499999999999,67,177.858725223895,50.4659034191732,1
4.34999999999999,67,179.203169416675,50.4659034191732,1
4.37499999999999,68,180.552611612073,50.4659034191732,1
4.4,71,181.921829261203,50.4659034191732,1
4.425,67,183.286048907715,50.4659034191732,1
4.45,66,184.625457658085,50.4659034191732,1
4.475,66,185.959830966047,50.4659034191732,1
4.5,66,187.294204274008,50.4659034191732,1
4.525,65,188.623503845568,50.4659034191732,1
4.55,70,189.972723809938,50.4659034191732,1
4.575,70,191.34693790352,50.4659034191732,1
4.6,71,192.726042500434,50.4659034191732,1
4.625,72,194.114893783415,50.4659034191732,1
4.65,68,195.488967615282,50.4659034191732,1
4.675,70,196.853294761081,50.4659034191732,1
4.7,64,198.197401807872,50.4659034191732,1
4.725,67,199.526623904262,50.4659034191732,1
4.75,69,200.881027485394,50.4659034191732,1
4.775,68,202.240429069144,50.4659034191732,1
4.8,68,203.594869267159,50.4659034191732,1
4.825,67,204.944311462557,50.4659034191732,1
4.85,64,206.273533558947,50.4659034191732,1
4.875,63,207.582380538211,50.4659034191732,1
4.9,62,208.880880414128,50.4659034191732,1
4.925,66,210.194719964761,50.4659034191732,1
4.95,64,211.518906618742,50.4659034191732,1
4.975,67,212.848128715132,50.4659034191732,1
5,65,214.182463729101,50.4659034191732,1
5.025,63,215.496423625944,50.4659034191732,1
5.05,64,216.805270605207,50.4659034191732,1
5.075,65,218.124383522786,50.4659034191732,1
5.10000000000001,64,219.443496440365,50.4659034191732,1
5.12500000000001,66,220.767683094346,50.4659034191732,1

5.15000000000001,66,222.102056402308,50.4659034191732,1
5.17500000000001,68,223.446463155296,50.4659034191732,1
5.20000000000001,65,224.785796171883,50.4659034191732,1
5.22500000000001,63,226.099756068725,50.4659034191732,1
5.25000000000001,67,227.423825144379,50.4659034191732,1
5.27500000000001,66,228.76323389475,50.4659034191732,1
5.30000000000001,71,230.122418098852,50.4659034191732,1
5.32500000000001,65,231.476528566554,50.4659034191732,1
5.35000000000001,63,232.790488463396,50.4659034191732,1
5.37500000000001,60,234.078472957274,50.4659034191732,1
5.40000000000001,62,235.361263368542,50.4659034191732,1
5.42500000000001,61,236.649333019829,50.4659034191732,1
5.45000000000001,59,237.921563869007,50.4659034191732,1
5.47500000000001,66,239.219564617531,50.4659034191732,1
5.50000000000001,61,240.528168026146,50.4659034191732,1
5.52500000000001,62,241.816237677433,50.4659034191732,1
5.55000000000001,60,243.0990280887,50.4659034191732,1
5.57500000000001,59,244.365979697859,50.4659034191732,1
5.60000000000001,59,245.627607886946,50.4659034191732,1
5.62500000000001,57,246.878452134487,50.4659034191732,1
5.65000000000001,59,248.129296382027,50.4659034191732,1
5.67500000000001,58,249.38555584289,50.4659034191732,1
5.70000000000001,55,250.620056509961,50.4659034191732,1
5.72500000000001,55,251.838167111467,50.4659034191732,1
5.75000000000001,53,253.045101436877,50.4659034191732,1
5.77500000000001,53,254.240859486191,50.4659034191732,1
5.80000000000001,52,255.430950307843,50.4659034191732,1
5.82500000000002,55,256.632217405591,50.4659034191732,1
5.85000000000002,54,257.844765741722,50.4659034191732,1
5.87500000000002,54,259.051751812478,50.4659034191732,1
5.90000000000002,51,260.241734657551,50.4659034191732,1
5.92500000000002,51,261.414714276939,50.4659034191732,1
5.95000000000002,48,262.570182776919,50.4659034191732,1
5.97500000000002,41,263.665018045205,50.4659034191732,1
6.00000000000002,36,264.683624623204,50.4659034191732,1
6.02500000000002,38,265.682627123198,50.4659034191732,1
6.05000000000002,35,266.674737675379,50.4659034191732,1
6.07500000000002,35,267.646453779753,50.4659034191732,1
6.10000000000002,37,268.63185870474,50.4659034191732,1
6.12500000000002,43,269.66993521635,50.4659034191732,1
6.15000000000002,45,270.759376103114,50.4659034191732,1
6.17500000000002,44,271.855042972887,50.4659034191732,1
6.20000000000002,46,272.956797444567,50.4659034191732,1
6.22500000000002,44,274.058551916247,50.4659034191732,1
6.25000000000002,48,275.172286228349,50.4659034191732,1
6.27500000000002,44,276.286020540451,50.4659034191732,1
6.30000000000002,48,277.399754852553,50.4659034191732,1
6.32500000000002,47,278.531754176898,50.4659034191732,1
6.35000000000002,46,279.65177366082,50.4659034191732,1
6.37500000000002,51,280.795262320378,50.4659034191732,1
6.40000000000002,48,281.950730820359,50.4659034191732,1
6.42500000000002,56,283.134276736423,50.4659034191732,1

```
6.45000000000002,54,284.352336997578,50.4659034191732,1
6.47500000000002,51,285.542319842651,50.4659034191732,1
6.50000000000002,57,286.748839805341,50.4659034191732,1
6.52500000000003,55,287.977925259091,50.4659034191732,1
6.55000000000003,51,289.173470369538,50.4659034191732,1
6.57500000000003,52,290.352171976227,50.4659034191732,1
6.60000000000003,53,291.542262797879,50.4659034191732,1
6.62500000000003,51,292.72663163223,50.4659034191732,1
6.65000000000003,52,293.905333238919,50.4659034191732,1
6.67500000000003,54,295.101038071292,50.4659034191732,1
6.70000000000003,55,296.313586407423,50.4659034191732,1
6.72500000000003,55,297.531697008929,50.4659034191732,1
6.75000000000003,56,298.755319535459,50.4659034191732,1
6.77500000000003,58,299.995332127556,50.4659034191732,1
6.80000000000003,55,301.229832794628,50.4659034191732,1
6.82500000000003,52,302.431099892375,50.4659034191732,1
6.85000000000003,51,303.609801499064,50.4659034191732,1
6.87500000000003,54,304.799784344137,50.4659034191732,1
6.90000000000003,50,305.983988823564,50.4659034191732,1
6.92500000000003,54,307.168193302992,50.4659034191732,1
6.95000000000003,49,308.34656133837,50.4659034191732,1
6.97500000000003,52,309.513648135365,50.4659034191732,1
7.00000000000003,52,310.698071729355,50.4659034191732,1
7.02500000000003,51,311.876773336044,50.4659034191732,1
7.05000000000003,55,313.072318446491,50.4659034191732,1
7.07500000000003,52,314.273585544238,50.4659034191732,1
7.10000000000003,55,315.474852641986,50.4659034191732,1
7.12500000000003,61,316.725324697373,50.4659034191732,1
7.15000000000003,52,317.958953249002,50.4659034191732,1
7.17500000000003,56,319.165732271774,50.4659034191732,1
7.20000000000003,58,320.405744863871,50.4659034191732,1
7.22500000000004,62,321.677843126843,50.4659034191732,1
7.25000000000004,55,322.933551324249,50.4659034191732,1
7.27500000000004,58,324.168051991321,50.4659034191732,1
7.30000000000004,60,325.429634872255,50.4659034191732,1
7.32500000000004,58,326.691217753188,50.4659034191732,1
7.35000000000004,58,327.942108485826,50.4659034191732,1
7.37500000000004,61,329.208970606779,50.4659034191732,1
7.40000000000004,66,330.517574015394,50.4659034191732,1
7.42500000000004,64,331.841760669375,50.4659034191732,1
7.45000000000004,57,333.118790822371,50.4659034191732,1
7.47500000000004,66,334.406007629348,50.4659034191732,1
7.50000000000004,56,335.687761509107,50.4659034191732,1
7.52500000000004,59,336.933142829428,50.4659034191732,1
7.55000000000004,58,338.189402290291,50.4659034191732,1
7.57500000000004,54,339.418340691988,50.4659034191732,1
7.60000000000004,55,340.630889028119,50.4659034191732,1
7.62500000000004,50,341.820655772921,50.4659034191732,1
7.65000000000004,48,342.970345907257,50.4659034191732,1
7.67500000000004,50,344.120036041593,50.4659034191732,1
7.70000000000004,54,345.304240521021,50.4659034191732,1
7.72500000000004,51,346.494223366093,50.4659034191732,1
```

7.75000000000004,47,347.643733809845,50.4659034191732,1
7.77500000000004,38,348.713006943897,50.4659034191732,1
7.80000000000004,30,349.669076594242,50.4659034191732,1
7.82500000000004,25,350.529518744593,50.4659034191732,1
7.85000000000004,22,351.325344138868,50.4659034191732,1
7.87500000000004,15,352.028613290451,50.4659034191732,1
7.90000000000004,5,352.530319130411,50.4659034191732,1
7.92500000000005,1,352.796081213063,0,0
7.95000000000005,2,352.994348501873,0,0
7.97500000000005,1,353.192615790683,0,0
8.00000000000005,0,353.274740790683,0,0
8.02500000000005,0,353.274740790683,0,0
8.05000000000005,1,353.356865790683,0,0
8.07500000000005,0,353.438990790683,0,0
8.10000000000005,1,353.521115790683,0,0
8.12500000000005,1,353.685365790683,0,0
8.15000000000005,0,353.767490790683,0,0
8.17500000000005,2,353.883633079492,0,0
8.20000000000005,2,354.115917657112,0,0
8.22500000000005,0,354.232059945922,0,0
8.25000000000005,2,354.348202234732,0,0
8.27500000000005,1,354.546469523542,0,0
8.30000000000005,1,354.710719523542,0,0
8.32500000000005,0,354.792844523542,0,0
8.35000000000005,0,354.792844523542,0,0
8.37500000000005,1,354.874969523542,0,0
8.40000000000005,0,354.957094523542,0,0
8.42500000000005,0,354.957094523542,0,0
8.45000000000005,1,355.039219523542,0,0
8.47500000000005,0,355.121344523542,0,0
8.50000000000005,1,355.203469523542,0,0
8.52500000000005,0,355.285594523542,0,0
8.55000000000005,0,355.285594523542,0,0
8.57500000000005,0,355.285594523542,0,0
8.60000000000005,0,355.285594523542,0,0
8.62500000000006,0,355.285594523542,0,0
8.65000000000006,1,355.367719523542,0,0
8.67500000000006,0,355.449844523542,0,0
8.70000000000006,0,355.449844523542,0,0
8.72500000000006,1,355.531969523542,0,0
8.75000000000006,0,355.614094523542,0,0
8.77500000000006,0,355.614094523542,0,0
8.80000000000006,1,355.696219523542,0,0
8.82500000000006,1,355.860469523542,0,0
8.85000000000006,1,356.024719523542,0,0
8.87500000000006,1,356.188969523542,0,0
8.90000000000006,1,356.353219523542,0,0
8.92500000000006,0,356.435344523542,0,0
8.95000000000006,0,356.435344523542,0,0
8.97500000000006,0,356.435344523542,0,0
9.00000000000006,0,356.435344523542,0,0
9.02500000000006,0,356.435344523542,0,0

9.05000000000006,0,356.435344523542,0,0
9.07500000000006,0,356.435344523542,0,0
9.10000000000006,0,356.435344523542,0,0
9.12500000000006,0,356.435344523542,0,0
9.15000000000006,0,356.435344523542,0,0
9.17500000000006,1,356.517469523542,0,0
9.20000000000006,1,356.681719523542,0,0
9.22500000000006,0,356.763844523542,0,0
9.25000000000006,1,356.845969523542,0,0
9.27500000000006,1,357.010219523542,0,0
9.30000000000006,1,357.174469523542,0,0
9.32500000000007,1,357.338719523542,0,0
9.35000000000007,0,357.420844523542,0,0
9.37500000000007,0,357.420844523542,0,0
9.40000000000007,1,357.502969523542,0,0
9.42500000000007,1,357.667219523542,0,0
9.45000000000007,0,357.749344523542,0,0
9.47500000000007,1,357.831469523542,0,0
9.50000000000007,1,357.995719523542,0,0
9.52500000000007,1,358.159969523542,0,0
9.55000000000007,1,358.324219523542,0,0
9.57500000000007,1,358.488469523542,0,0
9.60000000000007,1,358.652719523542,0,0
9.62500000000007,1,358.816969523542,0,0
9.65000000000007,1,358.981219523542,0,0
9.67500000000007,1,359.145469523542,0,0
9.70000000000007,1,359.309719523542,0,0
9.72500000000007,0,359.391844523542,0,0
9.75000000000007,0,359.391844523542,0,0
9.77500000000007,1,359.473969523542,0,0
9.80000000000007,1,359.638219523542,0,0
9.82500000000007,1,359.802469523542,0,0
9.85000000000007,3,360.026839196114,0,0
9.87500000000007,1,360.251208868685,0,0
9.90000000000007,1,360.415458868685,0,0
9.92500000000007,1,360.579708868685,0,0
9.95000000000007,1,360.743958868685,0,0
9.97500000000007,1,360.908208868685,0,0
10.0000000000001,1,361.072458868685,0,0
10.0250000000001,1,361.236708868685,0,0
10.0500000000001,1,361.400958868685,0,0
10.0750000000001,1,361.565208868685,0,0
10.1000000000001,1,361.729458868685,0,0
10.1250000000001,1,361.893708868685,0,0
10.1500000000001,1,362.057958868685,0,0
10.1750000000001,1,362.222208868685,0,0
10.2000000000001,1,362.386458868685,0,0
10.2250000000001,1,362.550708868685,0,0
10.2500000000001,1,362.714958868685,0,0
10.2750000000001,1,362.879208868685,0,0
10.3000000000001,1,363.043458868685,0,0
10.3250000000001,2,363.241726157495,0,0

10.3500000000001,1,363.439993446305,0,0
10.3750000000001,1,363.604243446305,0,0
10.4000000000001,1,363.768493446305,0,0
10.4250000000001,1,363.932743446305,0,0
10.4500000000001,1,364.096993446305,0,0
10.4750000000001,0,364.179118446305,0,0
10.5000000000001,1,364.261243446305,0,0
10.5250000000001,1,364.425493446305,0,0
10.5500000000001,1,364.589743446305,0,0
10.5750000000001,1,364.753993446305,0,0
10.6000000000001,1,364.918243446305,0,0
10.6250000000001,1,365.082493446305,0,0
10.6500000000001,1,365.246743446305,0,0
10.6750000000001,1,365.410993446305,0,0
10.7000000000001,1,365.575243446305,0,0
10.7250000000001,1,365.739493446304,0,0
10.7500000000001,1,365.903743446304,0,0
10.7750000000001,1,366.067993446304,0,0
10.8000000000001,1,366.232243446304,0,0
10.8250000000001,2,366.430510735114,0,0
10.8500000000001,1,366.628778023924,0,0
10.8750000000001,1,366.793028023924,0,0
10.9000000000001,1,366.957278023924,0,0
10.9250000000001,1,367.121528023924,0,0
10.9500000000001,1,367.285778023924,0,0
10.9750000000001,1,367.450028023924,0,0
11.0000000000001,1,367.614278023924,0,0
11.0250000000001,1,367.778528023924,0,0
11.0500000000001,1,367.942778023924,0,0
11.0750000000001,1,368.107028023924,0,0
11.1000000000001,1,368.271278023924,0,0
11.1250000000001,1,368.435528023924,0,0
11.1500000000001,0,368.517653023924,0,0
11.1750000000001,1,368.599778023924,0,0
11.2000000000001,1,368.764028023924,0,0
11.2250000000001,1,368.928278023924,0,0
11.2500000000001,1,369.092528023924,0,0
11.2750000000001,1,369.256778023924,0,0
11.3000000000001,0,369.338903023924,0,0
11.3250000000001,1,369.421028023924,0,0
11.3500000000001,6,369.70431736905,0,0
11.3750000000001,0,369.905481714176,0,0
11.4000000000001,5,370.089118796828,0,0
11.4250000000001,0,370.272755879481,0,0
11.4500000000001,0,370.272755879481,0,0
11.4750000000001,1,370.354880879481,0,0
11.5000000000001,0,370.437005879481,0,0
11.5250000000001,2,370.553148168291,0,0
11.5500000000001,1,370.7514154571,0,0
11.5750000000001,1,370.9156654571,0,0
11.6000000000001,1,371.0799154571,0,0
11.6250000000001,1,371.2441654571,0,0

```
11.6500000000001,3,371.468535129672,0,0
11.6750000000001,1,371.692904802244,0,0
11.7000000000001,0,371.775029802244,0,0
11.7250000000001,1,371.857154802244,0,0
11.7500000000001,0,371.939279802244,0,0
11.7750000000001,1,372.021404802244,0,0
11.8000000000001,2,372.219672091054,0,0
11.8250000000001,1,372.417939379863,0,0
11.8500000000001,1,372.582189379863,0,0
11.8750000000001,1,372.746439379863,0,0
11.9000000000001,1,372.910689379863,0,0
11.9250000000001,1,373.074939379863,0,0
11.9500000000001,1,373.239189379863,0,0
11.9750000000001,1,373.403439379863,0,0
12.0000000000001,1,373.567689379863,0,0
12.0250000000001,1,373.731939379863,0,0
12.0500000000001,1,373.896189379863,0,0
12.0750000000001,1,374.060439379863,0,0
12.1000000000001,1,374.224689379863,0,0
12.1250000000001,1,374.388939379863,0,0
12.1500000000001,1,374.553189379863,0,0
12.1750000000001,1,374.717439379863,0,0
12.2000000000001,1,374.881689379863,0,0
12.2250000000001,1,375.045939379863,0,0
12.2500000000001,1,375.210189379863,0,0
12.2750000000001,1,375.374439379863,0,0
12.3000000000001,1,375.538689379863,0,0
12.3250000000001,1,375.702939379863,0,0
12.3500000000001,1,375.867189379863,0,0
12.3750000000001,1,376.031439379863,0,0
12.4000000000001,1,376.195689379863,0,0
12.4250000000001,1,376.359939379863,0,0
12.4500000000001,1,376.524189379863,0,0
12.4750000000001,1,376.688439379863,0,0
12.5000000000001,1,376.852689379863,0,0
12.5250000000001,1,377.016939379863,0,0
12.5500000000001,1,377.181189379863,0,0
12.5750000000001,1,377.345439379863,0,0
12.6000000000001,1,377.509689379863,0,0
12.6250000000001,1,377.673939379863,0,0
12.6500000000001,1,377.838189379863,0,0
12.6750000000001,1,378.002439379863,0,0
12.7000000000001,1,378.166689379863,0,0
12.7250000000001,1,378.330939379863,0,0
12.7500000000001,0,378.413064379863,0,0
12.7750000000001,1,378.495189379863,0,0
12.8000000000001,1,378.659439379863,0,0
12.8250000000001,1,378.823689379863,0,0
12.8500000000001,1,378.987939379863,0,0
12.8750000000001,1,379.152189379863,0,0
12.9000000000001,1,379.316439379863,0,0
12.9250000000001,1,379.480689379863,0,0
```

12.9500000000001,1,379.644939379863,0,0
12.9750000000001,1,379.809189379863,0,0
13.0000000000001,1,379.973439379863,0,0
13.0250000000001,1,380.137689379863,0,0
13.0500000000001,1,380.301939379863,0,0
13.0750000000001,1,380.466189379863,0,0
13.1000000000001,1,380.630439379863,0,0
13.1250000000001,1,380.794689379863,0,0
13.1500000000001,1,380.958939379863,0,0
13.1750000000001,1,381.123189379863,0,0
13.2000000000001,1,381.287439379863,0,0
13.2250000000001,1,381.451689379863,0,0
13.2500000000001,1,381.615939379862,0,0
13.2750000000001,0,381.698064379863,0,0
13.3000000000001,1,381.780189379863,0,0
13.3250000000001,1,381.944439379863,0,0
13.3500000000001,1,382.108689379862,0,0
13.3750000000001,1,382.272939379862,0,0
13.4000000000001,1,382.437189379862,0,0
13.4250000000001,1,382.601439379862,0,0
13.4500000000001,1,382.765689379862,0,0
13.4750000000001,1,382.929939379862,0,0
13.5000000000001,1,383.094189379862,0,0
13.5250000000001,1,383.258439379862,0,0
13.5500000000001,1,383.422689379862,0,0
13.5750000000001,1,383.586939379862,0,0
13.6000000000001,1,383.751189379862,0,0
13.6250000000001,1,383.915439379862,0,0
13.6500000000001,1,384.079689379862,0,0
13.6750000000001,1,384.243939379862,0,0
13.7000000000001,1,384.408189379862,0,0
13.7250000000001,1,384.572439379862,0,0
13.7500000000001,3,384.796809052434,0,0
13.7750000000001,1,385.021178725005,0,0
13.8000000000001,1,385.185428725005,0,0
13.8250000000001,1,385.349678725005,0,0
13.8500000000001,1,385.513928725005,0,0
13.8750000000001,1,385.678178725005,0,0
13.9000000000001,1,385.842428725005,0,0
13.9250000000001,1,386.006678725005,0,0
13.9500000000001,1,386.170928725005,0,0
13.9750000000001,1,386.335178725005,0,0
14.0000000000001,1,386.499428725005,0,0
14.0250000000001,1,386.663678725005,0,0
14.0500000000001,1,386.827928725005,0,0
14.0750000000001,1,386.992178725005,0,0
14.1000000000001,1,387.156428725005,0,0
14.1250000000001,1,387.320678725005,0,0
14.1500000000001,1,387.484928725005,0,0
14.1750000000001,1,387.649178725005,0,0
14.2000000000001,1,387.813428725005,0,0
14.2250000000001,1,387.977678725005,0,0

14.2500000000001,1,388.141928725005,0,0
14.2750000000001,1,388.306178725005,0,0
14.3000000000001,1,388.470428725005,0,0
14.3250000000001,1,388.634678725005,0,0
14.3500000000001,1,388.798928725005,0,0
14.3750000000001,1,388.963178725005,0,0
14.4000000000001,1,389.127428725005,0,0
14.4250000000001,1,389.291678725005,0,0
14.4500000000001,1,389.455928725005,0,0
14.4750000000001,1,389.620178725005,0,0
14.5000000000001,1,389.784428725005,0,0
14.5250000000001,1,389.948678725005,0,0
14.5500000000001,1,390.112928725005,0,0
14.5750000000001,1,390.277178725005,0,0
14.6000000000001,1,390.441428725005,0,0
14.6250000000001,1,390.605678725005,0,0
14.6500000000001,1,390.769928725005,0,0
14.6750000000001,1,390.934178725005,0,0
14.7000000000001,1,391.098428725005,0,0
14.7250000000001,1,391.262678725005,0,0
14.7500000000001,1,391.426928725005,0,0
14.7750000000001,1,391.591178725005,0,0
14.8000000000001,1,391.755428725005,0,0
14.8250000000001,1,391.919678725005,0,0
14.8500000000001,0,392.001803725005,0,0
14.8750000000001,1,392.083928725005,0,0
14.9000000000001,1,392.248178725005,0,0
14.9250000000001,1,392.412428725005,0,0
14.9500000000001,0,392.494553725005,0,0
14.9750000000001,1,392.576678725005,0,0
15.0000000000001,1,392.740928725005,0,0
15.0250000000001,1,392.905178725005,0,0
15.0500000000001,1,393.069428725005,0,0
15.0750000000001,1,393.233678725005,0,0
15.1000000000001,1,393.397928725005,0,0
15.1250000000001,1,393.562178725005,0,0
15.1500000000001,1,393.726428725005,0,0
15.1750000000001,1,393.890678725004,0,0
15.2000000000001,1,394.054928725004,0,0
15.2250000000001,2,394.253196013814,0,0
15.2500000000001,0,394.369338302624,0,0
15.2750000000002,2,394.485480591434,0,0
15.3000000000002,1,394.683747880244,0,0
15.3250000000002,0,394.765872880244,0,0
15.3500000000002,1,394.847997880244,0,0
15.3750000000002,3,395.072367552816,0,0
15.4000000000002,7,395.431894551808,0,0
15.4250000000002,1,395.73130187823,0,0
15.4500000000002,3,395.955671550801,0,0
15.4750000000002,0,396.097916223373,0,0
15.5000000000002,1,396.180041223373,0,0
15.5250000000002,6,396.463330568499,0,0

15.5500000000002,1,396.746619913625,0,0
15.5750000000002,5,397.012381996277,0,0
15.6000000000002,0,397.196019078929,0,0
15.6250000000002,7,397.413301405351,0,0
15.6500000000002,0,397.630583731772,0,0
15.6750000000002,1,397.712708731772,0,0
15.7000000000002,1,397.876958731772,0,0
15.7250000000002,0,397.959083731772,0,0
15.7500000000002,1,398.041208731772,0,0
15.7750000000002,1,398.205458731772,0,0
15.8000000000002,1,398.369708731772,0,0
15.8250000000002,1,398.533958731772,0,0
15.8500000000002,1,398.698208731772,0,0
15.8750000000002,1,398.862458731772,0,0
15.9000000000002,1,399.026708731772,0,0
15.9250000000002,1,399.190958731772,0,0
15.9500000000002,1,399.355208731772,0,0
15.9750000000002,1,399.519458731772,0,0
16.0000000000002,1,399.683708731772,0,0
16.0250000000002,1,399.847958731772,0,0
16.0500000000002,1,400.012208731772,0,0
16.0750000000002,1,400.176458731772,0,0
16.1000000000002,1,400.340708731772,0,0
16.1250000000002,1,400.504958731772,0,0
16.1500000000002,1,400.669208731772,0,0
16.1750000000002,1,400.833458731771,0,0
16.2000000000001,1,400.997708731771,0,0
16.2250000000001,1,401.161958731771,0,0
16.2500000000001,1,401.326208731771,0,0
16.2750000000001,1,401.490458731771,0,0
16.3000000000001,1,401.654708731771,0,0
16.3250000000001,1,401.818958731771,0,0
16.3500000000001,1,401.983208731771,0,0
16.3750000000001,1,402.147458731771,0,0
16.4000000000001,1,402.311708731771,0,0
16.4250000000001,1,402.475958731771,0,0
16.4500000000001,1,402.640208731771,0,0
16.4750000000001,1,402.804458731771,0,0
16.5000000000001,1,402.968708731771,0,0
16.5250000000001,1,403.132958731771,0,0
16.5500000000001,1,403.297208731771,0,0
16.5750000000001,1,403.461458731771,0,0
16.6000000000001,1,403.625708731771,0,0
16.6250000000001,1,403.789958731771,0,0
16.6500000000001,1,403.954208731771,0,0
16.6750000000001,1,404.118458731771,0,0
16.7000000000001,0,404.200583731771,0,0
16.7250000000001,1,404.282708731771,0,0
16.7500000000001,1,404.446958731771,0,0
16.7750000000001,1,404.611208731771,0,0
16.8000000000001,1,404.775458731771,0,0
16.8250000000001,1,404.939708731771,0,0

16.8500000000001,1,405.103958731771,0,0
16.8750000000001,1,405.268208731771,0,0
16.9000000000001,1,405.432458731771,0,0
16.9250000000001,1,405.596708731771,0,0
16.9500000000001,1,405.760958731771,0,0
16.9750000000001,1,405.925208731771,0,0
17.0000000000001,1,406.089458731771,0,0
17.0250000000001,1,406.253708731771,0,0
17.0500000000001,1,406.417958731771,0,0
17.0750000000001,1,406.582208731771,0,0
17.1000000000001,1,406.746458731771,0,0
17.1250000000001,1,406.910708731771,0,0
17.1500000000001,1,407.074958731771,0,0
17.1750000000001,0,407.157083731771,0,0
17.2000000000001,1,407.239208731771,0,0
17.2250000000001,1,407.403458731771,0,0
17.2500000000001,1,407.567708731771,0,0
17.2750000000001,1,407.731958731771,0,0
17.3000000000001,1,407.896208731771,0,0
17.3250000000001,1,408.060458731771,0,0
17.3500000000001,1,408.224708731771,0,0
17.3750000000001,1,408.388958731771,0,0
17.4000000000001,1,408.553208731771,0,0
17.4250000000001,1,408.717458731771,0,0
17.4500000000001,1,408.881708731771,0,0
17.4750000000001,1,409.045958731771,0,0
17.5000000000001,1,409.210208731771,0,0
17.5250000000001,1,409.374458731771,0,0
17.5500000000001,1,409.538708731771,0,0
17.5750000000001,1,409.702958731771,0,0
17.6000000000001,1,409.867208731771,0,0
17.6250000000001,1,410.031458731771,0,0
17.6500000000001,1,410.195708731771,0,0
17.6750000000001,0,410.277833731771,0,0
17.7000000000001,1,410.359958731771,0,0
17.7250000000001,1,410.524208731771,0,0
17.7500000000001,1,410.688458731771,0,0
17.7750000000001,1,410.852708731771,0,0
17.8000000000001,1,411.016958731771,0,0
17.8250000000001,1,411.18120873177,0,0
17.8500000000001,1,411.34545873177,0,0
17.8750000000001,1,411.50970873177,0,0
17.9000000000001,1,411.67395873177,0,0
17.9250000000001,1,411.83820873177,0,0
17.95,1,412.00245873177,0,0
17.975,1,412.16670873177,0,0
18,1,412.33095873177,0,0
18.025,0,412.41308373177,0,0
18.05,1,412.49520873177,0,0
18.075,1,412.65945873177,0,0
18.1,1,412.82370873177,0,0
18.125,1,412.98795873177,0,0

18.15,0,413.07008373177,0,0
18.175,1,413.15220873177,0,0
18.2,0,413.23433373177,0,0
18.225,1,413.31645873177,0,0
18.25,1,413.48070873177,0,0
18.275,1,413.64495873177,0,0
18.3,1,413.80920873177,0,0
18.325,1,413.97345873177,0,0
18.35,1,414.13770873177,0,0
18.375,1,414.30195873177,0,0
18.4,1,414.46620873177,0,0
18.425,1,414.63045873177,0,0
18.45,1,414.79470873177,0,0
18.475,1,414.95895873177,0,0
18.5,1,415.12320873177,0,0
18.525,1,415.28745873177,0,0
18.55,1,415.45170873177,0,0
18.575,1,415.61595873177,0,0
18.6,1,415.78020873177,0,0
18.625,1,415.94445873177,0,0
18.65,0,416.02658373177,0,0
18.675,1,416.10870873177,0,0
18.7,1,416.27295873177,0,0
18.725,1,416.43720873177,0,0
18.75,1,416.60145873177,0,0
18.775,1,416.76570873177,0,0
18.8,1,416.92995873177,0,0
18.825,1,417.09420873177,0,0
18.85,1,417.25845873177,0,0
18.875,1,417.42270873177,0,0
18.9,1,417.58695873177,0,0
18.925,1,417.75120873177,0,0
18.95,1,417.91545873177,0,0
18.975,1,418.07970873177,0,0
19,1,418.24395873177,0,0
19.025,1,418.40820873177,0,0
19.05,1,418.57245873177,0,0
19.075,1,418.73670873177,0,0
19.1,1,418.90095873177,0,0
19.125,1,419.06520873177,0,0
19.15,0,419.14733373177,0,0
19.175,0,419.14733373177,0,0
19.2,1,419.22945873177,0,0
19.225,3,419.453828404341,0,0
19.25,0,419.596073076913,0,0
19.275,1,419.678198076913,0,0
19.3,1,419.842448076913,0,0
19.325,0,419.924573076913,0,0
19.35,1,420.006698076913,0,0
19.375,1,420.170948076913,0,0
19.4,0,420.253073076913,0,0
19.425,0,420.253073076913,0,0

19.45,5,420.436710159565,0,0
19.475,0,420.620347242217,0,0
19.5,1,420.702472242217,0,0
19.525,3,420.926841914789,0,0
19.55,1,421.151211587361,0,0
19.575,1,421.315461587361,0,0
19.6,1,421.479711587361,0,0
19.625,0,421.561836587361,0,0
19.65,1,421.643961587361,0,0
19.675,1,421.808211587361,0,0
19.7,1,421.972461587361,0,0
19.7249999999999,1,422.136711587361,0,0
19.7499999999999,1,422.300961587361,0,0
19.7749999999999,1,422.465211587361,0,0
19.7999999999999,1,422.629461587361,0,0
19.8249999999999,1,422.793711587361,0,0
19.8499999999999,1,422.957961587361,0,0
19.8749999999999,1,423.12221158736,0,0
19.8999999999999,1,423.28646158736,0,0
19.9249999999999,1,423.45071158736,0,0
19.9499999999999,1,423.61496158736,0,0
19.9749999999999,1,423.77921158736,0,0
19.9999999999999,1,423.94346158736,0,0
20.0249999999999,1,424.10771158736,0,0
20.0499999999999,1,424.27196158736,0,0
20.0749999999999,1,424.43621158736,0,0
20.0999999999999,1,424.60046158736,0,0
20.1249999999999,0,424.68258658736,0,0
20.1499999999999,1,424.76471158736,0,0
20.1749999999999,1,424.92896158736,0,0
20.1999999999999,1,425.09321158736,0,0
20.2249999999999,1,425.25746158736,0,0
20.2499999999999,1,425.42171158736,0,0
20.2749999999999,1,425.58596158736,0,0
20.2999999999999,1,425.75021158736,0,0
20.3249999999999,1,425.91446158736,0,0
20.3499999999999,1,426.07871158736,0,0
20.3749999999999,1,426.24296158736,0,0
20.3999999999999,1,426.40721158736,0,0
20.4249999999999,1,426.57146158736,0,0
20.4499999999999,1,426.73571158736,0,0
20.4749999999999,1,426.89996158736,0,0
20.4999999999999,1,427.06421158736,0,0
20.5249999999999,1,427.22846158736,0,0
20.5499999999999,1,427.39271158736,0,0
20.5749999999999,1,427.55696158736,0,0
20.5999999999999,0,427.63908658736,0,0
20.6249999999999,1,427.72121158736,0,0
20.6499999999999,1,427.88546158736,0,0
20.6749999999999,1,428.04971158736,0,0
20.6999999999999,1,428.21396158736,0,0
20.7249999999999,1,428.37821158736,0,0

20.7499999999999,1,428.54246158736,0,0
20.7749999999999,1,428.70671158736,0,0
20.7999999999999,1,428.87096158736,0,0
20.8249999999999,1,429.03521158736,0,0
20.8499999999999,1,429.19946158736,0,0
20.8749999999999,1,429.36371158736,0,0
20.8999999999999,1,429.52796158736,0,0
20.9249999999999,1,429.69221158736,0,0
20.9499999999999,1,429.85646158736,0,0
20.9749999999999,1,430.02071158736,0,0
20.9999999999999,1,430.18496158736,0,0
21.0249999999999,1,430.34921158736,0,0
21.0499999999999,1,430.51346158736,0,0
21.0749999999999,1,430.67771158736,0,0
21.0999999999999,0,430.75983658736,0,0
21.1249999999999,1,430.84196158736,0,0
21.1499999999999,1,431.00621158736,0,0
21.1749999999999,1,431.17046158736,0,0
21.1999999999999,1,431.33471158736,0,0
21.2249999999999,1,431.49896158736,0,0
21.2499999999999,1,431.66321158736,0,0
21.2749999999999,1,431.82746158736,0,0
21.2999999999999,1,431.99171158736,0,0
21.3249999999999,1,432.15596158736,0,0
21.3499999999999,1,432.32021158736,0,0
21.3749999999999,1,432.48446158736,0,0
21.3999999999999,0,432.56658658736,0,0
21.4249999999999,1,432.64871158736,0,0
21.4499999999999,1,432.81296158736,0,0
21.4749999999998,1,432.97721158736,0,0
21.4999999999998,1,433.14146158736,0,0
21.5249999999998,1,433.30571158736,0,0
21.5499999999998,0,433.38783658736,0,0
21.5749999999998,0,433.38783658736,0,0
21.5999999999998,1,433.46996158736,0,0
21.6249999999998,0,433.55208658736,0,0
21.6499999999998,1,433.63421158736,0,0
21.6749999999998,1,433.79846158736,0,0
21.6999999999998,0,433.88058658736,0,0
21.7249999999998,1,433.96271158736,0,0
21.7499999999998,0,434.04483658736,0,0
21.7749999999998,0,434.04483658736,0,0
21.7999999999998,1,434.12696158736,0,0
21.8249999999998,0,434.20908658736,0,0
21.8499999999998,1,434.29121158736,0,0
21.8749999999998,0,434.37333658736,0,0
21.8999999999998,0,434.37333658736,0,0
21.9249999999998,1,434.45546158736,0,0
21.9499999999998,1,434.61971158736,0,0
21.9749999999998,1,434.78396158736,0,0
21.9999999999998,1,434.94821158736,0,0
22.0249999999998,1,435.11246158736,0,0

22.0499999999998,1,435.27671158736,0,0
22.0749999999998,1,435.44096158736,0,0
22.0999999999998,0,435.52308658736,0,0
22.1249999999998,1,435.60521158736,0,0
22.1499999999998,1,435.76946158736,0,0
22.1749999999998,1,435.93371158736,0,0
22.1999999999998,1,436.09796158736,0,0
22.2249999999998,1,436.26221158736,0,0
22.2499999999998,0,436.34433658736,0,0
22.2749999999998,0,436.34433658736,0,0
22.2999999999998,1,436.42646158736,0,0
22.3249999999998,0,436.50858658736,0,0
22.3499999999998,1,436.59071158736,0,0
22.3749999999998,1,436.75496158736,0,0
22.3999999999998,1,436.91921158736,0,0
22.4249999999998,1,437.08346158736,0,0
22.4499999999998,0,437.16558658736,0,0
22.4749999999998,1,437.24771158736,0,0
22.4999999999998,1,437.41196158736,0,0
22.5249999999998,1,437.57621158736,0,0
22.5499999999998,1,437.74046158736,0,0
22.5749999999998,0,437.82258658736,0,0
22.5999999999998,1,437.90471158736,0,0
22.6249999999998,1,438.06896158736,0,0
22.6499999999998,1,438.23321158736,0,0
22.6749999999998,1,438.39746158736,0,0
22.6999999999998,1,438.56171158736,0,0
22.7249999999998,1,438.72596158736,0,0
22.7499999999998,0,438.80808658736,0,0
22.7749999999998,1,438.89021158736,0,0
22.7999999999998,1,439.05446158736,0,0
22.8249999999998,1,439.21871158736,0,0
22.8499999999998,0,439.30083658736,0,0
22.8749999999998,1,439.38296158736,0,0
22.8999999999998,1,439.54721158736,0,0
22.9249999999998,1,439.71146158736,0,0
22.9499999999998,1,439.87571158736,0,0
22.9749999999998,1,440.03996158736,0,0
22.9999999999998,0,440.12208658736,0,0
23.0249999999998,1,440.20421158736,0,0
23.0499999999998,1,440.36846158736,0,0
23.0749999999998,0,440.45058658736,0,0
23.0999999999998,1,440.53271158736,0,0
23.1249999999998,1,440.69696158736,0,0
23.1499999999998,0,440.77908658736,0,0
23.1749999999998,1,440.86121158736,0,0
23.1999999999998,0,440.94333658736,0,0
23.2249999999997,3,441.085581259931,0,0
23.2499999999997,6,441.428990277629,0,0
23.2749999999997,1,441.712279622755,0,0
23.2999999999997,0,441.794404622755,0,0
23.3249999999997,1,441.876529622755,0,0

23.3499999999997,0,441.958654622755,0,0
23.3749999999997,0,441.958654622755,0,0
23.3999999999997,0,441.958654622755,0,0
23.4249999999997,0,441.958654622755,0,0
23.4499999999997,4,442.122904622755,0,0
23.4749999999997,0,442.287154622755,0,0
23.4999999999997,1,442.369279622755,0,0
23.5249999999997,1,442.533529622755,0,0
23.5499999999997,1,442.697779622755,0,0
23.5749999999997,1,442.862029622755,0,0
23.5999999999997,1,443.026279622755,0,0
23.6249999999997,0,443.108404622755,0,0
23.6499999999997,1,443.190529622755,0,0
23.6749999999997,1,443.354779622755,0,0
23.6999999999997,1,443.519029622755,0,0
23.7249999999997,1,443.683279622755,0,0
23.7499999999997,1,443.847529622755,0,0
23.7749999999997,1,444.011779622755,0,0
23.7999999999997,1,444.176029622755,0,0
23.8249999999997,1,444.340279622755,0,0
23.8499999999997,1,444.504529622755,0,0
23.8749999999997,1,444.668779622755,0,0
23.8999999999997,1,444.833029622755,0,0
23.9249999999997,1,444.997279622755,0,0
23.9499999999997,1,445.161529622755,0,0
23.9749999999997,1,445.325779622755,0,0
23.9999999999997,1,445.490029622755,0,0
24.0249999999997,1,445.654279622755,0,0
24.0499999999997,1,445.818529622755,0,0
24.0749999999997,1,445.982779622755,0,0
24.0999999999997,0,446.064904622755,0,0
24.1249999999997,0,446.064904622755,0,0
24.1499999999997,1,446.147029622755,0,0
24.1749999999997,1,446.311279622755,0,0
24.1999999999997,1,446.475529622755,0,0
24.2249999999997,0,446.557654622755,0,0
24.2499999999997,1,446.639779622755,0,0
24.2749999999997,1,446.804029622755,0,0
24.2999999999997,0,446.886154622755,0,0
24.3249999999997,0,446.886154622755,0,0
24.3499999999997,1,446.968279622755,0,0
24.3749999999997,1,447.132529622755,0,0
24.3999999999997,0,447.214654622755,0,0
24.4249999999997,1,447.296779622755,0,0
24.4499999999997,0,447.378904622755,0,0
24.4749999999997,1,447.461029622755,0,0
24.4999999999997,1,447.625279622755,0,0
24.5249999999997,0,447.707404622755,0,0
24.5499999999997,1,447.789529622755,0,0
24.5749999999997,1,447.953779622755,0,0
24.5999999999997,0,448.035904622755,0,0
24.6249999999997,1,448.118029622755,0,0

24.6499999999997,1,448.282279622755,0,0
24.6749999999997,1,448.446529622755,0,0
24.6999999999997,0,448.528654622755,0,0
24.7249999999997,1,448.610779622755,0,0
24.7499999999997,1,448.775029622755,0,0
24.7749999999997,1,448.939279622755,0,0
24.7999999999997,1,449.103529622755,0,0
24.8249999999997,1,449.267779622755,0,0
24.8499999999997,1,449.432029622755,0,0
24.8749999999997,1,449.596279622755,0,0
24.8999999999997,1,449.760529622755,0,0
24.9249999999997,1,449.924779622755,0,0
24.9499999999997,1,450.089029622755,0,0
24.9749999999997,1,450.253279622755,0,0
24.9999999999996,1,450.417529622755,0,0
25.0249999999996,1,450.581779622755,0,0
25.0499999999996,1,450.746029622755,0,0
25.0749999999996,1,450.910279622755,0,0
25.0999999999996,1,451.074529622755,0,0
25.1249999999996,1,451.238779622755,0,0
25.1499999999996,1,451.403029622755,0,0
25.1749999999996,1,451.567279622754,0,0
25.1999999999996,1,451.731529622754,0,0
25.2249999999996,1,451.895779622754,0,0
25.2499999999996,0,451.977904622754,0,0
25.2749999999996,1,452.060029622754,0,0
25.2999999999996,0,452.142154622755,0,0
25.3249999999996,1,452.224279622755,0,0
25.3499999999996,1,452.388529622755,0,0
25.3749999999996,1,452.552779622754,0,0
25.3999999999996,0,452.634904622755,0,0
25.4249999999996,1,452.717029622755,0,0
25.4499999999996,1,452.881279622755,0,0
25.4749999999996,1,453.045529622754,0,0
25.4999999999996,0,453.127654622755,0,0
25.5249999999996,1,453.209779622755,0,0
25.5499999999996,0,453.291904622755,0,0
25.5749999999996,0,453.291904622755,0,0
25.5999999999996,1,453.374029622755,0,0
25.6249999999996,1,453.538279622755,0,0
25.6499999999996,1,453.702529622755,0,0
25.6749999999996,1,453.866779622755,0,0
25.6999999999996,1,454.031029622754,0,0
25.7249999999996,0,454.113154622755,0,0
25.7499999999996,0,454.113154622755,0,0
25.7749999999996,1,454.195279622755,0,0
25.7999999999996,0,454.277404622755,0,0
25.8249999999996,0,454.277404622755,0,0
25.8499999999996,1,454.359529622755,0,0
25.8749999999996,1,454.523779622755,0,0
25.8999999999996,1,454.688029622755,0,0
25.9249999999996,0,454.770154622755,0,0

25.9499999999996,1,454.852279622755,0,0
25.9749999999996,1,455.016529622755,0,0
25.9999999999996,0,455.098654622755,0,0
26.0249999999996,1,455.180779622755,0,0
26.0499999999996,1,455.345029622755,0,0
26.0749999999996,1,455.509279622755,0,0
26.0999999999996,1,455.673529622755,0,0
26.1249999999996,1,455.837779622755,0,0
26.1499999999996,1,456.002029622754,0,0
26.1749999999996,1,456.166279622754,0,0
26.1999999999996,1,456.330529622754,0,0
26.2249999999996,1,456.494779622754,0,0
26.2499999999996,1,456.659029622754,0,0
26.2749999999996,1,456.823279622754,0,0
26.2999999999996,1,456.987529622754,0,0
26.3249999999996,1,457.151779622754,0,0
26.3499999999996,1,457.316029622754,0,0
26.3749999999996,1,457.480279622754,0,0
26.3999999999996,1,457.644529622754,0,0
26.4249999999996,1,457.808779622754,0,0
26.4499999999996,1,457.973029622754,0,0
26.4749999999996,0,458.055154622754,0,0
26.4999999999996,1,458.137279622754,0,0
26.5249999999996,1,458.301529622754,0,0
26.5499999999996,1,458.465779622754,0,0
26.5749999999996,1,458.630029622754,0,0
26.5999999999996,1,458.794279622754,0,0
26.6249999999996,1,458.958529622754,0,0
26.6499999999996,1,459.122779622754,0,0
26.6749999999996,1,459.287029622754,0,0
26.6999999999996,1,459.451279622754,0,0
26.7249999999996,1,459.615529622754,0,0
26.7499999999995,1,459.779779622754,0,0
26.7749999999995,1,459.944029622754,0,0
26.7999999999995,1,460.108279622754,0,0
26.8249999999995,1,460.272529622754,0,0
26.8499999999995,1,460.436779622754,0,0
26.8749999999995,1,460.601029622754,0,0
26.8999999999995,1,460.765279622754,0,0
26.9249999999995,1,460.929529622754,0,0
26.9499999999995,0,461.011654622754,0,0
26.9749999999995,1,461.093779622754,0,0
26.9999999999995,0,461.175904622754,0,0
27.0249999999995,0,461.175904622754,0,0
27.0499999999995,1,461.258029622754,0,0
27.0749999999995,1,461.422279622754,0,0
27.0999999999995,4,461.668654622754,0,0
27.1249999999995,1,461.915029622754,0,0
27.1499999999995,1,462.079279622754,0,0
27.1749999999995,2,462.277546911564,0,0
27.1999999999995,0,462.393689200374,0,0
27.2249999999995,0,462.393689200374,0,0

27.2499999999995,2,462.509831489184,0,0
27.2749999999995,2,462.742116066804,0,0
27.2999999999995,0,462.858258355613,0,0
27.3249999999995,0,462.858258355613,0,0
27.3499999999995,1,462.940383355613,0,0
27.3749999999995,2,463.138650644423,0,0
27.3999999999995,1,463.336917933233,0,0
27.4249999999995,1,463.501167933233,0,0
27.4499999999995,0,463.583292933233,0,0
27.4749999999995,1,463.665417933233,0,0
27.4999999999995,1,463.829667933233,0,0
27.5249999999995,1,463.993917933233,0,0
27.5499999999995,1,464.158167933233,0,0
27.5749999999995,1,464.322417933233,0,0
27.5999999999995,1,464.486667933233,0,0
27.6249999999995,1,464.650917933233,0,0
27.6499999999995,1,464.815167933233,0,0
27.6749999999995,1,464.979417933233,0,0
27.6999999999995,1,465.143667933233,0,0
27.7249999999995,1,465.307917933233,0,0
27.7499999999995,1,465.472167933233,0,0
27.7749999999995,1,465.636417933233,0,0
27.7999999999995,1,465.800667933233,0,0
27.8249999999995,1,465.964917933233,0,0
27.8499999999995,1,466.129167933233,0,0
27.8749999999995,1,466.293417933233,0,0
27.8999999999995,1,466.457667933233,0,0
27.9249999999995,1,466.621917933233,0,0
27.9499999999995,1,466.786167933233,0,0
27.9749999999995,1,466.950417933233,0,0
27.9999999999995,1,467.114667933233,0,0
28.0249999999995,1,467.278917933233,0,0
28.0499999999995,1,467.443167933233,0,0
28.0749999999995,1,467.607417933233,0,0
28.0999999999995,1,467.771667933233,0,0
28.1249999999995,1,467.935917933233,0,0
28.1499999999995,1,468.100167933233,0,0
28.1749999999995,1,468.264417933233,0,0
28.1999999999995,1,468.428667933233,0,0
28.2249999999995,1,468.592917933233,0,0
28.2499999999995,1,468.757167933233,0,0
28.2749999999995,1,468.921417933233,0,0
28.2999999999995,1,469.085667933233,0,0
28.3249999999995,1,469.249917933233,0,0
28.3499999999995,1,469.414167933233,0,0
28.3749999999995,1,469.578417933233,0,0
28.3999999999995,0,469.660542933233,0,0
28.4249999999995,1,469.742667933233,0,0
28.4499999999995,1,469.906917933233,0,0
28.4749999999995,1,470.071167933233,0,0
28.4999999999994,1,470.235417933233,0,0
28.5249999999994,1,470.399667933233,0,0

28.5499999999994,1,470.563917933233,0,0
28.5749999999994,1,470.728167933233,0,0
28.5999999999994,1,470.892417933233,0,0
28.6249999999994,1,471.056667933233,0,0
28.6499999999994,1,471.220917933233,0,0
28.6749999999994,1,471.385167933232,0,0
28.6999999999994,1,471.549417933232,0,0
28.7249999999994,1,471.713667933232,0,0
28.7499999999994,1,471.877917933232,0,0
28.7749999999994,1,472.042167933232,0,0
28.7999999999994,1,472.206417933232,0,0
28.8249999999994,1,472.370667933232,0,0
28.8499999999994,1,472.534917933232,0,0
28.8749999999994,1,472.699167933232,0,0
28.8999999999994,1,472.863417933232,0,0
28.9249999999994,2,473.061685222042,0,0
28.9499999999994,1,473.259952510852,0,0
28.9749999999994,1,473.424202510852,0,0
28.9999999999994,1,473.588452510852,0,0
29.0249999999994,1,473.752702510852,0,0
29.0499999999994,1,473.916952510852,0,0
29.0749999999994,1,474.081202510852,0,0
29.0999999999994,1,474.245452510852,0,0
29.1249999999994,1,474.409702510852,0,0
29.1499999999994,1,474.573952510852,0,0
29.1749999999994,1,474.738202510852,0,0
29.1999999999994,1,474.902452510852,0,0
29.2249999999994,1,475.066702510852,0,0
29.2499999999994,1,475.230952510852,0,0
29.2749999999994,1,475.395202510852,0,0
29.2999999999994,1,475.559452510852,0,0
29.3249999999994,1,475.723702510852,0,0
29.3499999999994,1,475.887952510852,0,0
29.3749999999994,1,476.052202510852,0,0
29.3999999999994,2,476.250469799662,0,0
29.4249999999994,0,476.366612088472,0,0
29.4499999999994,1,476.448737088472,0,0
29.4749999999994,1,476.612987088472,0,0
29.4999999999994,0,476.695112088472,0,0
29.5249999999994,1,476.777237088472,0,0
29.5499999999994,1,476.941487088472,0,0
29.5749999999994,1,477.105737088472,0,0
29.5999999999994,1,477.269987088472,0,0
29.6249999999994,1,477.434237088472,0,0
29.6499999999994,1,477.598487088472,0,0
29.6749999999994,1,477.762737088472,0,0
29.6999999999994,1,477.926987088472,0,0
29.7249999999994,1,478.091237088472,0,0
29.7499999999994,1,478.255487088472,0,0
29.7749999999994,1,478.419737088471,0,0
29.7999999999994,1,478.583987088471,0,0
29.8249999999994,1,478.748237088471,0,0

29.8499999999994,1,478.912487088471,0,0
29.8749999999994,1,479.076737088471,0,0
29.8999999999994,0,479.158862088471,0,0
29.9249999999994,1,479.240987088471,0,0
29.9499999999994,1,479.405237088471,0,0
29.9749999999994,1,479.569487088471,0,0
29.9999999999994,0,479.651612088471,0,0
30.0249999999994,1,479.733737088471,0,0
30.0499999999994,1,479.897987088471,0,0
30.0749999999994,1,480.062237088471,0,0
30.0999999999994,1,480.226487088471,0,0
30.1249999999994,1,480.390737088471,0,0
30.1499999999994,1,480.554987088471,0,0
30.1749999999994,1,480.719237088471,0,0
30.1999999999994,1,480.883487088471,0,0
30.2249999999994,1,481.047737088471,0,0
30.2499999999994,1,481.211987088471,0,0
30.2749999999993,1,481.376237088471,0,0
30.2999999999993,1,481.540487088471,0,0
30.3249999999993,1,481.704737088471,0,0
30.3499999999993,1,481.868987088471,0,0
30.3749999999993,1,482.033237088471,0,0
30.3999999999993,1,482.197487088471,0,0
30.4249999999993,1,482.361737088471,0,0
30.4499999999993,1,482.525987088471,0,0
30.4749999999993,1,482.690237088471,0,0
30.4999999999993,1,482.854487088471,0,0
30.5249999999993,1,483.018737088471,0,0
30.5499999999993,1,483.182987088471,0,0
30.5749999999993,1,483.347237088471,0,0
30.5999999999993,1,483.511487088471,0,0
30.6249999999993,1,483.675737088471,0,0
30.6499999999993,1,483.839987088471,0,0
30.6749999999993,1,484.004237088471,0,0
30.6999999999993,1,484.168487088471,0,0
30.7249999999993,1,484.332737088471,0,0
30.7499999999993,1,484.496987088471,0,0
30.7749999999993,1,484.661237088471,0,0
30.7999999999993,1,484.825487088471,0,0
30.8249999999993,1,484.989737088471,0,0
30.8499999999993,1,485.153987088471,0,0
30.8749999999993,1,485.318237088471,0,0
30.8999999999993,0,485.400362088471,0,0
30.9249999999993,0,485.400362088471,0,0
30.9499999999993,7,485.617644414892,0,0
30.9749999999993,2,485.951069030123,0,0
30.9999999999993,1,486.149336318933,0,0
31.0249999999993,0,486.231461318933,0,0
31.0499999999993,1,486.313586318933,0,0
31.0749999999993,1,486.477836318933,0,0
31.0999999999993,1,486.642086318933,0,0
31.1249999999993,1,486.806336318933,0,0

31.1499999999993,1,486.970586318933,0,0
31.1749999999993,0,487.052711318933,0,0
31.1999999999993,0,487.052711318933,0,0
31.2249999999993,4,487.216961318933,0,0
31.2499999999993,4,487.545461318933,0,0
31.2749999999993,5,487.893348401585,0,0
31.2999999999993,0,488.076985484237,0,0
31.3249999999993,1,488.159110484237,0,0
31.3499999999993,1,488.323360484237,0,0
31.3749999999993,0,488.405485484237,0,0
31.3999999999993,1,488.487610484237,0,0
31.4249999999993,1,488.651860484237,0,0
31.4499999999993,1,488.816110484237,0,0
31.4749999999993,1,488.980360484237,0,0
31.4999999999993,1,489.144610484237,0,0
31.5249999999993,1,489.308860484237,0,0
31.5499999999993,1,489.473110484237,0,0
31.5749999999993,1,489.637360484237,0,0
31.5999999999993,1,489.801610484237,0,0
31.6249999999993,1,489.965860484237,0,0
31.6499999999993,1,490.130110484237,0,0
31.6749999999993,1,490.294360484237,0,0
31.6999999999993,1,490.458610484237,0,0
31.7249999999993,1,490.622860484237,0,0
31.7499999999993,1,490.787110484237,0,0
31.7749999999993,1,490.951360484237,0,0
31.7999999999993,1,491.115610484237,0,0
31.8249999999993,1,491.279860484237,0,0
31.8499999999993,0,491.361985484237,0,0
31.8749999999993,1,491.444110484237,0,0
31.8999999999993,1,491.608360484237,0,0
31.9249999999993,1,491.772610484237,0,0
31.9499999999993,1,491.936860484237,0,0
31.9749999999993,1,492.101110484237,0,0
31.9999999999993,1,492.265360484237,0,0
32.0249999999993,1,492.429610484237,0,0
32.0499999999993,1,492.593860484237,0,0
32.0749999999993,1,492.758110484237,0,0
32.0999999999992,1,492.922360484237,0,0
32.1249999999992,1,493.086610484237,0,0
32.1499999999992,1,493.250860484237,0,0
32.1749999999992,1,493.415110484237,0,0
32.1999999999992,1,493.579360484237,0,0
32.2249999999992,1,493.743610484237,0,0
32.2499999999992,1,493.907860484237,0,0
32.2749999999992,1,494.072110484237,0,0
32.2999999999992,1,494.236360484237,0,0
32.3249999999992,1,494.400610484237,0,0
32.3499999999992,1,494.564860484237,0,0
32.3749999999992,1,494.729110484237,0,0
32.3999999999992,1,494.893360484237,0,0
32.4249999999992,1,495.057610484237,0,0

32.4499999999992,1,495.221860484237,0,0
32.4749999999992,1,495.386110484237,0,0
32.4999999999992,1,495.550360484237,0,0
32.5249999999992,1,495.714610484237,0,0
32.5499999999992,1,495.878860484237,0,0
32.5749999999992,1,496.043110484237,0,0
32.5999999999992,1,496.207360484237,0,0
32.6249999999992,0,496.289485484237,0,0
32.6499999999992,1,496.371610484237,0,0
32.6749999999992,1,496.535860484237,0,0
32.6999999999992,1,496.700110484237,0,0
32.7249999999992,1,496.864360484237,0,0
32.7499999999992,1,497.028610484237,0,0
32.7749999999992,1,497.192860484237,0,0
32.7999999999992,1,497.357110484237,0,0
32.8249999999992,2,497.555377773046,0,0
32.8499999999992,1,497.753645061856,0,0
32.8749999999992,1,497.917895061856,0,0
32.8999999999992,1,498.082145061856,0,0
32.9249999999992,1,498.246395061856,0,0
32.9499999999992,1,498.410645061856,0,0
32.9749999999992,1,498.574895061856,0,0
32.9999999999992,1,498.739145061856,0,0
33.0249999999992,1,498.903395061856,0,0
33.0499999999992,1,499.067645061856,0,0
33.0749999999992,1,499.231895061856,0,0
33.0999999999992,1,499.396145061856,0,0
33.1249999999992,1,499.560395061856,0,0
33.1499999999992,1,499.724645061856,0,0
33.1749999999992,1,499.888895061856,0,0
33.1999999999992,1,500.053145061856,0,0
33.2249999999992,1,500.217395061856,0,0
33.2499999999992,1,500.381645061856,0,0
33.2749999999992,1,500.545895061856,0,0
33.2999999999992,1,500.710145061856,0,0
33.3249999999992,3,500.934514734428,0,0
33.3499999999992,1,501.158884406999,0,0
33.3749999999992,1,501.323134406999,0,0
33.3999999999992,1,501.487384406999,0,0
33.4249999999992,1,501.651634406999,0,0
33.4499999999992,1,501.815884406999,0,0
33.4749999999992,1,501.980134406999,0,0
33.4999999999992,1,502.144384406999,0,0
33.5249999999992,1,502.308634406999,0,0
33.5499999999992,1,502.472884406999,0,0
33.5749999999992,1,502.637134406999,0,0
33.5999999999992,1,502.801384406999,0,0
33.6249999999992,1,502.965634406999,0,0
33.6499999999992,1,503.129884406999,0,0
33.6749999999992,1,503.294134406999,0,0
33.6999999999992,1,503.458384406999,0,0
33.7249999999992,1,503.622634406999,0,0

33.7499999999992,1,503.786884406999,0,0
33.7749999999992,1,503.951134406999,0,0
33.7999999999992,1,504.115384406999,0,0
33.8249999999992,0,504.197509406999,0,0
33.8499999999991,1,504.279634406999,0,0
33.8749999999991,1,504.443884406999,0,0
33.8999999999991,1,504.608134406999,0,0
33.9249999999991,1,504.772384406999,0,0
33.9499999999991,1,504.936634406999,0,0
33.9749999999991,1,505.100884406999,0,0
33.9999999999991,1,505.265134406999,0,0
34.0249999999991,0,505.347259406999,0,0
34.0499999999991,1,505.429384406999,0,0
34.0749999999991,1,505.593634406999,0,0
34.0999999999991,1,505.757884406999,0,0
34.1249999999991,1,505.922134406999,0,0
34.1499999999991,1,506.086384406999,0,0
34.1749999999991,1,506.250634406999,0,0
34.1999999999991,1,506.414884406999,0,0
34.2249999999991,1,506.579134406999,0,0
34.2499999999991,1,506.743384406999,0,0
34.2749999999991,1,506.907634406999,0,0
34.2999999999991,0,506.989759406999,0,0
34.3249999999991,1,507.071884406999,0,0
34.3499999999991,1,507.236134406999,0,0
34.3749999999991,1,507.400384406999,0,0
34.3999999999991,1,507.564634406999,0,0
34.4249999999991,1,507.728884406999,0,0
34.4499999999991,1,507.893134406999,0,0
34.4749999999991,1,508.057384406999,0,0
34.4999999999991,1,508.221634406999,0,0
34.5249999999991,1,508.385884406999,0,0
34.5499999999991,1,508.550134406999,0,0
34.5749999999991,1,508.714384406998,0,0
34.5999999999991,1,508.878634406998,0,0
34.6249999999991,1,509.042884406998,0,0
34.6499999999991,1,509.207134406998,0,0
34.6749999999991,1,509.371384406998,0,0
34.6999999999991,1,509.535634406998,0,0
34.7249999999991,1,509.699884406998,0,0
34.7499999999991,1,509.864134406998,0,0
34.7749999999991,1,510.028384406998,0,0
34.7999999999991,0,510.110509406998,0,0
34.8249999999991,0,510.110509406998,0,0
34.8499999999991,2,510.226651695808,0,0
34.8749999999991,6,510.543958329744,0,0
34.8999999999991,4,510.90937267487,0,0
34.9249999999991,0,511.07362267487,0,0
34.9499999999991,0,511.07362267487,0,0
34.9749999999991,1,511.15574767487,0,0
34.9999999999991,2,511.35401496368,0,0
35.0249999999991,1,511.55228225249,0,0

35.0499999999991,2,511.7505495413,0,0
35.0749999999991,2,511.98283411892,0,0
35.0999999999991,0,512.09897640773,0,0
35.1249999999991,0,512.09897640773,0,0
35.1499999999991,0,512.09897640773,0,0
35.1749999999991,0,512.09897640773,0,0
35.1999999999991,2,512.21511869654,0,0
35.2249999999991,0,512.331260985349,0,0
35.2499999999991,0,512.331260985349,0,0
35.2749999999991,0,512.331260985349,0,0
35.2999999999991,1,512.413385985349,0,0
35.3249999999991,1,512.57763598535,0,0
35.3499999999991,1,512.74188598535,0,0
35.3749999999991,1,512.90613598535,0,0
35.3999999999991,1,513.07038598535,0,0
35.4249999999991,1,513.23463598535,0,0
35.4499999999991,1,513.39888598535,0,0
35.4749999999991,1,513.56313598535,0,0
35.4999999999991,1,513.72738598535,0,0
35.5249999999991,1,513.89163598535,0,0
35.5499999999991,1,514.05588598535,0,0
35.5749999999991,1,514.22013598535,0,0
35.599999999999,1,514.38438598535,0,0
35.624999999999,1,514.54863598535,0,0
35.649999999999,1,514.71288598535,0,0
35.674999999999,1,514.87713598535,0,0
35.699999999999,1,515.04138598535,0,0
35.724999999999,1,515.20563598535,0,0
35.749999999999,1,515.36988598535,0,0
35.774999999999,0,515.45201098535,0,0
35.799999999999,0,515.45201098535,0,0
35.824999999999,2,515.56815327416,0,0
35.849999999999,2,515.80043785178,0,0
35.874999999999,1,515.99870514059,0,0
35.899999999999,0,516.08083014059,0,0
35.924999999999,1,516.16295514059,0,0
35.949999999999,1,516.32720514059,0,0
35.974999999999,1,516.49145514059,0,0
35.999999999999,1,516.65570514059,0,0
36.024999999999,1,516.81995514059,0,0
36.049999999999,1,516.98420514059,0,0
36.074999999999,1,517.14845514059,0,0
36.099999999999,0,517.23058014059,0,0
36.124999999999,1,517.31270514059,0,0
36.149999999999,1,517.47695514059,0,0
36.174999999999,1,517.64120514059,0,0
36.199999999999,1,517.80545514059,0,0
36.224999999999,1,517.96970514059,0,0
36.249999999999,0,518.05183014059,0,0
36.274999999999,1,518.13395514059,0,0
36.299999999999,1,518.29820514059,0,0
36.324999999999,1,518.46245514059,0,0

36.349999999999,1,518.62670514059,0,0
36.374999999999,1,518.79095514059,0,0
36.399999999999,1,518.95520514059,0,0
36.424999999999,1,519.119455140591,0,0
36.449999999999,1,519.283705140591,0,0
36.474999999999,1,519.447955140591,0,0
36.499999999999,0,519.530080140591,0,0
36.524999999999,1,519.612205140591,0,0
36.549999999999,1,519.776455140591,0,0
36.574999999999,1,519.940705140591,0,0
36.599999999999,1,520.104955140591,0,0
36.624999999999,1,520.269205140591,0,0
36.649999999999,1,520.433455140591,0,0
36.674999999999,1,520.597705140591,0,0
36.699999999999,1,520.761955140591,0,0
36.724999999999,1,520.926205140591,0,0
36.749999999999,1,521.090455140591,0,0
36.774999999999,1,521.254705140591,0,0
36.799999999999,1,521.418955140591,0,0
36.824999999999,1,521.583205140591,0,0
36.849999999999,1,521.747455140591,0,0
36.874999999999,0,521.829580140591,0,0
36.899999999999,0,521.829580140591,0,0
36.924999999999,1,521.911705140591,0,0
36.949999999999,1,522.075955140591,0,0
36.974999999999,1,522.240205140591,0,0
36.999999999999,1,522.404455140591,0,0
37.024999999999,0,522.486580140591,0,0
37.049999999999,1,522.568705140591,0,0
37.074999999999,0,522.650830140591,0,0
37.099999999999,0,522.650830140591,0,0
37.124999999999,1,522.732955140591,0,0
37.149999999999,1,522.897205140591,0,0
37.174999999999,0,522.979330140591,0,0
37.199999999999,1,523.061455140591,0,0
37.224999999999,1,523.225705140591,0,0
37.249999999999,2,523.423972429401,0,0
37.274999999999,1,523.622239718211,0,0
37.299999999999,1,523.786489718211,0,0
37.324999999999,1,523.950739718211,0,0
37.349999999999,1,524.114989718211,0,0
37.3749999999989,1,524.279239718211,0,0
37.3999999999989,1,524.443489718211,0,0
37.4249999999989,1,524.607739718212,0,0
37.4499999999989,1,524.771989718212,0,0
37.4749999999989,1,524.936239718212,0,0
37.4999999999989,1,525.100489718212,0,0
37.5249999999989,1,525.264739718212,0,0
37.5499999999989,1,525.428989718212,0,0
37.5749999999989,1,525.593239718212,0,0
37.5999999999989,1,525.757489718212,0,0
37.6249999999989,1,525.921739718212,0,0

37.6499999999989,1,526.085989718212,0,0
37.6749999999989,1,526.250239718212,0,0
37.6999999999989,1,526.414489718212,0,0
37.7249999999989,0,526.496614718212,0,0
37.7499999999989,0,526.496614718212,0,0
37.7749999999989,1,526.578739718212,0,0
37.7999999999989,1,526.742989718212,0,0
37.8249999999989,1,526.907239718212,0,0
37.8499999999989,0,526.989364718212,0,0
37.8749999999989,1,527.071489718212,0,0
37.8999999999989,1,527.235739718212,0,0
37.9249999999989,1,527.399989718212,0,0
37.9499999999989,1,527.564239718212,0,0
37.9749999999989,1,527.728489718212,0,0
37.9999999999989,1,527.892739718212,0,0
38.0249999999989,1,528.056989718212,0,0
38.0499999999989,1,528.221239718212,0,0
38.0749999999989,1,528.385489718212,0,0
38.0999999999989,1,528.549739718212,0,0
38.1249999999989,1,528.713989718212,0,0
38.1499999999989,1,528.878239718213,0,0
38.1749999999989,1,529.042489718213,0,0
38.1999999999989,1,529.206739718213,0,0
38.2249999999989,1,529.370989718213,0,0
38.2499999999989,1,529.535239718213,0,0
38.2749999999989,1,529.699489718213,0,0
38.2999999999989,1,529.863739718213,0,0
38.3249999999989,1,530.027989718213,0,0
38.3499999999989,1,530.192239718213,0,0
38.3749999999989,1,530.356489718213,0,0
38.3999999999989,1,530.520739718213,0,0
38.4249999999989,1,530.684989718213,0,0
38.4499999999989,1,530.849239718213,0,0
38.4749999999989,1,531.013489718213,0,0
38.4999999999989,1,531.177739718213,0,0
38.5249999999989,1,531.341989718213,0,0
38.5499999999989,1,531.506239718213,0,0
38.5749999999989,1,531.670489718213,0,0
38.5999999999989,1,531.834739718213,0,0
38.6249999999989,1,531.998989718213,0,0
38.6499999999989,1,532.163239718213,0,0
38.6749999999989,1,532.327489718213,0,0
38.6999999999989,1,532.491739718213,0,0
38.7249999999989,4,532.738114718213,0,0
38.7499999999989,0,532.902364718213,0,0
38.7749999999989,4,533.066614718213,0,0
38.7999999999989,0,533.230864718213,0,0
38.8249999999989,4,533.395114718213,0,0
38.8499999999989,0,533.559364718213,0,0
38.8749999999989,1,533.641489718214,0,0
38.8999999999989,0,533.723614718214,0,0
38.9249999999989,2,533.839757007023,0,0

38.9499999999989,0,533.955899295833,0,0
38.9749999999989,0,533.955899295833,0,0
38.9999999999989,3,534.098143968405,0,0
39.0249999999989,5,534.424025723629,0,0
39.0499999999989,0,534.607662806281,0,0
39.0749999999989,3,534.749907478852,0,0
39.0999999999989,0,534.892152151424,0,0
39.1249999999988,1,534.974277151424,0,0
39.1499999999988,1,535.138527151424,0,0
39.1749999999988,1,535.302777151424,0,0
39.1999999999988,3,535.527146823996,0,0
39.2249999999988,1,535.751516496567,0,0
39.2499999999988,1,535.915766496567,0,0
39.2749999999988,1,536.080016496567,0,0
39.2999999999988,1,536.244266496567,0,0
39.3249999999988,1,536.408516496567,0,0
39.3499999999988,1,536.572766496567,0,0
39.3749999999988,1,536.737016496567,0,0
39.3999999999988,1,536.901266496567,0,0
39.4249999999988,1,537.065516496568,0,0
39.4499999999988,1,537.229766496568,0,0
39.4749999999988,1,537.394016496568,0,0
39.4999999999988,1,537.558266496568,0,0
39.5249999999988,1,537.722516496568,0,0
39.5499999999988,1,537.886766496568,0,0
39.5749999999988,1,538.051016496568,0,0
39.5999999999988,1,538.215266496568,0,0
39.6249999999988,1,538.379516496568,0,0
39.6499999999988,1,538.543766496568,0,0
39.6749999999988,1,538.708016496568,0,0
39.6999999999988,1,538.872266496568,0,0
39.7249999999988,1,539.036516496568,0,0
39.7499999999988,1,539.200766496568,0,0
39.7749999999988,1,539.365016496568,0,0
39.7999999999988,1,539.529266496568,0,0
39.8249999999988,1,539.693516496568,0,0
39.8499999999988,1,539.857766496568,0,0
39.8749999999988,1,540.022016496568,0,0
39.8999999999988,1,540.186266496568,0,0
39.9249999999988,1,540.350516496568,0,0
39.9499999999988,1,540.514766496568,0,0
39.9749999999988,1,540.679016496568,0,0
39.9999999999988,1,540.843266496568,0,0
40.0249999999988,1,541.007516496568,0,0
40.0499999999988,1,541.171766496568,0,0
40.0749999999988,1,541.336016496569,0,0
40.0999999999988,1,541.500266496569,0,0
40.1249999999988,1,541.664516496569,0,0
40.1499999999988,1,541.828766496569,0,0
40.1749999999988,3,542.05313616914,0,0
40.1999999999988,1,542.277505841712,0,0
40.2249999999988,1,542.441755841712,0,0

40.2499999999988,1,542.606005841712,0,0
40.2749999999988,1,542.770255841712,0,0
40.2999999999988,1,542.934505841712,0,0
40.3249999999988,1,543.098755841712,0,0
40.3499999999988,1,543.263005841712,0,0
40.3749999999988,1,543.427255841712,0,0
40.3999999999988,1,543.591505841712,0,0
40.4249999999988,1,543.755755841712,0,0
40.4499999999988,1,543.920005841712,0,0
40.4749999999988,1,544.084255841712,0,0
40.4999999999988,1,544.248505841712,0,0
40.5249999999988,1,544.412755841712,0,0
40.5499999999988,1,544.577005841712,0,0
40.5749999999988,1,544.741255841712,0,0
40.5999999999988,1,544.905505841712,0,0
40.6249999999988,1,545.069755841712,0,0
40.6499999999988,0,545.151880841712,0,0
40.6749999999988,0,545.151880841712,0,0
40.6999999999988,1,545.234005841712,0,0
40.7249999999988,1,545.398255841713,0,0
40.7499999999988,0,545.480380841713,0,0
40.7749999999988,1,545.562505841713,0,0
40.7999999999988,1,545.726755841713,0,0
40.8249999999988,0,545.808880841713,0,0
40.8499999999988,1,545.891005841713,0,0
40.8749999999987,1,546.055255841713,0,0
40.8999999999987,0,546.137380841713,0,0
40.9249999999987,1,546.219505841713,0,0
40.9499999999987,1,546.383755841713,0,0
40.9749999999987,1,546.548005841713,0,0
40.9999999999987,1,546.712255841713,0,0
41.0249999999987,1,546.876505841713,0,0
41.0499999999987,1,547.040755841713,0,0
41.0749999999987,1,547.205005841713,0,0
41.0999999999987,1,547.369255841713,0,0
41.1249999999987,1,547.533505841713,0,0
41.1499999999987,0,547.615630841713,0,0
41.1749999999987,1,547.697755841713,0,0
41.1999999999987,1,547.862005841713,0,0
41.2249999999987,1,548.026255841713,0,0
41.2499999999987,1,548.190505841713,0,0
41.2749999999987,1,548.354755841713,0,0
41.2999999999987,1,548.519005841713,0,0
41.3249999999987,1,548.683255841713,0,0
41.3499999999987,1,548.847505841713,0,0
41.3749999999987,1,549.011755841713,0,0
41.3999999999987,0,549.093880841713,0,0
41.4249999999987,1,549.176005841713,0,0
41.4499999999987,1,549.340255841713,0,0
41.4749999999987,1,549.504505841713,0,0
41.4999999999987,1,549.668755841714,0,0
41.5249999999987,1,549.833005841714,0,0

41.5499999999987,1,549.997255841714,0,0
41.5749999999987,1,550.161505841714,0,0
41.5999999999987,1,550.325755841714,0,0
41.6249999999987,1,550.490005841714,0,0
41.6499999999987,0,550.572130841714,0,0
41.6749999999987,1,550.654255841714,0,0
41.6999999999987,1,550.818505841714,0,0
41.7249999999987,1,550.982755841714,0,0
41.7499999999987,1,551.147005841714,0,0
41.7749999999987,1,551.311255841714,0,0
41.7999999999987,1,551.475505841714,0,0
41.8249999999987,1,551.639755841714,0,0
41.8499999999987,1,551.804005841714,0,0
41.8749999999987,1,551.968255841714,0,0
41.8999999999987,0,552.050380841714,0,0
41.9249999999987,1,552.132505841714,0,0
41.9499999999987,0,552.214630841714,0,0
41.9749999999987,1,552.296755841714,0,0
41.9999999999987,0,552.378880841714,0,0
42.0249999999987,1,552.461005841714,0,0
42.0499999999987,1,552.625255841714,0,0
42.0749999999987,0,552.707380841714,0,0
42.0999999999987,0,552.707380841714,0,0
42.1249999999987,0,552.707380841714,0,0
42.1499999999987,1,552.789505841714,0,0
42.1749999999987,1,552.953755841714,0,0
42.1999999999987,1,553.118005841714,0,0
42.2249999999987,1,553.282255841714,0,0
42.2499999999987,1,553.446505841714,0,0
42.2749999999987,1,553.610755841714,0,0
42.2999999999987,0,553.692880841714,0,0
42.3249999999987,1,553.775005841714,0,0
42.3499999999987,1,553.939255841715,0,0
42.3749999999987,1,554.103505841715,0,0
42.3999999999987,1,554.267755841715,0,0
42.4249999999987,1,554.432005841715,0,0
42.4499999999987,1,554.596255841715,0,0
42.4749999999987,1,554.760505841715,0,0
42.4999999999987,1,554.924755841715,0,0
42.5249999999987,1,555.089005841715,0,0
42.5499999999987,1,555.253255841715,0,0
42.5749999999987,1,555.417505841715,0,0
42.5999999999987,0,555.499630841715,0,0
42.6249999999987,0,555.499630841715,0,0
42.6499999999986,0,555.499630841715,0,0
42.6749999999986,1,555.581755841715,0,0
42.6999999999986,2,555.780023130525,0,0
42.7249999999986,5,556.079802501987,0,0
42.7499999999986,1,556.345564584639,0,0
42.7749999999986,1,556.509814584639,0,0
42.7999999999986,4,556.756189584639,0,0
42.8249999999986,0,556.920439584639,0,0

42.8499999999986,0,556.920439584639,0,0
42.8749999999986,0,556.920439584639,0,0
42.8999999999986,0,556.920439584639,0,0
42.9249999999986,1,557.002564584639,0,0
42.9499999999986,0,557.084689584639,0,0
42.9749999999986,0,557.084689584639,0,0
42.9999999999986,0,557.084689584639,0,0
43.0249999999986,1,557.166814584639,0,0
43.0499999999986,2,557.365081873449,0,0
43.0749999999986,1,557.563349162259,0,0
43.0999999999986,5,557.829111244911,0,0
43.1249999999986,0,558.012748327563,0,0
43.1499999999986,0,558.012748327563,0,0
43.1749999999986,3,558.154993000135,0,0
43.1999999999986,5,558.480874755359,0,0
43.2249999999986,1,558.746636838011,0,0
43.2499999999986,0,558.828761838011,0,0
43.2749999999986,1,558.910886838011,0,0
43.2999999999986,1,559.075136838011,0,0
43.3249999999986,1,559.239386838011,0,0
43.3499999999986,0,559.321511838011,0,0
43.3749999999986,0,559.321511838011,0,0
43.3999999999986,1,559.403636838011,0,0
43.4249999999986,1,559.567886838011,0,0
43.4499999999986,1,559.732136838011,0,0
43.4749999999986,1,559.896386838011,0,0
43.4999999999986,1,560.060636838011,0,0
43.5249999999986,1,560.224886838011,0,0
43.5499999999986,1,560.389136838011,0,0
43.5749999999986,1,560.553386838011,0,0
43.5999999999986,0,560.635511838011,0,0
43.6249999999986,1,560.717636838011,0,0
43.6499999999986,1,560.881886838011,0,0
43.6749999999986,1,561.046136838011,0,0
43.6999999999986,1,561.210386838011,0,0
43.7249999999986,0,561.292511838011,0,0
43.7499999999986,1,561.374636838011,0,0
43.7749999999986,1,561.538886838011,0,0
43.7999999999986,1,561.703136838011,0,0
43.8249999999986,1,561.867386838011,0,0
43.8499999999986,1,562.031636838011,0,0
43.8749999999986,1,562.195886838011,0,0
43.8999999999986,1,562.360136838012,0,0
43.9249999999986,1,562.524386838012,0,0
43.9499999999986,1,562.688636838012,0,0
43.9749999999986,1,562.852886838012,0,0
43.9999999999986,1,563.017136838012,0,0
44.0249999999986,1,563.181386838012,0,0
44.0499999999986,1,563.345636838012,0,0
44.0749999999986,1,563.509886838012,0,0
44.0999999999986,1,563.674136838012,0,0
44.1249999999986,1,563.838386838012,0,0

44.1499999999986,1,564.002636838012,0,0
44.1749999999986,1,564.166886838012,0,0
44.1999999999986,1,564.331136838012,0,0
44.2249999999986,1,564.495386838012,0,0
44.2499999999986,1,564.659636838012,0,0
44.2749999999986,0,564.741761838012,0,0
44.2999999999986,1,564.823886838012,0,0
44.3249999999986,1,564.988136838012,0,0
44.3499999999986,1,565.152386838012,0,0
44.3749999999986,1,565.316636838012,0,0
44.3999999999985,1,565.480886838012,0,0
44.4249999999985,1,565.645136838012,0,0
44.4499999999985,0,565.727261838012,0,0
44.4749999999985,1,565.809386838012,0,0
44.4999999999985,1,565.973636838012,0,0
44.5249999999985,1,566.137886838012,0,0
44.5499999999985,1,566.302136838012,0,0
44.5749999999985,0,566.384261838012,0,0
44.5999999999985,1,566.466386838012,0,0
44.6249999999985,1,566.630636838012,0,0
44.6499999999985,1,566.794886838013,0,0
44.6749999999985,1,566.959136838013,0,0
44.6999999999985,1,567.123386838013,0,0
44.7249999999985,1,567.287636838013,0,0
44.7499999999985,1,567.451886838013,0,0
44.7749999999985,1,567.616136838013,0,0
44.7999999999985,1,567.780386838013,0,0
44.8249999999985,1,567.944636838013,0,0
44.8499999999985,1,568.108886838013,0,0
44.8749999999985,1,568.273136838013,0,0
44.8999999999985,1,568.437386838013,0,0
44.9249999999985,1,568.601636838013,0,0
44.9499999999985,0,568.683761838013,0,0
44.9749999999985,1,568.765886838013,0,0
44.9999999999985,1,568.930136838013,0,0
45.0249999999985,0,569.012261838013,0,0
45.0499999999985,0,569.012261838013,0,0
45.0749999999985,0,569.012261838013,0,0
45.0999999999985,0,569.012261838013,0,0
45.1249999999985,0,569.012261838013,0,0
45.1499999999985,0,569.012261838013,0,0
45.1749999999985,0,569.012261838013,0,0
45.1999999999985,0,569.012261838013,0,0
45.2249999999985,0,569.012261838013,0,0
45.2499999999985,0,569.012261838013,0,0
45.2749999999985,0,569.012261838013,0,0
45.2999999999985,0,569.012261838013,0,0
45.3249999999985,0,569.012261838013,0,0
45.3499999999985,0,569.012261838013,0,0
45.3749999999985,0,569.012261838013,0,0
45.3999999999985,0,569.012261838013,0,0
45.4249999999985,0,569.012261838013,0,0

45.4499999999985,0,569.012261838013,0,0
45.4749999999985,1,569.094386838013,0,0
45.4999999999985,0,569.176511838013,0,0
45.5249999999985,0,569.176511838013,0,0
45.5499999999985,0,569.176511838013,0,0
45.5749999999985,0,569.176511838013,0,0
45.5999999999985,0,569.176511838013,0,0
45.6249999999985,0,569.176511838013,0,0
45.6499999999985,0,569.176511838013,0,0
45.6749999999985,0,569.176511838013,0,0
45.6999999999985,0,569.176511838013,0,0
45.7249999999985,0,569.176511838013,0,0
45.7499999999985,0,569.176511838013,0,0
45.7749999999985,0,569.176511838013,0,0
45.7999999999985,0,569.176511838013,0,0
45.8249999999985,0,569.176511838013,0,0
45.8499999999985,0,569.176511838013,0,0
45.8749999999985,0,569.176511838013,0,0
45.8999999999985,0,569.176511838013,0,0
45.9249999999985,0,569.176511838013,0,0
45.9499999999985,0,569.176511838013,0,0
45.9749999999985,0,569.176511838013,0,0
45.9999999999985,0,569.176511838013,0,0
46.0249999999985,0,569.176511838013,0,0
46.0499999999985,0,569.176511838013,0,0
46.0749999999985,0,569.176511838013,0,0
46.0999999999985,0,569.176511838013,0,0
46.1249999999985,0,569.176511838013,0,0
46.1499999999985,0,569.176511838013,0,0
46.1749999999984,0,569.176511838013,0,0
46.1999999999984,0,569.176511838013,0,0
46.2249999999984,0,569.176511838013,0,0
46.2499999999984,0,569.176511838013,0,0
46.2749999999984,0,569.176511838013,0,0
46.2999999999984,0,569.176511838013,0,0
46.3249999999984,0,569.176511838013,0,0
46.3499999999984,0,569.176511838013,0,0
46.3749999999984,0,569.176511838013,0,0
46.3999999999984,0,569.176511838013,0,0
46.4249999999984,0,569.176511838013,0,0
46.4499999999984,0,569.176511838013,0,0
46.4749999999984,0,569.176511838013,0,0
46.4999999999984,0,569.176511838013,0,0
46.5249999999984,0,569.176511838013,0,0
46.5499999999984,2,569.292654126823,0,0
46.5749999999984,0,569.408796415633,0,0
46.5999999999984,0,569.408796415633,0,0
46.6249999999984,0,569.408796415633,0,0
46.6499999999984,1,569.490921415633,0,0
46.6749999999984,0,569.573046415633,0,0
46.6999999999984,0,569.573046415633,0,0
46.7249999999984,3,569.715291088204,0,0

46.7499999999984,1,569.939660760776,0,0
46.7749999999984,1,570.103910760776,0,0
46.7999999999984,0,570.186035760776,0,0
46.8249999999984,0,570.186035760776,0,0
46.8499999999984,4,570.350285760776,0,0
46.8749999999984,5,570.698172843428,0,0
46.8999999999984,0,570.88180992608,0,0
46.9249999999984,2,570.99795221489,0,0
46.9499999999984,0,571.1140945037,0,0
46.9749999999984,0,571.1140945037,0,0
46.9999999999984,0,571.1140945037,0,0
47.0249999999984,2,571.23023679251,0,0
47.0499999999984,0,571.34637908132,0,0
47.0749999999984,0,571.34637908132,0,0
47.0999999999984,0,571.34637908132,0,0
47.1249999999984,0,571.34637908132,0,0
47.1499999999984,0,571.34637908132,0,0
47.1749999999984,0,571.34637908132,0,0
47.1999999999984,0,571.34637908132,0,0
47.2249999999984,0,571.34637908132,0,0
47.2499999999984,0,571.34637908132,0,0
47.2749999999984,0,571.34637908132,0,0
47.2999999999984,0,571.34637908132,0,0
47.3249999999984,1,571.42850408132,0,0
47.3499999999984,0,571.51062908132,0,0
47.3749999999984,0,571.51062908132,0,0
47.3999999999984,0,571.51062908132,0,0
47.4249999999984,0,571.51062908132,0,0
47.4499999999984,0,571.51062908132,0,0
47.4749999999984,0,571.51062908132,0,0
47.4999999999984,2,571.62677137013,0,0
47.5249999999984,0,571.74291365894,0,0
47.5499999999984,0,571.74291365894,0,0
47.5749999999984,0,571.74291365894,0,0
47.5999999999984,0,571.74291365894,0,0
47.6249999999984,0,571.74291365894,0,0
47.6499999999984,0,571.74291365894,0,0
47.6749999999984,0,571.74291365894,0,0
47.6999999999984,0,571.74291365894,0,0
47.7249999999984,0,571.74291365894,0,0
47.7499999999984,0,571.74291365894,0,0
47.7749999999984,0,571.74291365894,0,0
47.7999999999984,0,571.74291365894,0,0
47.8249999999984,0,571.74291365894,0,0
47.8499999999984,0,571.74291365894,0,0
47.8749999999984,0,571.74291365894,0,0
47.8999999999984,0,571.74291365894,0,0
47.9249999999983,1,571.82503865894,0,0
47.9499999999983,0,571.90716365894,0,0
47.9749999999983,0,571.90716365894,0,0
47.9999999999983,5,572.090800741592,0,0
48.0249999999983,0,572.274437824244,0,0

48.0499999999983,0,572.274437824244,0,0
48.0749999999983,0,572.274437824244,0,0
48.0999999999983,0,572.274437824244,0,0
48.1249999999983,0,572.274437824244,0,0
48.1499999999983,0,572.274437824244,0,0
48.1749999999983,0,572.274437824244,0,0
48.1999999999983,0,572.274437824244,0,0
48.2249999999983,0,572.274437824244,0,0
48.2499999999983,0,572.274437824244,0,0
48.2749999999983,0,572.274437824244,0,0
48.2999999999983,0,572.274437824244,0,0
48.3249999999983,0,572.274437824244,0,0
48.3499999999983,0,572.274437824244,0,0
48.3749999999983,0,572.274437824244,0,0
48.3999999999983,0,572.274437824244,0,0
48.4249999999983,0,572.274437824244,0,0
48.4499999999983,0,572.274437824244,0,0
48.4749999999983,0,572.274437824244,0,0
48.4999999999983,0,572.274437824244,0,0
48.5249999999983,0,572.274437824244,0,0
48.5499999999983,0,572.274437824244,0,0
48.5749999999983,0,572.274437824244,0,0
48.5999999999983,0,572.274437824244,0,0
48.6249999999983,0,572.274437824244,0,0
48.6499999999983,0,572.274437824244,0,0
48.6749999999983,0,572.274437824244,0,0
48.6999999999983,0,572.274437824244,0,0
48.7249999999983,0,572.274437824244,0,0
48.7499999999983,0,572.274437824244,0,0
48.7749999999983,0,572.274437824244,0,0
48.7999999999983,0,572.274437824244,0,0
48.8249999999983,0,572.274437824244,0,0
48.8499999999983,0,572.274437824244,0,0
48.8749999999983,0,572.274437824244,0,0
48.8999999999983,0,572.274437824244,0,0
48.9249999999983,0,572.274437824244,0,0
48.9499999999983,0,572.274437824244,0,0
48.9749999999983,0,572.274437824244,0,0
48.9999999999983,0,572.274437824244,0,0
49.0249999999983,0,572.274437824244,0,0
49.0499999999983,0,572.274437824244,0,0
49.0749999999983,0,572.274437824244,0,0
49.0999999999983,0,572.274437824244,0,0
49.1249999999983,0,572.274437824244,0,0
49.1499999999983,0,572.274437824244,0,0
49.1749999999983,0,572.274437824244,0,0
49.1999999999983,0,572.274437824244,0,0
49.2249999999983,0,572.274437824244,0,0
49.2499999999983,0,572.274437824244,0,0
49.2749999999983,0,572.274437824244,0,0
49.2999999999983,0,572.274437824244,0,0
49.3249999999983,0,572.274437824244,0,0

49.3499999999983,0,572.274437824244,0,0
49.3749999999983,0,572.274437824244,0,0
49.3999999999983,0,572.274437824244,0,0
49.4249999999983,0,572.274437824244,0,0
49.4499999999983,0,572.274437824244,0,0
49.4749999999983,0,572.274437824244,0,0
49.4999999999983,0,572.274437824244,0,0
49.5249999999983,1,572.356562824244,0,0
49.5499999999983,0,572.438687824244,0,0
49.5749999999983,1,572.520812824244,0,0
49.5999999999983,1,572.685062824244,0,0
49.6249999999983,0,572.767187824244,0,0
49.6499999999983,1,572.849312824244,0,0
49.6749999999982,0,572.931437824244,0,0
49.6999999999982,1,573.013562824244,0,0
49.7249999999982,0,573.095687824244,0,0
49.7499999999982,0,573.095687824244,0,0
49.7749999999982,0,573.095687824244,0,0
49.7999999999982,1,573.177812824244,0,0
49.8249999999982,0,573.259937824244,0,0
49.8499999999982,0,573.259937824244,0,0
49.8749999999982,0,573.259937824244,0,0
49.8999999999982,0,573.259937824244,0,0
49.9249999999982,0,573.259937824244,0,0
49.9499999999982,0,573.259937824244,0,0
49.9749999999982,0,573.259937824244,0,0
49.9999999999982,1,573.342062824244,0,0
50.0249999999982,0,573.424187824244,0,0
50.0499999999982,0,573.424187824244,0,0
50.0749999999982,0,573.424187824244,0,0
50.0999999999982,0,573.424187824244,0,0
50.1249999999982,0,573.424187824244,0,0
50.1499999999982,0,573.424187824244,0,0
50.1749999999982,0,573.424187824244,0,0
50.1999999999982,0,573.424187824244,0,0
50.2249999999982,0,573.424187824244,0,0
50.2499999999982,0,573.424187824244,0,0
50.2749999999982,0,573.424187824244,0,0
50.2999999999982,0,573.424187824244,0,0
50.3249999999982,0,573.424187824244,0,0
50.3499999999982,0,573.424187824244,0,0
50.3749999999982,0,573.424187824244,0,0
50.3999999999982,0,573.424187824244,0,0
50.4249999999982,0,573.424187824244,0,0
50.4499999999982,0,573.424187824244,0,0
50.4749999999982,0,573.424187824244,0,0
50.4999999999982,0,573.424187824244,0,0
50.5249999999982,0,573.424187824244,0,0
50.5499999999982,0,573.424187824244,0,0
50.5749999999982,3,573.566432496816,0,0
50.5999999999982,5,573.89231425204,0,0
50.6249999999982,4,574.240201334692,0,0

50.6499999999982,0,574.404451334692,0,0
50.6749999999982,1,574.486576334692,0,0
50.6999999999982,5,574.752338417344,0,0
50.7249999999982,0,574.935975499996,0,0
50.7499999999982,0,574.935975499996,0,0
50.7749999999982,5,575.119612582648,0,0
50.7999999999982,0,575.303249665301,0,0
50.8249999999982,0,575.303249665301,0,0
50.8499999999982,0,575.303249665301,0,0
50.8749999999982,2,575.41939195411,0,0
50.8999999999982,0,575.53553424292,0,0
50.9249999999982,0,575.53553424292,0,0
50.9499999999982,0,575.53553424292,0,0
50.9749999999982,0,575.53553424292,0,0
50.9999999999982,0,575.53553424292,0,0
51.0249999999982,0,575.53553424292,0,0
51.0499999999982,1,575.61765924292,0,0
51.0749999999982,0,575.69978424292,0,0
51.0999999999982,0,575.69978424292,0,0
51.1249999999982,0,575.69978424292,0,0
51.1499999999982,0,575.69978424292,0,0
51.1749999999982,0,575.69978424292,0,0
51.1999999999982,0,575.69978424292,0,0
51.2249999999982,0,575.69978424292,0,0
51.2499999999982,0,575.69978424292,0,0
51.2749999999982,0,575.69978424292,0,0
51.2999999999982,0,575.69978424292,0,0
51.3249999999982,0,575.69978424292,0,0
51.3499999999982,0,575.69978424292,0,0
51.3749999999982,0,575.69978424292,0,0
51.3999999999982,0,575.69978424292,0,0
51.4249999999982,1,575.78190924292,0,0
51.4499999999981,0,575.86403424292,0,0
51.4749999999981,0,575.86403424292,0,0
51.4999999999981,0,575.86403424292,0,0
51.5249999999981,0,575.86403424292,0,0
51.5499999999981,0,575.86403424292,0,0
51.5749999999981,0,575.86403424292,0,0
51.5999999999981,0,575.86403424292,0,0
51.6249999999981,0,575.86403424292,0,0
51.6499999999981,0,575.86403424292,0,0
51.6749999999981,0,575.86403424292,0,0
51.6999999999981,0,575.86403424292,0,0
51.7249999999981,0,575.86403424292,0,0
51.7499999999981,0,575.86403424292,0,0
51.7749999999981,0,575.86403424292,0,0
51.7999999999981,0,575.86403424292,0,0
51.8249999999981,0,575.86403424292,0,0
51.8499999999981,0,575.86403424292,0,0
51.8749999999981,0,575.86403424292,0,0
51.8999999999981,0,575.86403424292,0,0
51.9249999999981,0,575.86403424292,0,0

51.9499999999981,0,575.86403424292,0,0
51.9749999999981,0,575.86403424292,0,0
51.9999999999981,0,575.86403424292,0,0
52.0249999999981,0,575.86403424292,0,0
52.0499999999981,0,575.86403424292,0,0
52.0749999999981,0,575.86403424292,0,0
52.0999999999981,0,575.86403424292,0,0
52.1249999999981,0,575.86403424292,0,0
52.1499999999981,0,575.86403424292,0,0
52.1749999999981,0,575.86403424292,0,0
52.1999999999981,0,575.86403424292,0,0
52.2249999999981,0,575.86403424292,0,0
52.2499999999981,0,575.86403424292,0,0
52.2749999999981,0,575.86403424292,0,0
52.2999999999981,0,575.86403424292,0,0
52.3249999999981,0,575.86403424292,0,0
52.3499999999981,0,575.86403424292,0,0
52.3749999999981,0,575.86403424292,0,0
52.3999999999981,0,575.86403424292,0,0
52.4249999999981,0,575.86403424292,0,0
52.4499999999981,0,575.86403424292,0,0
52.4749999999981,0,575.86403424292,0,0
52.4999999999981,0,575.86403424292,0,0
52.5249999999981,0,575.86403424292,0,0
52.5499999999981,0,575.86403424292,0,0
52.5749999999981,0,575.86403424292,0,0
52.5999999999981,0,575.86403424292,0,0
52.6249999999981,0,575.86403424292,0,0
52.6499999999981,0,575.86403424292,0,0
52.6749999999981,1,575.94615924292,0,0
52.6999999999981,0,576.02828424292,0,0
52.7249999999981,0,576.02828424292,0,0
52.7499999999981,0,576.02828424292,0,0
52.7749999999981,0,576.02828424292,0,0
52.7999999999981,0,576.02828424292,0,0
52.8249999999981,0,576.02828424292,0,0
52.8499999999981,0,576.02828424292,0,0
52.8749999999981,0,576.02828424292,0,0
52.8999999999981,0,576.02828424292,0,0
52.9249999999981,0,576.02828424292,0,0
52.9499999999981,0,576.02828424292,0,0
52.9749999999981,0,576.02828424292,0,0
52.9999999999981,0,576.02828424292,0,0
53.0249999999981,0,576.02828424292,0,0
53.0499999999981,0,576.02828424292,0,0
53.0749999999981,0,576.02828424292,0,0
53.0999999999981,0,576.02828424292,0,0
53.1249999999981,0,576.02828424292,0,0
53.1499999999981,0,576.02828424292,0,0
53.1749999999981,0,576.02828424292,0,0
53.199999999998,0,576.02828424292,0,0
53.224999999998,0,576.02828424292,0,0

53.249999999998,0,576.02828424292,0,0
53.274999999998,1,576.11040924292,0,0
53.299999999998,0,576.19253424292,0,0
53.324999999998,0,576.19253424292,0,0
53.349999999998,0,576.19253424292,0,0
53.374999999998,0,576.19253424292,0,0
53.399999999998,1,576.274659242921,0,0
53.424999999998,1,576.438909242921,0,0
53.449999999998,1,576.603159242921,0,0
53.474999999998,3,576.827528915492,0,0
53.499999999998,4,577.134023588064,0,0
53.524999999998,6,577.49943793319,0,0
53.549999999998,10,577.960304331157,67.670126039248,1
53.574999999998,13,578.516112282496,67.670126039248,1
53.599999999998,17,579.150828230497,67.670126039248,1
53.624999999998,23,579.883295943853,67.670126039248,1
53.649999999998,27,580.70388762542,67.670126039248,1
53.674999999998,30,581.580438793486,67.670126039248,1
53.699999999998,33,582.502028151184,67.670126039248,1
53.724999999998,33,583.445572565878,67.670126039248,1
53.749999999998,36,584.410094773225,67.670126039248,1
53.774999999998,39,585.415715233843,67.670126039248,1
53.799999999998,42,586.460816524319,67.670126039248,1
53.824999999998,46,587.55004620404,67.670126039248,1
53.849999999998,49,588.681920053904,67.670126039248,1
53.874999999998,51,589.843284863598,67.670126039248,1
53.899999999998,53,591.027653697949,67.670126039248,1
53.924999999998,55,592.234588023359,67.670126039248,1
53.949999999998,57,593.463673477108,67.670126039248,1
53.974999999998,59,594.714517724649,67.670126039248,1
53.999999999998,65,596.007444736771,67.670126039248,1
54.024999999998,67,597.34177975074,67.670126039248,1
54.049999999998,71,598.705999397253,67.670126039248,1
54.074999999998,71,600.089994497497,67.670126039248,1
54.099999999998,75,601.493215410477,67.670126039248,1
54.124999999998,81,602.943563773335,67.670126039248,1
54.149999999998,83,604.430883131022,67.670126039248,1
54.174999999998,87,605.945088493444,67.670126039248,1
54.199999999998,87,607.477110502914,67.670126039248,1
54.224999999998,92,609.030836835353,67.670126039248,1
54.249999999998,94,610.614783579638,67.670126039248,1
54.274999999998,95,612.211470481786,67.670126039248,1
54.299999999998,101,613.837272002738,67.670126039248,1
54.324999999998,101,615.487964073507,67.670126039248,1
54.349999999998,110,617.174644375451,67.670126039248,1
54.374999999998,114,618.912833693459,67.670126039248,1
54.399999999998,109,620.647098916951,67.670126039248,1
54.424999999998,114,622.381364140444,67.670126039248,1
54.449999999998,112,624.127348497576,67.670126039248,1
54.474999999998,120,625.896112103963,67.670126039248,1
54.499999999998,123,627.706556715255,67.670126039248,1
54.524999999998,134,629.568032631485,67.670126039248,1

54.549999999998,124,631.433203533722,67.670126039248,1
54.574999999998,128,633.276847140796,67.670126039248,1
54.599999999998,134,635.156651056916,67.670126039248,1
54.624999999998,127,637.032818409923,67.670126039248,1
54.649999999998,130,638.89468922515,67.670126039248,1
54.674999999998,131,640.771021881071,67.670126039248,1
54.699999999998,131,642.650949057187,67.670126039248,1
54.724999999998,130,644.527281713108,67.670126039248,1
54.749999999998,129,646.396411476768,67.670126039248,1
54.774999999998,130,648.265541240428,67.670126039248,1
54.799999999998,132,650.145454722985,67.670126039248,1
54.824999999998,135,652.0432054096,67.670126039248,1
54.849999999998,129,653.93017237732,67.670126039248,1
54.874999999998,128,655.792071383597,67.670126039248,1
54.899999999998,129,657.653970389874,67.670126039248,1
54.924999999998,135,659.540937357593,67.670126039248,1
54.9499999999979,134,661.445809235157,67.670126039248,1
54.9749999999979,143,663.378547004326,67.670126039248,1
54.9999999999979,140,665.332335272227,67.670126039248,1
55.0249999999979,136,667.26178522533,67.670126039248,1
55.0499999999979,142,669.198151394569,67.670126039248,1
55.0749999999979,137,671.138032195249,67.670126039248,1
55.0999999999979,144,673.084780675417,67.670126039248,1
55.1249999999979,139,675.038520145731,67.670126039248,1
55.1499999999979,136,676.964493464774,67.670126039248,1
55.1749999999979,135,678.876433585424,67.670126039248,1
55.1999999999979,140,680.80235596172,67.670126039248,1
55.2249999999979,134,682.724737671736,67.670126039248,1
55.2499999999979,139,684.643642747692,67.670126039248,1
55.2749999999979,137,686.573130698175,67.670126039248,1
55.2999999999979,133,688.48149088143,67.670126039248,1
55.3249999999979,132,690.372146999211,67.670126039248,1
55.3499999999979,131,692.255655001963,67.670126039248,1
55.3749999999979,130,694.131987657884,67.670126039248,1
55.3999999999979,135,696.022562997668,67.670126039248,1
55.4249999999979,135,697.930975541512,67.670126039248,1
55.4499999999979,134,699.835847419076,67.670126039248,1
55.4749999999979,128,701.715651335196,67.670126039248,1
55.4999999999979,133,703.591901348763,67.670126039248,1
55.5249999999979,138,705.503763359572,67.670126039248,1
55.5499999999979,134,707.419179272936,67.670126039248,1
55.5749999999979,136,709.327578727306,67.670126039248,1
55.5999999999979,130,711.221681643897,67.670126039248,1
55.6249999999979,129,713.090811407557,67.670126039248,1
55.6499999999979,135,714.977778375277,67.670126039248,1
55.6749999999979,131,716.871948235257,67.670126039248,1
55.6999999999979,126,718.733762661981,67.670126039248,1
55.7249999999979,130,720.591982568511,67.670126039248,1
55.7499999999979,131,722.468315224431,67.670126039248,1
55.7749999999979,129,724.341039508287,67.670126039248,1
55.7999999999979,128,726.202938514564,67.670126039248,1
55.8249999999979,123,728.042887135632,67.670126039248,1

55.8499999999979,124,729.868202742816,67.670126039248,1
55.8749999999979,120,731.682342340113,67.670126039248,1
55.8999999999979,121,733.485351640815,67.670126039248,1
55.9249999999979,119,735.284604623229,67.670126039248,1
55.9499999999979,122,737.08758287918,67.670126039248,1
55.9749999999979,121,738.898058152717,67.670126039248,1
55.9999999999979,122,740.708533426253,67.670126039248,1
56.0249999999979,120,742.515268000492,67.670126039248,1
56.0499999999979,113,744.287903026065,67.670126039248,1
56.0749999999979,118,746.053009598776,67.670126039248,1
56.0999999999979,121,747.848490446616,67.670126039248,1
56.1249999999979,119,749.64774342903,67.670126039248,1
56.1499999999979,123,751.454431722034,67.670126039248,1
56.1749999999979,125,753.283427445883,67.670126039248,1
56.1999999999979,125,755.119798272405,67.670126039248,1
56.2249999999979,125,756.956169098927,67.670126039248,1
56.2499999999979,117,758.76267220768,67.670126039248,1
56.2749999999979,115,760.531682413005,67.670126039248,1
56.2999999999979,121,762.315749922837,67.670126039248,1
56.3249999999979,116,764.103638242409,67.670126039248,1
56.3499999999979,119,765.884029544396,67.670126039248,1
56.3749999999979,118,767.67201337465,67.670126039248,1
56.3999999999979,118,769.456225070329,67.670126039248,1
56.4249999999979,116,771.232844237741,67.670126039248,1
56.4499999999979,118,773.009463405153,67.670126039248,1
56.4749999999979,121,774.804944252993,67.670126039248,1
56.4999999999979,121,776.611694252993,67.670126039248,1
56.5249999999979,123,778.425879563581,67.670126039248,1
56.5499999999979,124,780.251195170765,67.670126039248,1
56.5749999999979,123,782.076510777949,67.670126039248,1
56.5999999999979,125,783.905506501799,67.670126039248,1
56.6249999999979,124,785.738197211654,67.670126039248,1
56.6499999999979,120,787.552336808951,67.670126039248,1
56.6749999999979,125,789.370156522915,67.670126039248,1
56.6999999999979,126,791.210192774842,67.670126039248,1
56.7249999999978,127,793.057545360873,67.670126039248,1
56.7499999999978,126,794.904897946904,67.670126039248,1
56.7749999999978,129,796.759509481368,67.670126039248,1
56.7999999999978,123,798.603080487755,67.670126039248,1
56.8249999999978,127,800.439392545708,67.670126039248,1
56.8499999999978,126,802.286745131739,67.670126039248,1
56.8749999999978,123,804.119406280994,67.670126039248,1
56.8999999999978,126,805.95206743025,67.670126039248,1
56.9249999999978,129,807.806678964714,67.670126039248,1
56.9499999999978,124,809.653944957106,67.670126039248,1
56.9749999999978,126,811.490301092368,67.670126039248,1
56.9999999999978,125,813.330337344295,67.670126039248,1
57.0249999999978,130,815.184891825418,67.670126039248,1
57.0499999999978,122,817.028361166817,67.670126039248,1
57.0749999999978,129,818.868222136152,67.670126039248,1
57.0999999999978,124,820.715488128544,67.670126039248,1
57.1249999999978,118,822.522099272979,67.670126039248,1

57.1499999999978,123,824.325015431408,67.670126039248,1
57.1749999999978,117,826.124143437489,67.670126039248,1
57.1999999999978,121,827.915836132981,67.670126039248,1
57.2249999999978,115,829.699903642814,67.670126039248,1
57.2499999999978,115,831.461288662479,67.670126039248,1
57.2749999999978,114,833.218836223759,67.670126039248,1
57.2999999999978,108,834.949159310637,67.670126039248,1
57.3249999999978,108,836.656095381496,67.670126039248,1
57.3499999999978,102,838.338985259988,67.670126039248,1
57.3749999999978,99,839.985540535775,67.670126039248,1
57.3999999999978,93,841.594658787246,67.670126039248,1
57.4249999999978,87,843.152654610727,67.670126039248,1
57.4499999999978,64,844.575665615462,67.670126039248,1
57.4749999999978,45,845.783576863419,67.670126039248,1
57.4999999999978,14,846.641771724264,67.670126039248,1
57.5249999999978,0,846.949055337153,67.670126039248,1
57.5499999999978,1,847.031180337153,0,0
57.5749999999978,0,847.113305337153,0,0
57.5999999999978,1,847.195430337153,0,0
57.6249999999978,0,847.277555337153,0,0
57.6499999999978,1,847.359680337153,0,0
57.6749999999978,1,847.523930337153,0,0
57.6999999999978,0,847.606055337153,0,0
57.7249999999978,0,847.606055337153,0,0
57.7499999999978,1,847.688180337153,0,0
57.7749999999978,0,847.770305337153,0,0
57.7999999999978,1,847.852430337153,0,0
57.8249999999978,0,847.934555337153,0,0
57.8499999999978,0,847.934555337153,0,0
57.8749999999978,0,847.934555337153,0,0
57.8999999999978,0,847.934555337153,0,0
57.9249999999978,0,847.934555337153,0,0
57.9499999999978,1,848.016680337153,0,0
57.9749999999978,1,848.180930337153,0,0
57.9999999999978,0,848.263055337153,0,0
58.0249999999978,0,848.263055337153,0,0
58.0499999999978,1,848.345180337153,0,0
58.0749999999978,0,848.427305337153,0,0
58.0999999999978,0,848.427305337153,0,0
58.1249999999978,1,848.509430337153,0,0
58.1499999999978,1,848.673680337153,0,0
58.1749999999978,0,848.755805337153,0,0
